# Oral and anal microbiome from HIV-exposed individuals: role of host-associated factors in taxa composition and metabolic pathways

**DOI:** 10.1038/s41522-023-00413-4

**Published:** 2023-07-12

**Authors:** Ezequiel Lacunza, Valeria Fink, María E. Salas, Romina Canzoneri, Julián Naipauer, Sion Williams, Omar Coso, Omar Sued, Pedro Cahn, Enrique A. Mesri, Martín C. Abba

**Affiliations:** 1 Centro de Investigaciones Inmunológicas Básicas y Aplicadas (CINIBA), Facultad de Ciencias Médicas, Universidad Nacional de La Plata, La Plata, Argentina; 2 Dirección de Investigaciones, Fundación Huésped, Buenos Aires, Argentina; 3 Instituto de Fisiología, Biología Molecular y Neurociencias (IFIBYNE), CONICET - Universidad de Buenos Aires, Buenos Aires, Argentina; 4 University of Miami - Center for AIDS Research (UM-CFAR) / Sylvester Comprehensive Cancer Center (CCC), University of Miami Miller School of Medicine, Miami, FL USA; 5 Pan American Health Organization, Washington, USA

**Keywords:** Infectious-disease diagnostics, Microbial genetics

## Abstract

Evidence indicates that the microbiome plays a significant role in HIV immunopathogenesis and associated complications. This study aimed to characterize the oral and anal microbiome of Men who have Sex with Men (MSM) and Transgender Women (TGW), with and without HIV. One hundred and thirty oral and anal DNA-derived samples were obtained from 78 participants and subjected to shotgun metagenomics sequencing for further microbiome analysis. Significant differences in the microbiome composition were found among subjects associated with HIV infection, gender, sex behavior, CD4+ T-cell counts, antiretroviral therapy (ART), and the presence of HPV-associated precancerous anal lesions. Results confirm the occurrence of oncogenic viromes in this high HIV-risk population. The oral microbiome in HIV-associated cases exhibited an enrichment of bacteria associated with periodontal disease pathogenesis. Conversely, anal bacteria showed a significant decrease in HIV-infected subjects *(Coprococcus comes, Finegoldia magna, Blautia obeum, Catenibacterium mitsuokai)*. TGW showed enrichment in species related to sexual transmission, which concurs that most recruited TGW are or have been sex workers. *Prevotella bivia and Fusobacterium gonidiaformans* were positively associated with anal precancerous lesions among HIV-infected subjects. The enrichment of *Holdemanella biformis* and *C. comes* was associated with detectable viral load and ART-untreated patients. Metabolic pathways were distinctly affected by predominant factors linked to sexual behavior or HIV pathogenesis. Gene family analysis identified bacterial gene signatures as potential prognostic and predictive biomarkers for HIV/AIDS-associated malignancies. Conclusions: Identified microbial features at accessible sites are potential biomarkers for predicting precancerous anal lesions and therapeutic targets for HIV immunopathogenesis.

## Introduction

The human body harbors complex microbial communities that ensure vital functions for the host and may affect health and disease susceptibility by modulating physiological homeostasis, energy metabolism, and immune-related bioprocess^1,2^. The gut and oral microbiomes constitute the first and the second largest microbial communities in humans, and their habitat—the mucosal immune system—is devastated after acute HIV infection^3,4^. Emergent evidence in recent years has indicated that the microbiome plays a significant role in human immunodeficiency virus (HIV-1) immunopathogenesis and associated complications^5–10^. A relevant subset of such complications includes virally induced squamous intraepithelial lesions (SILs) and HIV/AIDS-associated malignancies^8–11^.

Latin America has a high prevalence of HIV/AIDS-associated malignancies caused by viruses (including HPV, EBV, and KSHV) which tend to disproportionally affect underprivileged and socially vulnerable populations, such as men who have sex with men (MSM), transgender women (TGW), commercial sex workers (CSW) and people who inject drugs^12,13^. Worldwide, TGW have an extremely high HIV prevalence compared to other adults, with almost 50 times higher odds of infection^14^. In Argentina, the burden of HIV infection among MSM is 14%, while in TGW has been reported as high as 34% compared to 0.4% in the general population^15^. In addition to biological factors, stigma, discrimination and structural determinants increase the risks for HIV infection and other sexually transmitted infections and represent barriers to care. In particular, TGW suffer social exclusion at an early age, limiting their access to education and jobs, leading many TGW to engage in sex work^16,17^.

MSM and TGW populations have the highest risk for infection with KSHV and HPV, potentially developing Kaposi sarcoma (KS) and/or SILs and anal cancer. For AIDS-associated malignancies such as KS, the effect of antiretroviral therapy (ART) has been dramatic, with significant decreases in incidence and outcomes improvement. However, for other AIDS malignancies such as HPV-associated anal cancer, ART appears not to affect the natural history of the malignancy.

Beyond the oncogenic viruses, increasing evidence reveals that the microbiota may play a key role in carcinogenesis by affecting the balance of host cell proliferation and apoptosis, hindering anti-tumoral immunity, and influencing the metabolism of host-produced factors, ingested food components, and drugs^18^. However, limited HIV microbiome studies have been conducted using whole metagenome sequencing to define the compositional and functional consequences of HIV infection on the oral and anal microbiota at the species level^19^. Importantly, no study to date has comprehensively delineated the oral and anal microbiome portrait of TGW with and without HIV, which is the primary aim of the present study^20^.

Here we performed a metagenomics-based characterization of the oral and anal microbiome in a cohort of MSM and TGW patients exposed to HIV infection to investigate their relationship with associated factors such as gender, sexual behavior, ART, viral load, CD4+ T-cell counts and the presence of precancerous anal intraepithelial lesions.

## Results

### Richness and diversity

**Fig. 1:**
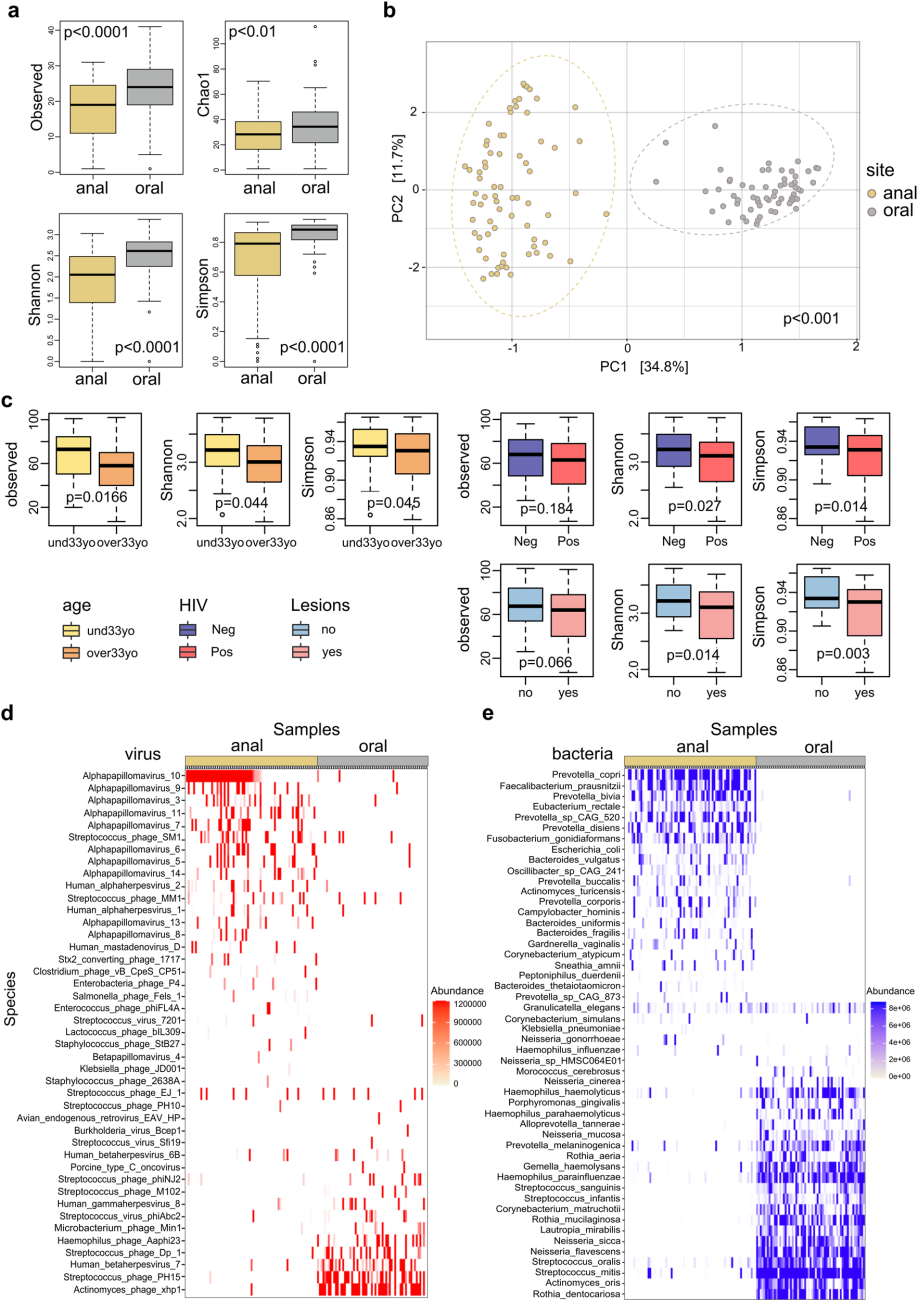
Richness, diversity, and taxonomy profile of oral and anal samples obtained from TGW and MSM. **a** Alpha diversity indices show a greater richness in oral samples compared with anal samples. The p-values displayed on the box plots represent the results of the Wilcoxon test. **b** Beta diversity shows two main clusters based on sample origin, which determine two locations completely different regarding microbial composition, with a higher inter-sample variability among anal samples assessed by Aitchison distance and PERMANOVA test (*p* < 0.001, Supplementary Fig. 2) **c** Box plots of alpha diversity indices of the variables age, HIV status and lesions in anal samples. A decrease in alpha diversity is observed in the group of older subjects, HIV-positive patients and subjects with lesions (*p* < 0.05). The *p*-values displayed on the box plots represent the results of the Wilcoxon test. **d**, **e** Heatmap representations of the most prevalent virus (**d**) and bacteria (**e**) identified in oral and anal samples. In both heatmaps taxa are ordered according to prevalence and abundance; samples were clustered by an unsupervised method. Dominant and mutually exclusive taxa were identified for both locations.

One hundred and thirty oral and anal DNA-derived samples were obtained from 78 participants (47 MSM and 31 TGW), 50 were HIV-positive and 28 were HIV-negative. When recruited, most of the participants with HIV were on antiretroviral therapy (Table 1 and Supplementary Table 1). We first tested whether microbial richness and diversity differed between anal and oral groups of samples across the different variables. For alpha diversity, there was a significant difference among oral and anal samples, with a greater richness in the distribution of the abundance of species in the oral ones, as indicated by Chao1, Shannon and Simpson indices (*p* < 0.01; Fig. 1a). As expected, beta diversity shows two main clusters based on sample origin, which determine two locations completely different regarding microbial composition (Fig. 1b). We further evaluated whether the samples cluster beyond that expected by sampling variability using PERMANOVA. There was a significantly greater dispersion of samples from the anal site, with higher inter-sample distance, compared with the oral site (*p* < 0.001, Supplementary Fig. 1).

**Table 1:** Participants, samples, and clinical variables included in the study.

Participants	MSM	TGW	TOTAL
*N*	47	31	78
AGE (Mean-Median-SD)	(34.8-33-9.5)	(34.0-32.3-8.2)	(34.5-33-8.9)
*Samples*			
Oral swabs	41	19	60
Anal swabs	42	28	70
Total	83	47	130
*HIV status*			
Positive	34	16	50
Negative	13	15	28
*Current viral load*			
Detectable (>50 copies/ml)	7	5	12
Undetectable (<50 copies/ml)	23	7	30
ND	4	4	8
*ART*			
Yes	29	15	44
No	5	1	6
*Current CD4+ T-cell counts*			
<500 (cells/mm^3^)	12	5	17
>500 (cells/mm^3^)	20	5	26
ND	2	5	7
*Cytology*			
Lesions	34	17	51
*ASCUS*	1	2	3
*LSILs*	28	12	40
*HSILs*	5	3	8
Normal	13	14	27

*ND* no data.

Richness estimation across the variables gender, age, CSW, HIV, ART, CD4+ T-cell counts, and the presence of lesions, was analyzed considering both sites separately. In anal samples, there were no differences in richness between both genders (MSM and TGW) nor in the CSW, CD4+ T-cell counts and ART groups (Supplementary Table 2). However, we found significant differences in aging (under33yo vs. over33yo), HIV status (positive vs. negative), and the presence of lesions (yes vs. no), with lower indices in the group of older subjects (over33yo; Observed, Shannon and Simpson indices, *p* < 0.05), HIV-positive patients (Shannon and Simpson, *p* < 0.05) and in the group of patients with lesions (Shannon and Simpson, *p* < 0.05) (Fig. 1c; Supplementary Table 2). We observed no differences in the analyzed variables when considering oral samples (*p* > 0.05; Supplementary Table 2).

These results suggest that aging, HIV, and the presence of lesions are associated with changes in the anal microbiome composition. However, further analysis is required to determine any potential interconnectedness between these variables contributing to microbial richness and diversity.

### Taxonomic composition and abundance

To define the taxonomic composition of the 130 samples, we used MetaPhlAn 3.0 software. The analysis identified 666 species detected at least once, of which 314 were considered “prevalent” when defined as present in >5% of samples at >0.1% relative abundance (Supplementary Table 3). Of the 666 species, 548 belong to the kingdom Bacteria, 113 to viruses, 4 to eukaryotes and 1 to Archaea. Of the prevalent species, 284 are bacteria, 28 viruses, 1 eukaryote and 1 archaea. Of all detected species, we found a similar number of taxa significantly associated with oral (*n* = 200) or anal sites (*n* = 203), according to the MaAsLin 2.0 default significance levels (*q* value < 0.25; Supplementary Table 3).

To visualize the relative abundance of taxa, we first evaluated the abundances at phylum level for site, gender, aging, CSW, HIV status and the presence of lesions (Supplementary Fig. 2). *Firmicutes*, *Bacteroidetes*, *Actinobacteria*, *Proteobacteria*, and *Fusobacteria* were the most prevalent phyla (Supplementary Fig. 2). We conducted a multivariate test for differences in the overall composition between groups of samples using the HMP package. Significant differences were observed among sites, with a higher prevalence of *Actinobacteria* (*p* < 0.0001), *Firmicutes* (*p* < 0.0001), and *Proteobacteria* (*p* < 0.0001) in oral samples. In contrast, *Bacteroidetes* (*p* < 0.0001), *Spirochaetes* (*p* < 0.01), the unusual group *Tenericutes* (*p* < 0.01) and *Viruses* (*p* < 0.001) were most abundant in anal samples (Supplementary Fig. 2). Interestingly, there was an enrichment of *Actinobacteria* in oral samples of older subjects (*p* = 0.009), while *Proteobacteria* (oral samples, *p* = 0.01) and *Fusobacteria* (anal samples, *p* = 0.001) were more abundant in the youngers (Supplementary Fig. 2). For HIV group, only *Viruses* yielded a positive association with anal samples of the infected patients (*p* = 0.038, Additional file 5). The presence of lesions was positively associated with *Viruses* in anal samples and *Proteobacteria* in oral samples (Supplementary Fig. 2). Conversely, a decrease of *Firmicutes* (*p* = 0.0069) and *Actinobacteria* (*p* = 0.0001) was observed in anal samples (Supplementary Fig. 2). Finally, no differences were observed between groups of gender or CSW condition (data not shown).

Next, we defined the most abundant taxa at the species level for viruses and bacteria in oral and anal sites (Pr > 5%, abundance >0.15). A few dominant and mutually exclusive taxa were identified in both locations. For viruses, species of *Alphapapillomavirus* were prevalent in anal samples, while *Human gammaherpesvirus 8* (KSHV) and Human *betaherpesvirus 7* (HHV-7) along with bacteriophages of *Actynomyces* and *Streptococcus*, among others, were dominant in oral samples (Fig. 1d). In Bacteria, oral samples were enriched by species of *Haemophilus* (*H. influenzae, H. parainfluenzae, H. haemolyticus, H. parahaemolyticus*), *Neisseria* (*N. flavescens, N. mucosa, N. sicca*), *Rothia* (*R. mucilaginosa, R. dentocariosa*) and *Streptococcus* (*S oralis, S mitis*) along with other relevant species such as *Lautropia mirabilis*, *Granullicatella elegans*, *Gemella hemolysans* or *Corynebacterium matruchotii*. In anal samples, species of *Prevotella* (*P. copri, P. corporis, P. bivia, P. disiens*, etc.) and *Bacteroides* (*B. vulgatus, B. fragilis, B. uniformis*, etc.) were among the most abundant species. Moreover, other frequent bacteria in the anal samples were *Eubacterium rectale*, *Faecalybacteriun prausnitzii* or *Fusobacterium gonidiaformans*, among others (Fig. 1e).

Overall, distinctive microbial compositions were identified in oral and anal mucosa of a cohort of MSM and TGW HIV-positive and negative cases, with a greater richness at the species level in the oral samples. According to the alpha indices, the dysbiosis observed in anal samples due to the HIV infection, aging and the presence of lesions could partly explain the more inter-sample variability of this location compared with oral mucosa.

### Taxonomic profile in a multivariable association between phenotypes and microbial species

**Fig. 2:**
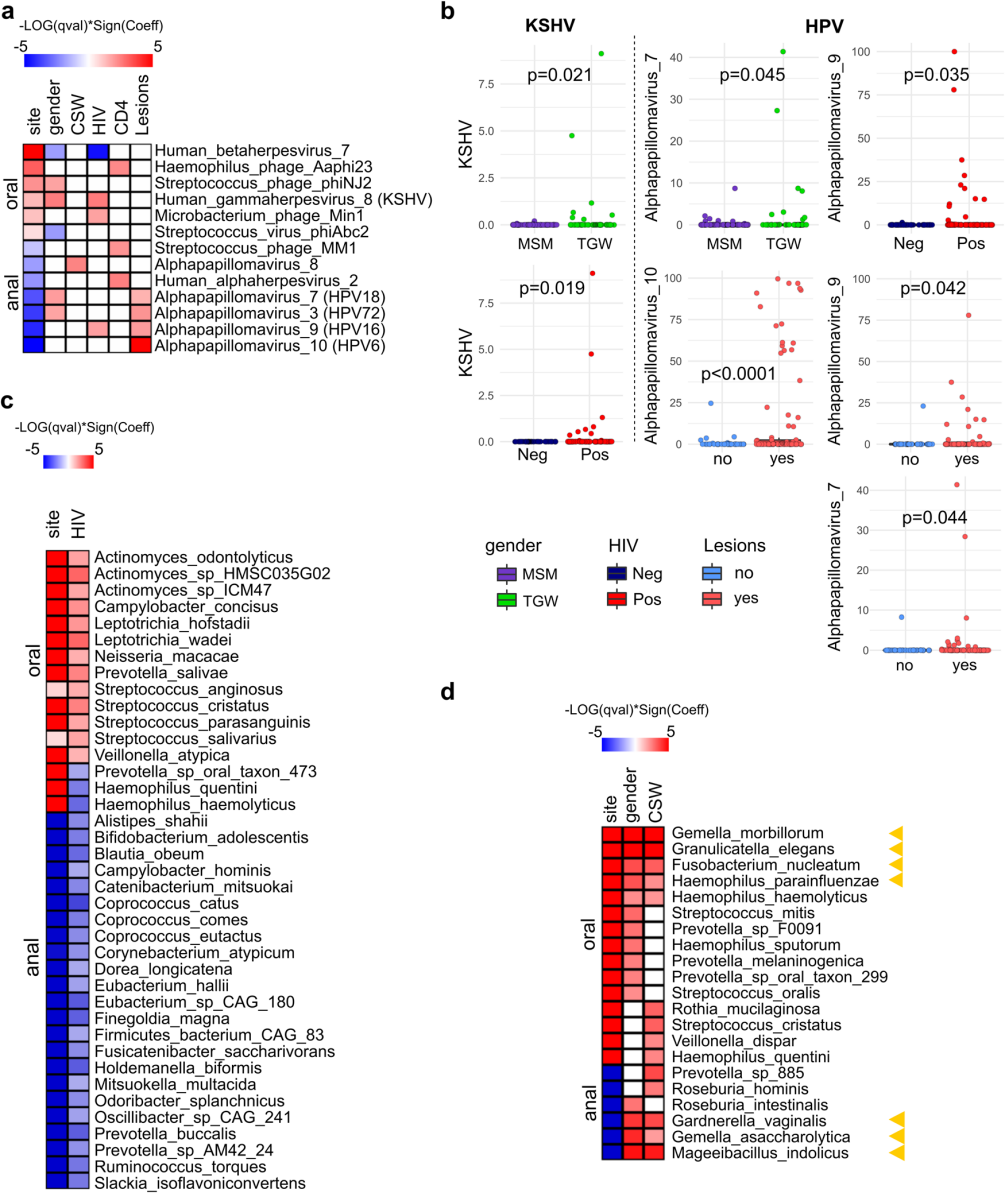
Top significant taxa associations (*p* < 0.05) detected by MaAsLin 2’s linear model. **a** Virus species associated with site, gender, CSW, HIV status, CD4+ T-cell counts and presence of lesions. **b** Box plots showing the significant associations of the relevant viruses KSHV (Human gammaherpesvirus 8), HPV6 (Alphapapillomavirus 10), HPV16 (Alphapapillomavirus 9) and HPV18 (Alphapapillomavirus 7) with the variables gender, HIV and lesions. The p-values displayed on the box plots represent the results of the Wilcoxon test, which was used to compare the dichotomous groups being depicted. **c** Bacteria species associated with site and HIV status. **d** Bacteria species associated to site, subject´s gender and CSW condition. Yellow arrowheads point to the most significant taxa shared by TGW and CSW. Heatmap color coding: The red color indicates a positive association to the reference value, referred to as the numerator of the ratio of both terms of each dichotomous variable: for site (oral/anal); gender (TGW/MSM); CSW (yes/no); HIV (positive/negative); CD4+ T-cell counts ( < 500cells/mm^3^/>500cells/mm^3^); the presence of lesions (yes/no). Blue color indicates a negative association with the reference value, or a positive association with the opposite term of the ratio (denominator).

To find associations between the microbiome and patients´ metadata, we used MaAsLin2 multivariate linear model. Results of all significant associations are provided in Supplementary Table 4. When pertinent variables such as alcohol use, tobacco use, or substance use (Table 2) were included in the model as additional adjusting factors, these variables did not show significant differences between MSM and TGW (Supplementary Table 5). Since some relevant species were detected in less than 10% of samples for virus analysis, we set the prevalence threshold at 0.05. Regarding gender, TGW showed enrichment of KSHV and *Streptoccuss phage phiNJ2* in oral samples, and *Alphapapillomavirus 7* (HPV18) and *3* (HPV72) in the anal ones when compared with MSM (Fig. 2a). For its part, MSM showed a greater abundance of the *Human betaherpesvirus 7* (HHV-7) than TGW (Fig. 2a). Just one virus taxon (*Alphapapillomavirus 8*) was positively associated with CSW (Fig. 2a). In addition, HIV-infected patients were positively associated with KSHV and negatively associated with HHV-7 in coincidence with the TGW group (Fig. 2a, b). Importantly, HIV-positive cases also showed more abundance of *Alphapapillomavirus 9* which contains the high-risk genital cancer HPV16, independently of gender condition (Fig. 2a, b). We further evaluated the HIV-related variables, presence of lesions, CD4+ T-cell counts and VL. As expected, the presence of lesions was positively associated with *Alphapapillomavirus*, which contains HPV types previously related to the development of SILs, such as the HPV16 and HPV18 (*Alphapapillomavirus 7*) and the low-risk HPV6 (*Alphapapillomavirus 10*) and HPV72 (*Alphapapillomavirus 3*) (Fig. 2b)^21,22^. Thus, since these HPV types would be related to SILs, HPV16 would also be favored by HIV-positive conditions, constituting a potential determinant of anal lesions in HIV-infected patients^23^.

**Table 2:** Sexual behavior and consumption habits of MSM and TGW: age distribution and variable frequencies in the study cohort.

	MSM	TGW	TOTAL
***Sexual behavior***			
AFSI (Mean-Median-SD)	(16.5-16-2.9)	(14.5-15-2.7)	(15.7-16-3.0)
AASI (Mean-Median-SD)	(18.6-18-3.6)	(14.6-15-2.8)	(17.0-17-3.8)
AOSI (Mean-Median-SD)	(18.1-18-3.4)	(14.7-15-3.1)	(16.7-17-3.7)
*Commercial sex worker*			
Yes	6	30	36
No	38	1	39
ND	3	0	3
*NSPLM*			
None	12	2	14
1–10	35	13	48
11–50	0	8	8
>50	0	8	8
*NSPL*			
1–10	5	1	6
11–50	20	2	22
51–100	12	1	13
>100	8	25	33
ND	2	2	4
*CUAS*			
Yes	20	21	41
No	22	9	30
ND	5	1	6
*CUOS*			
Yes	31/42	20/30	41
No	11/42	10/30	31
ND	5	1	6
***Consumption habits***			
*Alcohol*			
Yes	38/45	24/31	62
No	7/45	7/31	14
ND	2	–	2
*Tobacco*			
Yes	28/47	20/31	48
No	19/47	11/31	30
*Non-IV drug use*			
Yes	27/47	21/31	48
No	20/47	10/31	30
*IV drug use*			
Yes	1/47	0/31	1
No	46/47	31/31	77

*AFSI* age at first sexual intercourse, *AASI* age of anal sex initiation, *AOSI* age of oral sex initiation, *NPLSM* number of sexual partners in last month, *NSPL* number of sexual partners in life, *CUAS* condom use in anal sex, *CUOS* condom use in oral sex, *non-IV drug use* non-intravenous drug use, *IV drug use* intravenous drug use.

Three viral species were found positively associated with the low-CD4+ T-cell counts group: *Haemophilus phage Aaphi23*, *Streptococcus phage MM1*, and the *Human alphaherpesvirus 2* (HSV-2) (Fig. 2a). The latter is a common pathogen co-infecting more than half of HIV-positive adults and has been postulated to accelerate HIV disease^24^. Moreover, our results are in agreement with another study from Tan et al. (2013), who found an association of HSV-2 with CD4+ count <350 cells/mm3^25^. Finally, there were no significant associations between viral taxa and VL (data not shown).

Overall, the main results reveal that KSHV, HHV-7, and high-risk HPV16 and HPV18 were associated with gender and/or HIV status. While the high-risk HPV16 and HPV18 and the low-risk HPV6 and HPV72 were positively associated with the presence of lesions. Furthermore, HSV-2 correlated with a decrease in CD4+ T-cell counts.

For bacteria, we found 39 species associated with HIV status (*p* < 0.05; Pr > 0.1) (Fig. 2c). Most of them (26/39) showed a significant decrease in their relative abundances in HIV-positive compared with HIV-negative (*p* < 0.05) and belong mostly to the anal site. Notably, the species of higher abundance in HIV-positive (*p* < 0.05) are more typical of the oral mucosa (13/13) (Fig. 2c). Among those that showed enrichment in HIV-positive, there are species of *Streptococcus* (*S. parasanguinis*, *S. cristatus*, *S. anginosus* and *S. salivarius*) and *Leptotrichia* (*L. wadei* and *L. hofstadii*), together with *Prevotella salivae*, *Actynomyces odontolyticus*, *Neisseria macacae*, *Veillonella atypica* and *Campylobacter concisus* (Fig. 2c). Many of them share several characteristics, such as being common residents in the oral cavity, being colonizers in the formation of oral biofilms affecting the development of oral plaques (*S. parasanguinis, S. salivarius, V. atypica, A. odontolyticus*), or being related to the formation of caries and periodontitis processes (*V. atypica, S. parasanguinis, S. salivarius, L. wadei, L. hofstadii, C. concisus*)^26–30^. Remarkably, at least at the species level, information about these taxa and HIV status is scarce and scattered. Our results indicate that the enrichment of taxa in the oral site of HIV-positive could be associated with the pathogenesis of periodontal disease in HIV-infected patients on ART^31^.

On the other side, most taxa that showed a significant decrease in HIV-positive were associated with anal location (Fig. 2c), which would be linked to the lower diversity indices values observed in the anal samples from HIV-positive patients (Fig. 1b). Species of the gender *Coprococcus* (*C. catus, C. comes, C. euctatus*), *Ruminococcus torques*, *Blautia obeum*, *Finegoldia magna*, *Holdemanella biformis*, *Catenibacterium mitsuokai*, *Bifidobacterium adolescentis* and *Alistipes shahii*, have been previously found significantly associated to gut microbiome dysbiosis of HIV-positive individuals in different studies, consistent with our results^32–35^.

To further identify bacteria species related to HIV variables, we applied MaAsLin2 considering gender, CSW status, presence of lesions, current CD4+ T-cell counts, and current VL. We found fifteen species significantly associated with gender (*p* < 0.05; Fig. 2d; Supplementary Table 4). Of note, all were increased in TGW compared with MSM. The top relevant (*q*-value < 0.05) were *Gemella morbillorum, Granulicatella elegans, Mageeibacillus indolicus, Gemella asaccharolytica, Gardnerella vaginalis, Fusobacterium nucleatum*, and *Haemophilus parainfluenzae (*Fig. 2d*)*.

As the majority of TGW included in this study are or have been CSW (30/31) while MSM have this condition in a significantly lower proportion (6/38), we hypothesized that TGW-related taxa could be linked to sexual behavior. To support this reasoning we compared the sexual behavior variables between TGW and MSM (Table 2). The statistical analysis showed that TGW in our cohort have a history of engaging in oral and receptive anal sex since a younger age and with multiple partners compared to MSM (Supplementary Table 5), which is consistent with their activity as CSW. This suggests that this population may be at greater risk of acquiring sexually transmitted infections. Therefore, we further analyzed the taxa related to CSW condition and found fourteen species positively associated, of which seven were also associated with TGW (*p* < 0.05; Fig. 2d). These results led us to speculate that microbiome differences among gender in this cohort of participants could be partly attributable to sexual behavior independently of the HIV condition.

**Fig. 3:**
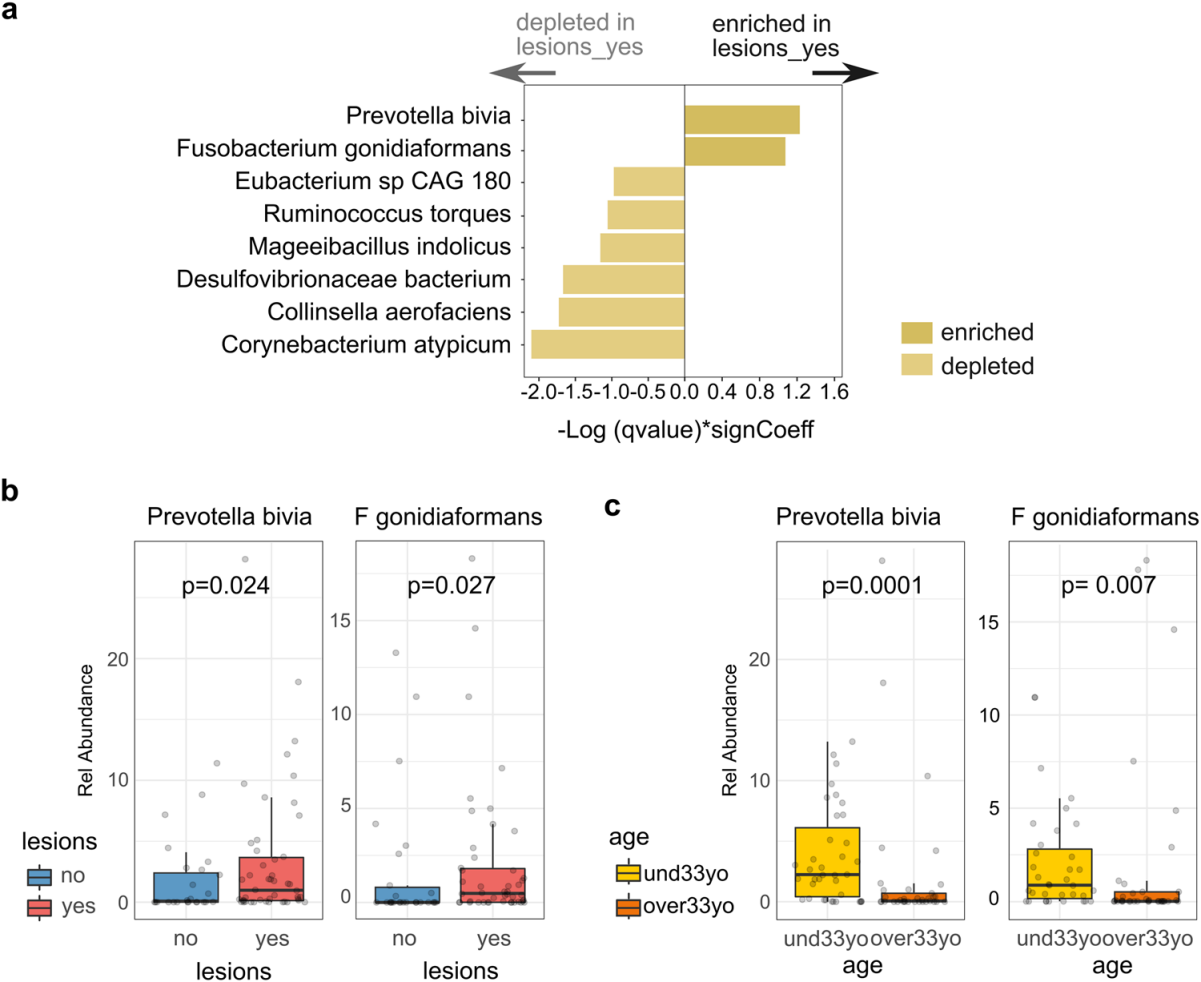
Bacteria species associated to the presence of lesions in MSM and TGW HIV related subjects. **a** Eight anal species were significantly associated with the presence of lesions (lesions_yes). **b** *P. bivia* and *F. gonidiaformans*, were the only two taxa significantly enriched in the group of subjects with lesions. **c** *P. bivia* and *F. gonidiaformans* were also negatively associated with aging. The p-values displayed on the boxp lots were calculated using the Wilcoxon test.

Conversely, the taxonomic profile of groups related to CD4+ T-cell counts, presence of lesions, and VL, showed a distinctive set of associated bacteria with almost no co-occurrence between variables. For the presence of lesions, eight anal bacterial species were significantly associated: *Prevotella bivia and Fusobacterium gonidiaformans* significantly enriched in patients with lesions; while *Mageeibacillus indolicus, Collinsella aerofaciens, Ruminococcus torques, Eubacterium sp CAG 180, Desulfovibrionaceae bacterium* and *Corynebacterium atypicum* were decreased in this group of subjects (Fig. 3a). *P. bivia and F. gonidiaformans*, in addition to being the two enriched species associated with lesions, highlight for their high abundance and prevalence among subjects (Pr > 0.25; max rel. abundance >0.2), which favors their identification and positions them as interesting markers of the presence of lesions in HIV-positive patients (Fig. 3b).

Remarkable, *P. bivia* was previously found in penile foreskin microbiome, increasing cytokine production, recruiting HIV-susceptible CD4+ T-cells, and contributing to HIV acquisition^36^. Moreover, it has recently observed to be significantly higher in samples with cervical intraepithelial neoplasia (CIN), creating a plausible environment for subsequent HPV infection, recurrence of CIN and cancer development^37^. Regarding *F. gonidiaformans*, although there is currently no link with premalignant epithelial lesions, it has been associated with other *Fusobacterium* species with higher abundance in patients with colorectal cancer tumors, meaning a potential association with epithelial malignancy^38^. We also found that *F. gonidiaformans* and *P. bivia* occurred preferentially in younger subjects (Fig. 3c), which would be in accordance with the lower diversity index observed in anal samples from older participants and the higher abundance of the phylum *Fusobacteria* in the younger group (Fig. 1c; Supplementary Fig. 2).

Furthermore, we estimated the relative risk (RR) associated with the presence of lesions in subjects with *P. bivia* and *F. gonidiaformans* in anal samples compared with individuals without detection of these taxa. Results demonstrated that those who had *P. bivia* or *F. gonidiaformans* had 2.5 (RR = 2.5; 95%CI = 1.1772−5.3172; *p* = 0.01) and 2.0 (RR = 2.0; 95%CI = 1.2292−3.2542; *p* = 0.005) higher risk have anal intraepithelial lesions, respectively, compared with those who did not have the infections (Table 3). Remarkably, when we considered those co-infected with the two species the RR increased to 9.5 (RR = 9.5; 95%CI = 1.4478–62.7231; *p* = 0.019) compared with those without none of both bacteria (Table 3).

**Table 3:** Relative risk (RR) estimation of the presence of *P. bivia* and *F. gonidiaformans* in anal samples as predisposing factors to the presence of SILs.

	Lesions		Results		
**Groups**	Yes	No	RR	95% CI	*p* value
With *P. bivia*	39	14	2.5	1.1772 −5.3172	0.017
Without *P. bivia*	5	12			
With *F. gonidiaformans*	33	9	2	1.2292 –3.2542	0.0053
Without *F. gonidiaformans*	11	17			
Coinfection with *P. bivia* and *F. gonidiaformans*	27	7	9.5	1.4478–62.7231	0.019
Without both bacteria	1	11			

Our results and previous studies provide strong evidence to propose *P. bivia* and *F. gonidiaformans* as relevant factors that increases susceptibility to anal epithelial lesions in a cohort of HIV-related MSM and TGW.

**Fig. 4:**
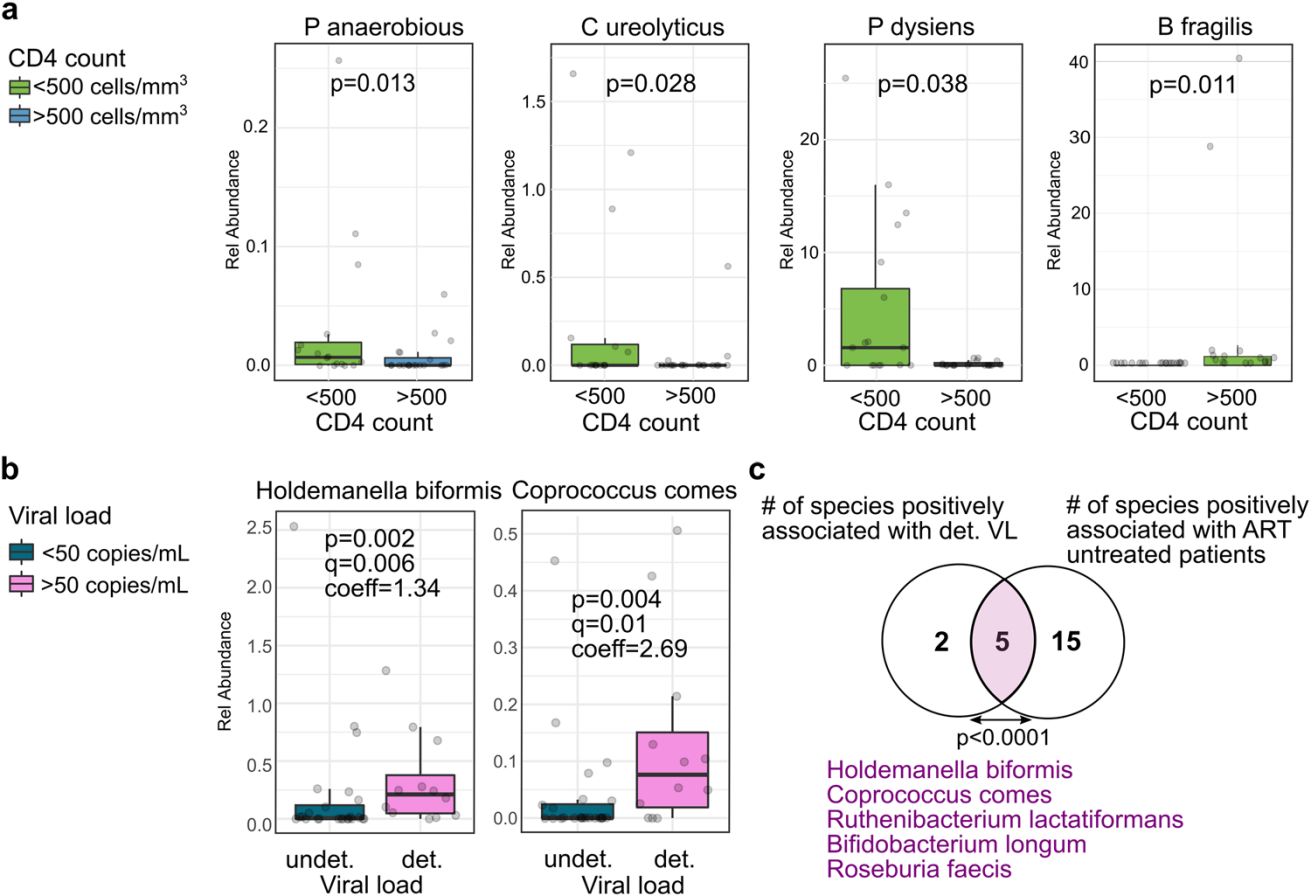
Relevant bacterial species associated to CD4+ T-cell counts and Viral Load groups. **a** Box plots showing the significant relationship of four anal bacterial species with CD4+ T-cell counts. The *p*-values displayed on the box plots were calculated using the Wilcoxon test. **b** Relative abundance of *H. biformis* and *C. comes* as the most relevant bacterial species associated with detectable VL in HIV-positive patients. The *p*-values displayed on the boxp lots were obtained from the multivariate analysis. **c** Venn diagram showing common species positively associated with detectable VL and ART untreated variable groups. det. = detectable, undet. = undetectable. To determine whether the observed intersection in the Venn diagram is statistically significant Fisher’s exact test was used.

CD4+ T-cell counts and HIV viral load are two biomarkers routinely collected from HIV-positive patients to monitor infection and treatment response. We treated the CD4+ T-cell variable as categorical to define taxa associated with the CD4+ T-cell counts group (<500 cells/mm^3^/>500 cells/mm^3^). Eight species were identified, six of them increased in the lower CD4+ T-cell counts group (*Peptostreptococcus anaerobius*, *Bacteroides fragilis*, *Campylobacter ureolyticus, Desulfovibrionaceae bacterium*, and *Prevotella disiens)* and one (*Anaerostipes hadrus)* enriched in the higher CD4+ T-cell counts group (Supplementary Table 4). Figure 4a shows four relevant enriched species in the lower CD4+ T-cell count group. *P. anaerobius* has been shown to modulate the immune microenvironment by recruiting immune-suppressive cells that could directly suppress CD4+ and CD8+ T cell activities^39^. In addition, *P. anaerobius* and *P. disiens* were shown to recruit HIV-susceptible CD4+ T cells to the inner foreskin, and were associated with HIV acquisition^36^. *C. ureolyticus* has been identified as an emerging gastrointestinal pathogen causative of inflammation, gastroenteritis and diarrhea in patients with HIV infection^40,41^. *B fragilis* has been shown to exert potent immunomodulatory activity to protect against a lethal viral neuroinflammatory disease^42^. Our results define a distinctive group of anal bacteria that would have immunomodulatory effects in HIV-positive patients.

For the VL group our analysis identified seven species positively associated with detectable HIV (*Holdemanella biformis*, *Coprococcus comes*, *Ruthenibacterium lactatiformans*, *Desulfovibrionaceae bacterium*, *Bifidobacterium longum*, *Gemmiger formicilis* and *Roseburia faecis*) and three negatively associated (*Neisseria flavescens*, *Anaerostipes hadrus* and *Actinomyces turicensis*) (Supplementary Table 4). *C. comes* (*p* = 0.004; Pr > 0.3) and *H. biformis* (*p* = 0.002; Pr > 0.3) were the most prevalent and positively associated with the anal samples (Fig. 4b), while *N. flavescens* (*p* < 0.003; Pr > 0.4) was the most prevalent and negatively associated with the oral samples (Supplementary Table 4).

*C. comes* and *H. biformis* were among the depleted bacteria in HIV-positive compared with HIV-negative (Fig. 2c), indicating that HIV-positive patients stratify into two groups regarding the enrichment of *C. comes* and *H. biformis* in detectable VL. To investigate whether this difference could be ascribed to the administration of ART treatment, we compared the abundance of bacteria in treated and untreated patients. By utilizing Venn diagrams, we found that the prevalence of *C. comes*, *H. biformis*, and three other species (*B. longum*, *R. faecis*, and *R. lactatiformis*) were consistently associated with both detectable viral load and ART-untreated patients (Fig. 4c). In this regard, it was reported that *H. biformis* is enriched in MSM where most individuals were not on treatment. In addition, *H. biformis* was enriched in untreated HIV-positive MSM or on a diversity of different ART drugs^43^. Similarly, *C. comes* was found to be enriched in a group of HIV-infected patients categorized as immunological ART non-responders compared to the group of patients immunologically responsive to treatment^44^. Together, our results and similar findings from other studies suggest that both *H. biformis* and *C. comes* could be relevant markers of immune response to ART treatment in HIV-infected patients.

### Microbial pathways and functional genes

**Fig. 5:**
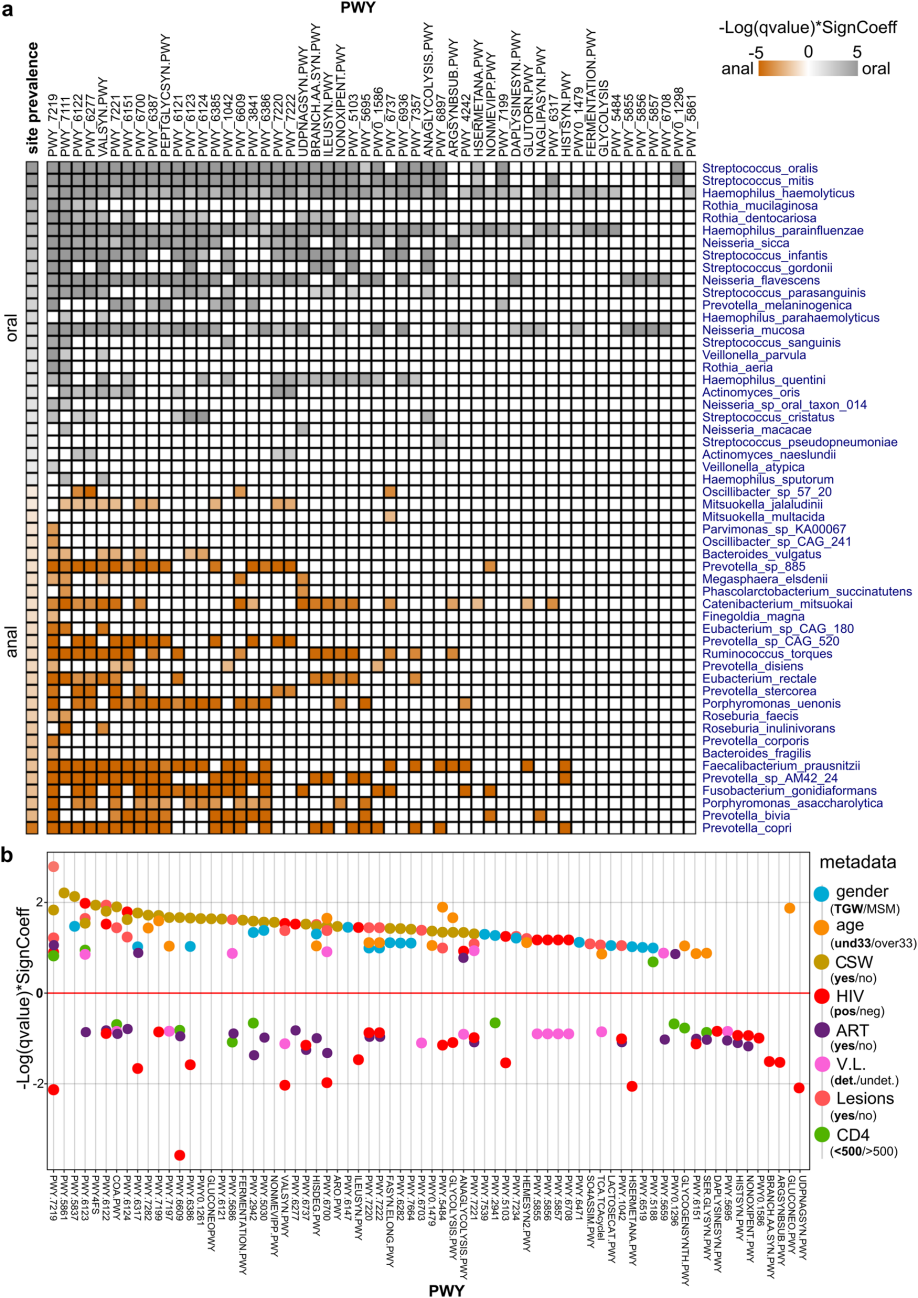
Pathway profile in a multivariable association between phenotypes and microbial species. **a** Heatmap representation of the fifty-two PWYs associated with oral and anal sites contributed by fifty-four species. **b** Scatter plot showing the relationship of the top significant differentially abundant PWYs associated with the different groups of variables, represented by their reference values (highlighted in bold): gender, TGW; age, und33yo; CSW, yes; HIV, positive; ART, yes; VL, detectable (det.); Lesions, yes; CD4+ T-cell counts (<500cells/mm^3^/>500cells/mm^3^). The full name of PWYs is available in Supplementary Table 6.

The relative abundance of pathways (PWYs) concerning the different groups was analyzed by MaAsLin2. We identified fifty-two pathways associated with oral and anal sites contributed by fifty-four species, of which twenty-six were prevalent in the oral site and twenty-eight in the anal site (Fig. 5a; Supplementary Table 6). The most overrepresented pathways (>50% species) were adenosine ribonucleotides de novo biosynthesis (PWY-7219), pyruvate fermentation to isobutanol (PWY-7111), 5-aminoimidazole ribonucleotide biosynthesis II (PWY-6122; PWY-6277), L.valinebiosynthesis (VALSYN.PWY) and guanosine ribonucleotides de novo biosynthesis (PWY-7221). The top five contributing species were *H haemolyticus*, *H parainfluenzae*, *S mitis*, *S oralis*, and *N mucosa* for the oral site, and *F prausnitzii*, *P copri*, *P sp AM42 24*, *C mitsuokai*, and *F gonidiaformans* for the anal site (Fig. 5a).

The multivariate analysis showed an enrichment of numerous pathways in TGW, CSW, younger subjects (under33yo), and subjects with lesions (Supplementary Table 7). In contrast, depletion of several distinctive pathways was observed for the groups on ART, HIV-positive and low CD4+ T-cell counts (Fig. 5b). In this sense, our results reveal two related but different settings: on one side the enrichment of several metabolic PWYs associated with younger subjects, TGW, Commercial Sex Workers, and subjects with precancerous anal lesions; on the other a predominant decrease in various pathways associated with people with HIV, detectable VL, ART treatment, and low CD4 cell counts, variables more related to the HIV condition (Fig. 5b). These pathways involved the biosynthesis of the basic amino acids arginine, histidine, and lysine and the hydrophobic one’s valine, methionine and isoleucine. We also found a depletion of oxidative and energetic processes such as pyruvate metabolism, glycolysis, and lipid biosynthesis (Supplementary Table 7). The few enriched pathways identified in HIV-infected cases were related to the ubiquinol biosynthesis (PWY-5855, PWY-5856, PWY-5857, PWY-6708); remarkably, they were found depleted in the group of detectable VL (Fig. 5b).

The taxa´s contribution to pathways differences were attributable to a few species associated with the defined conditions (Supplementary Table 7). These results would be consistent with the hypothesis that HIV-associated dysbiosis is independent of sexual practice or sexual preferences^45,46^.

**Fig. 6:**
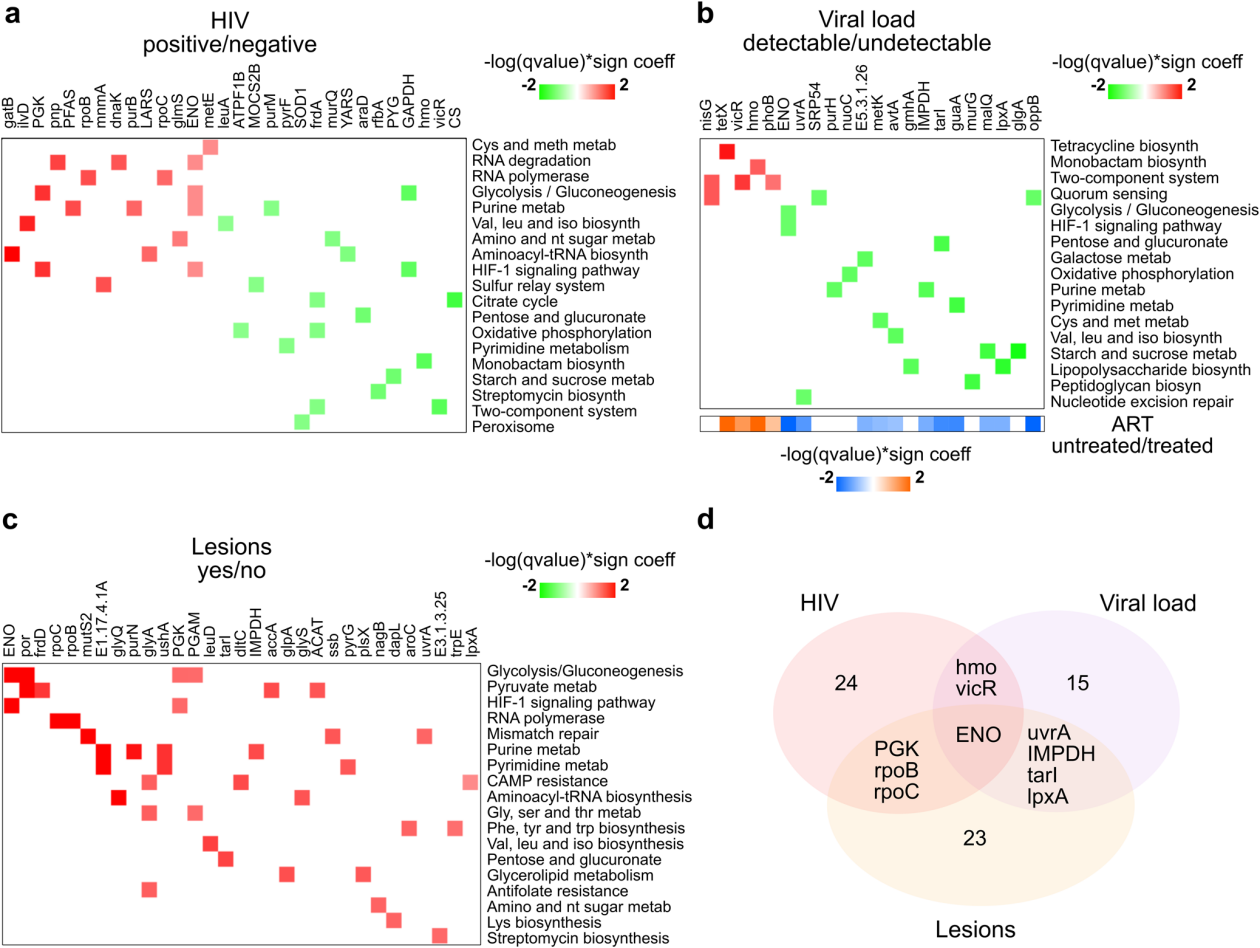
Differentially abundant bacterial genes between the groups of the variables: HIV, viral load and presence of lesions, and their associated KEGG pathways names. **a** HIV-positive vs. HIV-negative differentially abundant genes. Red color indicates a positive association with the reference value (HIV-positive); green color indicates a negative association with the reference value. **b** VL detectable vs. VL undetectable; reference value = VL detectable. In the bottom, it is indicated which genes are also differentially abundant among ART treated vs. ART untreated. Orange color indicates a positive association with the reference value (ART untreated) Blue color indicates a negative association with the reference value. **c** Presence of lesions_yes vs presence of lesions_no. Red color indicates a positive association with the reference value (lesions_yes); green color indicates a negative association to the reference value. **d** Venn diagram showing common genes among signatures of genes.

Furthermore, to assess the potential functionality of oral and anal microbiota in a multivariable analysis based on bacterial genomes, microbial gene families obtained from MaAsLin2 (*p* < 0.05; Pr > 0.2; Supplementary Table 8) were annotated and analyzed using different databases and functional systems: UniProt, KEGGREST, KEGGMapper, MinPath. We were able to define gene signatures related to HIV infection, the presence of lesions and detectable viral load (Fig. 6).

HIV-positive was mainly enriched by genes related to RNA metabolism (pnp. DNAk, ENO, rpoB, rpoC), glycolysis/glyconeogenesis (ENO, PGK), amino acid metabolism (gatB, ilvD, PGK, pnp, PFAS, mnmA, etc.) and HIF1-signaling (ENO, PGK) (Fig. 6a). Of these genes, the so-called moonlighting genes (which have more than one biological function), DNAk and ENO, stand out. DnaK is a plasminogen-binding protein that upregulates APOB3G expression in CD4+ T cells and, more importantly, competes with HIV for binding to CCR5, and can block HIV binding^47,48^. ENO (enolase) is a prototypic moonlighting protein, in both prokaryotes and eukaryotes, with putative roles in a variety of human diseases^49^. Remarkably, human enolase ENO1 has recently been shown to suppress HIV-1 integration in viral target cells^50^. Although more studies are needed, bacterial DnaK and ENO could have some therapeutic potential in HIV-positive subjects.

Among HIV-positive with detectable VL, we identified a group of enriched genes (Tetx, nisG, VicR, hmo, phoB) associated with antimicrobial resistance and sensing of stressful conditions (Quorum sensing, two-component system) (Fig. 6b). Tetx monooxygenase showed the highest significant difference (p = 0.009). This enzyme catalyzes efficient degradation of a broad range of tetracycline analogs and conferred resistance to these antibiotics in vivo^51^. Recently, it was shown that the ART drug zidovudine decreases Tetx-mediated bacterial resistance^52^. Consistent with this, we found that Tetx along with genes enriched in the detectable VL group (VicR, hmo, phoB), were also enriched in the group of patients who did not receive treatment compared with the ART-treated group, and vice versa (Fig. 6b), suggesting these genes could be relevant markers of ART treatment response.

On the other hand, genes identified in the group of subjects with lesions were all enriched in this group (Fig. 6c). These genes are involved in several processes related to amino acids metabolism (glyA, leuD, glyQ, trpE, aroC, etc.) carbohydrates metabolism (ENO, por, PGK, PGAM) and nucleotide metabolism (purN, ushA, pyrG, etc.), among others (Fig. 6c). Finally, we found the ENO gene common to all three signatures reinforcing the idea of the potential relevancy of enolase as a gene marker to be investigated in the context of HIV and related diseases (Fig. 6d).

## Discussion

### Anal and oral Virome of HIV-infected patients and related factors

Virome of anal samples was mainly represented by species of Alphapapillomavirus, being Alphapapillomavirus 10 (HPV6, HPV11), 9 (HPV16), 3 (HPV72), and 7 (HPV18) among the most prevalent. It is established that HIV infection is associated with a greater HPV capacity to persist and increase the risk and progression of precancerous anal intraepithelial lesions. We found a significant enrichment of the oncogenic HPV16 in HIV-infected subjects with anal intraepithelial lesions, reinforcing the fact these patients are at an increased risk of anal cancer^53^. Furthermore, lesions also correlated with enrichment of HPV18, HPV6, and HPV72, independently of HIV condition. In addition, HPV18 and HPV72 were more prevalent in TGW compared with MSM. Overall, the results indicate that HPV16 would be the main driver in the progression of anal lesions in HIV-infected patients. TGW showed a higher susceptibility to HPV infection.

Human alphaherpesvirus 2 (HSV-2) was detected in anal samples associated with decreased CD4+ T-cell counts independently of HIV infection. It has been postulated that HSV-2 and HIV coinfection accelerate HIV disease, characterized by a persistent decline of CD4+ T cells^24,25,54^. Whether HSV-2 and HIV synergistically contribute to CD4+ T cell depletion, our results suggest that HSV-2 might be a marker of immunocompromised individuals in the context of HIV.

The oral virome of our cohort showed enrichment in bacteriophages, KSHV (HHV-8) and HHV-7, among others, being KSHV significantly predominant in HIV-positive and TGW independently of sexual behavior, compared with HIV-negative and MSM, respectively. Since KHSV is highly oncogenic in HIV-infected patients, the TGW of our cohort would be at higher risk of Kaposi sarcoma development. These results also suggest that non-sexual routes may explain the transmission of KSHV in the TGW cohort^55^.

### HIV-associated microbiome and related factors

Previous studies have demonstrated that HIV infection and related factors exert profound changes in the gut microbiome. Most agree on a decrease in the richness and diversity indices with consequences in inflammatory processes and immune activation^34,56–58^. Consistent with those findings, our results showed lower indices in the anal samples of older subjects, HIV-positive patients, and the group of patients with lesions. Aging, HIV, and lesions are known to affect immunological status with an impact on the gut microbiome^9,32,59,60^. In particular, aging and HIV share features of intestinal damage and alterations in the communities of gut bacteria conducting to dysbiosis^60^. Less is known about diversity variations and the presence of anal SILs. However, several studies have suggested that microbiome variations contribute to cervical intraepithelial neoplasia (CIN) development^9,61^.

HIV infection significantly correlated with a decrease in species of *Lachnospiraceae*, *Ruminococcaceae*, *Erysipelotrichaceae*, *Porphyromonadaceae* or *Rikenellaceae*. In previous studies, taxa of these families have been found mostly depleted in HIV infection^62–66^.

Among the species found decreased, *C. comes* (*Lachnospiraceae*) and *H. biformis* (*Erysipelotrichaceae*) were associated with VL and ART treatment. It has been shown that *C. comes* has immunostimulatory properties in the acute phase response, which is the first immune response signal to HIV-1 infection with the increase of acute-phase reactants^67,68^. Additionally, *C. comes* was found to negatively correlate with the production of IL-22, a cytokine that downregulates CCR5 expression through induction of acute phase proteins^68,69^. These studies suggest *C. comes* plays a relevant role in the initial phase of HIV infection, modulating acute phase response with pro- or anti-inflammatory factors to manipulate the immune system.

Similarly, *H. biformis* was found to be enriched in untreated MSM patients and associated with an increased abundance of CCR5+CD4+ T cells, increasing the risk of HIV transmission^32,43^.

Our analysis identified that both *C. comes* and *H. biformis* were significantly enriched in untreated patients with detectable VL compared to individuals on ART and undetectable VL. Together, our results and those found in other studies indicate that *C. comes* and *H. biformis* could potentially accelerate HIV progression while the patient is not receiving treatment, placing them as potential markers of the immune response to ART treatment in HIV-infected patients.

While the microbiome has been well documented in the gut mucosa, less is known about the oral mucosa in the HIV context, particularly in TGW^31,70^. Another important finding that emerged from our analysis is the enrichment of bacterial species associated with periodontal pathological processes in the oral mucosa of HIV-positive. We identified common residents of the oral cavity such as *S. salivarius*, *S. parasanguinis*, and *S. cristatus*, as well as an enrichment of anaerobic bacteria such as *A. odontolyticus*, *L. hofstadii*, *L. wadei*, *C. concisus*, *P. salivae*, and *V. atypica*. Although many of these taxa have been previously characterized in the context of periodontal processes, little is known about their association with HIV. In this regard, compared with negative controls, *S. anginosus* and *A. odontolyticus* were found enriched in the gut of HIV-positive subjects on ART^19^. *S. cristatus* has been linked to the inhibition of HIV replication through the induction of APOBEC3 expression, while elevated levels of *C. concisus* were associated with detectable plasmatic VL in HIV-positive individuals^71,72^.

Interestingly, we also identified a signature of oral species associated with gender and/or sexual behavior independently of HIV condition. These species differed from those found associated with HIV infection, and were all enriched in TGW compared with MSM, and many were also prevalent in the group of CSW. Similarly, three species were found significantly enriched in the anal samples of both TGW and CSW groups. Importantly, many species enriched in TGW have been related to female genitalia and/or sexual transmission*. G. asaccharolytica* has been described as a vaginal bacteria associated with an increased risk of HIV infection in unprotected sex^73^. *M. indolicus* is a recently described species originally isolated from the female genital tract^74^. *H. parainfluenzae* has been postulated to be a sexually transmitted genitourinary pathogen among MSM^75^. *G. vaginalis* is mainly detected in women with bacterial vaginosis; conversely, it is uncommon in men or at sites other than the female genitalia; however, people with more sex partners and multiple sex partners are more at risk for getting infections of *G. vaginalis*^76^.

The fact that TGW of our cohort are most CSW led to the inescapable assumption that microbiome differences observed in this group are linked to sexual behavior. However, it is important to consider that TGW are often exposed to feminizing hormone therapy, which can impact microbiome composition^77^. In this regard, the higher abundance of bacteria usually found in the female genital tract, in the anal samples of TGW, compared with MSM, allows us to justify further studies to investigate this approach.

Furthermore, among the most enriched bacteria in the oral mucosa of TGW was *F. nucleatum*, a periodontal pathogen that can promote cancer by several mechanisms. It has been related to oral squamous cell carcinoma and colorectal cancer^78^. This finding, along with the higher prevalence of the oncogenic viruses KSHV and HPV-18 in TGW compared with MSM, suggest that this population is not only at increased risk of HIV infection but also of oncogenic infections.

### Microbiome changes associated with HPV-related precancerous anal lesions

A recent study revealed that mucosal bacteria could predict the presence of anal lesions in HIV-infected MSM^79^. Here, we provided important evidence of the association of bacterial taxa at species level and the presence of anal lesions in both MSM and TGW subjects.

Two anal species were significantly enriched: *P. bivia* and *F. gonidianformans*. Remarkably, *P. bivia* has been related to high-risk HPV persistent infection in vaginal microbiome^80^. Moreover, in a later study *P. bivia* was found significantly higher in samples of pre-menopausal, non-pregnant women with CIN before and after excision treatment compared with control samples with normal cytology^37^. Furthermore, we estimated the RR of *P. bivia* and found that subjects with the infection had 2.5 times more likely to have anal intraepithelial lesions, compared with those who did not have detectable *P. bivia*. In addition, the RR increased to 9.5 in subjects with both *P. bivia* and *F. gonidaformans*, the latter related to colorectal malignancies. Therefore, we consider that *P. bivia* and *F. gonidiaformans* would be two relevant bacterial infections associated with the development of precancerous anal lesions.

In this study, we also defined a distinctive group of anal bacteria associated with low CD4+ T-cell counts (*P. anaerobious*, *C. ureolyticus*, *P. dissiens and B. fragilis*) that would have immunomodulatory effects in HIV-positive patients^37,39–42^.

### Functional pathways and metabolic genes in HIV and related factors

We next analyzed the functional consequences of HIV infection and related factors. Pathways analysis revealed a significant decrease of several pathways in HIV-positive patients, mainly contributed by anal bacteria. These pathways involve, the biosynthesis of amino acids, pyruvate metabolism, glycolysis and lipid biosynthesis among others. It has been reported that an impaired metabolic capacity of the HIV gut microbiota to produce amino acids, which would be in line with our results^81^. Furthermore, the depletion of energetic bioprocesses would be related to the nutritional deficits observed in HIV disease^82^.

Interestingly, of the few enriched pathways in HIV-positive, the synthesis of ubiquinol (coenzyme Q7-10) stands out since these pathways were found to be depleted in the group of patients with detectable VL. Bacterial coenzyme Q contributes to the overall anti-oxidative stress system, antibiotics resistance, and modulation of bacterial virulence^83^. Diverse studies have suggested that HIV infection is characterized by oxidative stress contributing to several aspects of HIV disease pathogenesis, including viral replication, inflammatory response, loss of immune function, and chronic weight loss^84^. In addition, HIV may contribute to a decreased ability of the antioxidant system to control oxidative stress and increase HIV replication^85^. Thus, it is possible that the enrichment of bacteria that synthesize coenzyme Q benefits HIV patients, contributing to a relief of oxidative stress caused by the virus.

Pathways multivariable analysis revealed a significant enrichment of several metabolic processes in younger subjects, TGW, CSW, and subjects with precancerous anal lesions, all groups characterized by subjects with high frequency of sexual intercourse. While a predominant decrease of others bioprocess was associated with aging, HIV infection, detectable VL, ART treatment, and low CD4+ T-cell counts, variables more related to HIV pathogenesis. These results indicate the differential impact caused on the metabolic pathways of the microbiota by different but related factors. In addition, it would be in line with the fact that sexual practice has been revealed as a major source of microbiota variation, confounding prior interpretations of gut microbiota alterations among subjects with HIV^45,46^. Such studies have suggested that the differences between the gut microbiota of HIV-positive and negative subjects can be attributed in part to sexual preference, thus increasing the possibility that both scenarios, despite being closely related, contribute to a distinctive microbial microenvironment.

We finally described microbial gene richness associations with HIV infection, viral load, and the presence of lesions. For HIV-positive, we identified gene signatures related to oxidative and energy metabolism. Although some of these genes belong to bacterial proteins that are still poorly characterized, others deserve special attention since they have been previously associated with HIV as being involved in mechanisms of response to the infection (DNAk, ENO) or to the ART treatment (tetX)^47–51^ In addition, it is worth mentioning vicR and phoB, genes associated with response to nutritional stress, which were identified along with Tetx significantly more abundant in patients with detectable viral load. Together, these genes may constitute relevant markers of the presence of bacteria that respond to the stressful conditions generated by HIV disease.

Similarly, we defined a signature of genes enriched in the group of patients with lesions, many of them contributed by *F. gonidiaformans* and *P. bivia*, thus constituting potential prognostic biomarkers of developing anal intraepithelial lesions.

Overall, this study provides a comprehensive characterization of the oral and anal microbiome in a cohort of high-risk HIV-exposed MSM and TGW subjects from Argentina. Shotgun metagenomics sequencing allowed us to define viral and bacterial taxa associated with the subject´s gender, sex behavior, HIV infection, and related variables such as CD4+ T-cell counts, HIV viral load, and the development of precancerous anal lesions. Our results confirm the occurrence of oncogenic viromes in this high HIV-risk population. We also showed that TGW in our cohort may be at a greater risk of acquiring sexually transmitted infections explained in part by the sexual behavior of this group. However, we acknowledge that engagement in sex work and the influence of different sexual behaviors on the microbiome is complex, and care should be taken in interpreting the data. As we did not include a group of TGW who do not participate in sex work, we cannot generalize our findings to all TGW. We propose *C. comes* and *H. biformis* as potential markers of the immune response to ART treatment in HIV-infected patients. Moreover, we identified *P. bivia* and *F. gonidiaformans* as two relevant components that predispose to anal intraepithelial lesions. We showed that metabolic pathways are distinctively affected according to whether the predominant factors are associated with sexual behavior or HIV pathogenesis. Gene family analysis identified bacterial gene signatures associated with HIV status, viral load, ART, and the presence of lesions, which may have potential as prognostic and predictive biomarkers of HIV/AIDS-associated malignancies. Furthermore, our results confirm previous results from other studies and provide new insights that need to be explored in future studies.

## Methods

### Study participants

This study included an Argentinian cohort of HIV-positive and HIV-negative cases of TGW and MSM recruited at Fundación Huésped, Buenos Aires, Argentina. Participants were age > 18 at the beginning of the study (median: 33 years; range: 19–58 years).

### Ethics approval and consent to participate

All participants included in this study signed informed consent before being involved in the project. The study was approved by the institutional review board (Comité de Bioética, Fundación Huésped).

### Samples collection and DNA purification

A total of 130 samples (59 oral swabs and 71 anal swabs) were obtained from 47 MSM and 31 TGW.

Samples were collected in Qiagen specimen collection device (Qiagen, USA) by qualified staff at Fundación Huésped. DNA purification was performed with QIAMP DNA kits (Qiagen, USA). DNA integrity and concentration was evaluated and measured on Agarose (0.7%) gels electrophoresis stained with GelGreen® and Nanodrop spectrophotometer, respectively.

### Data collection and variables

The study collected data from participants on clinical variables, sexual practices and consumption habits, which are displayed in Tables 1 and 2, respectively. Clinical data included gender, age, HIV status, current CD4+ T-cell counts, current viral load (VL), use of antiretroviral therapy (ART), and anal cytology results indicating the presence of epithelial lesions, specifically Atypical Squamous Cells of Undetermined Significance (ASCUS), Low-grade Squamous Intraepithelial Lesion (LSIL), and High-Grade Squamous Intraepithelial Lesion (HSIL). Sexual behavior variables comprised Age at First Sexual Intercourse (AFSI), Age of Anal Sex Initiation (AASI), Age of Oral Sex Initiation (AOFSI), Number of Sexual Partners in the Last Month (NSPLM), Number of Sexual Partners in Life (NSPL), Condom Use in Anal (CUAS) and Oral Sex (CUOS), and whether participants are or have been Commercial Sex Workers (CSW). It is important to note that none of the TGW included in the study have undergone gender-affirming surgery. Consumption habits included Alcohol, Tobacco, and intravenous or non-intravenous drug use. All metadata of samples and participants is available in Supplementary Table 1.

### Shotgun metagenomics sequencing and data pre-processing

DNA samples were subject to cleanup and then processed for shotgun metagenomics sequencing using the PCR-free KAPA HyperPLUS (Roche, France) library preparation kit at the Onco-Genomics Shared Resource (OGSR, University of Miami, USA). We performed 100 nt paired-end sequencing using an Illumina NovaSeq 6000 System and obtained about 80 million reads per sample.

Raw data (510 GiB) was downloaded from Illumina BaseSpace to our dedicated server using BaseMount tools, and processed with the bioBakery toolkits^86^. Quality control on metagenomics sequencing data (Fastq files), were performed with KneadData tools, which separate the human (host) and the non-human (microbiome) reads for further QC-based filtering. The filtered reads from KneadData were then aligned and mapped to all reference microbiome genome sequences to determine the presence and abundance of different taxonomic groups among 130 DNA samples derived from oral and anal swabs. Host-removed metagenome sequences have been submitted to the SRA database with accession number PRJNA983219.

### Taxonomic profiling, richness, and diversity

For profiling the composition of microbial communities at the species level, we run MetaPhlAn 3.0 using default parameters. MetaPhlAn relies on unique clade-specific marker genes identified from ~17,000 reference genomes (~13,500 bacterial and archaeal, ~3500 viral, and ~110 eukaryotic)^86^. This bioinformatics tool provides the relative abundances of each microbial clade with species-level resolution.

Species richness and diversity were calculated using R function *estimate_richness* from R package “phyloseq”. We considered the observed species and Chao1 indices for richness, and the Shannon and Simpson indices for diversity. Beta diversity was measured by Bray–Curtis, weighted UniFrac, and unweighted UniFrac. For Principal Coordinate Analysis, the Aitchison distance was used as the distance metric to analyze the compositional data^87^. To test whether the samples cluster beyond that expected by sampling variability we applied permutational multivariate analysis of variance (PERMANOVA). Differences in richness and diversity indices between the two groups were determined using the Wilcoxon rank sum test with a significance level of 0.05. For relative abundance analysis and visualization, we used R phyloseq and HMP packages^87,88^.

### Differential abundance analysis

For determining the relative differential abundance and the multivariable association between subjects’ metadata and microbial features, we used the MaAsLin2 package from the bioBakery suite in R/Bioconductor^89^. We used default parameters for normalization (TSS method), transformation (Log), analysis method (LM), correction method (BH), and significance threshold (*q*-value < 0.25). Although association in MaAsLin 2.0 is considered significant at a *q*-value of below 0.25, a cut-off used in previous microbiome studies, for more stringency we selected the associated features (species, pathways, gene families) with a *q*-value < 0.15 (nominal *p*-value < 0.05)^86,89–91^. The minimum abundance for each feature was set to 0.001 (0.1%) while the minimum percent of samples for which a feature was detected (prevalence, Pr) at minimum abundance was used as follows: 0.05 (5%) for viruses, 0.1 (10%) for bacteria and pathways and 0.2 (20%) for gene families. To identify features that differ between sample categories, variables were defined and dichotomized as follows: site (oral/anal); gender (TGW/MSM); age (based on the median, under33yo/over33yo); CSW (yes/no); HIV status (positive/negative); current CD4+ T-cell counts (<500cel/mm^3^/>500cel/mm^3^); current VL (detectable: >50copies/ml, undetectable:<50copies/ml); for anal cytology, we grouped ASCUS (*n* = 3), LSILs (*n* = 40) and HSILs (*n* = 8) into the variable name presence of lesions (yes/no). For coefficients, referred to as the contrast between the category specified versus the reference category, we considered as reference category the first term or “numerator” of the dichotomous annotation^89^. Variables of consumption habits were included in the multivariate model to account for their potential impact on the microbiome as confounding factors. However, they were not the primary focus of this study and were only considered as covariates to adjust for additional factors.

### Pathways and gene family analysis

Whole genome metagenomics pathway analysis was adopted in the HMP Unified Metabolic Analysis Network 3 (HUMAnN3) pipeline to assess the potential differences in metabolic pathways. HUMAnN3 identifies the species profile from metagenomics shotgun sequencing data, aligns reads to their pan-genomes, performs translated search on unclassified reads, and quantifies gene families and pathways^86^. By default, gene families are annotated using the comprehensive protein database UniRef90^92^ and metabolic pathways are annotated using MetaCyc database^93^. The UniRef90 gene family abundance from HUMAnN3 was then regrouped to Kyoto Encyclopedia of Genes and Genomes (KEGG) orthology (KO)^94^. We used the KEGGREST package in R/Biconductor for KO identifiers^95^ and KEGG Mapper reconstruct tool for KEGG pathway maps and MinPath to biological pathway reconstruction/inference^96,97^.

### Statistical analysis and data visualization

Heatmap representation of taxa abundances was done with the R package “phyloseq”. For the unsupervised ordination of samples, we applied the NMDS method and Bray distance in the plot_heatmap function. Heatmap visualization of differentially represented taxa and pathways was done with R/Bioconductor and the MultiExperiment Viewer software (MeV v4.9). For the different statistical comparisons outside of MaAsLin’s analysis, we used R/Bioconductor. In order to analyze continuous variables, we utilized either *t* tests or Wilcoxon tests as appropriate. For categorical data, we employed Chi-squared and Fisher tests.

### Reporting summary

Further information on research design is available in the Nature Research Reporting Summary linked to this article.

## Supplementary information

Supplementary Tables and Figures

Reporting Summary

## Data Availability

Host-removed metagenomics sequences are available on the NCBI SRA database with the following link: https://www.ncbi.nlm.nih.gov/sra/PRJNA983219. All data generated or analyzed during this study are included in this published article [and its supplementary information files].

## Code availability

All open-source codes used to analyze and visualize the data of this study are available in bioBakery and R/Bioconductor websites: GitHub - biobakery/biobakery: bioBakery tools for meta’omic profiling bioconductor.org/packages/release/bioc/vignettes/phyloseq/inst/doc/phyloseq-analysis.R; bioconductor.org/packages/release/bioc/vignettes/Maaslin2/inst/doc/maaslin2.R.
