# Supplementary material for: Oral and anal microbiome from HIV-exposed individuals: role of host-associated factors in taxa composition and metabolic pathways
Source: NPJ Biofilms Microbiomes. 2023 Jul 12;9:48. doi: 10.1038/s41522-023-00413-4 (PMC10338440; doi:10.1038/s41522-023-00413-4)
Supplement: Supplementary file 1 — Supplementary Tables and Figures [file 41522_2023_413_MOESM1_ESM.pdf]

Supplementary information

Supplemental figures and tables

Index:

Supplementary Table 1. Samples and patients' metadata.

Supplementary Figure 1. Beta diversity of samples from oral and anal sites.

Supplementary Table 2. Wilcoxon Rank Sum Test p-values for the comparison of alpha-diversity indexes among variables

Supplementary Table 3. Taxonomic and phylogenetic composition of prevalent taxa at species level identified in the study.

Supplementary Figure 2. Microbial relative abundance comparisons at Phylum level.

Supplementary Table 4. Significant associations among taxa and subjects´ metadata.

Supplementary Table 5. Statistics of variables related to sexual behavior and consumption habits of MSM and TGW.

Supplementary Table 6. Significant associations of pathways and sample sites.

Supplementary Table 7. Significant associations among pathways and subjects´ metadata.

Supplementary Table 8. Significant associations between gene families and HIV, viral load, and the presence of lesions.

Supplementary Table 1. Samples and patients' metadata.

subject	sample	site	gender	HIV	age	SW	lesions	CD4+ T cell count	Viral_Load	ART	AFSI	AASI	AOSI	NPLSM	NSPL	CUAS	CUOS	IV drug	non-IV drug	Tobacco	Alcohol
FH27-0008	FH27-0008-A	anal	MSM	Pos	41.2	No	no	under500	detectable	yes	17	22	17	1-10	>500	0	1	0	1	1	1
FH27-0010	FH27-0010-A	anal	MSM	Pos	42.5	Yes	no	over500	undetectable	yes	12	14	22	1-10	101-500	1	1	NA	1	1	1
FH27-0012	FH27-0012-O	oral	MSM	Neg	33	NA	yes	NA	NA	NA	19	20	20	1-10	11-50	0	1	0	1	0	1
FH27-0013	FH27-0013-O	oral	MSM	Pos	58.3	No	yes	over500	undetectable	yes	18	25	25	1-10	>500	1	1	0	0	1	1
FH27-0016	FH27-0016-A	anal	TGW	Neg	26.5	Yes	yes	NA	NA	NA	17	17	17	101-500	NA	1	1	0	1	1	1
FH27-0016	FH27-0016-O	oral	TGW	Neg	26.5	Yes	yes	NA	NA	NA	17	17	17	101-500	NA	1	1	0	1	1	1
FH27-0017	FH27-0017-O	oral	TGW	Pos	33.2	Yes	yes	under500	undetectable	yes	13	13	14	101-500	101-500	0	1	0	1	1	0
FH27-0019	FH27-0019-A	anal	TGW	Neg	28.5	Yes	yes	NA	NA	NA	16	16	16	101-500	101-500	0	0	0	0	1	1
FH27-0022	FH27-0022-A	anal	MSM	Pos	50.9	No	yes	over500	undetectable	yes	16	21	16	0	>500	1	NA	0	0	0	0
FH27-0022	FH27-0022-O	oral	MSM	Pos	50.9	No	yes	over500	undetectable	yes	16	21	16	0	>500	1	NA	0	0	0	0
FH27-0023	FH27-0023-A	anal	MSM	Pos	40.1	No	yes	under500	undetectable	yes	20	25	20	1-10	51-100	1	1	0	1	1	1
FH27-0023	FH27-0023-O	oral	MSM	Pos	40.1	No	yes	under500	undetectable	yes	20	25	20	1-10	51-100	1	1	0	1	1	1
FH27-0024	FH27-0024-O	oral	MSM	Pos	34.9	NA	yes	over500	undetectable	yes	17	17	17	1-10	1-10	1	1	0	1	0	1
FH27-0026	FH27-0026-O	oral	MSM	Neg	30.3	No	yes	NA	NA	NA	19	19	19	1-10	11-50	NA	NA	0	0	0	1
FH27-0028	FH27-0028-A	anal	MSM	Pos	30.2	No	yes	over500	undetectable	yes	16	16	17	1-10	11-50	0	1	0	0	0	1
FH27-0028	FH27-0028-O	oral	MSM	Pos	30.2	No	yes	over500	undetectable	yes	16	16	17	1-10	11-50	0	1	0	0	0	1
FH27-0031	FH27-0031-A	anal	MSM	Pos	20.6	No	yes	over500	detectable	no	16	16	16	1-10	11-50	NA	NA	0	1	1	1
FH27-0031	FH27-0031-O	oral	MSM	Pos	20.6	No	yes	over500	detectable	no	16	16	16	1-10	11-50	NA	NA	0	1	1	1
FH27-0032	FH27-0032-A	anal	MSM	Pos	37.3	Yes	yes	over500	undetectable	yes	19	20	22	1-10	51-100	0	0	0	1	0	1
FH27-0032	FH27-0032-O	oral	MSM	Pos	37.3	Yes	yes	over500	undetectable	yes	19	20	22	1-10	51-100	0	0	0	1	0	1
FH27-0034	FH27-0034-A	anal	MSM	Pos	50.7	No	yes	over500	undetectable	yes	16	18	16	1-10	51-100	0	1	0	0	1	1
FH27-0034	FH27-0034-O	oral	MSM	Pos	50.7	No	yes	over500	undetectable	yes	16	18	16	1-10	51-100	0	1	0	0	1	1
FH27-0035	FH27-0035-A	anal	MSM	Pos	37.3	No	yes	over500	detectable	yes	22	22	22	1-10	11-50	0	1	0	1	1	1
FH27-0035	FH27-0035-O	oral	MSM	Pos	37.3	No	yes	over500	detectable	yes	22	22	22	1-10	11-50	0	1	0	1	1	1
FH27-0036	FH27-0036-A	anal	TGW	Pos	36.3	Yes	yes	over500	detectable	yes	18	19	18	11-50	101-500	1	1	0	1	1	1
FH27-0036	FH27-0036-O	oral	TGW	Pos	36.3	Yes	yes	over500	detectable	yes	18	19	18	11-50	101-500	1	1	0	1	1	1
FH27-0037	FH27-0037-A	anal	MSM	Pos	37.3	No	yes	over500	undetectable	yes	9	9	11	1-10	51-100	0	1	0	1	1	1
FH27-0037	FH27-0037-O	oral	MSM	Pos	37.3	No	yes	over500	undetectable	yes	9	9	11	1-10	51-100	0	1	0	1	1	1
FH27-0038	FH27-0038-A	anal	TGW	Pos	41.7	Yes	yes	NA	undetectable	yes	14	14	14	1-10	101-500	0	0	0	0	1	0
FH27-0038	FH27-0038-O	oral	TGW	Pos	41.7	Yes	yes	NA	undetectable	yes	14	14	14	1-10	101-500	0	0	0	0	1	0
FH27-0039	FH27-0039-A	anal	MSM	Pos	47.4	No	no	NA	undetectable	yes	15	20	15	1-10	51-100	NA	NA	0	1	0	0
FH27-0039	FH27-0039-O	oral	MSM	Pos	47.4	No	no	NA	undetectable	yes	15	20	15	1-10	51-100	NA	NA	0	1	0	0
FH27-0041	FH27-0041-A	anal	TGW	Pos	49.2	Yes	yes	over500	undetectable	yes	21	21	21	101-500	101-500	0	1	0	1	0	1
FH27-0041	FH27-0041-O	oral	TGW	Pos	49.2	Yes	yes	over500	undetectable	yes	21	21	21	101-500	101-500	0	1	0	1	0	1
FH27-0042	FH27-0042-A	anal	MSM	Pos	40.9	No	yes	under500	undetectable	yes	24	24	24	1-10	NA	1	1	0	0	0	1
FH27-0042	FH27-0042-O	oral	MSM	Pos	40.9	No	yes	under500	undetectable	yes	24	24	24	1-10	NA	1	1	0	0	0	1
FH27-0043	FH27-0043-A	anal	TGW	Neg	21	Yes	no	NA	NA	NA	12	12	12	101-500	101-500	1	1	0	1	1	1
FH27-0043	FH27-0043-O	oral	TGW	Neg	21	Yes	no	NA	NA	NA	12	12	12	101-500	101-500	1	1	0	1	1	1
FH27-0044	FH27-0044-A	anal	MSM	Pos	33	No	no	over500	undetectable	yes	13	16	13	1-10	51-100	1	1	0	1	1	1
FH27-0044	FH27-0044-O	oral	MSM	Pos	33	No	no	over500	undetectable	yes	13	16	13	1-10	51-100	1	1	0	1	1	1
FH27-0045	FH27-0045-A	anal	MSM	Neg	23.3	Yes	yes	NA	NA	NA	19	19	19	1-10	11-50	1	0	0	0	1	1
FH27-0045	FH27-0045-O	oral	MSM	Neg	23.3	Yes	yes	NA	NA	NA	19	19	19	1-10	11-50	1	0	0	0	1	1
FH27-0046	FH27-0046-A	anal	MSM	Pos	42.5	NA	yes	over500	undetectable	yes	14	14	17	1-10	11-50	0	1	0	0	0	0
FH27-0046	FH27-0046-O	oral	MSM	Pos	42.5	NA	yes	over500	undetectable	yes	14	14	17	1-10	11-50	0	1	0	0	0	0
FH27-0047	FH27-0047-A	anal	MSM	Pos	47.6	No	yes	under500	undetectable	yes	17	26	28	1-10	11-50	0	0	0	0	0	0
FH27-0047	FH27-0047-O	oral	MSM	Pos	47.6	No	yes	under500	undetectable	yes	17	26	28	1-10	11-50	0	0	0	0	0	0
FH27-0049	FH27-0049-A	anal	MSM	Neg	30.4	No	no	NA	NA	NA	14	23	23	1-10	11-50	1	1	0	1	1	1
FH27-0051	FH27-0051-A	anal	MSM	Pos	25.9	No	yes	over500	undetectable	yes	5	15	15	0	11-50	NA	1	0	0	0	0
FH27-0051	FH27-0051-O	oral	MSM	Pos	25.9	No	yes	over500	undetectable	yes	5	15	15	0	11-50	NA	1	0	0	0	0
FH27-0053	FH27-0053-A	anal	MSM	Pos	29.8	No	yes	over500	undetectable	yes	15	15	15	1-10	NA	1	1	NA	1	1	1
FH27-0053	FH27-0053-O	oral	MSM	Pos	29.8	No	yes	over500	undetectable	yes	15	15	15	1-10	NA	1	1	NA	1	1	1
FH27-0054	FH27-0054-A	anal	MSM	Pos	38.6	Yes	yes	over500	undetectable	yes	18	23	20	0	101-500	0	1	0	1	0	1
FH27-0054	FH27-0054-O	oral	MSM	Pos	38.6	Yes	yes	over500	undetectable	yes	18	23	20	0	101-500	0	1	0	1	0	1
FH27-0057	FH27-0057-A	oral	MSM	Neg	29.9	No	no	NA	NA	NA	19	20	19	1-10	101-500	0	1	0	0	0	1
FH27-0058	FH27-0058-A	anal	MSM	Neg	26.2	No	yes	NA	NA	NA	14	17	18	1-10	1-10	NA	NA	0	0	0	1
FH27-0058	FH27-0058-O	oral	MSM	Neg	26.2	No	yes	NA	NA	NA	14	17	18	1-10	1-10	NA	NA	0	0	0	1
FH27-0059	FH27-0059-A	anal	MSM	Pos	31.2	No	yes	over500	undetectable	yes	19	19	19	1-10	101-500	0	1	0	1	1	1
FH27-0059	FH27-0059-O	oral	MSM	Pos	31.2	No	yes	over500	undetectable	yes	19	19	19	1-10	101-500	0	1	0	1	1	1
FH27-0060	FH27-0060-A	anal	MSM	Pos	42.6	Yes	yes	ND	NA	no	17	17	17	0	51-100	0	1	0	1	1	1
FH27-0060	FH27-0060-O	oral	MSM	Pos	42.6	Yes	yes	ND	NA	no	17	17	17	0	51-100	0	1	0	1	1	1
FH27-0061	FH27-0061-A	anal	MSM	Pos	25	No	yes	under500	detectable	no	19	19	19	1-10	11-50	0	1	0	0	1	1
FH27-0061	FH27-0061-O	oral	MSM	Pos	25	No	yes	under500	detectable	no	19	19	19	1-10	11-50	0	1	0	0	1	1
FH27-0063	FH27-0063-A	anal	MSM	Pos	46.5	No	yes	under500	NA	yes	13	18	15	0	11-50	0	1	0	0	1	1
FH27-0063	FH27-0063-O	oral	MSM	Pos	46.5	No	yes	under500	NA	yes	13	18	15	0	11-50	0	1	0	0	1	1
FH27-0064	FH27-0064-A	anal	MSM	Pos	29.3	No	yes	over500	NA	yes	25	25	25	0	1-10	1	1	0	0	1	1
FH27-0064	FH27-0064-O	oral	MSM	Pos	29.3	No	yes	over500	NA	yes	25	25	25	0	1-10	1	1	0	0	1	1
FH27-0065	FH27-0065-A	anal	MSM	Pos	25.2	No	yes	under500	detectable	no	18	18	18	0	11-50	1	1	0	0	0	0
FH27-0065	FH27-0065-O	oral	MSM	Pos	25.2	No	yes	under500	detectable	no	18	18	18	0	11-50	1	1	0	0	0	0
FH27-0066	FH27-0066-A	anal	MSM	Pos	46.3	Yes	no	under500	detectable	yes	13	17	16	1-10	51-100	1	1	1	1	1	1
FH27-0066	FH27-0066-O	oral	MSM	Pos	46.3	Yes	no	under500	detectable	yes	13	17	16	1-10	51-100	1	1	1	1	1	1
FH27-0067	FH27-0067-A	anal	MSM	Pos	32.9	No	no	under500	detectable	no	15	17	15	0	11-50	0	0	0	1	1	1
FH27-0067	FH27-0067-O	oral	MSM	Pos	32.9	No	no	under500	detectable	no	15	17	15	0	11-50	0	0	0	1	1	1
FH27-0068	FH27-0068-A	anal	MSM	Pos	35.6	No	yes	under500	undetectable	yes	14	19	19	0	11-50	0	1	0	1	1	1
FH27-0068	FH27-0068-O	oral	MSM	Pos	35.6	No	yes	under500	undetectable	yes	14	19	19	0	11-50	0	1	0	1	1	1
FH27-0071	FH27-0071-A	anal	TGW	Neg	29.8	Yes	yes	NA	NA	NA	17	17	17	11-50	101-500	0	1	0	0	0	0
FH27-0071	FH27-0071-O	oral	TGW	Neg	29.8	Yes	yes	NA	NA	NA	17	17	17	11-50	101-500	0	1	0	0	0	0
FH27-0072	FH27-0072-A	anal	TGW	Pos	51	Yes	yes	under500	undetectable	yes	19	19	19	1-10	>500	NA	NA	0	0	0	0
FH27-0072	FH27-0072-O	oral	TGW	Pos	51	Yes	yes	under500	undetectable	yes	19	19	19	1-10	>500	NA	NA	0	0	0	0
FH27-0074	FH27-0074-A	anal	MSM	Neg	24.9	No	no	NA	NA	NA	14	14	14	1-10	51-100	0	1	0	1	0	NA
FH27-0074	FH27-0074-O	oral	MSM	Neg	24.9	No	no	NA	NA	NA	14	14	14	1-10	51-100	0	1	0	1	0	NA
FH27-0090	FH27-0090-A	anal	MSM	Neg	21.7	No	yes	NA	NA	NA	14	14	14	1-10	11-50	1	0	0	0	0	1
FH27-0090	FH27-0090-O	oral	MSM	Neg	21.7	No	yes	NA	NA	NA	14	14	14	1-10	11-50	1	0	0	0	0	1
FH27-0094	FH27-0094-A	anal	MSM	Pos	45.4	No	no	over500	NA	yes	13	13	13	0	51-100	0	0	0	1	1	0
FH27-0094	FH27-0094-O	oral	MSM	Pos	45.4	No	no	over500	NA	yes	13	13	13	0	51-100	0	0	0	1	1	0
FH27-0097	FH27-0097-A	anal	TGW	Neg	26.9	Yes	no	NA	NA	NA	13	13	13	101-500	NA	1	0	0	1	1	1
FH27-0097	FH27-0097-O	oral	TGW	Neg	26.9	Yes	no	NA	NA	NA	13	13	13	101-500	NA	1	0	0	1	1	1
FH27-0098	FH27-0098-A	anal	MSM	Neg	24	No	yes	NA	NA	NA	17	17	17	1-10	11-50	1	1	0	1	1	1
FH30-0103	FH30-0103-A	anal	TGW	Neg	29	Yes	yes	NA	NA	NA	9	9	9	101-500	>500	1	1	0	1	0	1
FH30-0104	FH30-0104-A	anal	MSM	Pos	42.7	No	no	over500	undetectable	yes	17	17	17	1-10	101-500	0	0	0	1	1	1
FH30-0104	FH30-0104-O	oral	MSM	Pos	42.7	No	no	over500	undetectable	yes	17	17	17	1-10	101-500	0	0	0	1	1	1
FH30-0110	FH30-0110-A	anal	TGW	Pos	28.2	Yes	yes	under500	detectable	no	14	14	9	0	>500	0	0	0	1	0	0
FH30-0110	FH30-0110-O	oral	TGW	Pos	28.2	Yes	yes	under500	detectable	no	14	14	9	0	>500	0	0	0	1	0	0
FH30-0113	FH30-0113-A	anal	TGW	Neg	40	Yes	no	NA	NA	NA	16	16	16	11-50	>500	1	1	0	1	1	1
FH30-0113	FH30-0113-O	oral	TGW	Neg	40	Yes	no	NA	NA	NA	16	16	16	11-50	>500	1	1	0	1	1	1
FH30-0119	FH30-0119-A	anal	TGW	Pos	28.8	Yes	no	NA	undetectable	yes	9	9	9	1-10	11-50	1	1	0	1	0	0
FH30-0120	FH30-0120-A	anal	MSM	Neg	19.4	No	yes	NA	NA	NA	15	16	16	1-10	51-100	1	0	0	1	1	1
FH30-0120	FH30-0120-O	oral	MSM	Neg	19.4	No	yes	NA	NA	NA	15	16	16	1-10	51-100	1	0	0	1	1	1

Supplementary Table 1.(continuation)

subject	sample	site	gender	HIV	age	SW	ence of le	CD4+ T cell count	Viral_Load	ART	AFSI	AASI	AOSI	NPLSM	NSPL	CUAS	CUOS	IV drug	non-IV drug	Tobacco	Alcohol
FH30-0122	FH30-0122-A	anal	MSM	Neg	24.8	No	yes	NA	NA	NA	18	18	17	0	11-50	1	1	0	1	1	1
FH30-0122	FH30-0122-O	oral	MSM	Neg	24.8	No	yes	NA	NA	NA	18	18	17	0	11-50	1	1	0	1	1	1
FH30-0123	FH30-0123-A	anal	MSM	Pos	25.1	No	yes	under500	undetectable	yes	19	20	20	1-10	51-100	1	0	0	0	0	1
FH30-0123	FH30-0123-O	oral	MSM	Pos	25.1	No	yes	under500	undetectable	yes	19	20	20	1-10	51-100	1	0	0	0	0	1
FH30-0125	FH30-0125-A	anal	MSM	Pos	21.4	No	yes	under500	undetectable	yes	17	18	18	1-10	1-10	1	1	0	1	0	NA
FH30-0125	FH30-0125-O	oral	MSM	Pos	21.4	No	yes	under500	undetectable	yes	17	18	18	1-10	1-10	1	1	0	1	0	NA
FH30-0126	FH30-0126-A	anal	TGW	Pos	31.4	Yes	yes	under500	detectable	yes	15	17	15	1-10	101-500	1	0	0	0	0	1
FH30-0128	FH30-0128-A	anal	TGW	Pos	38	Yes	no	NA	NA	yes	13	13	13	11-50	>500	1	1	0	1	1	1
FH30-0133	FH30-0133-A	anal	MSM	Neg	41.4	No	no	NA	NA	NA	16	22	20	1-10	51-100	0	0	0	0	1	0
FH30-0140	FH30-0140-O	oral	TGW	Neg	23	Yes	yes	NA	NA	NA	11	11	15	1-10	101-500	1	1	0	0	1	1
FH30-0144	FH30-0144-A	anal	TGW	Pos	30.3	No	yes	NA	NA	yes	15	15	21	1-10	101-500	1	0	0	1	1	1
FH30-0168	FH30-0168-A	anal	MSM	Neg	27.1	No	no	NA	NA	NA	15	18	18	1-10	11-50	1	1	0	0	1	1
FH30-0168	FH30-0168-O	oral	MSM	Neg	27.1	No	no	NA	NA	NA	15	18	18	1-10	11-50	1	1	0	0	1	1
FH30-0170	FH30-0170-A	anal	TGW	Neg	40.7	Yes	no	NA	NA	NA	15	15	15	11-50	>500	1	1	0	1	1	1
FH30-0170	FH30-0170-O	oral	TGW	Neg	40.7	Yes	no	NA	NA	NA	15	15	15	11-50	>500	1	1	0	1	1	1
FH30-0172	FH30-0172-A	anal	TGW	Pos	49.8	Yes	no	over500	undetectable	yes	17	17	17	0	101-500	1	1	0	1	0	1
FH30-0173	FH30-0173-O	oral	TGW	Neg	30.7	Yes	yes	NA	NA	NA	13	13	13	1-10	>500	0	0	0	1	0	1
FH30-0176	FH30-0176-A	anal	TGW	Neg	32.3	Yes	no	NA	NA	NA	14	14	14	1-10	>500	1	1	0	0	1	1
FH30-0177	FH30-0177-A	anal	TGW	Pos	35.1	Yes	yes	under500	detectable	yes	13	13	13	1-10	>500	1	1	0	1	1	1
FH30-0177	FH30-0177-O	oral	TGW	Pos	35.1	Yes	yes	under500	detectable	yes	13	13	13	1-10	>500	1	1	0	1	1	1
FH30-0182	FH30-0182-A	anal	TGW	Neg	46.8	Yes	no	NA	NA	NA	14	14	14	11-50	>500	1	1	0	1	1	1
FH30-0184	FH30-0184-A	anal	TGW	Neg	24.1	Yes	no	NA	NA	NA	17	18	18	1-10	11-50	1	1	0	0	1	1
FH30-0185	FH30-0185-A	anal	TGW	Neg	33.3	Yes	no	NA	NA	NA	16	16	16	1-10	1-10	0	0	0	0	0	1
FH30-0186	FH30-0186-A	anal	MSM	Pos	43.6	No	yes	over500	undetectable	yes	16	23	20	0	1-10	0	0	0	1	1	1
FH30-0189	FH30-0189-A	anal	TGW	Pos	31.9	Yes	no	over500	detectable	yes	10	10	10	11-50	101-500	1	0	0	1	1	1
FH30-0189	FH30-0189-O	oral	TGW	Pos	31.9	Yes	no	over500	detectable	yes	10	10	10	11-50	101-500	1	0	0	1	1	1
FH30-0191	FH30-0191-A	anal	TGW	Pos	36.4	Yes	no	NA	NA	yes	15	15	15	11-50	>500	1	1	0	0	1	1
FH30-0191	FH30-0191-O	oral	TGW	Pos	36.4	Yes	no	NA	NA	yes	15	15	15	11-50	>500	1	1	0	0	1	1
FH30-0193	FH30-0193-A	anal	TGW	Pos	22.1	Yes	yes	over500	NA	yes	13	13	11	51-100	>500	1	0	0	1	1	1
FH30-0204	FH30-0204-A	anal	TGW	Neg	44.5	Yes	no	NA	NA	NA	16	16	16	1-10	>500	1	1	0	1	1	1
FH30-0204	FH30-0204-O	oral	TGW	Neg	44.5	Yes	no	NA	NA	NA	16	16	16	1-10	>500	1	1	0	1	1	1
FH30-0205	FH30-0205-A	anal	TGW	Pos	35.7	Yes	yes	over500	undetectable	yes	15	15	15	1-10	51-100	0	1	0	1	0	0
FH30-0205	FH30-0205-O	oral	TGW	Pos	35.7	Yes	yes	over500	undetectable	yes	15	15	15	1-10	51-100	0	1	0	1	0	0

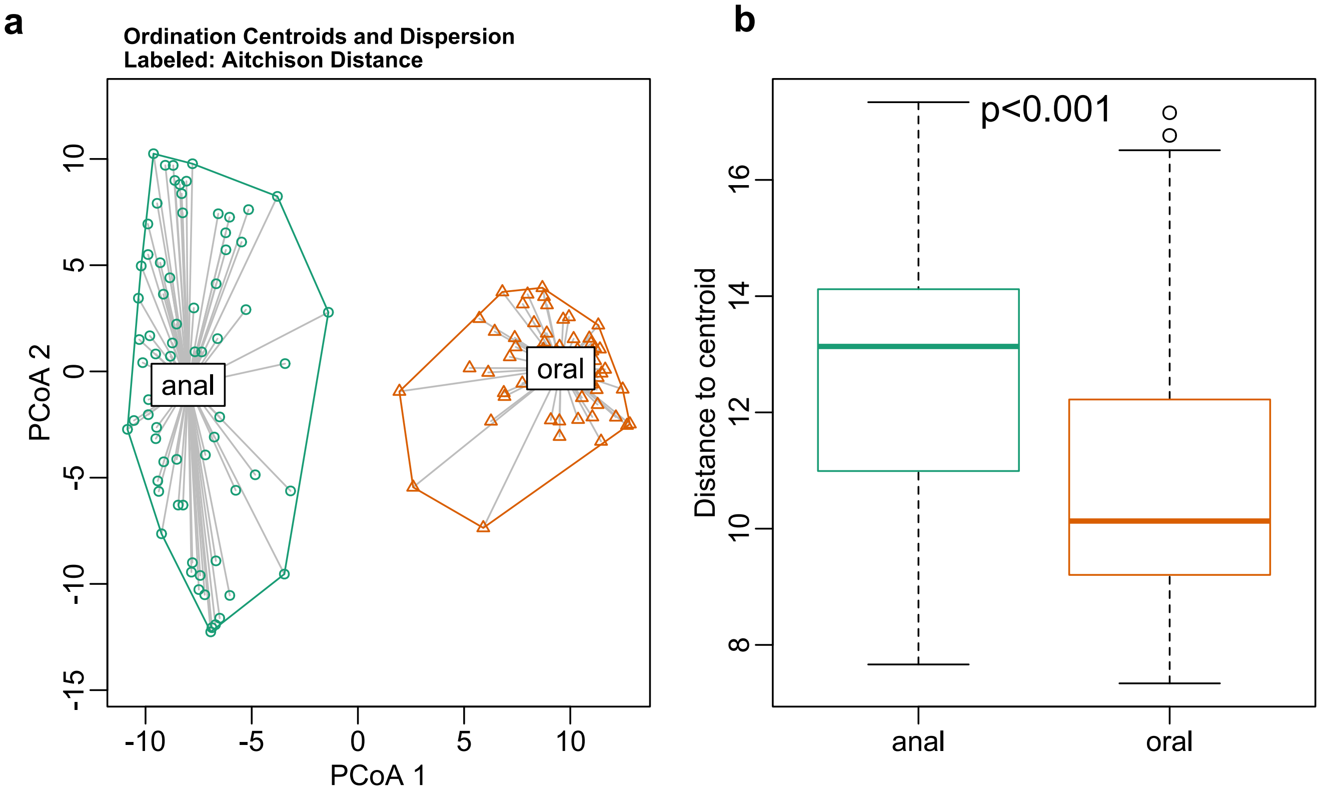

Supplementary Figure 1: Beta diversity of oral and anal site samples. a. Principal coordinates analysis (PCoA) plot based on the Aitchison distance beta diversity metric with centroids depicted with the name of sample origin site for the 130 samples included in the study. **b.** Boxplot representation of the distances to centroid for the oral and anal sites obtained PCoA. Community differences were verified by PERMANOVA, $p < 0.001$.

Supplementary Table 2: Wilcoxon Rank Sum Test p-values for the comparison of alpha-diversity indexes among variables

Site	Variable	Observed	Chao1	Shannon	Simpson
Anal	Gender	0.195	0.180	0.434	0.668
	Age	**0.017**	0.211	**0.046**	**0.044**
	HIV	0.184	0.508	**0.027**	**0.014**
	Lesions	0.066	0.066	**0.014**	**0.003**
	SW	0.1601	0.09084	0.4198	0.5792
	CD4+ T cell counts	0.4269	0.8143	0.3222	0.2943
	ART	0.6035	0.5418	0.5215	0.3942
Oral	Gender	0.180	0.296	0.074	0.185
	Age	0.665	0.183	0.642	0.355
	HIV	0.785	0.871	0.728	0.562
	Lesions	0.539	0.542	0.942	0.852
	SW	0.22	0.2856	0.1245	0.1887
	CD4+ T cell counts	0.8539	1	0.9638	0.844
	ART	0.6658	0.9713	0.7927	0.8748

Supplementary Table 3. Taxonomic and phylogenetic composition of prevalent taxa at species level identified in the study.

Kingdom	Phylum	Class	Order	Family	Gender	Specie	metadata	value	coef	N	prevalence	pval	qval
Bacteria	Bacteroidetes	Bacteroidia	Bacteroidales	Prevotellaceae	Prevotella	Prevotella_copri	site	oral	-9.359521557	130	0.461538462	2.71E-25	9.40E-24
Bacteria	Firmicutes	Tissierellia	Tissierellales	Peptoniphilaceae	Finegoldia	Finegoldia_magna	site	oral	-8.858814565	130	0.438461538	5.76E-18	1.12E-16
Bacteria	Bacteroidetes	Bacteroidia	Bacteroidales	Prevotellaceae	Prevotella	Prevotella_bivia	site	oral	-7.439633322	130	0.438461538	1.04E-17	1.70E-16
Bacteria	Bacteroidetes	Bacteroidia	Bacteroidales	Prevotellaceae	Prevotella	Prevotella_disiens	site	oral	-6.785246816	130	0.353846154	4.47E-13	3.67E-12
Bacteria	Firmicutes	Clostridia	Clostridiales	Lachnospiraceae	Coprococcus	Coprococcus_comes	site	oral	-6.377228451	130	0.353846154	1.20E-16	1.50E-15
Bacteria	Bacteroidetes	Bacteroidia	Bacteroidales	Prevotellaceae	Prevotella	Prevotella_sp_CAG_520	site	oral	-6.299248807	130	0.346153846	1.99E-13	1.77E-12
Bacteria	Firmicutes	Negativicutes	Acidaminococcales	Acidaminococcaceae	Phascolarctobacterium	Phascolarctobacterium_succinatutens	site	oral	-6.200598684	130	0.3	5.21E-11	2.76E-10
Bacteria	Bacteroidetes	Bacteroidia	Bacteroidales	Prevotellaceae	Prevotella	Prevotella_sp_AM42_24	site	oral	-5.978080838	130	0.323076923	1.44E-11	9.15E-11
Bacteria	Firmicutes	Clostridia	Clostridiales	Lachnospiraceae	Lachnospiraceae_unclas	Eubacterium_rectale	site	oral	-5.754821103	130	0.361538462	4.18E-17	5.92E-16
Bacteria	Firmicutes	Clostridia	Clostridiales	Lachnospiraceae	Blautia	Blautia_wexlerae	site	oral	-4.918430132	130	0.323076923	1.84E-13	1.69E-12
Bacteria	Firmicutes	Clostridia	Clostridiales	Lachnospiraceae	Roseburia	Roseburia_inulinivorans	site	oral	-4.872812562	130	0.330769231	5.08E-14	4.95E-13
Bacteria	Firmicutes	Tissierellia	Tissierellales	Peptoniphilaceae	Parvimonas	Parvimonas_sp_KA00067	site	oral	-4.763305483	130	0.323076923	1.34E-13	1.27E-12
Bacteria	Bacteroidetes	Bacteroidia	Bacteroidales	Prevotellaceae	Prevotella	Prevotella_sp_885	site	oral	-4.76217321	130	0.346153846	4.10E-12	2.78E-11
Bacteria	Firmicutes	Clostridia	Clostridiales	Ruminococcaceae	Faecalibacterium	Faecalibacterium_prausnitzii	site	oral	-4.745637715	130	0.438461538	8.04E-22	1.93E-20
Bacteria	Bacteroidetes	Bacteroidia	Bacteroidales	Bacteroidaceae	Bacteroides	Bacteroides_vulgatus	site	oral	-4.681914172	130	0.284615385	2.61E-11	1.45E-10
Bacteria	Firmicutes	Clostridia	Clostridiales	Lachnospiraceae	Dorea	Dorea_formicigenerans	site	oral	-4.436956696	130	0.361538462	1.77E-15	1.90E-14
Bacteria	Firmicutes	Clostridia	Clostridiales	Oscillospiraceae	Oscillibacter	Oscillibacter_sp_57_20	site	oral	-4.389559474	130	0.338461538	6.77E-15	7.04E-14
Bacteria	Firmicutes	Clostridia	Clostridiales	Lachnospiraceae	Fusicatenibacter	Fusicatenibacter_saccharivorans	site	oral	-4.361636173	130	0.3	1.27E-12	9.43E-12
Bacteria	Actinobacteria	Coriobacteriia	Coriobacteriales	Coriobacteriaceae	Collinsella	Collinsella_aerofaciens	site	oral	-4.169990982	130	0.446153846	2.80E-20	6.24E-19
Bacteria	Bacteroidetes	Bacteroidia	Bacteroidales	Prevotellaceae	Prevotella	Prevotella_buccalis	site	oral	-4.075716031	130	0.284615385	8.42E-09	3.46E-08
Bacteria	Firmicutes	Clostridia	Clostridiales	Peptostreptococcaceae	Peptostreptococcus	Peptostreptococcus_anaerobius	site	oral	-4.069673416	130	0.369230769	2.45E-12	1.78E-11
Bacteria	Bacteroidetes	Bacteroidia	Bacteroidales	Prevotellaceae	Prevotella	Prevotella_stercorea	site	oral	-4.047497651	130	0.346153846	4.46E-13	3.67E-12
Viruses	Viruses_unclassified	Viruses_unclassified	Viruses_unclassified	Papillomaviridae	Alphapapillomavirus	Alphapapillomavirus_10	site	oral	-3.892786365	130	0.346153846	1.22E-09	5.58E-09
Bacteria	Actinobacteria	Actinobacteria	Actinomycetales	Actinomycetaceae	Actinomyces	Actinomyces_turicensis	site	oral	-3.739244867	130	0.269230769	5.54E-10	2.70E-09
Bacteria	Firmicutes	Clostridia	Clostridiales	Lachnospiraceae	Dorea	Dorea_longicatena	site	oral	-3.656827035	130	0.369230769	3.74E-16	4.49E-15
Bacteria	Firmicutes	Clostridia	Clostridiales	Lachnospiraceae	Roseburia	Roseburia_faecis	site	oral	-3.616269314	130	0.269230769	6.09E-10	2.92E-09
Bacteria	Fusobacteria	Fusobacteriia	Fusobacteriales	Fusobacteriaceae	Fusobacterium	Fusobacterium_gonidiaformans	site	oral	-3.589154249	130	0.338461538	2.82E-12	1.96E-11
Bacteria	Proteobacteria	Gammaproteobacteria	Enterobacterales	Enterobacteriaceae	Escherichia	Escherichia_coli	site	oral	-3.461374366	130	0.3	2.37E-11	1.38E-10
Bacteria	Firmicutes	Clostridia	Clostridiales	Oscillospiraceae	Oscillibacter	Oscillibacter_sp_CAG_241	site	oral	-3.459079852	130	0.284615385	1.26E-10	6.33E-10
Bacteria	Bacteroidetes	Bacteroidia	Bacteroidales	Prevotellaceae	Prevotella	Prevotella_corporis	site	oral	-3.427917133	130	0.230769231	9.04E-08	3.36E-07
Bacteria	Bacteroidetes	Bacteroidia	Bacteroidales	Prevotellaceae	Prevotella	Prevotella_sp_CAG_279	site	oral	-3.349248904	130	0.3	1.58E-11	9.65E-11
Bacteria	Firmicutes	Negativicutes	Veillonellales	Veillonellaceae	Megasphaera	Megasphaera_elsdenii	site	oral	-3.334856512	130	0.269230769	2.15E-09	9.33E-09
Bacteria	Firmicutes	Clostridia	Clostridiales	Lachnospiraceae	Blautia	Ruminococcus_torques	site	oral	-3.287496981	130	0.346153846	1.15E-11	7.61E-11
Bacteria	Actinobacteria	Actinobacteria	Bifidobacteriales	Bifidobacteriaceae	Bifidobacterium	Bifidobacterium_adolescentis	site	oral	-3.238973164	130	0.207692308	1.23E-07	4.27E-07
Bacteria	Bacteroidetes	Bacteroidia	Bacteroidales	Prevotellaceae	Prevotella	Prevotella_timonensis	site	oral	-3.114349038	130	0.323076923	3.65E-11	2.00E-10
Bacteria	Firmicutes	Erysipelotrichia	Erysipelotrichales	Erysipelotrichaceae	Catenibacterium	Catenibacterium_mitsuokai	site	oral	-3.10212881	130	0.369230769	8.57E-15	8.62E-14
Bacteria	Bacteroidetes	Bacteroidia	Bacteroidales	Prevotellaceae	Prevotella	Prevotella_sp_CAG_5226	site	oral	-3.100216905	130	0.161538462	9.80E-06	2.28E-05
Bacteria	Firmicutes	Tissierellia	Tissierellales	Peptoniphilaceae	Peptoniphilus	Peptoniphilus_harei	site	oral	-3.08628407	130	0.223076923	3.28E-05	6.64E-05
Bacteria	Bacteroidetes	Bacteroidia	Bacteroidales	Porphyromonadaceae	Porphyromonas	Porphyromonas_uenonis	site	oral	-3.066577531	130	0.207692308	3.06E-07	9.65E-07
Bacteria	Firmicutes	Firmicutes_unclassifiec	Firmicutes_unclassified	Firmicutes_unclassified	Firmicutes_unclassified	Firmicutes_bacterium_CAG_170	site	oral	-3.023711942	130	0.192307692	5.98E-07	1.79E-06
Bacteria	Firmicutes	Clostridia	Clostridiales	Eubacteriaceae	Eubacterium	Eubacterium_hallii	site	oral	-2.891756212	130	0.215384615	9.90E-08	3.59E-07
Bacteria	Firmicutes	Negativicutes	Selenomonadales	Selenomonadaceae	Mitsuokella	Mitsuokella_jalaludinii	site	oral	-2.832808227	130	0.215384615	7.36E-08	2.77E-07
Bacteria	Firmicutes	Clostridia	Clostridiales	Ruminococcaceae	Ruminococcus	Ruminococcus_bromii	site	oral	-2.797199211	130	0.184615385	8.54E-07	2.49E-06
Bacteria	Firmicutes	Clostridia	Clostridiales	Ruminococcaceae	Ruminococcaceae_uncla	Eubacterium_siraeum	site	oral	-2.74897449	130	0.184615385	2.19E-06	5.84E-06
Bacteria	Firmicutes	Clostridia	Clostridiales	Lachnospiraceae	Coprococcus	Coprococcus_catus	site	oral	-2.697011676	130	0.207692308	1.65E-07	5.48E-07
Bacteria	Firmicutes	Negativicutes	Selenomonadales	Selenomonadaceae	Mitsuokella	Mitsuokella_multacida	site	oral	-2.616177679	130	0.161538462	5.19E-06	1.29E-05
Bacteria	Firmicutes	Clostridia	Clostridiales	Eubacteriaceae	Eubacterium	Eubacterium_sp_CAG_180	site	oral	-2.608204358	130	0.215384615	9.43E-07	2.65E-06
Bacteria	Firmicutes	Tissierellia	Tissierellales	Peptoniphilaceae	Peptoniphilus	Peptoniphilus_lacrimalis	site	oral	-2.558595621	130	0.192307692	8.79E-07	2.52E-06
Bacteria	Firmicutes	Firmicutes_unclassifiec	Firmicutes_unclassified	Firmicutes_unclassified	Firmicutes_unclassified	Firmicutes_bacterium_CAG_83	site	oral	-2.556531166	130	0.261538462	1.93E-09	8.48E-09
Bacteria	Proteobacteria	Deltaproteobacteria	Desulfovibrionales	Desulfovibrionaceae	Desulfovibrio	Desulfovibrio_piger	site	oral	-2.504279903	130	0.169230769	4.45E-06	1.13E-05
Bacteria	Bacteroidetes	Bacteroidia	Bacteroidales	Porphyromonadaceae	Porphyromonas	Porphyromonas_asaccharolytica	site	oral	-2.474949667	130	0.223076923	1.25E-07	4.28E-07
Bacteria	Firmicutes	Negativicutes	Veillonellales	Veillonellaceae	Dialister	Dialister_micraerophilus	site	oral	-2.46025348	130	0.192307692	8.69E-07	2.51E-06
Bacteria	Bacteroidetes	Bacteroidia	Bacteroidales	Bacteroidaceae	Bacteroides	Bacteroides_sp_CAG_530	site	oral	-2.425208764	130	0.207692308	1.14E-07	4.00E-07
Bacteria	Firmicutes	Clostridia	Clostridiales	Ruminococcaceae	Flavonifractor	Flavonifractor_plautii	site	oral	-2.410160746	130	0.215384615	1.09E-07	3.86E-07
Bacteria	Firmicutes	Clostridia	Clostridiales	Peptostreptococcaceae	Peptostreptococcus	Peptostreptococcus_sp_MV1	site	oral	-2.385422363	130	0.215384615	2.01E-07	6.60E-07
Bacteria	Firmicutes	Erysipelotrichia	Erysipelotrichales	Erysipelotrichaceae	Holdemanella	Holdemanella_biformis	site	oral	-2.384291318	130	0.3	2.38E-11	1.38E-10
Bacteria	Bacteroidetes	Bacteroidia	Bacteroidales	Bacteroidaceae	Bacteroides	Bacteroides_fragilis	site	oral	-2.301376582	130	0.176923077	1.87E-05	4.06E-05
Bacteria	Spirochaetes	Spirochaetia	Brachyspirales	Brachyspiraceae	Brachyspira	Brachyspira_sp_CAG_700	site	oral	-2.29145183	130	0.2	9.45E-06	2.23E-05
Bacteria	Bacteroidetes	Bacteroidia	Bacteroidales	Porphyromonadaceae	Porphyromonas	Porphyromonas_sp_HMSC065F10	site	oral	-2.280807124	130	0.169230769	1.11E-05	2.54E-05
Bacteria	Bacteroidetes	Bacteroidia	Bacteroidales	Bacteroidaceae	Bacteroides	Bacteroides_stercoris	site	oral	-2.261376582	130	0.053846154	1.87E-05	4.06E-05
Bacteria	Proteobacteria	Epsilonproteobacteria	Campylobacterales	Campylobacteraceae	Campylobacter	Campylobacter_ureolyticus	site	oral	-2.226708426	130	0.192307692	2.76E-06	7.23E-06
Bacteria	Bacteroidetes	Bacteroidia	Bacteroidales	Tannerellaceae	Parabacteroides	Parabacteroides_distasonis	site	oral	-2.211367023	130	0.230769231	2.07E-07	6.73E-07
Bacteria	Firmicutes	Clostridia	Clostridiales	Ruminococcaceae	Gemmiger	Gemmiger_formicilis	site	oral	-2.200718057	130	0.238461538	2.38E-08	9.29E-08
Bacteria	Firmicutes	Bacilli	Bacillales	Bacillales_unclassified	Gemella	Gemella_asaccharolytica	site	oral	-2.115146617	130	0.161538462	5.53E-06	1.36E-05
Bacteria	Proteobacteria	Epsilonproteobacteria	Campylobacterales	Campylobacteraceae	Campylobacter	Campylobacter_hominis	site	oral	-2.109664148	130	0.184615385	7.80E-06	1.86E-05
Bacteria	Firmicutes	Clostridia	Clostridiales	Lachnospiraceae	Anaerostipes	Anaerostipes_hadrus	site	oral	-2.047673819	130	0.161538462	9.59E-06	2.25E-05
Bacteria	Actinobacteria	Actinobacteria	Corynebacteriales	Corynebacteriaceae	Corynebacterium	Corynebacterium_atypicum	site	oral	-1.989288251	130	0.123076923	0.000330391	0.000572678
Bacteria	Bacteroidetes	Bacteroidia	Bacteroidales	Bacteroidaceae	Bacteroides	Bacteroides_uniformis	site	oral	-1.983351478	130	0.176923077	1.70E-05	3.74E-05
Bacteria	Firmicutes	Clostridia	Clostridiales	Ruminococcaceae	Agathobaculum	Agathobaculum_butyriciproducens	site	oral	-1.958075172	130	0.146153846	3.84E-05	7.73E-05
Bacteria	Actinobacteria	Coriobacteriia	Coriobacteriales	Coriobacteriaceae	Collinsella	Collinsella_stercoris	site	oral	-1.856931488	130	0.192307692	7.92E-07	2.33E-06
Bacteria	Fusobacteria	Fusobacteriia	Fusobacteriales	Fusobacteriaceae	Fusobacterium	Fusobacterium_mortiferum	site	oral	-1.843255934	130	0.192307692	9.89E-06	2.29E-05
Bacteria	Firmicutes	Firmicutes_unclassifiec	Firmicutes_unclassified	Firmicutes_unclassified	Firmicutes_unclassified	Firmicutes_bacterium_CAG_110	site	oral	-1.827143421	130	0.176923077	2.26E-06	5.99E-06
Bacteria	Fusobacteria	Fusobacteriia	Fusobacteriales	Leptotrichiaceae	Sneathia	Sneathia_amnii	site	oral	-1.816525696	130	0.115384615	0.00043952	0.000721739
Bacteria	Firmicutes	Tissierellia	Tissierellales	Peptoniphilaceae	Anaerococcus	Anaerococcus_vaginalis	site	oral	-1.809390075	130	0.215384615	5.59E-07	1.69E-06
Bacteria	Bacteroidetes	Bacteroidia	Bacteroidales	Odoribacteraceae	Odoribacter	Odoribacter_splanchnicus	site	oral	-1.789791666	130	0.153846154	2.88E-05	5.96E-05
Bacteria	Firmicutes	Clostridia	Clostridiales	Peptostreptococcaceae	Criibacterium	Criibacterium_bergeronii	site	oral	-1.783321245	130	0.146153846	4.74E-05	9.48E-05
Viruses	Viruses_unclassified	Viruses_unclassified	Viruses_unclassified	Papillomaviridae	Alphapapillomavirus	Alphapapillomavirus_9	site	oral	-1.747623085	130	0.161538462	0.000497045	0.000807698
Bacteria	Fusobacteria	Fusobacteriia	Fusobacteriales	Fusobacteriaceae	Fusobacterium	Fusobacterium_equinum	site	oral	-1.747540704	130	0.138461538	0.00012399	0.000235883
Bacteria	Proteobacteria	Deltaproteobacteria	Desulfovibrionales	Desulfovibrionaceae	Desulfovibrionaceae_un	Desulfovibrionaceae_bacterium	site	oral	-1.719618971	130	0.130769231	9.04E-05	0.000175133
Bacteria	Firmicutes	Clostridia	Clostridiales	Eubacteriaceae	Eubacterium	Eubacterium_ramulus	site	oral	-1.703534385	130	0.192307692	1.81E-06	4.99E-06
Bacteria	Fusobacteria	Fusobacteriia	Fusobacteriales	Fusobacteriaceae	Fusobacterium	Fusobacterium_naviforme	site	oral	-1.675721716	130	0.1	0.001203046	0.001762208
Bacteria	Actinobacteria	Coriobacteriia	Eggerthellales	Eggerthellaceae	Slackia	Slackia_isoflavoniconvertens	site	oral	-1.653732736	130	0.238461538	3.86E-08	1.47E-07
Viruses	Viruses_unclassified	Viruses_unclassified	Caudovirales	Siphoviridae	Siphoviridae_unclassifie	Streptococcus_phage_SM1	site	oral	-1.51546515	130	0.184615385	0.009953855	0.011763647
Bacteria	Firmicutes	Clostridia	Clostridiales	Lachnospiraceae	Blautia	Blautia_obeum	site	oral	-1.47499694	130	0.169230769	7.65E-06	1.84E-05
Viruses	Viruses_unclassified	Viruses_unclassified	Viruses_unclassified	Papillomaviridae	Alphapapillomavirus	Alphapapillomavirus_7	site	oral	-1.459611779	130	0.130769231	0.000151912	0.000278803
Bacteria	Bacteroidetes	Bacteroidia	Bacteroidales	Prevotellaceae	Prevotella	Prevotella_sp_CAG_1092	site	oral	-1.389022572	130	0.169230769	1.61E-05	3.57E-05
Bacteria	Firmicutes	Clostridia	Clostridiales	Lachnospiraceae	Roseburia	Roseburia_hominis	site	oral	-1.366492504	130	0.123076923	0.000146586	0.000272231
Viruses	Viruses_unclassified	Viruses_unclassified	Viruses_unclassified	Papillomaviridae	Alphapapillomavirus	Alphapapillomavirus_3	site	oral	-1.301944374	130	0.146153846	0.000943473	0.001419567
Archaea	Euryarchaeota	Methanobacteria	Methanobacteriales	Methanobacteriaceae	Methanobrevibacter	Methanobrevibacter_smithii	site	oral	-1.290344919	130	0.146153846	4.91E-05	9.75E-05
Bacteria	Firmicutes	Clostridia	Clostridiales	Lachnospiraceae	Blautia	Ruminococcus_gnavus	site	oral	-1.21714437	130	0.1	0.001251426	0.001824509
Bacteria	Bacteroidetes	Bacteroidia	Bacteroidales	Bacteroidaceae	Bacteroides	Bacteroides_dorei	site	oral	-1.215676481	130	0.1	0.001572745	0.002230439
Bacteria	Bacteroidetes	Bacteroidia	Bacteroidales	Odoribacteraceae	Butyricimonas	Butyricimonas_virosa	site	oral	-1.214572931	130	0.123076923	0.000198788	0.000354411
Bacteria	Firmicutes	Clostridia	Clostridiales	Lachnospiraceae	Roseburia	Roseburia_intestinalis	site	oral	-1.207962072	130	0.107692308	0.00064809	0.00100101
Bacteria	Firmicutes	Tissierellia	Tissierellia_unclassified	Tissierellia_unclassified	Tissierellia_unclassified	Bacteroides_coagulans	site	oral	-1.192397689	130	0.107692308	0.000600961	0.000951777
Bacteria	Firmicutes	Clostridia	Clostridiales	Lachnospiraceae	Coprococcus	Coprococcus_eutactus	site	oral	-1.151053236	130	0.107692308	0.000613889	0.000966232
Bacteria	Spirochaetes	Spirochaetia	Brachyspirales	Brachyspiraceae	Brachyspira	Brachyspira_pilosicoli	site	oral	-1.147508333	130	0.107692308	0.000570948	0.000918226
Bacteria	Bacteroidetes	Bacteroidia	Bacteroidales	Rikenellaceae	Alistipes	Alistipes_putredinis	site	oral	-1.134774372	130	0.092307692	0.001999622	0.002736325
Viruses	Viruses_unclassified	Viruses_unclassified	Viruses_unclassified	Papillomaviridae	Alphapapillomavirus	Alphapapillomavirus_5	site	oral	-1.124184031	130	0.107692308	0.005056149	0.006438852
Bacteria	Actinobacteria	Actinobacteria	Bifidobacteriales	Bifidobacteriaceae	Gardnerella	Gardnerella_vaginalis	site	oral	-1.112002228	130	0.130769231	0.000207385	0.000367637
Bacteria	Firmicutes	Clostridia	Clostridiales	Ruminococcaceae	Ruthenibacterium	Ruthenibacterium_lactatiformans	site	oral	-1.092146394	130	0.123076923	0.000616283	0.000966232
Bacteria	Bacteroidetes	Bacteroidia	Bacteroidales	Bacteroidaceae	Bacteroides	Bacteroides_thetaiotaomicron	site	oral	-1.076953037	130	0.092307692	0.002435372	0.003318061
Bacteria	Firmicutes	Clostridia	Clostridiales	Hungateiclostridiaceae	Mageeibacillus	Mageeibacillus_indolicus	site	oral	-1.060574784	130	0.107692308	0.001364979	0.001971636
Bacteria	Bacteroidetes	Bacteroidia	Bacteroidales	Rikenellaceae	Alistipes	Alistipes_shahii	site	oral	-1.041972596	130	0.138461538	0.000148156	0.00027352
Bacteria	Firmicutes	Tissierellia	Tissierellales	Peptoniphilaceae	Peptoniphilus	Peptoniphilus_asaccharolyticus	site	oral	-1.040515745	130	0.092307692	0.002644717	0.003562029
Bacteria	Firmicutes	Clostridia	Clostridiales	Ruminococcaceae	Ruminococcus	Ruminococcus_bicirculans	site	oral	-1.015311014	130	0.076923077	0.004995583	0.006387795
Bacteria	Firmicutes	Clostridia	Clostridiales	Lachnospiraceae	Lachnospira	Lachnospira_pectinoschiza	site	oral	-1.004370885	130	0.115384615	0.000309357	0.000539214
Bacteria	Bacteroidetes	Bacteroidia	Bacteroidales	Prevotellaceae	Prevotella	Prevotella_colorans	site	oral	-0.992224034	130	0.084615385	0.005254375	0.006637105
Bacteria	Firmicutes	Clostridia	Clostridiales	Eubacteriaceae	Eubacterium	Eubacterium_sp_CAG_251	site	oral	-0.974115444	130	0.1	0.000981923	0.001458857
Bacteria	Bacteroidetes	Bacteroidia	Bacteroidales	Tannerellaceae	Parabacteroides	Parabacteroides_merdae	site	oral	-0.956032247	130	0.130769231	0.000353848	0.000603281

Bacteria	Tenericutes	Mollicutes	Mycoplasmatales	Mycoplasmataceae	Mycoplasma	Mycoplasma_hominis	site	oral	-0.951438203	130	0.076923077	0.004057447	0.005209562
Viruses	Viruses_unclassified	Viruses_unclassified	Viruses_unclassified	Papillomaviridae	Alphapapillomavirus	Alphapapillomavirus_11	site	oral	-0.945617865	130	0.130769231	0.000270193	0.000473597
Bacteria	Firmicutes	Clostridia	Clostridiales	Eubacteriaceae	Eubacterium	Eubacterium_eligens	site	oral	-0.917402777	130	0.115384615	0.000744215	0.001138212
Bacteria	Proteobacteria	Gammaproteobacteria	Aeromonadales	Succinivibrionaceae	Anaerobiospirillum	Anaerobiospirillum_thomasii	site	oral	-0.906450626	130	0.107692308	0.000465235	0.000759966
Viruses	Viruses_unclassified	Viruses_unclassified	Viruses_unclassified	Papillomaviridae	Alphapapillomavirus	Alphapapillomavirus_14	site	oral	-0.882552855	130	0.092307692	0.006611317	0.008218051
Bacteria	Firmicutes	Tissierellia	Tissierellales	Peptoniphilaceae	Peptoniphilus	Peptoniphilus_sp_HMSC062D09	site	oral	-0.877216423	130	0.123076923	0.006596245	0.008218051
Bacteria	Actinobacteria	Coriobacteriia	Coriobacteriales	Atopobiaceae	Atopobium	Atopobium_minutum	site	oral	-0.839432427	130	0.084615385	0.003637249	0.004768159
Bacteria	Actinobacteria	Coriobacteriia	Coriobacteriales	Atopobiaceae	Atopobium	Atopobium_vaginae	site	oral	-0.823687321	130	0.069230769	0.010348868	0.012138522
Bacteria	Actinobacteria	Actinobacteria	Corynebacteriales	Corynebacteriaceae	Corynebacterium	Corynebacterium_amycolatum	site	oral	-0.818078738	130	0.084615385	0.028695462	0.031861153
Viruses	Viruses_unclassified	Viruses_unclassified	Viruses_unclassified	Papillomaviridae	Alphapapillomavirus	Alphapapillomavirus_6	site	oral	-0.812448471	130	0.123076923	0.003532731	0.004650684
Bacteria	Actinobacteria	Coriobacteriia	Coriobacteriales	Atopobiaceae	Atopobium	Atopobium_deltae	site	oral	-0.78326001	130	0.1	0.001514527	0.00216758
Eukaryota	Eukaryota_unclassifie	Eukaryota_unclassified	Eukaryota_unclassified	Eukaryota_unclassified	Blastocystis	Blastocystis_sp_subtype_1	site	oral	-0.770864319	130	0.069230769	0.009497644	0.011267167
Bacteria	Firmicutes	Negativicutes	Acidaminococcales	Acidaminococcaceae	Acidaminococcus	Acidaminococcus_fermentans	site	oral	-0.757178528	130	0.1	0.001310428	0.001901644
Bacteria	Actinobacteria	Actinobacteria	Bifidobacteriales	Bifidobacteriaceae	Bifidobacterium	Bifidobacterium_longum	site	oral	-0.74618729	130	0.107692308	0.006778393	0.008392296
Viruses	Viruses_unclassified	Viruses_unclassified	Viruses_unclassified	Papillomaviridae	Alphapapillomavirus	Alphapapillomavirus_8	site	oral	-0.706102654	130	0.053846154	0.027762544	0.030935407
Bacteria	Bacteroidetes	Bacteroidia	Bacteroidales	Bacteroidaceae	Bacteroides	Bacteroides_caccae	site	oral	-0.703325413	130	0.069230769	0.009249193	0.011099032
Viruses	Viruses_unclassified	Viruses_unclassified	Herpesvirales	Herpesviridae	Simplexvirus	Human_alphaherpesvirus_2	site	oral	-0.700824325	130	0.092307692	0.013754268	0.015776955
Bacteria	Firmicutes	Clostridia	Clostridiales	Lachnospiraceae	Roseburia	Roseburia_sp_CAG_471	site	oral	-0.684359179	130	0.061538462	0.017972283	0.020539752
Bacteria	Bacteroidetes	Bacteroidia	Bacteroidales	Bacteroidaceae	Bacteroides	Bacteroides_ovatus	site	oral	-0.678525136	130	0.092307692	0.003297934	0.00435998
Bacteria	Actinobacteria	Actinobacteria	Corynebacteriales	Corynebacteriales_unclassi	Lawsonella	Lawsonella_clevelandensis	site	oral	-0.662105898	130	0.084615385	0.003999823	0.005178194
Bacteria	Firmicutes	Clostridia	Clostridiales	Clostridiales_unclassified	Lawsonibacter	Lawsonibacter_asaccharolyticus	site	oral	-0.648871572	130	0.084615385	0.003760357	0.004908918
Bacteria	Firmicutes	Tissierellia	Tissierellales	Peptoniphilaceae	Peptoniphilus	Peptoniphilus_duerdenii	site	oral	-0.643802782	130	0.1	0.001061252	0.001561842
Viruses	Viruses_unclassified	Viruses_unclassified	Viruses_unclassified	Adenoviridae	Mastadenovirus	Human_mastadenovirus_D	site	oral	-0.616278184	130	0.053846154	0.031420178	0.034396826
Bacteria	Firmicutes	Clostridia	Clostridiales	Lachnospiraceae	Lachnoclostridium	Clostridium_bolteae	site	oral	-0.612688206	130	0.069230769	0.011001517	0.012807737
Bacteria	Bacteroidetes	Bacteroidia	Bacteroidales	Prevotellaceae	Paraprevotella	Paraprevotella_xylaniphila	site	oral	-0.600094398	130	0.069230769	0.012116139	0.014000872
Viruses	Viruses_unclassified	Viruses_unclassified	Herpesvirales	Herpesviridae	Simplexvirus	Human_alphaherpesvirus_1	site	oral	-0.595688613	130	0.076923077	0.074319598	0.079138958
Bacteria	Firmicutes	Bacilli	Lactobacillales	Streptococcaceae	Streptococcus	Streptococcus_thermophilus	site	oral	-0.581141273	130	0.076923077	0.217631241	0.227093469
Bacteria	Actinobacteria	Coriobacteriia	Coriobacteriales	Coriobacteriaceae	Enorma	Collinsella_massiliensis	site	oral	-0.562699918	130	0.084615385	0.003246775	0.004329034
Bacteria	Tenericutes	Mollicutes	Mycoplasmatales	Mycoplasmataceae	Ureaplasma	Ureaplasma_urealyticum	site	oral	-0.548779613	130	0.069230769	0.008728135	0.010514201
Bacteria	Firmicutes	Firmicutes_unclassifiec	Firmicutes_unclassified	Firmicutes_unclassified	Firmicutes_unclassified	Firmicutes_bacterium_CAG_791	site	oral	-0.548148427	130	0.1	0.000927332	0.001404503
Bacteria	Firmicutes	Bacilli	Lactobacillales	Streptococcaceae	Streptococcus	Streptococcus_milleri	site	oral	-0.540528697	130	0.061538462	0.032616387	0.035581513
Bacteria	Bacteroidetes	Bacteroidia	Bacteroidales	Bacteroidaceae	Bacteroides	Bacteroides_xylanisolvens	site	oral	-0.532867629	130	0.069230769	0.008375824	0.010128903
Bacteria	Proteobacteria	Betaproteobacteria	Burkholderiales	Sutterellaceae	Sutterella	Sutterella_parvirubra	site	oral	-0.525069211	130	0.1	0.020348105	0.022973348
Bacteria	Bacteroidetes	Bacteroidia	Bacteroidales	Prevotellaceae	Prevotella	Prevotella_sp_CAG_873	site	oral	-0.519087635	130	0.053846154	0.028923006	0.031999921
Bacteria	Firmicutes	Negativicutes	Veillonellales	Veillonellaceae	Negativicoccus	Negativicoccus_succinicivorans	site	oral	-0.51435049	130	0.061538462	0.019587036	0.022222382
Bacteria	Firmicutes	Erysipelotrichia	Erysipelotrichales	Erysipelotrichaceae	Coprobacillus	Coprobacillus_cateniformis	site	oral	-0.513540622	130	0.053846154	0.031039083	0.034099275
Bacteria	Firmicutes	Clostridia	Clostridiales	Ruminococcaceae	Ruminococcus	Ruminococcus_callidus	site	oral	-0.458739848	130	0.076923077	0.006997	0.008527594
Bacteria	Actinobacteria	Actinobacteria	Corynebacteriales	Corynebacteriaceae	Corynebacterium	Corynebacterium_aurimucosum	site	oral	-0.455786954	130	0.084615385	0.042527973	0.045754233
Bacteria	Proteobacteria	Deltaproteobacteria	Desulfovibrionales	Desulfovibrionaceae	Bilophila	Bilophila_wadsworthia	site	oral	-0.455227077	130	0.076923077	0.005412771	0.006809615
Bacteria	Firmicutes	Bacilli	Bacillales	Staphylococcaceae	Staphylococcus	Staphylococcus_hominis	site	oral	-0.439720348	130	0.061538462	0.065607847	0.070101535
Viruses	Viruses_unclassified	Viruses_unclassified	Viruses_unclassified	Papillomaviridae	Alphapapillomavirus	Alphapapillomavirus_13	site	oral	-0.401190299	130	0.061538462	0.023753414	0.026658508
Bacteria	Actinobacteria	Coriobacteriia	Coriobacteriales	Atopobiaceae	Olsenella	Olsenella_scatoligenes	site	oral	-0.375389655	130	0.069230769	0.010716054	0.01252213
Bacteria	Firmicutes	Bacilli	Lactobacillales	Streptococcaceae	Streptococcus	Streptococcus_anginosus_group	site	oral	-0.390501245	130	0.169230769	0.147606576	0.247622115
Bacteria	Proteobacteria	Betaproteobacteria	Neisseriales	Neisseriaceae	Neisseria	Neisseria_gonorrhoeae	site	oral	-0.354740053	130	0.053846154	0.191568713	0.201243901
Bacteria	Firmicutes	Clostridia	Clostridiales	Ruminococcaceae	Ruminococcus	Ruminococcus_sp_CAG_330	site	oral	-0.287634237	130	0.053846154	0.034481304	0.037354746
Bacteria	Bacteroidetes	Bacteroidia	Bacteroidales	Prevotellaceae	Prevotella	Prevotella_sp_S7_1_8	site	oral	-0.25831086	130	0.053846154	0.033293203	0.036193308
Bacteria	Firmicutes	Negativicutes	Veillonellales	Veillonellaceae	Veillonella	Veillonella_rogosae	site	oral	0.1813299	130	0.053846154	0.029600067	0.032633289
Bacteria	Firmicutes	Clostridia	Clostridiales	Clostridiales_Family_XIII_Ir	Mogibacterium	Mogibacterium_diversum	site	oral	0.194732116	130	0.061538462	0.099989241	0.106111031
Viruses	Viruses_unclassified	Viruses_unclassified	Caudovirales	Siphoviridae	Sfi21dt1virus	Streptococcus_virus_Sfi21	site	oral	0.228986083	130	0.061538462	0.213125958	0.223138587
Bacteria	Bacteroidetes	Bacteroidia	Bacteroidales	Prevotellaceae	Prevotella	Prevotella_baroniae	site	oral	0.306593851	130	0.053846154	0.011254385	0.013053413
Bacteria	Firmicutes	Erysipelotrichia	Erysipelotrichales	Erysipelotrichaceae	Bulleidia	Bulleidia_extructa	site	oral	0.308752232	130	0.053846154	0.020396209	0.022973348
Viruses	Viruses_unclassified	Viruses_unclassified	Herpesvirales	Herpesviridae	Roseolovirus	Human_betaherpesvirus_6B	site	oral	0.318271147	130	0.076923077	0.143832167	0.1521208
Bacteria	Fusobacteria	Fusobacteriia	Fusobacteriales	Leptotrichiaceae	Leptotrichia	Leptotrichia_sp_oral_taxon_212	site	oral	0.335433486	130	0.053846154	0.007085755	0.008602162
Bacteria	Firmicutes	Bacilli	Lactobacillales	Streptococcaceae	Streptococcus	Streptococcus_mutans	site	oral	0.34711722	130	0.053846154	0.006843054	0.008405641
Bacteria	Firmicutes	Bacilli	Lactobacillales	Carnobacteriaceae	Granulicatella	Granulicatella_adiacens	site	oral	0.357209498	130	0.053846154	0.006831826	0.008405641
Bacteria	Firmicutes	Bacilli	Lactobacillales	Streptococcaceae	Streptococcus	Streptococcus_sp_HMSC070B10	site	oral	0.416875744	130	0.053846154	0.041456197	0.044755479
Bacteria	Proteobacteria	Betaproteobacteria	Neisseriales	Neisseriaceae	Neisseria	Neisseria_subflava	site	oral	0.426054193	130	0.084615385	0.000517389	0.000836401
Viruses	Viruses_unclassified	Viruses_unclassified	Caudovirales	Siphoviridae	Siphoviridae_unclassifie	Streptococcus_phage_phiNJ2	site	oral	0.44301371	130	0.076923077	0.010241449	0.012057857
Bacteria	Firmicutes	Bacilli	Lactobacillales	Aerococcaceae	Abiotrophia	Abiotrophia_defectiva	site	oral	0.448718976	130	0.069230769	0.001821249	0.002548116
Bacteria	Proteobacteria	Betaproteobacteria	Neisseriales	Neisseriaceae	Neisseria	Neisseria_perflava	site	oral	0.4527395	130	0.053846154	0.009333286	0.011157032
Bacteria	Synergistetes	Synergistia	Synergistales	Synergistaceae	Fretibacterium	Fretibacterium_fastidiosum	site	oral	0.452827546	130	0.076923077	0.059841155	0.064159589
Bacteria	Bacteroidetes	Bacteroidia	Bacteroidales	Prevotellaceae	Alloprevotella	Alloprevotella_rava	site	oral	0.458585314	130	0.069230769	0.001857652	0.002587444
Bacteria	Bacteroidetes	Flavobacteriia	Flavobacteriales	Flavobacteriaceae	Capnocytophaga	Capnocytophaga_sp_oral_taxon_332	site	oral	0.482584629	130	0.069230769	0.003292836	0.00435998
Bacteria	Spirochaetes	Spirochaetia	Spirochaetales	Spirochaetaceae	Treponema	Treponema_medium	site	oral	0.486314266	130	0.053846154	0.018304514	0.020843096
Bacteria	Firmicutes	Clostridia	Clostridiales	Lachnospiraceae	Lachnospiraceae_unclas	Lachnospiraceae_bacterium_oral_taxor	site	oral	0.501308581	130	0.061538462	0.005246245	0.006637105
Bacteria	Bacteroidetes	Bacteroidia	Bacteroidales	Prevotellaceae	Prevotella	Prevotella_fusca	site	oral	0.508743409	130	0.069230769	0.002648688	0.003562029
Bacteria	Fusobacteria	Fusobacteriia	Fusobacteriales	Leptotrichiaceae	Leptotrichia	Leptotrichia_buccalis	site	oral	0.527158547	130	0.076923077	0.002692718	0.003605699
Bacteria	Bacteroidetes	Bacteroidia	Bacteroidales	Prevotellaceae	Prevotella	Prevotella_veroralis	site	oral	0.531549016	130	0.092307692	0.00018111	0.000326626
Bacteria	Firmicutes	Negativicutes	Veillonellales	Veillonellaceae	Megasphaera	Megasphaera_micronuciformis	site	oral	0.54130851	130	0.053846154	0.006977397	0.008527594
Viruses	Viruses_unclassified	Viruses_unclassified	Herpesvirales	Herpesviridae	Rhadinovirus	Human_gammaherpesvirus_8	site	oral	0.543413147	130	0.076923077	0.009462699	0.011267167
Viruses	Viruses_unclassified	Viruses_unclassified	Caudovirales	Siphoviridae	Siphoviridae_unclassifie	Microbacterium_phage_Min1	site	oral	0.568977025	130	0.076923077	0.004022154	0.005185587
Bacteria	Firmicutes	Bacilli	Lactobacillales	Streptococcaceae	Streptococcus	Streptococcus_vestibularis	site	oral	0.578350979	130	0.061538462	0.01367022	0.015738408
Viruses	Viruses_unclassified	Viruses_unclassified	Caudovirales	Siphoviridae	Sfi21dt1virus	Streptococcus_virus_phiAbc2	site	oral	0.583130474	130	0.076923077	0.024456198	0.027348866
Bacteria	Actinobacteria	Actinobacteria	Actinomycetales	Actinomycetaceae	Actinomyces	Actinomyces_sp_oral_taxon_181	site	oral	0.597089791	130	0.061538462	0.003778938	0.004912619
Bacteria	Bacteroidetes	Bacteroidia	Bacteroidales	Prevotellaceae	Prevotella	Prevotella_scopos	site	oral	0.614464298	130	0.084615385	0.000643207	0.000998411
Bacteria	Proteobacteria	Betaproteobacteria	Neisseriales	Neisseriaceae	Kingella	Kingella_denitrificans	site	oral	0.621512546	130	0.076923077	0.001375076	0.001977067
Bacteria	Proteobacteria	Gammaproteobacteria	Cardiobacteriales	Cardiobacteriaceae	Cardiobacterium	Cardiobacterium_valvarum	site	oral	0.625791732	130	0.084615385	0.000661033	0.001015972
Bacteria	Fusobacteria	Fusobacteriia	Fusobacteriales	Leptotrichiaceae	Leptotrichia	Leptotrichia_sp_oral_taxon_225	site	oral	0.634223474	130	0.076923077	0.000946378	0.001419567
Bacteria	Firmicutes	Bacilli	Lactobacillales	Aerococcaceae	Abiotrophia	Abiotrophia_sp_HMSC24B09	site	oral	0.63811235	130	0.084615385	0.000377462	0.000639045
Bacteria	Proteobacteria	Gammaproteobacteria	Cardiobacteriales	Cardiobacteriaceae	Cardiobacterium	Cardiobacterium_hominis	site	oral	0.653656863	130	0.069230769	0.001556562	0.002217567
Bacteria	Firmicutes	Clostridia	Clostridiales	Peptostreptococcaceae	Filifactor	Filifactor_alocis	site	oral	0.657850996	130	0.092307692	0.000335046	0.000577538
Bacteria	Proteobacteria	Betaproteobacteria	Neisseriales	Neisseriaceae	Morococcus	Morococcus_cerebrosus	site	oral	0.664332494	130	0.069230769	0.001937012	0.002685991
Bacteria	Firmicutes	Bacilli	Lactobacillales	Streptococcaceae	Streptococcus	Streptococcus_australis	site	oral	0.67368646	130	0.092307692	0.000198562	0.000354411
Bacteria	Actinobacteria	Actinobacteria	Actinomycetales	Actinomycetaceae	Actinomyces	Actinomyces_sp_oral_taxon_180	site	oral	0.675333956	130	0.076923077	0.000964136	0.001439285
Bacteria	Firmicutes	Negativicutes	Selenomonadales	Selenomonadaceae	Selenomonas	Selenomonas_noxia	site	oral	0.683678806	130	0.092307692	0.000351505	0.000602579
Bacteria	Bacteroidetes	Bacteroidia	Bacteroidales	Prevotellaceae	Prevotella	Prevotella_shahii	site	oral	0.756545353	130	0.084615385	0.000625646	0.000976008
Bacteria	Bacteroidetes	Bacteroidia	Bacteroidales	Prevotellaceae	Prevotella	Prevotella_pleuritidis	site	oral	0.758977983	130	0.1	0.000169647	0.000307733
Bacteria	Actinobacteria	Actinobacteria	Actinomycetales	Actinomycetaceae	Actinomyces	Actinomyces_sp_oral_taxon_170	site	oral	0.771150092	130	0.053846154	0.00593681	0.007438895
Bacteria	Fusobacteria	Fusobacteriia	Fusobacteriales	Leptotrichiaceae	Leptotrichia	Leptotrichia_sp_oral_taxon_498	site	oral	0.783220927	130	0.076923077	0.001720249	0.002417647
Bacteria	Bacteroidetes	Bacteroidetes_unclassi	Bacteroidetes_unclassif	Bacteroidetes_unclassified	Bacteroidetes_unclassifi	Bacteroidetes_oral_taxon_274	site	oral	0.789489415	130	0.069230769	0.001976781	0.002721958
Bacteria	Firmicutes	Bacilli	Lactobacillales	Streptococcaceae	Streptococcus	Streptococcus_sp_M334	site	oral	0.795223282	130	0.084615385	0.001980399	0.002721958
Bacteria	Firmicutes	Negativicutes	Veillonellales	Veillonellaceae	Veillonella	Veillonella_infantium	site	oral	0.805895951	130	0.146153846	0.000378921	0.000639045
Bacteria	Actinobacteria	Actinobacteria	Actinomycetales	Actinomycetaceae	Actinomyces	Actinomyces_sp_oral_taxon_897	site	oral	0.819056558	130	0.069230769	0.002513487	0.003409599
Bacteria	Proteobacteria	Betaproteobacteria	Neisseriales	Neisseriaceae	Neisseria	Neisseria_macacae	site	oral	0.819139303	130	0.138461538	0.000143885	0.000268816
Bacteria	Proteobacteria	Betaproteobacteria	Neisseriales	Neisseriaceae	Kingella	Kingella_oralis	site	oral	0.844960852	130	0.130769231	7.38E-06	1.80E-05
Bacteria	Actinobacteria	Actinobacteria	Actinomycetales	Actinomycetaceae	Actinomyces	Actinomyces_sp_HMSC035G02	site	oral	0.845587109	130	0.123076923	6.35E-05	0.000124599
Bacteria	Firmicutes	Bacilli	Lactobacillales	Streptococcaceae	Streptococcus	Streptococcus_salivarius	site	oral	0.847972546	130	0.253846154	0.169996983	0.179186009
Bacteria	Firmicutes	Clostridia	Clostridiales	Lachnospiraceae	Stomatobaculum	Stomatobaculum_longum	site	oral	0.852665665	130	0.084615385	0.000580789	0.000929263
Bacteria	Firmicutes	Clostridia	Clostridiales	Lachnospiraceae	Oribacterium	Oribacterium_sinus	site	oral	0.854678928	130	0.053846154	0.044481304	0.047354746
Bacteria	Actinobacteria	Actinobacteria	Actinomycetales	Actinomycetaceae	Actinomyces	Actinomyces_viscosus	site	oral	0.877614357	130	0.1	0.000225527	0.00039754
Bacteria	Proteobacteria	Epsilonproteobacteria	Campylobacterales	Campylobacteraceae	Campylobacter	Campylobacter_gracilis	site	oral	0.895410043	130	0.084615385	0.000437059	0.000721494
Bacteria	Spirochaetes	Spirochaetia	Spirochaetales	Spirochaetaceae	Treponema	Treponema_denticola	site	oral	0.905200445	130	0.107692308	8.31E-05	0.000161963
Bacteria	Fusobacteria	Fusobacteriia	Fusobacteriales	Leptotrichiaceae	Leptotrichia	Leptotrichia_sp_oral_taxon_215	site	oral	0.91026489	130	0.084615385	0.000590047	0.000939259
Bacteria	Firmicutes	Negativicutes	Veillonellales	Veillonellaceae	Veillonella	Veillonella_sp_T11011_6	site	oral	0.914678928	130	0.115384615	0.001022684	0.001512215
Bacteria	Firmicutes	Bacilli	Lactobacillales	Streptococcaceae	Streptococcus	Streptococcus_sp_A12	site	oral	0.934861602	130	0.107692308	0.000400663	0.000672079
Bacteria	Firmicutes	Negativicutes	Selenomonadales	Selenomonadaceae	Selenomonas	Selenomonas_sputigena	site	oral	0.946050077	130	0.069230769	0.001650209	0.002329707
Bacteria	Actinobacteria	Actinobacteria	Actinomycetales	Actinomycetaceae	Actinomyces	Actinomyces_sp_oral_taxon_448	site	oral	1.05924207	130	0.138461538	1.47E-05	3.27E-05
Bacteria	Bacteroidetes	Bacteroidia	Bacteroidales	Prevotellaceae	Prevotella	Prevotella_jejuni	site	oral	1.09918711	130	0.153846154	2.33E-05	4.89E-05
Bacteria	Proteobacteria	Betaproteobacteria	Neisseriales	Neisseriaceae	Neisseria	Neisseria_bacilliformis	site	oral	1.109308163	130	0.084615385	0.000804896	0.001225012
Bacteria	Actinobacteria	Actinobacteria	Propionibacteriales	Propionibacteriaceae	Pseudopropionibacteriu	Pseudopropionibacterium_propionicum	site	oral	1.118537191	130	0.084615385	0.000407438	0.000679789
Bacteria	Bacteroidetes	Bacteroidia	Bacteroidales	Prevotellaceae	Prevotella	Prevotella_sp_oral_taxon_306	site	oral	1.149517297	130	0.123076923	2.04E-05	4.34E-05

Bacteria	Bacteroidetes	Bacteroidia	Bacteroidales	Prevotellaceae	Prevotella	Prevotella_sp_F0091	site	oral	1.165552562	130	0.123076923	3.25E-05	6.63E-05
Viruses	Viruses_unclassified	Viruses_unclassified	Caudovirales	Myoviridae	Myoviridae_unclassified	Haemophilus_phage_Aaphi23	site	oral	1.169335211	130	0.130769231	4.38E-06	1.12E-05
Bacteria	Bacteroidetes	Bacteroidia	Bacteroidales	Prevotellaceae	Prevotella	Prevotella_oulorum	site	oral	1.181883755	130	0.130769231	1.92E-05	4.12E-05
Bacteria	Bacteroidetes	Bacteroidia	Bacteroidales	Tannerellaceae	Tannerella	Tannerella_forsythia	site	oral	1.196543515	130	0.115384615	2.02E-05	4.32E-05
Bacteria	Bacteroidetes	Bacteroidia	Bacteroidales	Prevotellaceae	Prevotella	Prevotella_salivae	site	oral	1.201696303	130	0.123076923	2.34E-05	4.89E-05
Bacteria	Firmicutes	Clostridia	Clostridiales	Lachnospiraceae	Lachnoanaerobaculum	Lachnoanaerobaculum_saburreum	site	oral	1.209810791	130	0.1	0.000103544	0.000199418
Bacteria	Fusobacteria	Fusobacteriia	Fusobacteriales	Leptotrichiaceae	Leptotrichia	Leptotrichia_hofstadii	site	oral	1.2685526	130	0.138461538	2.02E-06	5.48E-06
Bacteria	Proteobacteria	Betaproteobacteria	Neisseriales	Neisseriaceae	Eikenella	Eikenella_corrodens	site	oral	1.283081486	130	0.130769231	4.47E-06	1.13E-05
Bacteria	Bacteroidetes	Flavobacteriia	Flavobacteriales	Flavobacteriaceae	Capnocytophaga	Capnocytophaga_sputigena	site	oral	1.30581044	130	0.161538462	2.28E-07	7.34E-07
Bacteria	Actinobacteria	Actinobacteria	Actinomycetales	Actinomycetaceae	Actinomyces	Actinomyces_massiliensis	site	oral	1.307592497	130	0.130769231	3.21E-06	8.35E-06
Viruses	Viruses_unclassified	Viruses_unclassified	Caudovirales	Siphoviridae	Siphoviridae_unclassifie	Streptococcus_phage_Dp_1	site	oral	1.317698493	130	0.153846154	1.76E-05	3.84E-05
Bacteria	Proteobacteria	Gammaproteobacteria	Pasteurellales	Pasteurellaceae	Haemophilus	Haemophilus_paraphrohaemolyticus	site	oral	1.320680952	130	0.123076923	0.000120635	0.00023091
Bacteria	Proteobacteria	Epsilonproteobacteria	Campylobacterales	Campylobacteraceae	Campylobacter	Campylobacter_rectus	site	oral	1.349502255	130	0.138461538	3.77E-06	9.71E-06
Bacteria	Firmicutes	Bacilli	Lactobacillales	Streptococcaceae	Streptococcus	Streptococcus_pseudopneumoniae	site	oral	1.351422566	130	0.192307692	5.75E-05	0.000113446
Bacteria	Actinobacteria	Actinobacteria	Corynebacteriales	Corynebacteriaceae	Corynebacterium	Corynebacterium_durum	site	oral	1.436306104	130	0.123076923	1.16E-05	2.63E-05
Bacteria	Proteobacteria	Betaproteobacteria	Neisseriales	Neisseriaceae	Neisseria	Neisseria_sp_oral_taxon_014	site	oral	1.447377746	130	0.176923077	1.43E-07	4.80E-07
Bacteria	Bacteroidetes	Bacteroidia	Bacteroidales	Prevotellaceae	Alloprevotella	Prevotella_sp_oral_taxon_473	site	oral	1.455492552	130	0.169230769	3.50E-07	1.09E-06
Bacteria	Bacteroidetes	Flavobacteriia	Flavobacteriales	Flavobacteriaceae	Capnocytophaga	Capnocytophaga_granulosa	site	oral	1.458625216	130	0.153846154	8.88E-07	2.52E-06
Bacteria	Bacteroidetes	Bacteroidia	Bacteroidales	Prevotellaceae	Prevotella	Prevotella_oris	site	oral	1.546624089	130	0.176923077	9.37E-08	3.44E-07
Bacteria	Actinobacteria	Actinobacteria	Actinomycetales	Actinomycetaceae	Actinomyces	Actinomyces_sp_oral_taxon_414	site	oral	1.555967591	130	0.123076923	1.22E-05	2.76E-05
Bacteria	Proteobacteria	Epsilonproteobacteria	Campylobacterales	Campylobacteraceae	Campylobacter	Campylobacter_showae	site	oral	1.560588669	130	0.115384615	3.07E-05	6.30E-05
Bacteria	Actinobacteria	Actinobacteria	Actinomycetales	Actinomycetaceae	Actinomyces	Actinomyces_naeslundii	site	oral	1.566453262	130	0.207692308	5.14E-09	2.17E-08
Bacteria	Proteobacteria	Gammaproteobacteria	Pasteurellales	Pasteurellaceae	Haemophilus	Haemophilus_sp_HMSC71H05	site	oral	1.598727432	130	0.176923077	0.000126331	0.000238881
Bacteria	Firmicutes	Negativicutes	Veillonellales	Veillonellaceae	Veillonella	Veillonella_atypica	site	oral	1.647605435	130	0.2	0.00014038	0.000263847
Bacteria	Actinobacteria	Actinobacteria	Actinomycetales	Actinomycetaceae	Actinobaculum	Actinobaculum_sp_oral_taxon_183	site	oral	1.658918436	130	0.169230769	2.44E-07	7.78E-07
Bacteria	Actinobacteria	Actinobacteria	Actinomycetales	Actinomycetaceae	Actinomyces	Actinomyces_johnsonii	site	oral	1.68190603	130	0.123076923	1.25E-05	2.82E-05
Bacteria	Bacteroidetes	Bacteroidia	Bacteroidales	Porphyromonadaceae	Porphyromonas	Porphyromonas_sp_oral_taxon_278	site	oral	1.708488565	130	0.138461538	2.11E-06	5.67E-06
Bacteria	Bacteroidetes	Flavobacteriia	Flavobacteriales	Flavobacteriaceae	Capnocytophaga	Capnocytophaga_ochracea	site	oral	1.803697483	130	0.138461538	1.28E-06	3.56E-06
Bacteria	Actinobacteria	Actinobacteria	Actinomycetales	Actinomycetaceae	Actinomyces	Actinomyces_graevenitzii	site	oral	1.81619017	130	0.130769231	4.63E-06	1.16E-05
Bacteria	Proteobacteria	Gammaproteobacteria	Pasteurellales	Pasteurellaceae	Haemophilus	Haemophilus_sputorum	site	oral	1.833945963	130	0.146153846	4.25E-07	1.31E-06
Bacteria	Firmicutes	Negativicutes	Veillonellales	Veillonellaceae	Veillonella	Veillonella_dispar	site	oral	1.845702501	130	0.184615385	7.56E-06	1.83E-05
Bacteria	Bacteroidetes	Bacteroidia	Bacteroidales	Prevotellaceae	Prevotella	Prevotella_histicola	site	oral	1.864174349	130	0.153846154	4.28E-07	1.31E-06
Bacteria	Proteobacteria	Betaproteobacteria	Neisseriales	Neisseriaceae	Neisseria	Neisseria_elongata	site	oral	1.90481853	130	0.184615385	2.54E-08	9.79E-08
Bacteria	Firmicutes	Bacilli	Lactobacillales	Streptococcaceae	Streptococcus	Streptococcus_cristatus	site	oral	1.916976256	130	0.176923077	9.42E-09	3.82E-08
Bacteria	Bacteroidetes	Bacteroidia	Bacteroidales	Prevotellaceae	Prevotella	Prevotella_sp_oral_taxon_299	site	oral	1.952703525	130	0.115384615	2.48E-05	5.16E-05
Bacteria	Proteobacteria	Gammaproteobacteria	Pasteurellales	Pasteurellaceae	Haemophilus	Haemophilus_quentini	site	oral	2.015337813	130	0.215384615	0.000155691	0.000284069
Bacteria	Bacteroidetes	Bacteroidia	Bacteroidales	Prevotellaceae	Prevotella	Prevotella_pallens	site	oral	2.078068532	130	0.169230769	7.36E-07	2.19E-06
Bacteria	Proteobacteria	Gammaproteobacteria	Pasteurellales	Pasteurellaceae	Aggregatibacter	Aggregatibacter_segnis	site	oral	2.092760407	130	0.2	7.72E-10	3.65E-09
Bacteria	Bacteroidetes	Bacteroidia	Bacteroidales	Prevotellaceae	Prevotella	Prevotella_denticola	site	oral	2.09463448	130	0.207692308	8.80E-10	4.10E-09
Bacteria	Bacteroidetes	Bacteroidia	Bacteroidales	Prevotellaceae	Prevotella	Prevotella_nigrescens	site	oral	2.105473288	130	0.238461538	1.02E-10	5.24E-10
Bacteria	Bacteroidetes	Flavobacteriia	Flavobacteriales	Flavobacteriaceae	Capnocytophaga	Capnocytophaga_gingivalis	site	oral	2.110811361	130	0.192307692	1.49E-09	6.65E-09
Bacteria	Proteobacteria	Betaproteobacteria	Neisseriales	Neisseriaceae	Neisseria	Neisseria_cinerea	site	oral	2.162350933	130	0.146153846	1.90E-06	5.20E-06
Viruses	Viruses_unclassified	Viruses_unclassified	Herpesvirales	Herpesviridae	Roseolovirus	Human_betaherpesvirus_7	site	oral	2.219885707	130	0.230769231	1.24E-09	5.60E-09
Bacteria	Bacteroidetes	Bacteroidia	Bacteroidales	Tannerellaceae	Tannerella	Tannerella_sp_oral_taxon_HOT_286	site	oral	2.337129934	130	0.223076923	4.47E-11	2.41E-10
Bacteria	Proteobacteria	Epsilonproteobacteria	Campylobacterales	Campylobacteraceae	Campylobacter	Campylobacter_concisus	site	oral	2.376360424	130	0.176923077	1.82E-08	7.19E-08
Bacteria	Bacteroidetes	Flavobacteriia	Flavobacteriales	Flavobacteriaceae	Capnocytophaga	Capnocytophaga_leadbetteri	site	oral	2.444338198	130	0.2	3.79E-10	1.88E-09
Bacteria	Actinobacteria	Actinobacteria	Actinomycetales	Actinomycetaceae	Actinomyces	Actinomyces_sp_ICM47	site	oral	2.590244982	130	0.246153846	1.57E-11	9.65E-11
Bacteria	Proteobacteria	Betaproteobacteria	Neisseriales	Neisseriaceae	Neisseria	Neisseria_mucosa	site	oral	2.647720186	130	0.253846154	1.01E-12	7.71E-12
Viruses	Viruses_unclassified	Viruses_unclassified	Caudovirales	Siphoviridae	Siphoviridae_unclassifie	Streptococcus_phage_PH15	site	oral	2.662014805	130	0.253846154	4.95E-13	3.96E-12
Bacteria	Proteobacteria	Gammaproteobacteria	Pasteurellales	Pasteurellaceae	Aggregatibacter	Aggregatibacter_sp_oral_taxon_458	site	oral	2.664056746	130	0.223076923	5.33E-11	2.77E-10
Bacteria	Proteobacteria	Gammaproteobacteria	Pasteurellales	Pasteurellaceae	Haemophilus	Haemophilus_parahaemolyticus	site	oral	2.672194207	130	0.230769231	6.84E-09	2.85E-08
Bacteria	Bacteroidetes	Bacteroidia	Bacteroidales	Porphyromonadaceae	Porphyromonas	Porphyromonas_endodontalis	site	oral	2.730094364	130	0.330769231	1.30E-07	4.42E-07
Bacteria	Bacteroidetes	Bacteroidia	Bacteroidales	Prevotellaceae	Alloprevotella	Alloprevotella_tannerae	site	oral	2.881686524	130	0.253846154	2.49E-11	1.41E-10
Bacteria	Fusobacteria	Fusobacteriia	Fusobacteriales	Fusobacteriaceae	Fusobacterium	Fusobacterium_nucleatum	site	oral	2.914825187	130	0.376923077	0.000429414	0.000712644
Bacteria	Fusobacteria	Fusobacteriia	Fusobacteriales	Fusobacteriaceae	Fusobacterium	Fusobacterium_periodonticum	site	oral	2.925402092	130	0.261538462	2.10E-11	1.26E-10
Bacteria	Fusobacteria	Fusobacteriia	Fusobacteriales	Leptotrichiaceae	Leptotrichia	Leptotrichia_wadei	site	oral	3.194163381	130	0.176923077	1.23E-08	4.91E-08
Bacteria	Firmicutes	Bacilli	Lactobacillales	Streptococcaceae	Streptococcus	Streptococcus_sanguinis	site	oral	3.265197114	130	0.323076923	6.47E-18	1.19E-16
Viruses	Viruses_unclassified	Viruses_unclassified	Caudovirales	Siphoviridae	Siphoviridae_unclassifie	Actinomyces_phage_xhp1	site	oral	3.412052085	130	0.307692308	3.55E-17	5.28E-16
Bacteria	Bacteroidetes	Bacteroidia	Bacteroidales	Prevotellaceae	Prevotella	Prevotella_intermedia	site	oral	3.488195331	130	0.284615385	3.97E-13	3.44E-12
Bacteria	Actinobacteria	Actinobacteria	Actinomycetales	Actinomycetaceae	Actinomyces	Actinomyces_odontolyticus	site	oral	3.522334484	130	0.315384615	6.23E-17	8.09E-16
Bacteria	Firmicutes	Bacilli	Bacillales	Bacillales_unclassified	Gemella	Gemella_sanguinis	site	oral	3.594261319	130	0.346153846	3.56E-17	5.28E-16
Bacteria	Firmicutes	Bacilli	Lactobacillales	Streptococcaceae	Streptococcus	Streptococcus_gordonii	site	oral	3.767469343	130	0.261538462	1.17E-11	7.63E-11
Bacteria	Bacteroidetes	Bacteroidia	Bacteroidales	Porphyromonadaceae	Porphyromonas	Porphyromonas_gingivalis	site	oral	3.795298149	130	0.246153846	2.69E-12	1.91E-11
Bacteria	Actinobacteria	Actinobacteria	Micrococcales	Micrococcaceae	Rothia	Rothia_aeria	site	oral	3.823710975	130	0.284615385	4.42E-16	5.11E-15
Bacteria	Firmicutes	Bacilli	Lactobacillales	Streptococcaceae	Streptococcus	Streptococcus_infantis	site	oral	3.937535519	130	0.353846154	1.02E-17	1.70E-16
Bacteria	Firmicutes	Bacilli	Bacillales	Bacillales_unclassified	Gemella	Gemella_morbillorum	site	oral	4.000725325	130	0.261538462	1.03E-07	3.68E-07
Bacteria	Firmicutes	Bacilli	Lactobacillales	Streptococcaceae	Streptococcus	Streptococcus_parasanguinis	site	oral	4.445988663	130	0.384615385	2.54E-09	1.09E-08
Bacteria	Proteobacteria	Betaproteobacteria	Neisseriales	Neisseriaceae	Neisseria	Neisseria_sicca	site	oral	4.695336857	130	0.376923077	2.44E-25	9.40E-24
Bacteria	Actinobacteria	Actinobacteria	Corynebacteriales	Corynebacteriaceae	Corynebacterium	Corynebacterium_matruchotii	site	oral	4.778034089	130	0.346153846	5.04E-22	1.31E-20
Bacteria	Proteobacteria	Betaproteobacteria	Neisseriales	Neisseriaceae	Neisseria	Neisseria_flavescens	site	oral	5.150808263	130	0.415384615	2.89E-32	3.01E-30
Bacteria	Bacteroidetes	Bacteroidia	Bacteroidales	Prevotellaceae	Prevotella	Prevotella_melaninogenica	site	oral	5.379688804	130	0.476923077	1.07E-15	1.20E-14
Bacteria	Firmicutes	Negativicutes	Veillonellales	Veillonellaceae	Veillonella	Veillonella_parvula	site	oral	5.442444178	130	0.369230769	2.80E-18	5.83E-17
Bacteria	Proteobacteria	Gammaproteobacteria	Pasteurellales	Pasteurellaceae	Haemophilus	Haemophilus_haemolyticus	site	oral	5.614659416	130	0.461538462	5.86E-13	4.57E-12
Bacteria	Bacteroidetes	Bacteroidia	Bacteroidales	Porphyromonadaceae	Porphyromonas	Porphyromonas_somerae	site	oral	5.647661782	130	0.338461538	6.21E-17	8.09E-16
Bacteria	Actinobacteria	Actinobacteria	Actinomycetales	Actinomycetaceae	Actinomyces	Actinomyces_oris	site	oral	5.984783848	130	0.407692308	8.95E-33	1.40E-30
Bacteria	Proteobacteria	Betaproteobacteria	Burkholderiales	Burkholderiaceae	Lautropia	Lautropia_mirabilis	site	oral	6.457833196	130	0.376923077	2.74E-26	1.22E-24
Bacteria	Firmicutes	Bacilli	Lactobacillales	Carnobacteriaceae	Granulicatella	Granulicatella_elegans	site	oral	0.490645578	130	0.584615385	0.127571455	0.218975745
Bacteria	Firmicutes	Bacilli	Lactobacillales	Streptococcaceae	Streptococcus	Streptococcus_oralis	site	oral	6.857891931	130	0.623076923	4.28E-22	1.21E-20
Bacteria	Proteobacteria	Gammaproteobacteria	Pasteurellales	Pasteurellaceae	Haemophilus	Haemophilus_parainfluenzae	site	oral	7.284651957	130	0.538461538	3.06E-29	1.59E-27
Bacteria	Firmicutes	Bacilli	Bacillales	Bacillales_unclassified	Gemella	Gemella_haemolysans	site	oral	7.954501608	130	0.484615385	3.08E-22	9.62E-21
Bacteria	Actinobacteria	Actinobacteria	Micrococcales	Micrococcaceae	Rothia	Rothia_dentocariosa	site	oral	8.50681078	130	0.430769231	4.74E-37	1.48E-34
Bacteria	Actinobacteria	Actinobacteria	Micrococcales	Micrococcaceae	Rothia	Rothia_mucilaginosa	site	oral	9.157013865	130	0.461538462	4.16E-31	2.60E-29
Bacteria	Firmicutes	Bacilli	Lactobacillales	Streptococcaceae	Streptococcus	Streptococcus_mitis	site	oral	10.94198589	130	0.676923077	6.78E-32	5.29E-30
Viruses	Viruses_unclassified	Viruses_unclassified	Caudovirales	Myoviridae	Myoviridae_unclassified	Streptococcus_phage_EJ_1	site	oral	0.255563607	130	0.092307692	0.243123721	0.25284867
Bacteria	Proteobacteria	Gammaproteobacteria	Pasteurellales	Pasteurellaceae	Haemophilus	Haemophilus_pittmaniae	site	oral	0.26251501	130	0.076923077	0.378962608	0.391511039
Bacteria	Firmicutes	Erysipelotrichia	Erysipelotrichales	Erysipelotrichaceae	Solobacterium	Solobacterium_moorei	site	oral	-0.196154747	130	0.061538462	0.385157513	0.396597835
Bacteria	Firmicutes	Tissierellia	Tissierellales	Peptoniphilaceae	Anaerococcus	Anaerococcus_prevotii	site	oral	-0.137733381	130	0.061538462	0.396578627	0.407014907
Bacteria	Firmicutes	Bacilli	Bacillales	Bacillales_unclassified	Gemella	Gemella_bergeri	site	oral	-0.19015031	130	0.069230769	0.43364373	0.443596209
Bacteria	Actinobacteria	Actinobacteria	Corynebacteriales	Corynebacteriaceae	Corynebacterium	Corynebacterium_simulans	site	oral	-0.274790191	130	0.076923077	0.435084006	0.443615065
Viruses	Viruses_unclassified	Viruses_unclassified	Caudovirales	Siphoviridae	Siphoviridae_unclassifie	Streptococcus_phage_MM1	site	oral	-0.14050881	130	0.123076923	0.659564938	0.668130717
Bacteria	Firmicutes	Negativicutes	Veillonellales	Veillonellaceae	Dialister	Dialister_pneumosintes	site	oral	-0.098514035	130	0.1	0.698333863	0.705113804
Bacteria	Actinobacteria	Actinobacteria	Corynebacteriales	Corynebacteriaceae	Corynebacterium	Corynebacterium_jeikeium	site	oral	-0.060964093	130	0.053846154	0.829064097	0.834412898
Bacteria	Proteobacteria	Gammaproteobacteria	Pasteurellales	Pasteurellaceae	Haemophilus	Haemophilus_influenzae	site	oral	-0.053905936	130	0.092307692	0.849858429	0.852591093
Viruses	Viruses_unclassified	Viruses_unclassified	Caudovirales	Siphoviridae	C2virus	Lactococcus_virus_bIL67	site	oral	0.024027071	130	0.053846154	0.899225841	0.899225841

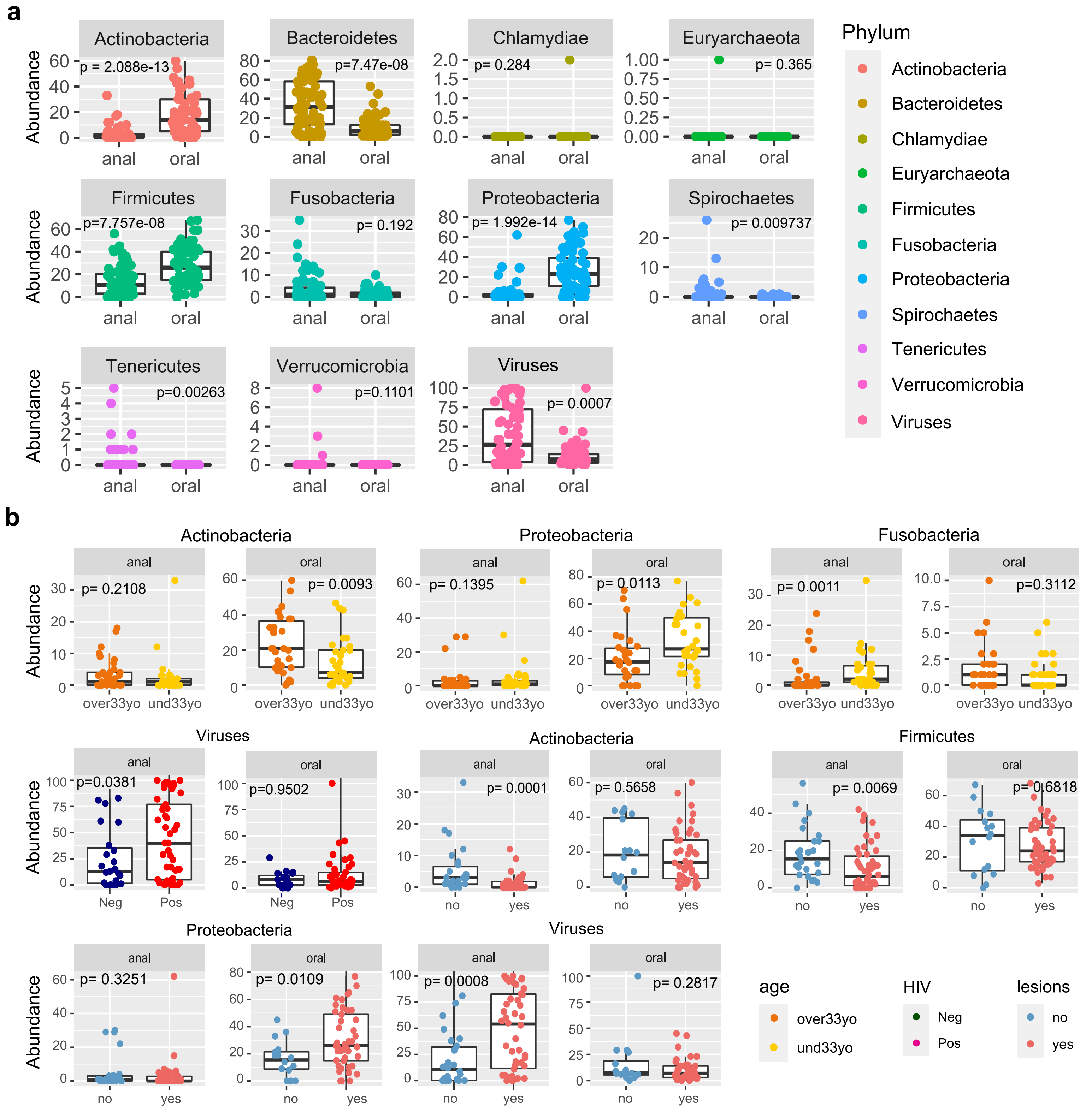

Supplementary Figure 2: **Microbial relative abundance comparisons at Phylum level**. **a**. Oral site versus anal site. **b.** Phyla that showed significant differences among the group of variables age, HIV and presence of lesions (lesions) in the oral and anal sites. The p-values were obtained using the Wilcoxon test.

Supplementary Table 4. Significant associations among taxa and subjects´ metadata

feature	metadata	value	coef	N	prevalence	pval	qval
**VIRUS**							
Human_betaherpesvirus_7	gender	TGW	-0.738650016	130	0.22556391	0.043637061	0.123315247
Streptococcus_phage_phiNJ2	gender	TGW	0.672809405	130	0.076923077	0.046269955	0.128364697
Human_gammaherpesvirus_8	gender	TGW	0.451424607	130	0.076923077	0.021210889	0.093774455
Streptococcus_virus_phiAbc2	gender	TGW	-0.508105847	130	0.076923077	0.049005022	0.124656874
Alphapapillomavirus_7	gender	TGW	0.771673563	130	0.130769231	0.045059508	0.123461041
Alphapapillomavirus_3	gender	TGW	0.928883449	130	0.146153846	0.046850809	0.12452265
Human_betaherpesvirus_7	HIV	Pos	-1.243638186	130	0.22556391	0.001884137	0.01357111
Human_gammaherpesvirus_8	HIV	Pos	0.672845273	130	0.07518797	0.019539752	0.091185511
Alphapapillomavirus_9	HIV	Pos	0.977415055	130	0.157894737	0.035656151	0.13315247
Microbacterium_phage_Min1	HIV	Pos	0.327774255	130	0.07518797	0.046612372	0.138183712
Human_alphaherpesvirus_2	CD4	under500	1.632064437	130	0.090225564	0.014558363	0.098089401
Haemophilus_phage_Aaphi23	CD4	under500	0.786430831	130	0.127819549	0.023956344	0.118372526
Streptococcus_phage_MM1	CD4	under500	1.741701171	130	0.120300752	0.030459311	0.135143451
Alphapapillomavirus_10	lesions	yes	2.330560915	130	0.338345865	2.89E-05	0.000346313
Alphapapillomavirus_3	lesions	yes	1.095435725	130	0.142857143	0.039485448	0.144207724
Alphapapillomavirus_9	lesions	yes	1.009526705	130	0.157894737	0.041818269	0.145669385
Alphapapillomavirus_7	lesions	yes	0.692529826	130	0.127819549	0.044144961	0.146206679
Alphapapillomavirus_8	SW	Yes	0.867244797	133	0.052631579	0.0228316	0.106547468
Actinomyces_phage_xhp1	site	oral	2.563534575	130	0.30075188	4.54481E-17	3.81764E-15
Alphapapillomavirus_10	site	oral	-3.533716016	130	0.338345865	5.73991E-10	1.60718E-08
Alphapapillomavirus_11	site	oral	-1.491995653	130	0.127819549	0.000160031	0.001920376
Alphapapillomavirus_13	site	oral	-0.584818054	130	0.060150376	0.022956281	0.096416382
Alphapapillomavirus_14	site	oral	-1.022334435	130	0.090225564	0.004839015	0.029034088
Alphapapillomavirus_3	site	oral	-1.552486919	130	0.142857143	0.001891077	0.01357111
Alphapapillomavirus_5	site	oral	-0.631652764	130	0.105263158	0.019312695	0.091185511
Alphapapillomavirus_6	site	oral	-0.91611016	130	0.120300752	0.005650112	0.031640629
Alphapapillomavirus_7	site	oral	-1.35156617	130	0.127819549	0.000472761	0.004412435
Alphapapillomavirus_8	site	oral	-0.76737844	130	0.052631579	0.039120907	0.140210233
Alphapapillomavirus_9	site	oral	-1.708754935	130	0.157894737	0.000333728	0.003504142
Haemophilus_phage_Aaphi23	site	oral	1.25789205	130	0.127819549	1.76111E-06	2.46555E-05
Human_alphaherpesvirus_1	site	oral	-0.770737649	130	0.07518797	0.068130888	0.190766485
Human_alphaherpesvirus_2	site	oral	-0.841593556	130	0.090225564	0.030244681	0.115479691
Human_betaherpesvirus_7	site	oral	2.223329442	130	0.22556391	7.2524E-09	1.523E-07
Human_gammaherpesvirus_8	site	oral	0.554865913	130	0.07518797	0.002957126	0.019107581
Human_mastadenovirus_D	site	oral	-0.655987974	130	0.052631579	0.040060067	0.140210233
Microbacterium_phage_Min1	site	oral	0.501826773	130	0.07518797	0.00193873	0.01357111
Streptococcus_phage_Dp_1	site	oral	1.549337171	130	0.15037594	1.32261E-06	2.22199E-05
Streptococcus_phage_PH15	site	oral	4.490767272	130	0.248120301	1.67307E-14	7.02688E-13
Streptococcus_phage_phiNJ2	site	oral	0.876638716	130	0.07518797	0.011017801	0.057843457
Streptococcus_phage_SM1	site	oral	-1.211062072	130	0.180451128	0.026044116	0.104176463
**BACTERIA**							
feature	metadata	value	coef	N	prevalence	pval	qval
Streptococcus_parasanguinis	HIV	Pos	1.621120451	130	0.384615385	0.03640775	0.091784243
Leptotrichia_wadei	HIV	Pos	1.609691064	130	0.176923077	0.005266068	0.015641787
Streptococcus_salivarius	HIV	Pos	1.450075534	130	0.253846154	0.032872479	0.083574099
Campylobacter_concisus	HIV	Pos	1.029979638	130	0.176923077	0.018264945	0.048706519
Veillonella_atypica	HIV	Pos	0.922739835	130	0.2	0.045188605	0.125474776
Streptococcus_anginosus	HIV	Pos	0.895015403	130	0.169230769	0.043498	0.106523357
Streptococcus_cristatus	HIV	Pos	0.873405514	130	0.176923077	0.011557691	0.032104696
Actinomyces_odontolyticus	HIV	Pos	0.868291534	130	0.315384615	0.030962543	0.079391135
Actinomyces_sp_ICM47	HIV	Pos	0.818272422	130	0.246153846	0.034753678	0.087983994
Prevotella_salivae	HIV	Pos	0.75870737	130	0.123076923	0.01227886	0.033950765
Leptotrichia_hofstadii	HIV	Pos	0.74122845	130	0.138461538	0.021673654	0.05703593
Actinomyces_sp_HMSC035G02	HIV	Pos	0.729145377	130	0.123076923	0.005094734	0.015208162
Neisseria_macacae	HIV	Pos	0.646368763	130	0.138461538	0.046004966	0.111752954
Prevotella_sp_oral_taxon_473	HIV	Pos	-0.625499421	130	0.169230769	0.03686642	0.092551682
Alistipes_shahii	HIV	Pos	-0.73357513	130	0.138461538	0.015213138	0.041490377
Firmicutes_bacterium_CAG_83	HIV	Pos	-0.763494437	130	0.261538462	0.038246607	0.095616518
Slackia_isoflavoniconvertens	HIV	Pos	-0.827554975	130	0.238461538	0.009162109	0.025930497
Coprococcus_eutactus	HIV	Pos	-0.867874208	130	0.107692308	0.016298323	0.044049523
Dorea_longicatena	HIV	Pos	-0.898218152	130	0.369230769	0.040104814	0.099433423
Blautia_obeum	HIV	Pos	-0.953701594	130	0.169230769	0.005999283	0.017644949
Catenibacterium_mitsuokai	HIV	Pos	-0.963319136	130	0.369230769	0.014291397	0.039334119
Campylobacter_hominis	HIV	Pos	-1.016082489	130	0.184615385	0.041115107	0.101518782
Holdemanella_biformis	HIV	Pos	-1.022525726	130	0.3	0.003848291	0.011780484
Haemophilus_haemolyticus	HIV	Pos	-1.028809064	130	0.230769231	0.005885783	0.017396402
Odoribacter_splanchnicus	HIV	Pos	-1.111147269	130	0.153846154	0.01704942	0.045668088
Oscillibacter_sp_CAG_241	HIV	Pos	-1.16033995	130	0.284615385	0.030232797	0.078526745
Mitsuokella_multacida	HIV	Pos	-1.227656087	130	0.161538462	0.043674576	0.106523357
Eubacterium_hallii	HIV	Pos	-1.231135438	130	0.215384615	0.030702429	0.079062049
Ruminococcus_torques	HIV	Pos	-1.276820181	130	0.346153846	0.008652473	0.024839636

Supplementary Table 4. (Continuation)

feature	metadata	value	coef	N	prevalence	pval	qval
Corynebacterium_atypicum	HIV	Pos	-1.395217024	130	0.123076923	0.019514011	0.051578885
Haemophilus_quentini	HIV	Pos	-1.473275624	130	0.215384615	0.010605226	0.029824894
Fusicatenibacter_saccharivorans	HIV	Pos	-1.478624914	130	0.3	0.016637684	0.044765068
Bifidobacterium_adolescentis	HIV	Pos	-1.568968722	130	0.207692308	0.010653885	0.029824894
Eubacterium_sp_CAG_180	HIV	Pos	-1.615789571	130	0.215384615	0.003709897	0.011415068
Coprococcus_catus	HIV	Pos	-1.640452216	130	0.207692308	0.002035776	0.006328837
Coprococcus_comes	HIV	Pos	-1.8734579	130	0.353846154	0.010687254	0.029824894
Prevotella_sp_AM42_24	HIV	Pos	-2.078150742	130	0.323076923	0.019158937	0.050864434
Prevotella_buccalis	HIV	Pos	-2.10401006	130	0.284615385	0.00418971	0.012760538
Finegoldia_magna	HIV	Pos	-2.751305978	130	0.438461538	0.004476613	0.013429839
Capnocytophaga_sputigena	HIV	Pos	0.511512331	130	0.161538462	0.052140345	0.125474776
Collinsella_stercoris	HIV	Pos	-0.772321772	130	0.192307692	0.053544496	0.127486895
Prevotella_oulorum	HIV	Pos	0.5568678	130	0.130769231	0.057464851	0.136280279
Capnocytophaga_granulosa	HIV	Pos	0.591938569	130	0.153846154	0.058188784	0.137453819
Veillonella_dispar	HIV	Pos	0.840954943	130	0.184615385	0.058638136	0.137972084
Anaerococcus_vaginalis	HIV	Pos	-0.729062758	130	0.215384615	0.060584935	0.141995941
Anaerostipes_hadrus	HIV	Pos	-0.919967949	130	0.161538462	0.063752429	0.148838355
Alphapapillomavirus_9	HIV	Pos	0.987451597	130	0.161538462	0.069875291	0.162500677
Prevotella_sp_oral_taxon_306	HIV	Pos	0.519955425	130	0.123076923	0.070509849	0.163343279
Dorea_formicigenerans	HIV	Pos	-0.923056786	130	0.361538462	0.070783465	0.163346458
Prevotella_histicola	HIV	Pos	0.688714842	130	0.153846154	0.073142211	0.168143014
Actinomyces_oris	HIV	Pos	0.734794909	130	0.407692308	0.073451349	0.168209196
Veillonella_sp_T11011_6	HIV	Pos	0.550147467	130	0.115384615	0.07496235	0.170368977
Prevotella_pallens	HIV	Pos	0.565566228	130	0.169230769	0.085332158	0.192478552
Peptoniphilus_sp_HMSC062D09	HIV	Pos	-0.609083949	130	0.123076923	0.088570923	0.199035782
Neisseria_cinerea	HIV	Pos	-0.806883567	130	0.146153846	0.089536515	0.200454884
Parabacteroides_distasonis	HIV	Pos	-0.76995079	130	0.230769231	0.090086297	0.200935979
Capnocytophaga_ochracea	HIV	Pos	0.653812905	130	0.138461538	0.096340301	0.21251537
Desulfovibrionaceae_bacterium	HIV	Pos	-0.783935498	130	0.130769231	0.098195639	0.215280634
Peptoniphilus_harei	HIV	Pos	-1.320887637	130	0.223076923	0.09831149	0.215280634
Prevotella_corporis	HIV	Pos	-1.0900052	130	0.230769231	0.099677655	0.216690553
Oscillibacter_sp_57_20	HIV	Pos	-0.87924236	130	0.338461538	0.104010289	0.224482639
Campylobacter_rectus	HIV	Pos	-0.498119864	130	0.138461538	0.10450948	0.224669279
Faecalibacterium_prausnitzii	HIV	Pos	-0.700227396	130	0.438461538	0.105043216	0.224669279
Capnocytophaga_gingivalis	HIV	Pos	0.581793638	130	0.192307692	0.105220112	0.224669279
Roseburia_inulinivorans	HIV	Pos	-0.986697131	130	0.330769231	0.113813214	0.242155775
Gemella_morbillorum	gender	TGW	2.757367196	130	0.261538462	0.00025539	0.000896106
Granulicatella_elegans	gender	TGW	2.864225854	130	0.584615385	0.000790109	0.00253511
Mageeibacillus_indolicus	gender	TGW	0.996608099	130	0.107692308	0.00435896	0.013142593
Gemella_asaccharolytica	gender	TGW	1.293994349	130	0.161538462	0.006960172	0.020272346
Gardnerella_vaginalis	gender	TGW	0.829746375	130	0.130769231	0.009095525	0.025864052
Fusobacterium_nucleatum	gender	TGW	2.116976952	130	0.376923077	0.014506029	0.039742546
Haemophilus_parainfluenzae	gender	TGW	1.295583636	130	0.538461538	0.016121713	0.043769357
Streptococcus_mitis	gender	TGW	1.688515469	130	0.676923077	0.025282476	0.066242296
Prevotella_sp_F0091	gender	TGW	0.630569292	130	0.123076923	0.029572082	0.077144563
Haemophilus_sputorum	gender	TGW	0.794745998	130	0.146153846	0.030406651	0.07863789
Roseburia_intestinalis	gender	TGW	0.789760731	130	0.107692308	0.03160049	0.080682103
Prevotella_melaninogenica	gender	TGW	1.312581455	130	0.476923077	0.039636847	0.098680948
Prevotella_sp_oral_taxon_299	gender	TGW	0.950658788	130	0.115384615	0.041389466	0.101777375
Streptococcus_oralis	gender	TGW	1.17614621	130	0.623076923	0.045228116	0.125474776
Haemophilus_haemolyticus	gender	TGW	1.459218737	130	0.461538462	0.045294869	0.126570567
Anaerobiospirillum_thomasii	gender	TGW	0.484680373	130	0.107692308	0.073822066	0.16841536
Actinomyces_sp_oral_taxon_414	gender	TGW	0.63232282	130	0.123076923	0.085297985	0.192478552
Slackia_isoflavoniconvertens	gender	TGW	-0.514407981	130	0.238461538	0.092164283	0.204809517
Ruminococcus_bromii	gender	TGW	-0.930834254	130	0.184615385	0.093483632	0.206974831
Eubacterium_siraeum	gender	TGW	-0.985429767	130	0.184615385	0.099663176	0.216690553
Roseburia_hominis	gender	TGW	0.619314853	130	0.123076923	0.100212195	0.217066126
Rothia_dentocariosa	site	oral	8.469372045	130	0.430769231	1.53525E-36	9.2115E-34
Actinomyces_oris	site	oral	5.989376939	130	0.407692308	1.41597E-32	4.2479E-30
Streptococcus_mitis	site	oral	11.1078276	130	0.676923077	7.25606E-32	1.45121E-29
Rothia_mucilaginosa	site	oral	9.125380362	130	0.461538462	1.37653E-30	2.06479E-28
Neisseria_flavescens	site	oral	4.633646602	130	0.415384615	3.84421E-30	4.61305E-28
Haemophilus_parainfluenzae	site	oral	7.428857924	130	0.538461538	1.38782E-29	1.38782E-27
Lautropia_mirabilis	site	oral	6.434528291	130	0.376923077	9.67218E-26	8.29044E-24
Prevotella_copri	site	oral	-9.161086678	130	0.461538462	2.03261E-25	1.52446E-23
Neisseria_sicca	site	oral	4.671969768	130	0.376923077	1.07198E-24	7.14653E-23
Streptococcus_oralis	site	oral	6.746128437	130	0.623076923	1.75504E-22	1.05303E-20
Gemella_haemolysans	site	oral	8.078496093	130	0.484615385	4.34574E-22	2.3704E-20
Corynebacterium_matruchotii	site	oral	4.827009798	130	0.346153846	6.334E-22	3.167E-20
Faecalibacterium_prausnitzii	site	oral	-4.573915754	130	0.438461538	7.31138E-22	3.37448E-20
Collinsella_aerofaciens	site	oral	-3.432601279	130	0.446153846	2.30019E-18	9.56422E-17
Finegoldia_magna	site	oral	-8.906738869	130	0.438461538	2.39106E-18	9.56422E-17

Supplementary Table 4. (Continuation)

feature	metadata	value	coef	N	prevalence	pval	qval
Veillonella_parvula	site	oral	5.466869998	130	0.369230769	5.99789E-18	2.24921E-16
Streptococcus_sanguinis	site	oral	3.289674365	130	0.323076923	1.24E-17	4.37648E-16
Eubacterium_rectale	site	oral	-5.891528093	130	0.361538462	2.82851E-17	8.93214E-16
Prevotella_bivia	site	oral	-7.450184497	130	0.438461538	2.78074E-17	8.93214E-16
Coprococcus_comes	site	oral	-6.452836573	130	0.353846154	3.76698E-17	1.13009E-15
Streptococcus_infantis	site	oral	3.858425826	130	0.353846154	6.14405E-17	1.75544E-15
Actinomyces_phage_xhp1	site	oral	3.432125202	130	0.307692308	6.44266E-17	1.75709E-15
Gemella_sanguinis	site	oral	3.51400438	130	0.346153846	8.90723E-17	2.32363E-15
Dorea_longicatena	site	oral	-3.790869325	130	0.369230769	1.08392E-16	2.7098E-15
Actinomyces_odontolyticus	site	oral	3.47483675	130	0.315384615	1.23808E-16	2.9714E-15
Porphyromonas_somerae	site	oral	5.693570542	130	0.338461538	1.89253E-16	4.36738E-15
Prevotella_melaninogenica	site	oral	5.514159977	130	0.476923077	6.35649E-16	1.41255E-14
Rothia_aeria	site	oral	3.81575746	130	0.284615385	1.04782E-15	2.24533E-14
Dorea_formicigenerans	site	oral	-4.180831558	130	0.361538462	2.33315E-15	4.82721E-14
Catenibacterium_mitsuokai	site	oral	-3.172146727	130	0.369230769	3.66548E-15	7.33096E-14
Oscillibacter_sp_57_20	site	oral	-4.350290345	130	0.338461538	5.86863E-15	1.13586E-13
Roseburia_inulinivorans	site	oral	-4.717862722	130	0.330769231	1.12232E-13	2.10436E-12
Prevotella_sp_CAG_520	site	oral	-6.462603295	130	0.346153846	1.4839E-13	2.698E-12
Prevotella_intermedia	site	oral	3.557203672	130	0.284615385	2.40929E-13	4.2517E-12
Haemophilus_haemolyticus	site	oral	5.762652113	130	0.461538462	2.51951E-13	4.31915E-12
Blautia_wexlerae	site	oral	-4.983332879	130	0.323076923	3.39593E-13	5.54829E-12
Parvimonas_sp_KA00067	site	oral	-4.814351845	130	0.323076923	3.42144E-13	5.54829E-12
Prevotella_disiens	site	oral	-6.945819647	130	0.353846154	4.96139E-13	7.83378E-12
Fusicatenibacter_saccharivorans	site	oral	-4.466120431	130	0.3	6.10447E-13	9.39149E-12
Neisseria_mucosa	site	oral	2.7027516	130	0.253846154	7.04995E-13	1.0317E-11
Prevotella_stercorea	site	oral	-3.832742577	130	0.346153846	7.00554E-13	1.0317E-11
Streptococcus_phage_PH15	site	oral	2.650947578	130	0.253846154	1.24016E-12	1.77166E-11
Porphyromonas_gingivalis	site	oral	3.871254485	130	0.246153846	2.45777E-12	3.42944E-11
Peptostreptococcus_anaerobius	site	oral	-4.10428888	130	0.369230769	4.3578E-12	5.94245E-11
Ruminococcus_torques	site	oral	-3.332439001	130	0.346153846	5.36437E-12	7.15249E-11
Fusobacterium_gonidiaformans	site	oral	-3.70313752	130	0.338461538	5.8982E-12	7.52961E-11
Prevotella_sp_AM42_24	site	oral	-6.083454633	130	0.323076923	5.83439E-12	7.52961E-11
Prevotella_sp_885	site	oral	-4.751202505	130	0.346153846	9.07471E-12	1.13434E-10
Streptococcus_gordonii	site	oral	3.825407307	130	0.261538462	9.66675E-12	1.18368E-10
Holdemanella_biformis	site	oral	-2.355760657	130	0.3	1.49805E-11	1.79767E-10
Bacteroides_vulgatus	site	oral	-4.79171288	130	0.284615385	1.73676E-11	2.00395E-10
Prevotella_sp_CAG_279	site	oral	-3.344389817	130	0.3	1.71879E-11	2.00395E-10
Aggregatibacter_sp_oral_taxon_458	site	oral	2.738221997	130	0.223076923	2.55678E-11	2.89446E-10
Alloprevotella_tannerae	site	oral	2.8030494	130	0.253846154	2.74783E-11	3.05314E-10
Fusobacterium_periodonticum	site	oral	2.967319291	130	0.261538462	3.50177E-11	3.82011E-10
Actinomyces_sp_ICM47	site	oral	2.539161001	130	0.246153846	3.76734E-11	4.03643E-10
Tannerella_sp_oral_taxon_HOT_286	site	oral	2.355572362	130	0.223076923	5.70236E-11	6.00248E-10
Prevotella_timonensis	site	oral	-3.133545231	130	0.323076923	6.29459E-11	6.51164E-10
Oscillibacter_sp_CAG_241	site	oral	-3.420860654	130	0.284615385	9.43627E-11	9.59621E-10
Phascolarctobacterium_succinatutens	site	oral	-6.152563426	130	0.3	1.19034E-10	1.19034E-09
Escherichia_coli	site	oral	-3.085634368	130	0.3	1.25532E-10	1.23474E-09
Prevotella_nigrescens	site	oral	2.133191653	130	0.238461538	1.29934E-10	1.25743E-09
Capnocytophaga_leadbetteri	site	oral	2.496844881	130	0.2	2.30424E-10	2.19451E-09
Alphapapillomavirus_10	site	oral	-4.053410831	130	0.346153846	4.24015E-10	3.97514E-09
Prevotella_denticola	site	oral	2.148380805	130	0.207692308	6.35036E-10	5.86187E-09
Aggregatibacter_segnis	site	oral	2.137628025	130	0.2	8.07346E-10	7.33951E-09
Actinomyces_turicensis	site	oral	-3.762361104	130	0.269230769	1.03457E-09	9.16758E-09
Capnocytophaga_gingivalis	site	oral	2.150452655	130	0.192307692	1.03899E-09	9.16758E-09
Roseburia_faecis	site	oral	-3.617823676	130	0.269230769	1.14234E-09	9.79147E-09
Human_betaherpesvirus_7	site	oral	2.209877268	130	0.230769231	1.12691E-09	9.79147E-09
Prevotella_buccalis	site	oral	-4.2180956	130	0.284615385	2.86399E-09	2.38666E-08
Streptococcus_parasanguinis	site	oral	4.482832719	130	0.384615385	2.83297E-09	2.38666E-08
Streptococcus_cristatus	site	oral	1.97497686	130	0.176923077	3.78572E-09	3.11155E-08
Megasphaera_elsdenii	site	oral	-3.349609714	130	0.269230769	3.8677E-09	3.13597E-08
Actinomyces_naeslundii	site	oral	1.574224421	130	0.207692308	5.91356E-09	4.73084E-08
Leptotrichia_wadei	site	oral	3.227746752	130	0.176923077	6.52662E-09	5.1526E-08
Gemella_morbillorum	site	oral	4.285511096	130	0.261538462	7.47732E-09	5.82648E-08
Firmicutes_bacterium_CAG_83	site	oral	-2.055507446	130	0.261538462	8.76326E-09	6.74097E-08
Haemophilus_parahaemolyticus	site	oral	2.667359266	130	0.230769231	9.93577E-09	7.54616E-08
Campylobacter_concisus	site	oral	2.410774409	130	0.176923077	1.09067E-08	8.18002E-08
Slackia_isoflavoniconvertens	site	oral	-1.742651708	130	0.238461538	1.238E-08	9.17038E-08
Neisseria_elongata	site	oral	1.934998722	130	0.184615385	3.10098E-08	2.26901E-07
Gemmiger_formicilis	site	oral	-2.203517679	130	0.238461538	3.81837E-08	2.76027E-07
Bifidobacterium_adolescentis	site	oral	-3.205398977	130	0.207692308	5.26391E-08	3.75994E-07
Prevotella_oris	site	oral	1.576508658	130	0.176923077	6.17276E-08	4.35724E-07
Coprococcus_catus	site	oral	-2.73898871	130	0.207692308	6.36237E-08	4.43886E-07
Mitsuokella_jalaludinii	site	oral	-2.867897939	130	0.215384615	8.05771E-08	5.55704E-07

Supplementary Table 4. (Continuation)

feature	metadata	value	coef	N	prevalence	pval	qval
Eubacterium_hallii	site	oral	-2.924919786	130	0.215384615	8.96104E-08	6.01352E-07
Porphyromonas_asaccharolytica	site	oral	-2.601477643	130	0.223076923	8.87434E-08	6.01352E-07
Prevotella_corporis	site	oral	-3.411038158	130	0.230769231	9.02029E-08	6.01352E-07
Haemophilus_sputorum	site	oral	1.930906736	130	0.146153846	1.07606E-07	7.09488E-07
Prevotella_sp_oral_taxon_473	site	oral	1.516453294	130	0.169230769	1.32857E-07	8.66461E-07
Parabacteroides_distasonis	site	oral	-2.291534716	130	0.230769231	1.55131E-07	1.00084E-06
Porphyromonas_endodontalis	site	oral	2.783618316	130	0.330769231	1.5945E-07	1.01776E-06
Neisseria_sp_oral_taxon_014	site	oral	1.480389362	130	0.176923077	1.62762E-07	1.02797E-06
Flavonifractor_plautii	site	oral	-2.41172806	130	0.215384615	1.76555E-07	1.10347E-06
Bacteroides_sp_CAG_530	site	oral	-2.402007524	130	0.207692308	2.16615E-07	1.33782E-06
Capnocytophaga_sputigena	site	oral	1.308583838	130	0.161538462	2.18511E-07	1.33782E-06
Actinobaculum_sp_oral_taxon_183	site	oral	1.694980691	130	0.169230769	2.39959E-07	1.45429E-06
Peptostreptococcus_sp_MV1	site	oral	-2.456364943	130	0.215384615	2.59739E-07	1.55843E-06
Anaerococcus_vaginalis	site	oral	-1.898593467	130	0.215384615	3.32832E-07	1.97722E-06
Eubacterium_sp_CAG_180	site	oral	-2.67986383	130	0.215384615	3.81769E-07	2.2457E-06
Capnocytophaga_granulosa	site	oral	1.510517026	130	0.153846154	4.31857E-07	2.5038E-06
Ruminococcus_bromii	site	oral	-2.771084827	130	0.184615385	4.33991E-07	2.5038E-06
Porphyromonas_uenonis	site	oral	-3.107588797	130	0.207692308	5.25981E-07	3.0056E-06
Prevotella_histicola	site	oral	1.825349731	130	0.153846154	6.67184E-07	3.77651E-06
Capnocytophaga_ochracea	site	oral	1.854202618	130	0.138461538	8.02039E-07	4.49742E-06
Peptoniphilus_lacrimalis	site	oral	-2.614067237	130	0.192307692	9.27801E-07	5.15445E-06
Collinsella_stercoris	site	oral	-1.869010239	130	0.192307692	9.52452E-07	5.24285E-06
Firmicutes_bacterium_CAG_170	site	oral	-2.854730249	130	0.192307692	1.00486E-06	5.43165E-06
Leptotrichia_hofstadii	site	oral	1.298033118	130	0.138461538	9.99398E-07	5.43165E-06
Eubacterium_siraeum	site	oral	-2.877481015	130	0.184615385	1.05339E-06	5.64315E-06
Prevotella_pallens	site	oral	1.513031151	130	0.169230769	1.37527E-06	7.30232E-06
Neisseria_cinerea	site	oral	2.182085742	130	0.146153846	1.45738E-06	7.67045E-06
Dialister_micraerophilus	site	oral	-2.494441432	130	0.192307692	1.51948E-06	7.92774E-06
Firmicutes_bacterium_CAG_110	site	oral	-1.900957919	130	0.176923077	1.60677E-06	8.31088E-06
Porphyromonas_sp_oral_taxon_278	site	oral	1.745836692	130	0.138461538	1.6653E-06	8.54E-06
Actinomyces_massiliensis	site	oral	1.3208266	130	0.130769231	2.23581E-06	1.13685E-05
Campylobacter_rectus	site	oral	1.375548649	130	0.138461538	2.47325E-06	1.24702E-05
Campylobacter_ureolyticus	site	oral	-2.247045231	130	0.192307692	2.68851E-06	1.34425E-05
Eubacterium_ramulus	site	oral	-1.694363932	130	0.192307692	2.95189E-06	1.46375E-05
Haemophilus_phage_Aaphi23	site	oral	1.180258753	130	0.130769231	3.23337E-06	1.59018E-05
Eikenella_corrodens	site	oral	1.246490131	130	0.130769231	3.4956E-06	1.70517E-05
Actinomyces_sp_oral_taxon_414	site	oral	1.63323306	130	0.123076923	5.53012E-06	2.67587E-05
Mitsuokella_multacida	site	oral	-2.611292954	130	0.161538462	5.74378E-06	2.75702E-05
Brachyspira_sp_CAG_700	site	oral	-2.392662746	130	0.2	5.86504E-06	2.79288E-05
Prevotella_sp_CAG_5226	site	oral	-3.240756754	130	0.161538462	7.02108E-06	3.31705E-05
Actinomyces_graevenitzii	site	oral	1.79366322	130	0.130769231	7.6764E-06	3.54499E-05
Blautia_obeum	site	oral	-1.458325988	130	0.169230769	7.57998E-06	3.54499E-05
Campylobacter_hominis	site	oral	-2.102001281	130	0.184615385	7.73989E-06	3.54499E-05
Desulfovibrio_piger	site	oral	-2.128417969	130	0.169230769	7.68485E-06	3.54499E-05
Prevotella_sp_oral_taxon_299	site	oral	2.016249172	130	0.115384615	8.96338E-06	4.07426E-05
Fusobacterium_mortiferum	site	oral	-1.90645217	130	0.192307692	9.12734E-06	4.1176E-05
Actinomyces_johnsonii	site	oral	1.728261232	130	0.123076923	9.94866E-06	4.45463E-05
Veillonella_dispar	site	oral	1.855882241	130	0.184615385	1.00401E-05	4.46229E-05
Prevotella_sp_F0091	site	oral	1.242568251	130	0.123076923	1.03915E-05	4.58449E-05
Anaerostipes_hadrus	site	oral	-2.057667732	130	0.161538462	1.13729E-05	4.98082E-05
Kingella_oralis	site	oral	0.831049861	130	0.130769231	1.21107E-05	5.26552E-05
Bacteroides_uniformis	site	oral	-1.987356739	130	0.176923077	1.22884E-05	5.30434E-05
Porphyromonas_sp_HMSC065F10	site	oral	-2.302045036	130	0.169230769	1.33552E-05	5.72366E-05
Gemella_asaccharolytica	site	oral	-2.010727853	130	0.161538462	1.43036E-05	6.08662E-05
Prevotella_salivae	site	oral	1.225811105	130	0.123076923	1.61779E-05	6.83574E-05
Streptococcus_phage_Dp_1	site	oral	1.330537229	130	0.153846154	1.68748E-05	7.08033E-05
Odoribacter_splanchnicus	site	oral	-1.873394483	130	0.153846154	1.83412E-05	7.64218E-05
Prevotella_oulorum	site	oral	1.183002395	130	0.130769231	1.85696E-05	7.68396E-05
Campylobacter_showae	site	oral	1.611787269	130	0.115384615	2.1642E-05	8.89397E-05
Corynebacterium_durum	site	oral	1.399633787	130	0.123076923	2.25023E-05	9.18462E-05
Tannerella_forsythia	site	oral	1.22290615	130	0.115384615	2.32409E-05	9.422E-05
Actinomyces_sp_oral_taxon_448	site	oral	1.036909058	130	0.138461538	2.40518E-05	9.6853E-05
Bacteroides_fragilis	site	oral	-2.351461343	130	0.176923077	2.62287E-05	0.00010422
Prevotella_sp_oral_taxon_306	site	oral	1.138781887	130	0.123076923	2.61219E-05	0.00010422
Prevotella_sp_CAG_1092	site	oral	-1.358385955	130	0.169230769	3.03633E-05	0.000119855
Actinomyces_sp_HMSC035G02	site	oral	0.8424843	130	0.123076923	4.31362E-05	0.000169162
Peptoniphilus_harei	site	oral	-3.064360595	130	0.223076923	4.59314E-05	0.000178953
Methanobrevibacter_smithii	site	oral	-1.335019233	130	0.146153846	4.79958E-05	0.00018579
Agathobaculum_butyriciproducens	site	oral	-1.944820761	130	0.146153846	5.1596E-05	0.000198446
Treponema_denticola	site	oral	0.936265368	130	0.107692308	5.80343E-05	0.000221787
Criibacterium_bergeronii	site	oral	-1.837243991	130	0.146153846	6.10637E-05	0.000231888
Prevotella_jejuni	site	oral	1.065137419	130	0.153846154	7.5222E-05	0.000283856

Supplementary Table 4. (Continuation)

feature	metadata	value	coef	N	prevalence	pval	qval
Desulfovibrionaceae_bacterium	site	oral	-1.730364937	130	0.130769231	0.000100139	0.000375523
Alistipes_shahii	site	oral	-1.091766226	130	0.138461538	0.000106676	0.000397549
Streptococcus_pseudopneumoniae	site	oral	1.321168812	130	0.192307692	0.000119295	0.000441834
Haemophilus_paraphrohaemolyticus	site	oral	1.330822642	130	0.123076923	0.000131829	0.000485259
Fusobacterium_equinum	site	oral	-1.804233645	130	0.138461538	0.000137427	0.000502782
Haemophilus_quentini	site	oral	2.024092838	130	0.215384615	0.0001592	0.000578908
Fusobacterium_nucleatum	site	oral	3.131375952	130	0.376923077	0.000165665	0.00059879
Veillonella_atypica	site	oral	1.666579074	130	0.2	0.000171654	0.00061672
Haemophilus_sp_HMSC71H05	site	oral	1.585491529	130	0.176923077	0.000184911	0.000660398
Butyricimonas_virosa	site	oral	-1.254066422	130	0.123076923	0.000205022	0.00072789
Neisseria_macacae	site	oral	0.773844348	130	0.138461538	0.000210509	0.000742973
Alphapapillomavirus_7	site	oral	-1.426280916	130	0.130769231	0.00026834	0.00093607
Parabacteroides_merdae	site	oral	-1.001333322	130	0.130769231	0.000289323	0.001003431
Roseburia_hominis	site	oral	-1.314901602	130	0.123076923	0.000294688	0.001011587
Alphapapillomavirus_9	site	oral	-1.840600164	130	0.161538462	0.000295046	0.001011587
Coprococcus_eutactus	site	oral	-1.207186193	130	0.107692308	0.000320759	0.001093495
Alphapapillomavirus_11	site	oral	-1.000994809	130	0.130769231	0.000335051	0.001135765
Ruthenibacterium_lactatiformans	site	oral	-1.158324873	130	0.123076923	0.000343731	0.001158644
Lachnospira_pectinoschiza	site	oral	-0.975945408	130	0.115384615	0.000421706	0.001413541
Corynebacterium_atypicum	site	oral	-1.952125789	130	0.123076923	0.000430855	0.001436182
Gardnerella_vaginalis	site	oral	-1.060428416	130	0.130769231	0.000479822	0.00159057
Streptococcus_sp_A12	site	oral	0.924586392	130	0.107692308	0.000569721	0.001878202
Sneathia_amnii	site	oral	-1.849492145	130	0.115384615	0.000655929	0.002150587
Brachyspira_pilosicoli	site	oral	-1.157966779	130	0.107692308	0.000661089	0.002155725
Veillonella_infantium	site	oral	0.813143901	130	0.146153846	0.000696026	0.002257383
Bacteroides_coagulans	site	oral	-1.16489421	130	0.107692308	0.000752378	0.002427027
Eubacterium_eligens	site	oral	-0.926177047	130	0.115384615	0.000862564	0.002752865
Anaerobiospirillum_thomasii	site	oral	-0.860164094	130	0.107692308	0.000939838	0.002983613
Alphapapillomavirus_3	site	oral	-1.286146405	130	0.146153846	0.001293747	0.004085516
Roseburia_intestinalis	site	oral	-1.125650335	130	0.107692308	0.001340033	0.004209529
Veillonella_sp_T11011_6	site	oral	0.897619291	130	0.115384615	0.001736522	0.005426632
Mageeibacillus_indolicus	site	oral	-0.971800655	130	0.107692308	0.0032829	0.010153298
Alphapapillomavirus_6	site	oral	-0.824691973	130	0.123076923	0.004245926	0.012866442
Alphapapillomavirus_5	site	oral	-1.113305939	130	0.107692308	0.006814012	0.019943451
Peptoniphilus_sp_HMSC062D09	site	oral	-0.882992574	130	0.123076923	0.00749778	0.021732696
Bifidobacterium_longum	site	oral	-0.753422323	130	0.107692308	0.007762712	0.022392437
Streptococcus_phage_SM1	site	oral	-1.583263097	130	0.184615385	0.008857177	0.025306221
Prevotella_bivia	lesions	yes	1.833583547	130	0.428571429	0.029982439	0.079953172
Fusobacterium_gonidiaformans	lesions	yes	0.976008287	130	0.255639098	0.027193309	0.083982544
Mageeibacillus_indolicus	lesions	yes	-0.824050231	130	0.105263158	0.025823448	0.070108908
Collinsella_aerofaciens	lesions	yes	-1.005978035	130	0.436090226	0.006362978	0.018714641
Ruminococcus_torques	lesions	yes	-1.024043125	130	0.338345865	0.034659476	0.087376829
Eubacterium_sp_CAG_180	lesions	yes	-1.117771614	130	0.210526316	0.044153857	0.106394837
Desulfovibrionaceae_bacterium	lesions	yes	-1.269168733	130	0.127819549	0.007397106	0.02154497
Corynebacterium_atypicum	lesions	yes	-1.802668645	130	0.120300752	0.002461256	0.007612131
Finegoldia_magna	lesions	yes	-1.875436875	130	0.428571429	0.051842954	0.123435604
Aggregatibacter_segnis	lesions	yes	0.669612097	130	0.195488722	0.060846587	0.142054288
Neisseria_macacae	lesions	yes	-0.413410366	130	0.135338346	0.066407368	0.151459205
Fusobacterium_equinum	lesions	yes	0.912300666	130	0.135338346	0.072904962	0.165067839
Capnocytophaga_leadbetteri	lesions	yes	0.720397478	130	0.195488722	0.074575901	0.168216317
Haemophilus_parahaemolyticus	lesions	yes	0.828204623	130	0.22556391	0.08610201	0.190631755
Peptoniphilus_harei	lesions	yes	-1.372323732	130	0.218045113	0.089135074	0.195901262
Actinomyces_sp_ICM47	lesions	yes	-0.636744019	130	0.240601504	0.102637122	0.222318677
Tannerella_forsythia	lesions	yes	0.490431834	130	0.112781955	0.112475062	0.240160276
Granulicatella_elegans	SW	Yes	2.64198752	130	0.571428571	0.001286959	0.004085583
Gemella_morbillorum	SW	Yes	2.303445136	130	0.255639098	0.001720258	0.005403952
Mageeibacillus_indolicus	SW	Yes	0.960784998	130	0.105263158	0.005116854	0.015427699
Prevotella_sp_885	SW	Yes	1.612902775	130	0.338345865	0.013250758	0.037151657
Gardnerella_vaginalis	SW	Yes	0.772497287	130	0.127819549	0.013366945	0.037303104
Fusobacterium_nucleatum	SW	Yes	1.962093672	130	0.368421053	0.019791443	0.053976662
Streptococcus_cristatus	SW	Yes	0.732873559	130	0.172932331	0.021033691	0.056592891
Rothia_mucilaginosa	SW	Yes	1.418282761	130	0.45112782	0.022357721	0.059356782
Roseburia_hominis	SW	Yes	0.776383157	130	0.120300752	0.035039847	0.089084356
Haemophilus_parainfluenzae	SW	Yes	1.013599194	130	0.526315789	0.041911727	0.132074946
Haemophilus_haemolyticus	SW	Yes	1.359069049	130	0.45112782	0.043341919	0.148457624
Gemella_asaccharolytica	SW	Yes	0.845439462	130	0.157894737	0.044287948	0.13693921
Haemophilus_quentini	SW	Yes	1.04242142	130	0.210526316	0.044554426	0.111386064
Veillonella_dispar	SW	Yes	0.818760524	130	0.180451128	0.048182129	0.117517387
Veillonella_parvula	SW	Yes	1.059192407	130	0.360902256	0.053726092	0.128428906
Prevotella_melaninogenica	SW	Yes	1.190174237	130	0.466165414	0.055402826	0.131390101
Neisseria_mucosa	SW	Yes	0.616371546	130	0.248120301	0.066290181	0.154763068
Gemella_sanguinis	SW	Yes	0.68767757	130	0.338345865	0.067186184	0.155769347

Supplementary Table 4. (Continuation)

feature	metadata	value	coef	N	prevalence	pval	qval
Haemophilus_sputorum	SW	Yes	0.630759661	130	0.142857143	0.073039399	0.165998635
Prevotella_sp_F0091	SW	Yes	0.491935578	130	0.120300752	0.082974408	0.182361337
Roseburia_faecis	SW	Yes	0.981234953	130	0.263157895	0.084792329	0.185676633
Streptococcus_parasanguinis	SW	Yes	1.22092796	130	0.37593985	0.085545478	0.186644679
Slackia_isoflavoniconvertens	SW	Yes	-0.510722495	130	0.233082707	0.087628054	0.189808059
Aggregatibacter_sp_oral_taxon_458	SW	Yes	0.622475899	130	0.218045113	0.100332293	0.212718642
Roseburia_intestinalis	SW	Yes	0.579013014	130	0.105263158	0.109739214	0.231029924
Streptococcus_mitis	SW	Yes	1.164778955	130	0.661654135	0.116828949	0.243393644
Prevotella_oris	SW	Yes	0.432003631	130	0.172932331	0.120571895	0.248601846
Prevotella_bivia	age_code	und33yo	3.088531384	130	0.438461538	0.000135458	0.00066077
Bacteroides_fragilis	age_code	und33yo	2.07401239	130	0.176923077	0.000314448	0.001398773
Peptostreptococcus_anaerobius	age_code	und33yo	1.839710963	130	0.369230769	0.001423202	0.005868876
Gemella_asaccharolytica	age_code	und33yo	1.471479624	130	0.161538462	0.00212891	0.008601657
Neisseria_sicca	age_code	und33yo	1.124157924	130	0.376923077	0.004291791	0.01691346
Fusobacterium_gonidiaformans	age_code	und33yo	1.461429913	130	0.338461538	0.005532313	0.021589513
Fusobacterium_mortiferum	age_code	und33yo	1.2040905	130	0.192307692	0.006848313	0.026595389
Dialister_micraerophilus	age_code	und33yo	1.366579553	130	0.192307692	0.010594484	0.039421334
Neisseria_cinerea	age_code	und33yo	1.177991545	130	0.146153846	0.011655729	0.042970429
Prevotella_timonensis	age_code	und33yo	1.172297306	130	0.323076923	0.013646506	0.049850249
Alphapapillomavirus_11	age_code	und33yo	0.678902271	130	0.130769231	0.021066492	0.074243144
Campylobacter_ureolyticus	age_code	und33yo	1.142073817	130	0.192307692	0.02124371	0.074539332
Prevotella_disiens	age_code	und33yo	2.08808074	130	0.353846154	0.025773994	0.088116218
Sneathia_amnii	age_code	und33yo	1.27831461	130	0.115384615	0.025997764	0.088128014
Brachyspira_pilosicoli	age_code	und33yo	0.794181359	130	0.107692308	0.027295413	0.091667638
Ruthenibacterium_lactatiformans	age_code	und33yo	0.730568224	130	0.123076923	0.032529033	0.106652568
Granulicatella_elegans	age_code	und33yo	1.806808293	130	0.584615385	0.034410793	0.111905019
Actinobaculum_sp_oral_taxon_183	age_code	und33yo	-0.711867727	130	0.169230769	0.034757861	0.112576069
Neisseria_elongata	age_code	und33yo	0.737396432	130	0.184615385	0.038576051	0.122951557
Capnocytophaga_sputigena	age_code	und33yo	0.528247755	130	0.161538462	0.041794356	0.130607363
Prevotella_disiens	CD4	under500	2.123185404	130	0.345864662	0.045845176	0.155426989
Bacteroides_fragilis	CD4	under500	2.051336759	130	0.172932331	0.001654392	0.007245511
Peptostreptococcus_anaerobius	CD4	under500	1.611089879	130	0.360902256	0.013247011	0.043432824
Campylobacter_ureolyticus	CD4	under500	1.265484394	130	0.187969925	0.044673957	0.132694921
Desulfovibrionaceae_bacterium	CD4	under500	1.279146351	130	0.127819549	0.008878268	0.030439777
Veillonella_dispar	CD4	under500	1.253524771	130	0.180451128	0.028553722	0.089230382
Methanobrevibacter_smithii	CD4	under500	0.955406119	130	0.142857143	0.025716877	0.081211192
Anaerostipes_hadrus	CD4	under500	-1.292216274	130	0.157894737	0.031700942	0.096063462
Veillonella_sp_T11011_6	CD4	under500	0.650645608	130	0.112781955	0.056806958	0.166264266
Dialister_micraerophilus	CD4	under500	1.131368633	130	0.187969925	0.057167449	0.166507132
Parabacteroides_distasonis	CD4	under500	1.015071605	130	0.22556391	0.058920508	0.170784081
Streptococcus_phage_MM1	CD4	under500	0.872716603	130	0.120300752	0.059392211	0.171323686
Corynebacterium_durum	CD4	under500	0.874676512	130	0.120300752	0.063799835	0.181421332
Eubacterium_siraeum	CD4	under500	1.355159604	130	0.180451128	0.066611394	0.186760918
Prevotella_timonensis	CD4	under500	1.012203306	130	0.315789474	0.072590928	0.201641468
Eubacterium_rectale	CD4	under500	-1.365664926	130	0.353383459	0.078377169	0.21473197
Alistipes_shahii	CD4	under500	0.521449321	130	0.135338346	0.079592804	0.216280309
Streptococcus_salivarius	CD4	under500	1.552574641	130	0.248120301	0.082909041	0.223073653
Campylobacter_rectus	CD4	under500	-0.556500079	130	0.135338346	0.083840277	0.224572169
Haemophilus_sputorum	CD4	under500	0.812258444	130	0.142857143	0.090597607	0.239465041
Holdemanella_biformis	Viral_Load	Detectable	1.340785224	130	0.303233083	0.002294023	0.006242238
Coprococcus_comes	Viral_Load	Detectable	2.690754065	130	0.345864662	0.004682192	0.011855415
Ruthenibacterium_lactatiformans	Viral_Load	Detectable	1.377326371	130	0.120300752	0.00769781	0.018219668
Desulfovibrionaceae_bacterium	Viral_Load	Detectable	1.356017472	130	0.127819549	0.011396232	0.025466441
Bifidobacterium_longum	Viral_Load	Detectable	1.052271227	130	0.105263158	0.017354227	0.037320918
Gemmiger_formicilis	Viral_Load	Detectable	1.132991958	130	0.233082707	0.04050168	0.080201346
Roseburia_faecis	Viral_Load	Detectable	1.640935159	130	0.263157895	0.043918782	0.085695184
Actinomyces_turicensis	Viral_Load	Detectable	-1.76995686	130	0.263157895	0.032433687	0.064867375
Anaerostipes_hadrus	Viral_Load	Detectable	-1.426633267	130	0.157894737	0.027313211	0.055741246
Neisseria_flavescens	Viral_Load	Detectable	-1.118830587	130	0.406015038	0.018550802	0.039260956
Bacteroides_vulgatus	Viral_Load	Detectable	1.841396572	130	0.278195489	0.055028578	0.106851608
Gemella_sanguinis	Viral_Load	Detectable	-1.035978544	130	0.338345865	0.056503023	0.109184585
Streptococcus_salivarius	Viral_Load	Detectable	1.854526973	130	0.248120301	0.057232398	0.110062303
Bacteroides_coagulans	Viral_Load	Detectable	0.732862441	130	0.105263158	0.05799104	0.110987636
Firmicutes_bacterium_CAG_110	Viral_Load	Detectable	-0.999171851	130	0.172932331	0.068745528	0.130943864
Criibacterium_bergeronii	Viral_Load	Detectable	1.010023018	130	0.142857143	0.071417289	0.135388225
Escherichia_coli	Viral_Load	Detectable	1.092396514	130	0.293233083	0.072182768	1.36E-01
Bacteroides_sp_CAG_530	Viral_Load	Detectable	1.034288207	130	0.203007519	0.079156597	1.49E-01
Streptococcus_sp_A12	Viral_Load	Detectable	-0.700864555	130	0.105263158	0.082808496	1.54E-01
Catenibacterium_mitsuokai	Viral_Load	Detectable	0.908858433	130	0.360902256	0.084787391	1.57E-01
Mitsuokella_multacida	Viral_Load	Detectable	1.343103678	130	0.157894737	0.087631073	1.62E-01
Streptococcus_infantis	Viral_Load	Detectable	-1.036310521	130	0.345864662	0.088590297	1.62E-01
Prevotella_copri	Viral_Load	Detectable	1.795410192	130	0.45112782	0.089199185	1.62E-01

Supplementary Table 4. (Continuation)

feature	metadata	value	coef	N	prevalence	pval	qval
Fusobacterium_equinum	Viral_Load	Detectable	-1.0858693	130	0.135338346	0.093208916	1.69E-01
Eubacterium_siraeum	Viral_Load	Detectable	1.330730052	130	0.180451128	0.096961615	1.75E-01
Corynebacterium_atypicum	Viral_Load	Detectable	-1.054834848	130	0.120300752	0.099737458	1.79E-01
Bifidobacterium_adolescentis	Viral_Load	Detectable	1.210632065	130	0.203007519	0.105269727	1.88E-01
Prevotella_oulorum	Viral_Load	Detectable	0.671910469	130	0.127819549	0.114581876	2.04E-01
Odoribacter_splanchnicus	Viral_Load	Detectable	0.852681754	130	0.15037594	0.116691389	2.07E-01
Roseburia_inulinivorans	Viral_Load	Detectable	1.27983495	130	0.323308271	0.130116181	0.228274002
Campylobacter_ureolyticus	Viral_Load	Detectable	1.062729539	130	0.187969925	0.131936649	0.230457029
Fusobacterium_periodonticum	Viral_Load	Detectable	-0.903861961	130	0.255639098	0.135673332	0.23595362
Oscillibacter_sp_57_20	Viral_Load	Detectable	1.029953211	130	0.330827068	0.137672836	0.238394521
Prevotella_sp_AM42_24	Viral_Load	Detectable	1.735993098	130	0.315789474	0.14154313	0.24403988
Campylobacter_hominis	Viral_Load	Detectable	0.766417509	130	0.180451128	0.142656401	0.244903693
Butyricimonas_virosa	Alcohol	yes	1.61593823	130	0.123076923	0.000472873	0.003315311
Bacteroides_uniformis	Alcohol	yes	1.947687243	130	0.176923077	0.001378448	0.008798604
Parabacteroides_distasonis	Alcohol	yes	1.830755981	130	0.230769231	0.001682452	0.010570381
Bacteroides_coagulans	Alcohol	yes	1.37681258	130	0.107692308	0.004561702	0.027234039
Parabacteroides_merdae	Alcohol	yes	1.037929869	130	0.130769231	0.005939585	0.034599527
Bacteroides_vulgatus	Alcohol	yes	2.501628714	130	0.284615385	0.007559525	0.042389862
Bifidobacterium_longum	Alcohol	yes	1.008344859	130	0.107692308	0.012460745	0.06558287
Desulfovibrio_piger	Alcohol	yes	1.650038179	130	0.169230769	0.012305835	0.06558287
Porphyromonas_sp_HMSC065F10	Alcohol	yes	1.665829142	130	0.169230769	0.021510888	0.10696828
Roseburia_faecis	Alcohol	yes	1.794735803	130	0.269230769	0.023118652	0.113698289
Corynebacterium_durum	Alcohol	yes	0.927432426	130	0.123076923	0.028471925	0.135580597
Ruminococcus_bromii	Alcohol	yes	1.528370455	130	0.184615385	0.043610607	0.188926816
Treponema_denticola	Alcohol	yes	-0.669239616	130	0.107692308	0.044437653	0.190447084
Porphyromonas_asaccharolytica	Alcohol	yes	1.328616145	130	0.223076923	0.04551349	0.193674424
Fusobacterium_mortiferum	Alcohol	yes	1.199934231	130	0.192307692	0.046703441	0.197338482
Alistipes_shahii	Alcohol	yes	0.757388729	130	0.138461538	0.052460451	0.217719532
Flavonifractor_plautii	Alcohol	yes	1.228183229	130	0.215384615	0.052615554	0.217719532
Agathobaculum_butyriciproducens	Alcohol	yes	1.283285004	130	0.146153846	0.057500008	0.232323263
Bifidobacterium_adolescentis	Alcohol	yes	1.541287533	130	0.207692308	0.059277592	0.237110367
Eubacterium_rectale	Alcohol	yes	1.618661885	130	0.361538462	0.062271723	0.245809433
Anaerostipes_hadrus	Alcohol	yes	1.208328743	130	0.161538462	0.063646345	0.247972775
Prevotella_copri	nonIVD	yes	2.274876824	130	0.461538462	0.007298267	0.041116999
Bacteroides_coagulans	nonIVD	yes	-0.844016426	130	0.107692308	0.031340017	0.14633471
Porphyromonas_sp_HMSC065F10	nonIVD	yes	-1.209601339	130	0.169230769	0.039760115	0.176711622
Alistipes_shahii	nonIVD	yes	-0.652476802	130	0.138461538	0.040231407	0.178146952
Campylobacter_hominis	nonIVD	yes	-1.045450062	130	0.184615385	0.041546243	0.181292697
Haemophilus_sp_HMSC71H05	nonIVD	yes	0.979485196	130	0.176923077	0.046563983	0.197338482
Prevotella_sp_CAG_5226	nonIVD	yes	1.605680097	130	0.161538462	0.049935478	0.208064492
Prevotella_sp_CAG_520	nonIVD	yes	1.788327862	130	0.346153846	0.054254445	0.222963471
Prevotella_sp_885	nonIVD	yes	1.41884056	130	0.346153846	0.055340026	0.225111969
Granulicatella_elegans	nonIVD	yes	2.403903318	130	0.584615385	0.009458833	0.05182922
Prevotella_sp_885	nonIVD	yes	1.768089953	130	0.346153846	0.014235229	0.074270757
Prevotella_copri	nonIVD	yes	1.797739553	130	0.461538462	0.025387603	0.122843242
Campylobacter_hominis	nonIVD	yes	-0.986674699	130	0.184615385	0.04344584	0.187220934
Alistipes_shahii	nonIVD	yes	-0.607666701	130	0.138461538	0.04551625	0.190977275
Streptococcus_oralis	Tobacco	yes	1.598454668	130	0.623076923	0.025128768	0.121590813
Eubacterium_sp_CAG_180	Tobacco	yes	1.139359334	130	0.215384615	0.063620946	0.247972775
Gemella_sanguinis	Tobacco	yes	0.846946132	130	0.346153846	0.054476401	0.22311154
Corynebacterium_durum	Tobacco	yes	-0.68070735	130	0.123076923	0.059041865	0.236957317
Eubacterium_eligens	Tobacco	yes	-0.723029188	130	0.115384615	0.033770131	0.155862144
Streptococcus_sp_A12	Tobacco	yes	-0.799747145	130	0.107692308	0.013724302	0.071017951
Firmicutes_bacterium_CAG_83	Tobacco	yes	-0.812459292	130	0.261538462	0.049491106	0.206931454
Lachnospira_pectinoschiza	Tobacco	yes	-0.914144641	130	0.115384615	0.006892131	0.039196952
Faecalibacterium_prausnitzii	Tobacco	yes	-0.998504718	130	0.438461538	0.040848648	0.179554497
Collinsella_stercoris	Tobacco	yes	-1.001114184	130	0.192307692	0.028059459	0.134148806
Parabacteroides_distasonis	Tobacco	yes	-1.029496174	130	0.230769231	0.036348006	0.166479416
Odoribacter_splanchnicus	Tobacco	yes	-1.032191815	130	0.153846154	0.045103275	0.192611851
Butyricimonas_virosa	Tobacco	yes	-1.102051104	130	0.123076923	0.004838479	0.028601849
Flavonifractor_plautii	Tobacco	yes	-1.117030554	130	0.215384615	0.039215374	0.174938472
Desulfovibrio_piger	Tobacco	yes	-1.167315282	130	0.169230769	0.037309489	0.170233409
Oscillibacter_sp_CAG_241	Tobacco	yes	-1.251657832	130	0.284615385	0.040669854	0.179425828
Prevotella_sp_CAG_279	Tobacco	yes	-1.27037772	130	0.3	0.023338766	0.114312325
Peptoniphilus_lacrimalis	Tobacco	yes	-1.27588801	130	0.192307692	0.042939578	0.186693818
Agathobaculum_butyriciproducens	Tobacco	yes	-1.353251953	130	0.146153846	0.019473765	0.097368825
Fusobacterium_equinum	Tobacco	yes	-1.441043751	130	0.138461538	0.012431017	0.06558287
Eubacterium_rectale	Tobacco	yes	-1.538198159	130	0.361538462	0.038353569	0.17302362
Anaerostipes_hadrus	Tobacco	yes	-1.58606697	130	0.161538462	0.004754566	0.028244946
Bacteroides_uniformis	Tobacco	yes	-1.600732711	130	0.176923077	0.002044442	0.01277776
Bacteroides_vulgatus	Tobacco	yes	-1.651262906	130	0.284615385	0.037783928	0.171097034
Ruminococcus_bromii	Tobacco	yes	-1.711270061	130	0.184615385	0.008548869	0.046843119

Supplementary Table 4. (Continuation)

feature	metadata	value	coef	N	prevalence	pval	qval
Roseburia_faecis	Tobacco	yes	-1.821293438	130	0.269230769	0.007201759	0.040764673
Slackia_isoflavoniconvertens	ART	yes	-1.440395187	130	0.238461538	0.003443344	0.008054607
Ruthenibacterium_lactatiformans	ART	yes	-1.72796727	130	0.123076923	0.003476334	0.008084498
Prevotella_sp_AM42_24	ART	yes	-4.124431883	130	0.323076923	0.003733098	0.008532796
Haemophilus_paraphrohaemolyticus	ART	yes	-1.617440145	130	0.123076923	0.00465383	0.010399621
Brachyspira_pilosicoli	ART	yes	-1.714671261	130	0.107692308	0.007099667	0.015186454
Bacteroides_sp_CAG_530	ART	yes	-1.853919959	130	0.207692308	0.01061699	0.02211873
Haemophilus_parahaemolyticus	ART	yes	-1.881376953	130	0.230769231	0.011248011	0.023191775
Holdemanella_biformis	ART	yes	-2.092791119	130	0.338461538	0.012715863	0.025819012
Prevotella_sp_CAG_1092	ART	yes	-1.2606975	130	0.169230769	0.015914732	0.031989412
Coprococcus_comes	ART	yes	-2.79895867	130	0.353846154	0.01659652	0.03319304
Prevotella_sp_CAG_520	ART	yes	-3.227501674	130	0.346153846	0.019306396	0.038230487
Roseburia_faecis	ART	yes	-2.148246105	130	0.269230769	0.026887024	0.052462485
Brachyspira_sp_CAG_700	ART	yes	-2.055138976	130	0.2	0.029547403	0.057373599
Oscillibacter_sp_57_20	ART	yes	-1.13234832	130	0.3	0.031399296	0.060674968
Oscillibacter_sp_CAG_241	ART	yes	-1.661793226	130	0.284615385	0.032987816	0.063438109
Lachnospira_pectinoschiza	ART	yes	-0.897298696	130	0.115384615	0.034197969	0.065450659
Bifidobacterium_longum	ART	yes	-1.113177917	130	0.107692308	0.038653238	0.073625216
Bacteroides_coagulans	ART	yes	-1.103097339	130	0.107692308	0.041283741	0.077975423
Prevotella_salivae	ART	yes	-1.142772021	130	0.123076923	0.041326974	0.077975423
Prevotella_sp_oral_taxon_306	ART	yes	-1.035840258	130	0.123076923	0.042189409	0.079228936
Streptococcus_infantis	ART	yes	1.480128307	130	0.353846154	0.057039353	0.105628431
Alphapapillomavirus_3	ART	yes	-1.475179297	130	0.146153846	0.057345409	0.105705822
Bifidobacterium_adolescentis	ART	yes	-1.707673685	130	0.207692308	0.060305554	0.110652393
Mitsuokella_jalaludinii	ART	yes	-1.638385809	130	0.215384615	0.061249706	0.111363102
Streptococcus_salivarius	ART	yes	-2.227293862	130	0.253846154	0.061111256	0.111363102
Mitsuokella_multacida	ART	yes	-1.718692443	130	0.161538462	0.064524778	0.116786928
Streptococcus_phage_Dp_1	ART	yes	1.003149368	130	0.153846154	0.076450973	0.137749501
Actinomyces_graevenitzii	ART	yes	-1.274366855	130	0.130769231	0.077322526	0.138695115
Neisseria_cinerea	ART	yes	-1.250937	130	0.146153846	0.080881425	0.144431117
Fusicatenibacter_saccharivorans	ART	yes	-1.670088993	130	0.3	0.081823269	0.14546359
Dorea_formicigenerans	ART	yes	-1.387164821	130	0.361538462	0.084169488	0.148972544
Prevotella_stercorea	ART	yes	-1.470745107	130	0.346153846	0.087005843	0.153314261
Fusobacterium_periodonticum	ART	yes	1.191475577	130	0.261538462	0.092234233	0.161814444
Firmicutes_bacterium_CAG_83	ART	yes	-0.903778636	130	0.261538462	0.0975713	0.170430218
Eubacterium_siraeum	ART	yes	-1.60209364	130	0.184615385	0.098052711	0.170526453
Megasphaera_elsdenii	ART	yes	-1.56505151	130	0.269230769	0.099256325	0.171872424
Desulfovibrionaceae_bacterium	ART	yes	-1.101821092	130	0.130769231	0.102158397	0.176135167
Escherichia_coli	ART	yes	-1.206737866	130	0.3	0.108366214	0.185241392
Faecalibacterium_prausnitzii	ART	yes	-1.101054118	130	0.438461538	0.117947317	0.200761391
Prevotella_oris	ART	yes	0.768725972	130	0.176923077	0.119606837	0.202723453
Capnocytophaga_gingivalis	ART	yes	0.922889379	130	0.192307692	0.12602653	0.211795915
Prevotella_copri	ART	yes	-2.015708533	130	0.461538462	0.126548059	0.211795915
Eubacterium_sp_CAG_180	ART	yes	-1.170525252	130	0.215384615	0.128044109	0.213406849
Haemophilus_phage_Aaphi23	ART	yes	-0.589956011	130	0.130769231	0.135899107	0.225558683
Neisseria_flavescens	ART	yes	0.953510495	130	0.415384615	0.13665849	0.225881802
Bacteroides_fragilis	ART	yes	-1.382246177	130	0.176923077	0.143011871	0.235410486
Prevotella_denticola	ART	yes	0.87230876	130	0.207692308	0.150356139	0.246485474

Supplementary Table 5: Statistics of variables related to sexual behavior and consumption habits of MSM and TGW.

	**MSM**	**TGW**	
**Sexual behavior**	Mean-SD		*P value **
**AFSI**	16.5-2.9	14.5-2.7	0.0017
**AASI**	18.6-3.6	14.6-2.8	< 0.0001
**AOSI**	18.1-3.4	14.7-3.1	< 0.0001
**NSPLM**	*Cases*		*P value ***
None	12	2	< 0.00001
1-10	35	13	
11-50	-	8	
>50	-	8	
**NSPL**	*Cases*		*P value ***
1-10	5	*1*	< 0.00001
11-50	*20*	*2*	
51-100	*12*	*1*	
>100	*8*	*25*	
ND	*2*	*2*	
**CUAS**	*Cases*		*P value ***
Yes	*20*	*21*	0.058
No	*22*	*9*	
ND	*5*	*1*	
**CUOS**	*Cases*		*P value ***
Yes	*31*	*20*	0.516
No	*11*	*10*	
ND	*5*	*1*	
**Consumption habits**			
**Alcohol**	*Casesl*		*P value ***
Yes	*38/45*	*24/31*	0.437
no	*7/45*	*7/31*	
ND	*2*	-	
**Tobacco**	*Cases*		*P value ***
Yes	*28/47*	*20/31*	0.206
no	*19/47*	*11/31*	
**non-IV drug use**	*Cases*		*P value ***
Yes	*27/47*	*21/31*	0.360
No	*20/47*	*10/31*	
**IV drug use**	*Cases*		*P value ***
Yes	*1/47*	*0/31*	0.584
No	*46/47*	*31/31*	

[1]**Note: AFSI**: Age at First Sexual Intercourse. **AASI**: Age of Anal Sex Initiation **AOSI:** Age of Oral Sex Initiation **NPLSM:** Number of Sexual Partners in Last Month **NSPL:** Number of Sexual Partners in Life. **CUAS:** Condom Use in Anal Sex **CUOS:** Condom Use in Oral Sex. **non-IV drug use:** non-intravenous drug use. **IV drug use:** Intravenous drug use. * p value obtained by T-test . ** p value obtained by Fisher's Exact Test.

Supplementary table 6. Significant associations of pathways and site

PWY	sps	metadata	value	coef	N	Prevalence	pval	qval
PWY.6122.5.aminoimidazoleribonucleotidebiosynthesisII	Actinomyces_naeslundii	site	oral	0.62	130	0.1308	0.0040	0.0159
PWY.6277.superpathwayof5.aminoimidazoleribonucleotidebiosynthesis	Actinomyces_naeslundii	site	oral	0.62	130	0.1308	0.0040	0.0159
PWY.7220.adenosinedeoxyribonucleotidesdenovobiosynthesisII	Actinomyces_naeslundii	site	oral	0.60	130	0.1077	0.0081	0.0303
PWY.7222.guanosinedeoxyribonucleotidesdenovobiosynthesisII	Actinomyces_naeslundii	site	oral	0.60	130	0.1077	0.0081	0.0303
PWY.7220.adenosinedeoxyribonucleotidesdenovobiosynthesisII	Actinomyces_oris	site	oral	0.83	130	0.1385	0.0003	0.0017
PWY.7222.guanosinedeoxyribonucleotidesdenovobiosynthesisII	Actinomyces_oris	site	oral	0.83	130	0.1385	0.0003	0.0017
PWY.6151.S.adenosyl.L.methioninesalvageI	Actinomyces_oris	site	oral	0.93	130	0.1385	0.0006	0.0035
PWY.7111.pyruvatefermentationtoisobutanol.engineered.	Actinomyces_oris	site	oral	0.84	130	0.1308	0.0009	0.0047
VALSYN.PWY.L.valinebiosynthesis	Actinomyces_oris	site	oral	0.84	130	0.1308	0.0009	0.0047
PWY.7221.guanosineribonucleotidesdenovobiosynthesis	Actinomyces_oris	site	oral	0.66	130	0.1385	0.0026	0.0112
PWY.6122.5.aminoimidazoleribonucleotidebiosynthesisII	Actinomyces_oris	site	oral	0.71	130	0.1538	0.0037	0.0150
PWY.6277.superpathwayof5.aminoimidazoleribonucleotidebiosynthesis	Actinomyces_oris	site	oral	0.71	130	0.1538	0.0037	0.0150
PWY.6121.5.aminoimidazoleribonucleotidebiosynthesisI	Actinomyces_oris	site	oral	0.69	130	0.1231	0.0130	0.0460
VALSYN.PWY.L.valinebiosynthesis	Haemophilus_haemolyticus	site	oral	2.80	130	0.3154	0.0000	0.0000
PWY.7111.pyruvatefermentationtoisobutanol.engineered.	Haemophilus_haemolyticus	site	oral	2.57	130	0.3077	0.0000	0.0000
PWY.7220.adenosinedeoxyribonucleotidesdenovobiosynthesisII	Haemophilus_haemolyticus	site	oral	2.16	130	0.2846	0.0000	0.0000
PWY.7222.guanosinedeoxyribonucleotidesdenovobiosynthesisII	Haemophilus_haemolyticus	site	oral	2.16	130	0.2846	0.0000	0.0000
PWY.7357.thiaminephosphateformationfrompyrithiamineandoxythiamine.y	Haemophilus_haemolyticus	site	oral	1.19	130	0.2538	0.0000	0.0001
PWY.6936.seleno.aminoacidbiosynthesis.plants.	Haemophilus_haemolyticus	site	oral	1.60	130	0.2615	0.0000	0.0001
PWY.5103.L.isoleucinebiosynthesisIII	Haemophilus_haemolyticus	site	oral	1.29	130	0.2615	0.0000	0.0002
BRANCHED.CHAIN.AA.SYN.PWY.superpathwayofbranchedchainaminoacidbi	Haemophilus_haemolyticus	site	oral	1.27	130	0.2615	0.0000	0.0002
ILEUSYN.PWY.L.isoleucinebiosynthesisI.fromthreonine.	Haemophilus_haemolyticus	site	oral	1.24	130	0.2615	0.0000	0.0002
PWY.6897.thiaminediphosphatesalvageII	Haemophilus_haemolyticus	site	oral	0.98	130	0.1846	0.0000	0.0002
NONOXIPENT.PWY.pentosephosphatepathway.non.oxidativebranch.I	Haemophilus_haemolyticus	site	oral	1.02	130	0.2538	0.0000	0.0005
PWY.6151.S.adenosyl.L.methioninesalvageI	Haemophilus_haemolyticus	site	oral	1.33	130	0.2385	0.0001	0.0010
PWY.6122.5.aminoimidazoleribonucleotidebiosynthesisII	Haemophilus_haemolyticus	site	oral	0.97	130	0.2000	0.0001	0.0012
PWY.6277.superpathwayof5.aminoimidazoleribonucleotidebiosynthesis	Haemophilus_haemolyticus	site	oral	0.97	130	0.2000	0.0001	0.0012
PWY.6121.5.aminoimidazoleribonucleotidebiosynthesisI	Haemophilus_haemolyticus	site	oral	0.70	130	0.1769	0.0002	0.0014
HSERMETANA.PWY.L.methioninebiosynthesisIII	Haemophilus_haemolyticus	site	oral	1.09	130	0.1846	0.0002	0.0015
PWY.5695.inosine5..phosphatedegradation	Haemophilus_haemolyticus	site	oral	0.97	130	0.2308	0.0002	0.0017
PWY.1042.glycolysisIV	Haemophilus_haemolyticus	site	oral	1.42	130	0.2385	0.0003	0.0018
PWY.6124.inosine.5..phosphatebiosynthesisII	Haemophilus_haemolyticus	site	oral	0.97	130	0.2308	0.0004	0.0023
PWY.7219.adenosineribonucleotidesdenovobiosynthesis	Haemophilus_haemolyticus	site	oral	1.02	130	0.2462	0.0005	0.0029
PWY.6609.adenineandadenosinesalvageIII	Haemophilus_haemolyticus	site	oral	0.80	130	0.1615	0.0006	0.0036
NAGLIPASYN.PWY.lipidIVAbiosynthesis.E.coli.	Haemophilus_haemolyticus	site	oral	0.77	130	0.1462	0.0010	0.0050
PWY.6737.starchdegradationV	Haemophilus_haemolyticus	site	oral	0.77	130	0.1538	0.0011	0.0056
PWY0.1586.peptidoglycanmaturation.meso.diaminopimelatecontaining.	Haemophilus_haemolyticus	site	oral	1.04	130	0.2231	0.0012	0.0058
FERMENTATION.PWY.mixedacidfermentation	Haemophilus_haemolyticus	site	oral	0.44	130	0.1077	0.0012	0.0061
PWY.7199.pyrimidinedeoxyribonucleosidessalvage	Haemophilus_haemolyticus	site	oral	0.84	130	0.1769	0.0014	0.0068
PEPTIDOGLYCANSYN.PWY.peptidoglycanbiosynthesisI.meso.diaminopimelat	Haemophilus_haemolyticus	site	oral	0.75	130	0.1462	0.0015	0.0071
PWY.6385.peptidoglycanbiosynthesisIII.mycobacteria.	Haemophilus_haemolyticus	site	oral	0.75	130	0.1462	0.0015	0.0071
PWY0.1479.tRNAprocessing	Haemophilus_haemolyticus	site	oral	0.61	130	0.1385	0.0015	0.0073
PWY.6387.UDP.N.acetylmuramoyl.pentapeptidebiosynthesisI.meso.diamino	Haemophilus_haemolyticus	site	oral	0.76	130	0.1462	0.0016	0.0074
PWY.6700.queuosinebiosynthesisI.denovo.	Haemophilus_haemolyticus	site	oral	0.95	130	0.1769	0.0017	0.0078
PWY.6317.D.galactosedegradationI.Leloirpathway.	Haemophilus_haemolyticus	site	oral	0.74	130	0.1615	0.0024	0.0105
UDPNAGSYN.PWY.UDP.N.acetyl.D.glucosaminebiosynthesisI	Haemophilus_haemolyticus	site	oral	0.78	130	0.2000	0.0025	0.0108
PWY.6123.inosine.5..phosphatebiosynthesisI	Haemophilus_haemolyticus	site	oral	0.94	130	0.1923	0.0025	0.0108
PWY.3841.folatetransformationsII.plants.	Haemophilus_haemolyticus	site	oral	0.75	130	0.2000	0.0026	0.0110
NONMEVIPP.PWY.methylerythritolphosphatepathwayI	Haemophilus_haemolyticus	site	oral	0.68	130	0.1385	0.0032	0.0132
GLYCOLYSIS.glycolysisI.fromglucose6.phosphate.	Haemophilus_haemolyticus	site	oral	0.51	130	0.1154	0.0035	0.0143
ANAGLYCOLYSIS.PWY.glycolysisIII.fromglucose.	Haemophilus_haemolyticus	site	oral	0.51	130	0.1154	0.0035	0.0144
PWY.5484.glycolysisII.fromfructose6.phosphate.	Haemophilus_haemolyticus	site	oral	0.51	130	0.1154	0.0035	0.0144
PWY.6386.UDP.N.acetylmuramoyl.pentapeptidebiosynthesisII.lysine.contain	Haemophilus_haemolyticus	site	oral	0.51	130	0.1154	0.0035	0.0144
PWY.7221.guanosineribonucleotidesdenovobiosynthesis	Haemophilus_haemolyticus	site	oral	0.83	130	0.1846	0.0037	0.0150
PWY.4242	Haemophilus_haemolyticus	site	oral	0.66	130	0.1385	0.0038	0.0154
PWY.5861.superpathwayofdemethylmenaquinol.8biosynthesisI	Haemophilus_haemolyticus	site	oral	0.58	130	0.1462	0.0050	0.0198
DAPLYSINESYN.PWY.L.lysinebiosynthesisI	Haemophilus_haemolyticus	site	oral	0.52	130	0.1308	0.0055	0.0213
PWY.7111.pyruvatefermentationtoisobutanol.engineered.	Haemophilus_parahaemolyticus	site	oral	1.14	130	0.1308	0.0045	0.0178
VALSYN.PWY.L.valinebiosynthesis	Haemophilus_parahaemolyticus	site	oral	1.16	130	0.1231	0.0053	0.0209
VALSYN.PWY.L.valinebiosynthesis	Haemophilus_parainfluenzae	site	oral	1.90	130	0.3308	0.0000	0.0000
PWY.7111.pyruvatefermentationtoisobutanol.engineered.	Haemophilus_parainfluenzae	site	oral	1.70	130	0.3077	0.0000	0.0000
PWY.7220.adenosinedeoxyribonucleotidesdenovobiosynthesisII	Haemophilus_parainfluenzae	site	oral	2.74	130	0.3154	0.0000	0.0000
PWY.7222.guanosinedeoxyribonucleotidesdenovobiosynthesisII	Haemophilus_parainfluenzae	site	oral	2.74	130	0.3154	0.0000	0.0000
NONOXIPENT.PWY.pentosephosphatepathway.non.oxidativebranch.I	Haemophilus_parainfluenzae	site	oral	1.93	130	0.2923	0.0000	0.0000
PWY.1042.glycolysisIV	Haemophilus_parainfluenzae	site	oral	1.91	130	0.2846	0.0000	0.0000
PWY.6936.seleno.aminoacidbiosynthesis.plants.	Haemophilus_parainfluenzae	site	oral	2.10	130	0.2769	0.0000	0.0000
PWY.6700.queuosinebiosynthesisI.denovo.	Haemophilus_parainfluenzae	site	oral	1.58	130	0.2615	0.0000	0.0000
PWY.7219.adenosineribonucleotidesdenovobiosynthesis	Haemophilus_parainfluenzae	site	oral	1.96	130	0.2692	0.0000	0.0000
PWY0.1586.peptidoglycanmaturation.meso.diaminopimelatecontaining.	Haemophilus_parainfluenzae	site	oral	2.19	130	0.2538	0.0000	0.0001
PWY.6124.inosine.5..phosphatebiosynthesisII	Haemophilus_parainfluenzae	site	oral	1.30	130	0.2538	0.0000	0.0001
PWY.5695.inosine5..phosphatedegradation	Haemophilus_parainfluenzae	site	oral	1.35	130	0.2538	0.0000	0.0001
PWY.7221.guanosineribonucleotidesdenovobiosynthesis	Haemophilus_parainfluenzae	site	oral	1.26	130	0.2538	0.0000	0.0001
UDPNAGSYN.PWY.UDP.N.acetyl.D.glucosaminebiosynthesisI	Haemophilus_parainfluenzae	site	oral	1.46	130	0.2154	0.0000	0.0001
PWY.6151.S.adenosyl.L.methioninesalvageI	Haemophilus_parainfluenzae	site	oral	2.06	130	0.2615	0.0000	0.0001
PWY.5484.glycolysisII.fromfructose6.phosphate.	Haemophilus_parainfluenzae	site	oral	1.19	130	0.2308	0.0000	0.0002
PWY.7357.thiaminephosphateformationfrompyrithiamineandoxythiamine.y	Haemophilus_parainfluenzae	site	oral	1.65	130	0.2462	0.0000	0.0003
ILEUSYN.PWY.L.isoleucinebiosynthesisI.fromthreonine.	Haemophilus_parainfluenzae	site	oral	1.22	130	0.2538	0.0000	0.0003
FERMENTATION.PWY.mixedacidfermentation	Haemophilus_parainfluenzae	site	oral	1.09	130	0.2231	0.0000	0.0003

Supplementary Table 6. (Continuation)

PWY	sps	metadata	value	coef	N	Prevalence	pval	qval
BRANCHED.CHAIN.AA.SYN.PWY.superpathwayofbranchedchainaminoacidbi	Haemophilus_parainfluenzae	site	oral	1.30	130	0.2538	0.0000	0.0004
PWY.6122.5.aminoimidazoleribonucleotidebiosynthesisII	Haemophilus_parainfluenzae	site	oral	0.97	130	0.2308	0.0000	0.0004
PWY.6277.superpathwayof5.aminoimidazoleribonucleotidebiosynthesis	Haemophilus_parainfluenzae	site	oral	0.97	130	0.2308	0.0000	0.0004
PWY.5103.L.isoleucinebiosynthesisIII	Haemophilus_parainfluenzae	site	oral	1.22	130	0.2538	0.0000	0.0005
PWY.6123.inosine.5..phosphatebiosynthesisI	Haemophilus_parainfluenzae	site	oral	1.11	130	0.2154	0.0001	0.0006
GLYCOLYSIS.glycolysisI.fromglucose6.phosphate.	Haemophilus_parainfluenzae	site	oral	0.99	130	0.2077	0.0001	0.0011
ANAGLYCOLYSIS.PWY.glycolysisIII.fromglucose.	Haemophilus_parainfluenzae	site	oral	0.97	130	0.2077	0.0001	0.0011
NONMEVIPP.PWY.methylerythritolphosphatepathwayI	Haemophilus_parainfluenzae	site	oral	1.04	130	0.1923	0.0001	0.0011
HSERMETANA.PWY.L.methioninebiosynthesisIII	Haemophilus_parainfluenzae	site	oral	1.14	130	0.1769	0.0002	0.0015
PWY.6387.UDP.N.acetylmuramoyl.pentapeptidebiosynthesisI.meso.diamino	Haemophilus_parainfluenzae	site	oral	0.91	130	0.1769	0.0003	0.0022
PWY.6121.5.aminoimidazoleribonucleotidebiosynthesisI	Haemophilus_parainfluenzae	site	oral	0.80	130	0.1846	0.0004	0.0023
PWY.7199.pyrimidinedeoxyribonucleosidessalvage	Haemophilus_parainfluenzae	site	oral	0.92	130	0.1923	0.0004	0.0025
NAGLIPASYN.PWY.lipidIVAbiosynthesis.E.coli.	Haemophilus_parainfluenzae	site	oral	1.11	130	0.1846	0.0004	0.0026
PWY.6609.adenineandadenosinesalvageIII	Haemophilus_parainfluenzae	site	oral	1.25	130	0.2077	0.0005	0.0027
PWY.6317.D.galactosedegradationI.Leloirpathway.	Haemophilus_parainfluenzae	site	oral	1.23	130	0.2077	0.0005	0.0029
PWY.6385.peptidoglycanbiosynthesisIII.mycobacteria.	Haemophilus_parainfluenzae	site	oral	0.91	130	0.1692	0.0005	0.0031
PEPTIDOGLYCANSYN.PWY.peptidoglycanbiosynthesisI.meso.diaminopimelat	Haemophilus_parainfluenzae	site	oral	0.88	130	0.1692	0.0006	0.0035
DAPLYSINESYN.PWY.L.lysinebiosynthesisI	Haemophilus_parainfluenzae	site	oral	0.80	130	0.1692	0.0009	0.0048
PWY.3841.folatetransformationsII.plants.	Haemophilus_parainfluenzae	site	oral	0.74	130	0.1615	0.0018	0.0085
PWY.6897.thiaminediphosphatesalvageII	Haemophilus_parainfluenzae	site	oral	1.02	130	0.1846	0.0021	0.0093
PWY.6737.starchdegradationV	Haemophilus_parainfluenzae	site	oral	0.80	130	0.1846	0.0021	0.0094
PWY0.1479.tRNAprocessing	Haemophilus_parainfluenzae	site	oral	0.67	130	0.1308	0.0026	0.0110
PWY.4242	Haemophilus_parainfluenzae	site	oral	0.69	130	0.1462	0.0030	0.0126
PWY.6386.UDP.N.acetylmuramoyl.pentapeptidebiosynthesisII.lysine.contain	Haemophilus_parainfluenzae	site	oral	0.66	130	0.1385	0.0056	0.0219
PWY.5695.inosine5..phosphatedegradation	Haemophilus_quentini	site	oral	0.85	130	0.1308	0.0003	0.0021
VALSYN.PWY.L.valinebiosynthesis	Haemophilus_quentini	site	oral	1.08	130	0.1615	0.0004	0.0026
NONOXIPENT.PWY.pentosephosphatepathway.non.oxidativebranch.I	Haemophilus_quentini	site	oral	0.72	130	0.1385	0.0005	0.0030
PWY.6936.seleno.aminoacidbiosynthesis.plants.	Haemophilus_quentini	site	oral	0.62	130	0.1308	0.0005	0.0032
PWY.7220.adenosinedeoxyribonucleotidesdenovobiosynthesisII	Haemophilus_quentini	site	oral	0.93	130	0.1615	0.0006	0.0036
PWY.7222.guanosinedeoxyribonucleotidesdenovobiosynthesisII	Haemophilus_quentini	site	oral	0.93	130	0.1615	0.0006	0.0036
PWY.6124.inosine.5..phosphatebiosynthesisII	Haemophilus_quentini	site	oral	0.52	130	0.1077	0.0009	0.0047
PWY.7111.pyruvatefermentationtoisobutanol.engineered.	Haemophilus_quentini	site	oral	1.07	130	0.1462	0.0010	0.0050
PWY.1042.glycolysisIV	Haemophilus_quentini	site	oral	0.55	130	0.1308	0.0010	0.0052
PWY.5103.L.isoleucinebiosynthesisIII	Haemophilus_quentini	site	oral	1.02	130	0.1462	0.0011	0.0057
BRANCHED.CHAIN.AA.SYN.PWY.superpathwayofbranchedchainaminoacidbi	Haemophilus_quentini	site	oral	1.00	130	0.1462	0.0013	0.0065
PWY.7219.adenosineribonucleotidesdenovobiosynthesis	Haemophilus_quentini	site	oral	0.95	130	0.1462	0.0015	0.0070
ILEUSYN.PWY.L.isoleucinebiosynthesisI.fromthreonine.	Haemophilus_quentini	site	oral	0.94	130	0.1462	0.0018	0.0082
PWY.6151.S.adenosyl.L.methioninesalvageI	Haemophilus_quentini	site	oral	0.57	130	0.1154	0.0029	0.0123
UDPNAGSYN.PWY.UDP.N.acetyl.D.glucosaminebiosynthesisI	Haemophilus_quentini	site	oral	0.53	130	0.1154	0.0030	0.0128
PWY0.1586.peptidoglycanmaturation.meso.diaminopimelatecontaining.	Haemophilus_quentini	site	oral	0.83	130	0.1231	0.0033	0.0137
PWY.7357.thiaminephosphateformationfrompyrithiamineandoxythiamine.y	Haemophilus_quentini	site	oral	0.48	130	0.1154	0.0048	0.0190
VALSYN.PWY.L.valinebiosynthesis	Haemophilus_sputorum	site	oral	0.56	130	0.1077	0.0015	0.0073
PWY.7111.pyruvatefermentationtoisobutanol.engineered.	Haemophilus_sputorum	site	oral	0.42	130	0.1077	0.0019	0.0086
PWY.7219.adenosineribonucleotidesdenovobiosynthesis	Neisseria_flavescens	site	oral	1.47	130	0.2154	0.0000	0.0001
PWY.6121.5.aminoimidazoleribonucleotidebiosynthesisI	Neisseria_flavescens	site	oral	1.23	130	0.1615	0.0000	0.0001
PWY.7221.guanosineribonucleotidesdenovobiosynthesis	Neisseria_flavescens	site	oral	1.13	130	0.1615	0.0000	0.0001
UDPNAGSYN.PWY.UDP.N.acetyl.D.glucosaminebiosynthesisI	Neisseria_flavescens	site	oral	1.43	130	0.2154	0.0000	0.0001
PWY.6936.seleno.aminoacidbiosynthesis.plants.	Neisseria_flavescens	site	oral	1.46	130	0.1769	0.0000	0.0001
PWY.6700.queuosinebiosynthesisI.denovo.	Neisseria_flavescens	site	oral	1.06	130	0.1615	0.0000	0.0002
PWY.7111.pyruvatefermentationtoisobutanol.engineered.	Neisseria_flavescens	site	oral	1.14	130	0.2000	0.0000	0.0003
PWY.6124.inosine.5..phosphatebiosynthesisII	Neisseria_flavescens	site	oral	0.97	130	0.1769	0.0000	0.0003
PWY.6151.S.adenosyl.L.methioninesalvageI	Neisseria_flavescens	site	oral	1.36	130	0.1846	0.0000	0.0003
PWY.6387.UDP.N.acetylmuramoyl.pentapeptidebiosynthesisI.meso.diamino	Neisseria_flavescens	site	oral	0.95	130	0.1538	0.0000	0.0003
PWY.6385.peptidoglycanbiosynthesisIII.mycobacteria.	Neisseria_flavescens	site	oral	0.93	130	0.1538	0.0000	0.0003
PEPTIDOGLYCANSYN.PWY.peptidoglycanbiosynthesisI.meso.diaminopimelat	Neisseria_flavescens	site	oral	0.91	130	0.1538	0.0000	0.0004
PWY.6122.5.aminoimidazoleribonucleotidebiosynthesisII	Neisseria_flavescens	site	oral	1.06	130	0.1846	0.0000	0.0004
PWY.6277.superpathwayof5.aminoimidazoleribonucleotidebiosynthesis	Neisseria_flavescens	site	oral	1.06	130	0.1846	0.0000	0.0004
VALSYN.PWY.L.valinebiosynthesis	Neisseria_flavescens	site	oral	1.09	130	0.1846	0.0000	0.0005
PWY.6609.adenineandadenosinesalvageIII	Neisseria_flavescens	site	oral	1.12	130	0.1154	0.0001	0.0009
PWY0.1586.peptidoglycanmaturation.meso.diaminopimelatecontaining.	Neisseria_flavescens	site	oral	1.23	130	0.1615	0.0001	0.0009
PWY.7220.adenosinedeoxyribonucleotidesdenovobiosynthesisII	Neisseria_flavescens	site	oral	0.78	130	0.1231	0.0001	0.0009
PWY.7222.guanosinedeoxyribonucleotidesdenovobiosynthesisII	Neisseria_flavescens	site	oral	0.78	130	0.1231	0.0001	0.0009
PWY.6123.inosine.5..phosphatebiosynthesisI	Neisseria_flavescens	site	oral	1.01	130	0.1692	0.0001	0.0010
PWY.5695.inosine5..phosphatedegradation	Neisseria_flavescens	site	oral	0.97	130	0.1154	0.0001	0.0010
DAPLYSINESYN.PWY.L.lysinebiosynthesisI	Neisseria_flavescens	site	oral	0.74	130	0.1154	0.0001	0.0011
NONOXIPENT.PWY.pentosephosphatepathway.non.oxidativebranch.I	Neisseria_flavescens	site	oral	0.86	130	0.1385	0.0001	0.0012
PWY.3841.folatetransformationsII.plants.	Neisseria_flavescens	site	oral	0.76	130	0.1231	0.0001	0.0012
PWY.5855.ubiquinol.7biosynthesis.earlydecarboxylation.	Neisseria_flavescens	site	oral	0.76	130	0.1231	0.0003	0.0019
PWY.5856.ubiquinol.9biosynthesis.earlydecarboxylation.	Neisseria_flavescens	site	oral	0.76	130	0.1231	0.0003	0.0019
PWY.5857.ubiquinol.10biosynthesis.earlydecarboxylation.	Neisseria_flavescens	site	oral	0.76	130	0.1231	0.0003	0.0019
PWY.6708.ubiquinol.8biosynthesis.earlydecarboxylation.	Neisseria_flavescens	site	oral	0.76	130	0.1231	0.0003	0.0019
PWY.6386.UDP.N.acetylmuramoyl.pentapeptidebiosynthesisII.lysine.contain	Neisseria_flavescens	site	oral	0.95	130	0.1077	0.0007	0.0039
ARGSYNBSUB.PWY.L.argininebiosynthesisII.acetylcycle.	Neisseria_flavescens	site	oral	0.74	130	0.1077	0.0013	0.0065
UDPNAGSYN.PWY.UDP.N.acetyl.D.glucosaminebiosynthesisI	Neisseria_macacae	site	oral	0.66	130	0.1077	0.0016	0.0077
PWY.7111.pyruvatefermentationtoisobutanol.engineered.	Neisseria_macacae	site	oral	0.55	130	0.1077	0.0018	0.0084
PWY.6151.S.adenosyl.L.methioninesalvageI	Neisseria_mucosa	site	oral	1.07	130	0.1769	0.0000	0.0003
PWY.7111.pyruvatefermentationtoisobutanol.engineered.	Neisseria_mucosa	site	oral	1.25	130	0.1769	0.0000	0.0004
PWY.6386.UDP.N.acetylmuramoyl.pentapeptidebiosynthesisII.lysine.contain	Neisseria_mucosa	site	oral	0.77	130	0.1385	0.0001	0.0006

Supplementary Table 6. (Continuation)

PWY	sps	metadata	value	coef	N	Prevalence	pval	qval
VALSYN.PWY.L.valinebiosynthesis	Neisseria_mucosa	site	oral	1.22	130	0.1385	0.0001	0.0007
PWY.7219.adenosineribonucleotidesdenovobiosynthesis	Neisseria_mucosa	site	oral	1.13	130	0.1462	0.0001	0.0007
PWY.6121.5.aminoimidazoleribonucleotidebiosynthesisI	Neisseria_mucosa	site	oral	1.02	130	0.1385	0.0001	0.0007
PWY0.1586.peptidoglycanmaturation.meso.diaminopimelatecontaining.	Neisseria_mucosa	site	oral	1.41	130	0.1769	0.0001	0.0007
UDPNAGSYN.PWY.UDP.N.acetyl.D.glucosaminebiosynthesisI	Neisseria_mucosa	site	oral	1.12	130	0.1769	0.0001	0.0008
PWY.5855.ubiquinol.7biosynthesis.earlydecarboxylation.	Neisseria_mucosa	site	oral	0.76	130	0.1231	0.0001	0.0008
PWY.5856.ubiquinol.9biosynthesis.earlydecarboxylation.	Neisseria_mucosa	site	oral	0.76	130	0.1231	0.0001	0.0008
PWY.5857.ubiquinol.10biosynthesis.earlydecarboxylation.	Neisseria_mucosa	site	oral	0.76	130	0.1231	0.0001	0.0008
PWY.6708.ubiquinol.8biosynthesis.earlydecarboxylation.	Neisseria_mucosa	site	oral	0.76	130	0.1231	0.0001	0.0008
PWY.6936.seleno.aminoacidbiosynthesis.plants.	Neisseria_mucosa	site	oral	0.94	130	0.1308	0.0001	0.0010
PWY.6124.inosine.5..phosphatebiosynthesisII	Neisseria_mucosa	site	oral	0.79	130	0.1231	0.0001	0.0011
PWY.6123.inosine.5..phosphatebiosynthesisI	Neisseria_mucosa	site	oral	0.81	130	0.1462	0.0001	0.0012
PWY.6122.5.aminoimidazoleribonucleotidebiosynthesisII	Neisseria_mucosa	site	oral	1.14	130	0.1538	0.0002	0.0013
PWY.6277.superpathwayof5.aminoimidazoleribonucleotidebiosynthesis	Neisseria_mucosa	site	oral	1.14	130	0.1538	0.0002	0.0013
PWY.6387.UDP.N.acetylmuramoyl.pentapeptidebiosynthesisI.meso.diamino	Neisseria_mucosa	site	oral	0.80	130	0.1615	0.0003	0.0018
PWY.6385.peptidoglycanbiosynthesisIII.mycobacteria.	Neisseria_mucosa	site	oral	0.80	130	0.1615	0.0003	0.0020
PWY.7221.guanosineribonucleotidesdenovobiosynthesis	Neisseria_mucosa	site	oral	0.84	130	0.1231	0.0003	0.0022
PWY.6700.queuosinebiosynthesisI.denovo.	Neisseria_mucosa	site	oral	0.65	130	0.1462	0.0009	0.0047
PEPTIDOGLYCANSYN.PWY.peptidoglycanbiosynthesisI.meso.diaminopimelat	Neisseria_mucosa	site	oral	0.68	130	0.1538	0.0009	0.0048
GLUTORN.PWY.L.ornithinebiosynthesisI	Neisseria_mucosa	site	oral	0.82	130	0.1231	0.0011	0.0058
NAGLIPASYN.PWY.lipidIVAbiosynthesis.E.coli.	Neisseria_mucosa	site	oral	0.70	130	0.1385	0.0013	0.0065
ARGSYNBSUB.PWY.L.argininebiosynthesisII.acetylcycle.	Neisseria_mucosa	site	oral	0.51	130	0.1385	0.0020	0.0091
PWY0.1479.tRNAprocessing	Neisseria_mucosa	site	oral	0.46	130	0.1077	0.0020	0.0092
NONOXIPENT.PWY.pentosephosphatepathway.non.oxidativebranch.I	Neisseria_mucosa	site	oral	0.66	130	0.1385	0.0022	0.0096
PWY.3841.folatetransformationsII.plants.	Neisseria_mucosa	site	oral	0.55	130	0.1231	0.0024	0.0104
PWY.4242	Neisseria_mucosa	site	oral	0.58	130	0.1154	0.0026	0.0111
PWY.7220.adenosinedeoxyribonucleotidesdenovobiosynthesisII	Neisseria_mucosa	site	oral	0.50	130	0.1308	0.0032	0.0132
PWY.7222.guanosinedeoxyribonucleotidesdenovobiosynthesisII	Neisseria_mucosa	site	oral	0.50	130	0.1308	0.0032	0.0132
DAPLYSINESYN.PWY.L.lysinebiosynthesisI	Neisseria_mucosa	site	oral	0.52	130	0.1308	0.0033	0.0135
UDPNAGSYN.PWY.UDP.N.acetyl.D.glucosaminebiosynthesisI	Neisseria_sicca	site	oral	1.85	130	0.2385	0.0000	0.0000
PWY.6151.S.adenosyl.L.methioninesalvageI	Neisseria_sicca	site	oral	1.35	130	0.2154	0.0000	0.0002
PWY.7219.adenosineribonucleotidesdenovobiosynthesis	Neisseria_sicca	site	oral	1.07	130	0.2231	0.0000	0.0003
PWY.7111.pyruvatefermentationtoisobutanol.engineered.	Neisseria_sicca	site	oral	1.36	130	0.2231	0.0001	0.0006
PWY.6124.inosine.5..phosphatebiosynthesisII	Neisseria_sicca	site	oral	1.18	130	0.1692	0.0001	0.0011
PWY.6387.UDP.N.acetylmuramoyl.pentapeptidebiosynthesisI.meso.diamino	Neisseria_sicca	site	oral	0.88	130	0.1692	0.0002	0.0013
PEPTIDOGLYCANSYN.PWY.peptidoglycanbiosynthesisI.meso.diaminopimelat	Neisseria_sicca	site	oral	0.90	130	0.1692	0.0002	0.0013
PWY.6936.seleno.aminoacidbiosynthesis.plants.	Neisseria_sicca	site	oral	1.01	130	0.1538	0.0002	0.0017
PWY.6123.inosine.5..phosphatebiosynthesisI	Neisseria_sicca	site	oral	1.18	130	0.2000	0.0003	0.0019
PWY.6385.peptidoglycanbiosynthesisIII.mycobacteria.	Neisseria_sicca	site	oral	0.86	130	0.1615	0.0003	0.0021
ARGSYNBSUB.PWY.L.argininebiosynthesisII.acetylcycle.	Neisseria_sicca	site	oral	0.86	130	0.1615	0.0003	0.0021
PWY.6121.5.aminoimidazoleribonucleotidebiosynthesisI	Neisseria_sicca	site	oral	0.78	130	0.1692	0.0004	0.0025
PWY.6122.5.aminoimidazoleribonucleotidebiosynthesisII	Neisseria_sicca	site	oral	0.89	130	0.1923	0.0004	0.0026
PWY.6277.superpathwayof5.aminoimidazoleribonucleotidebiosynthesis	Neisseria_sicca	site	oral	0.89	130	0.1923	0.0004	0.0026
PWY0.1586.peptidoglycanmaturation.meso.diaminopimelatecontaining.	Neisseria_sicca	site	oral	0.83	130	0.1769	0.0005	0.0031
NONOXIPENT.PWY.pentosephosphatepathway.non.oxidativebranch.I	Neisseria_sicca	site	oral	0.91	130	0.1538	0.0007	0.0037
PWY.6700.queuosinebiosynthesisI.denovo.	Neisseria_sicca	site	oral	0.81	130	0.1769	0.0008	0.0043
PWY.7220.adenosinedeoxyribonucleotidesdenovobiosynthesisII	Neisseria_sicca	site	oral	1.00	130	0.1615	0.0008	0.0045
PWY.7222.guanosinedeoxyribonucleotidesdenovobiosynthesisII	Neisseria_sicca	site	oral	1.00	130	0.1615	0.0008	0.0045
VALSYN.PWY.L.valinebiosynthesis	Neisseria_sicca	site	oral	0.96	130	0.1769	0.0009	0.0047
PWY.6386.UDP.N.acetylmuramoyl.pentapeptidebiosynthesisII.lysine.contain	Neisseria_sicca	site	oral	0.86	130	0.1385	0.0029	0.0122
PWY.7221.guanosineribonucleotidesdenovobiosynthesis	Neisseria_sicca	site	oral	0.74	130	0.1308	0.0030	0.0127
GLUTORN.PWY.L.ornithinebiosynthesisI	Neisseria_sicca	site	oral	0.67	130	0.1231	0.0030	0.0127
PWY.7219.adenosineribonucleotidesdenovobiosynthesis	Neisseria_sp_oral_taxon_014	site	oral	0.80	130	0.1154	0.0013	0.0065
PWY.7111.pyruvatefermentationtoisobutanol.engineered.	Neisseria_sp_oral_taxon_014	site	oral	0.53	130	0.1077	0.0050	0.0198
PWY.7219.adenosineribonucleotidesdenovobiosynthesis	Prevotella_melaninogenica	site	oral	1.30	130	0.2000	0.0000	0.0003
PEPTIDOGLYCANSYN.PWY.peptidoglycanbiosynthesisI.meso.diaminopimelat	Prevotella_melaninogenica	site	oral	0.86	130	0.1154	0.0002	0.0013
PWY.7221.guanosineribonucleotidesdenovobiosynthesis	Prevotella_melaninogenica	site	oral	0.76	130	0.1154	0.0002	0.0014
PWY.1042.glycolysisIV	Prevotella_melaninogenica	site	oral	0.73	130	0.1231	0.0003	0.0019
PWY.3841.folatetransformationsII.plants.	Prevotella_melaninogenica	site	oral	0.70	130	0.1308	0.0004	0.0023
PWY.6151.S.adenosyl.L.methioninesalvageI	Prevotella_melaninogenica	site	oral	0.69	130	0.1077	0.0004	0.0026
PWY.6385.peptidoglycanbiosynthesisIII.mycobacteria.	Prevotella_melaninogenica	site	oral	0.73	130	0.1077	0.0010	0.0050
PWY.6387.UDP.N.acetylmuramoyl.pentapeptidebiosynthesisI.meso.diamino	Prevotella_melaninogenica	site	oral	0.75	130	0.1308	0.0011	0.0056
PWY.7199.pyrimidinedeoxyribonucleosidessalvage	Prevotella_melaninogenica	site	oral	0.60	130	0.1231	0.0016	0.0077
PWY.7219.adenosineribonucleotidesdenovobiosynthesis	Rothia_aeria	site	oral	0.89	130	0.1538	0.0001	0.0011
PWY.7111.pyruvatefermentationtoisobutanol.engineered.	Rothia_aeria	site	oral	1.00	130	0.1308	0.0004	0.0025
PWY.7219.adenosineribonucleotidesdenovobiosynthesis	Rothia_dentocariosa	site	oral	2.08	130	0.1846	0.0000	0.0001
PWY.7111.pyruvatefermentationtoisobutanol.engineered.	Rothia_dentocariosa	site	oral	1.48	130	0.1692	0.0000	0.0004
PWY.6151.S.adenosyl.L.methioninesalvageI	Rothia_dentocariosa	site	oral	0.84	130	0.1154	0.0008	0.0045
PWY.3841.folatetransformationsII.plants.	Rothia_dentocariosa	site	oral	0.67	130	0.1154	0.0009	0.0047
PWY.6122.5.aminoimidazoleribonucleotidebiosynthesisII	Rothia_dentocariosa	site	oral	1.01	130	0.1154	0.0012	0.0059
PWY.6277.superpathwayof5.aminoimidazoleribonucleotidebiosynthesis	Rothia_dentocariosa	site	oral	1.01	130	0.1154	0.0012	0.0059
VALSYN.PWY.L.valinebiosynthesis	Rothia_dentocariosa	site	oral	0.70	130	0.1154	0.0014	0.0068
UDPNAGSYN.PWY.UDP.N.acetyl.D.glucosaminebiosynthesisI	Rothia_dentocariosa	site	oral	0.67	130	0.1077	0.0014	0.0068
PWY.6121.5.aminoimidazoleribonucleotidebiosynthesisI	Rothia_dentocariosa	site	oral	0.90	130	0.1154	0.0018	0.0082
PWY.5103.L.isoleucinebiosynthesisIII	Rothia_dentocariosa	site	oral	0.65	130	0.1077	0.0019	0.0087
BRANCHED.CHAIN.AA.SYN.PWY.superpathwayofbranchedchainaminoacidbi	Rothia_dentocariosa	site	oral	0.64	130	0.1077	0.0019	0.0088
ILEUSYN.PWY.L.isoleucinebiosynthesisI.fromthreonine.	Rothia_dentocariosa	site	oral	0.63	130	0.1077	0.0021	0.0094
PWY.6123.inosine.5..phosphatebiosynthesisI	Rothia_dentocariosa	site	oral	0.64	130	0.1154	0.0036	0.0148

Supplementary Table 6. (Continuation)

PWY	sps	metadata	value	coef	N	Prevalence	pval	qval
PWY.6124.inosine.5..phosphatebiosynthesisII	Rothia_dentocariosa	site	oral	0.64	130	0.1154	0.0053	0.0207
PWY.7219.adenosineribonucleotidesdenovobiosynthesis	Rothia_mucilaginosa	site	oral	2.17	130	0.2000	0.0000	0.0001
PWY.7111.pyruvatefermentationtoisobutanol.engineered.	Rothia_mucilaginosa	site	oral	1.02	130	0.1385	0.0005	0.0030
PWY.6122.5.aminoimidazoleribonucleotidebiosynthesisII	Rothia_mucilaginosa	site	oral	0.71	130	0.1077	0.0041	0.0166
PWY.6277.superpathwayof5.aminoimidazoleribonucleotidebiosynthesis	Rothia_mucilaginosa	site	oral	0.71	130	0.1077	0.0041	0.0166
PWY.6124.inosine.5..phosphatebiosynthesisII	Streptococcus_cristatus	site	oral	0.77	130	0.1308	0.0000	0.0004
PWY.6123.inosine.5..phosphatebiosynthesisI	Streptococcus_cristatus	site	oral	0.62	130	0.1385	0.0002	0.0013
ANAGLYCOLYSIS.PWY.glycolysisIII.fromglucose.	Streptococcus_cristatus	site	oral	0.45	130	0.1077	0.0013	0.0063
PWY.7219.adenosineribonucleotidesdenovobiosynthesis	Streptococcus_cristatus	site	oral	0.53	130	0.1077	0.0031	0.0131
PWY.6121.5.aminoimidazoleribonucleotidebiosynthesisI	Streptococcus_cristatus	site	oral	0.29	130	0.1231	0.0067	0.0257
PWY.6122.5.aminoimidazoleribonucleotidebiosynthesisII	Streptococcus_cristatus	site	oral	0.44	130	0.1462	0.0098	0.0357
PWY.6277.superpathwayof5.aminoimidazoleribonucleotidebiosynthesis	Streptococcus_cristatus	site	oral	0.44	130	0.1462	0.0098	0.0357
VALSYN.PWY.L.valinebiosynthesis	Streptococcus_gordonii	site	oral	1.65	130	0.1923	0.0000	0.0000
PWY.7111.pyruvatefermentationtoisobutanol.engineered.	Streptococcus_gordonii	site	oral	0.88	130	0.1769	0.0000	0.0002
PWY.7219.adenosineribonucleotidesdenovobiosynthesis	Streptococcus_gordonii	site	oral	0.88	130	0.1231	0.0001	0.0006
PWY.6123.inosine.5..phosphatebiosynthesisI	Streptococcus_gordonii	site	oral	1.25	130	0.1385	0.0001	0.0010
BRANCHED.CHAIN.AA.SYN.PWY.superpathwayofbranchedchainaminoacidbi	Streptococcus_gordonii	site	oral	0.63	130	0.1462	0.0001	0.0011
PWY.5103.L.isoleucinebiosynthesisIII	Streptococcus_gordonii	site	oral	0.61	130	0.1462	0.0001	0.0012
ILEUSYN.PWY.L.isoleucinebiosynthesisI.fromthreonine.	Streptococcus_gordonii	site	oral	0.59	130	0.1462	0.0002	0.0012
ANAGLYCOLYSIS.PWY.glycolysisIII.fromglucose.	Streptococcus_gordonii	site	oral	0.67	130	0.1231	0.0003	0.0018
PWY.1042.glycolysisIV	Streptococcus_gordonii	site	oral	0.63	130	0.1154	0.0007	0.0040
PWY.7219.adenosineribonucleotidesdenovobiosynthesis	Streptococcus_infantis	site	oral	1.79	130	0.2615	0.0000	0.0000
PWY.7111.pyruvatefermentationtoisobutanol.engineered.	Streptococcus_infantis	site	oral	1.67	130	0.2462	0.0000	0.0000
PWY.6122.5.aminoimidazoleribonucleotidebiosynthesisII	Streptococcus_infantis	site	oral	1.43	130	0.1923	0.0000	0.0000
PWY.6277.superpathwayof5.aminoimidazoleribonucleotidebiosynthesis	Streptococcus_infantis	site	oral	1.43	130	0.1923	0.0000	0.0000
VALSYN.PWY.L.valinebiosynthesis	Streptococcus_infantis	site	oral	1.48	130	0.2385	0.0000	0.0000
PWY.6737.starchdegradationV	Streptococcus_infantis	site	oral	1.43	130	0.2231	0.0000	0.0001
ILEUSYN.PWY.L.isoleucinebiosynthesisI.fromthreonine.	Streptococcus_infantis	site	oral	1.35	130	0.1923	0.0000	0.0001
PWY.6121.5.aminoimidazoleribonucleotidebiosynthesisI	Streptococcus_infantis	site	oral	1.24	130	0.1769	0.0000	0.0001
ANAGLYCOLYSIS.PWY.glycolysisIII.fromglucose.	Streptococcus_infantis	site	oral	1.26	130	0.1769	0.0000	0.0001
PWY.7221.guanosineribonucleotidesdenovobiosynthesis	Streptococcus_infantis	site	oral	0.99	130	0.2000	0.0000	0.0002
PWY.6700.queuosinebiosynthesisI.denovo.	Streptococcus_infantis	site	oral	1.35	130	0.2077	0.0000	0.0002
BRANCHED.CHAIN.AA.SYN.PWY.superpathwayofbranchedchainaminoacidbi	Streptococcus_infantis	site	oral	1.15	130	0.1846	0.0000	0.0003
PWY.5103.L.isoleucinebiosynthesisIII	Streptococcus_infantis	site	oral	1.15	130	0.1846	0.0000	0.0003
PWY.6936.seleno.aminoacidbiosynthesis.plants.	Streptococcus_infantis	site	oral	1.08	130	0.1846	0.0000	0.0003
UDPNAGSYN.PWY.UDP.N.acetyl.D.glucosaminebiosynthesisI	Streptococcus_infantis	site	oral	0.92	130	0.1077	0.0000	0.0005
PWY.6123.inosine.5..phosphatebiosynthesisI	Streptococcus_infantis	site	oral	1.05	130	0.2000	0.0001	0.0007
PWY.7220.adenosinedeoxyribonucleotidesdenovobiosynthesisII	Streptococcus_infantis	site	oral	0.76	130	0.1538	0.0003	0.0022
PWY.7222.guanosinedeoxyribonucleotidesdenovobiosynthesisII	Streptococcus_infantis	site	oral	0.76	130	0.1538	0.0003	0.0022
PWY.1042.glycolysisIV	Streptococcus_infantis	site	oral	0.60	130	0.1154	0.0003	0.0022
PWY.6151.S.adenosyl.L.methioninesalvageI	Streptococcus_infantis	site	oral	0.80	130	0.1308	0.0004	0.0025
NONOXIPENT.PWY.pentosephosphatepathway.non.oxidativebranch.I	Streptococcus_infantis	site	oral	0.63	130	0.1385	0.0010	0.0053
PWY.6124.inosine.5..phosphatebiosynthesisII	Streptococcus_infantis	site	oral	0.65	130	0.1077	0.0031	0.0130
PWY.7219.adenosineribonucleotidesdenovobiosynthesis	Streptococcus_mitis	site	oral	2.93	130	0.4154	0.0000	0.0000
PWY.7221.guanosineribonucleotidesdenovobiosynthesis	Streptococcus_mitis	site	oral	4.87	130	0.4538	0.0000	0.0000
VALSYN.PWY.L.valinebiosynthesis	Streptococcus_mitis	site	oral	4.59	130	0.4308	0.0000	0.0000
PWY.6122.5.aminoimidazoleribonucleotidebiosynthesisII	Streptococcus_mitis	site	oral	4.72	130	0.3923	0.0000	0.0000
PWY.6277.superpathwayof5.aminoimidazoleribonucleotidebiosynthesis	Streptococcus_mitis	site	oral	4.72	130	0.3923	0.0000	0.0000
PWY.7111.pyruvatefermentationtoisobutanol.engineered.	Streptococcus_mitis	site	oral	4.69	130	0.4308	0.0000	0.0000
NONOXIPENT.PWY.pentosephosphatepathway.non.oxidativebranch.I	Streptococcus_mitis	site	oral	2.79	130	0.3846	0.0000	0.0000
PWY.6737.starchdegradationV	Streptococcus_mitis	site	oral	2.74	130	0.3846	0.0000	0.0000
PWY.6121.5.aminoimidazoleribonucleotidebiosynthesisI	Streptococcus_mitis	site	oral	4.30	130	0.3538	0.0000	0.0000
PWY.6151.S.adenosyl.L.methioninesalvageI	Streptococcus_mitis	site	oral	2.77	130	0.3538	0.0000	0.0000
PWY.1042.glycolysisIV	Streptococcus_mitis	site	oral	2.41	130	0.3615	0.0000	0.0000
PWY.6386.UDP.N.acetylmuramoyl.pentapeptidebiosynthesisII.lysine.contain	Streptococcus_mitis	site	oral	2.88	130	0.3769	0.0000	0.0000
ILEUSYN.PWY.L.isoleucinebiosynthesisI.fromthreonine.	Streptococcus_mitis	site	oral	4.01	130	0.3769	0.0000	0.0000
BRANCHED.CHAIN.AA.SYN.PWY.superpathwayofbranchedchainaminoacidbi	Streptococcus_mitis	site	oral	3.95	130	0.3769	0.0000	0.0000
PWY.5103.L.isoleucinebiosynthesisIII	Streptococcus_mitis	site	oral	4.00	130	0.3769	0.0000	0.0000
PWY.6123.inosine.5..phosphatebiosynthesisI	Streptococcus_mitis	site	oral	2.74	130	0.3692	0.0000	0.0000
ANAGLYCOLYSIS.PWY.glycolysisIII.fromglucose.	Streptococcus_mitis	site	oral	2.47	130	0.3615	0.0000	0.0000
UDPNAGSYN.PWY.UDP.N.acetyl.D.glucosaminebiosynthesisI	Streptococcus_mitis	site	oral	2.58	130	0.3308	0.0000	0.0000
PWY.6387.UDP.N.acetylmuramoyl.pentapeptidebiosynthesisI.meso.diamino	Streptococcus_mitis	site	oral	2.06	130	0.3308	0.0000	0.0000
PWY.7357.thiaminephosphateformationfrompyrithiamineandoxythiamine.y	Streptococcus_mitis	site	oral	2.64	130	0.3692	0.0000	0.0000
PWY.6936.seleno.aminoacidbiosynthesis.plants.	Streptococcus_mitis	site	oral	2.44	130	0.3385	0.0000	0.0000
PWY.6124.inosine.5..phosphatebiosynthesisII	Streptococcus_mitis	site	oral	2.80	130	0.3692	0.0000	0.0000
PWY0.1586.peptidoglycanmaturation.meso.diaminopimelatecontaining.	Streptococcus_mitis	site	oral	2.51	130	0.3308	0.0000	0.0000
PEPTIDOGLYCANSYN.PWY.peptidoglycanbiosynthesisI.meso.diaminopimelat	Streptococcus_mitis	site	oral	2.03	130	0.3231	0.0000	0.0000
PWY.7220.adenosinedeoxyribonucleotidesdenovobiosynthesisII	Streptococcus_mitis	site	oral	3.32	130	0.3769	0.0000	0.0000
PWY.7222.guanosinedeoxyribonucleotidesdenovobiosynthesisII	Streptococcus_mitis	site	oral	3.32	130	0.3769	0.0000	0.0000
PWY.7199.pyrimidinedeoxyribonucleosidessalvage	Streptococcus_mitis	site	oral	2.49	130	0.3385	0.0000	0.0000
PWY0.1298.superpathwayofpyrimidinedeoxyribonucleosidesdegradation	Streptococcus_mitis	site	oral	2.40	130	0.3154	0.0000	0.0000
PWY.6385.peptidoglycanbiosynthesisIII.mycobacteria.	Streptococcus_mitis	site	oral	1.94	130	0.3154	0.0000	0.0000
PWY.6609.adenineandadenosinesalvageIII	Streptococcus_mitis	site	oral	2.27	130	0.3769	0.0000	0.0000
PWY.3841.folatetransformationsII.plants.	Streptococcus_mitis	site	oral	2.32	130	0.3385	0.0000	0.0000
PWY.6700.queuosinebiosynthesisI.denovo.	Streptococcus_mitis	site	oral	2.17	130	0.3308	0.0000	0.0000
PWY.6317.D.galactosedegradationI.Leloirpathway.	Streptococcus_mitis	site	oral	1.61	130	0.3077	0.0000	0.0000
PWY.6897.thiaminediphosphatesalvageII	Streptococcus_mitis	site	oral	1.48	130	0.2846	0.0000	0.0000
PWY.5695.inosine5..phosphatedegradation	Streptococcus_mitis	site	oral	0.66	130	0.1385	0.0012	0.0059

Supplementary Table 6. (Continuation)

PWY	sps	metadata	value	coef	N	Prevalence	pval	qval
HSERMETANA.PWY.L.methioninebiosynthesisIII	Streptococcus_mitis	site	oral	0.68	130	0.1846	0.0045	0.0178
PWY.6122.5.aminoimidazoleribonucleotidebiosynthesisII	Streptococcus_oralis	site	oral	3.39	130	0.3923	0.0000	0.0000
PWY.6277.superpathwayof5.aminoimidazoleribonucleotidebiosynthesis	Streptococcus_oralis	site	oral	3.39	130	0.3923	0.0000	0.0000
PWY.7111.pyruvatefermentationtoisobutanol.engineered.	Streptococcus_oralis	site	oral	3.93	130	0.4154	0.0000	0.0000
PWY.6609.adenineandadenosinesalvageIII	Streptococcus_oralis	site	oral	3.24	130	0.3769	0.0000	0.0000
NONOXIPENT.PWY.pentosephosphatepathway.non.oxidativebranch.I	Streptococcus_oralis	site	oral	2.66	130	0.3615	0.0000	0.0000
PWY.7221.guanosineribonucleotidesdenovobiosynthesis	Streptococcus_oralis	site	oral	3.39	130	0.3923	0.0000	0.0000
ANAGLYCOLYSIS.PWY.glycolysisIII.fromglucose.	Streptococcus_oralis	site	oral	2.87	130	0.3308	0.0000	0.0000
PWY.7357.thiaminephosphateformationfrompyrithiamineandoxythiamine.y	Streptococcus_oralis	site	oral	1.87	130	0.3385	0.0000	0.0000
PWY.1042.glycolysisIV	Streptococcus_oralis	site	oral	2.69	130	0.3154	0.0000	0.0000
VALSYN.PWY.L.valinebiosynthesis	Streptococcus_oralis	site	oral	3.07	130	0.4154	0.0000	0.0000
PWY.6123.inosine.5..phosphatebiosynthesisI	Streptococcus_oralis	site	oral	2.83	130	0.3462	0.0000	0.0000
PWY0.1586.peptidoglycanmaturation.meso.diaminopimelatecontaining.	Streptococcus_oralis	site	oral	2.27	130	0.3692	0.0000	0.0000
PWY.6151.S.adenosyl.L.methioninesalvageI	Streptococcus_oralis	site	oral	2.63	130	0.3769	0.0000	0.0000
PWY.6387.UDP.N.acetylmuramoyl.pentapeptidebiosynthesisI.meso.diamino	Streptococcus_oralis	site	oral	1.42	130	0.2769	0.0000	0.0000
PWY.6386.UDP.N.acetylmuramoyl.pentapeptidebiosynthesisII.lysine.contain	Streptococcus_oralis	site	oral	1.58	130	0.3000	0.0000	0.0000
PWY.7220.adenosinedeoxyribonucleotidesdenovobiosynthesisII	Streptococcus_oralis	site	oral	2.22	130	0.3308	0.0000	0.0000
PWY.7222.guanosinedeoxyribonucleotidesdenovobiosynthesisII	Streptococcus_oralis	site	oral	2.22	130	0.3308	0.0000	0.0000
PWY.6124.inosine.5..phosphatebiosynthesisII	Streptococcus_oralis	site	oral	2.48	130	0.3385	0.0000	0.0000
PWY.7219.adenosineribonucleotidesdenovobiosynthesis	Streptococcus_oralis	site	oral	1.55	130	0.3308	0.0000	0.0000
BRANCHED.CHAIN.AA.SYN.PWY.superpathwayofbranchedchainaminoacidbi	Streptococcus_oralis	site	oral	2.75	130	0.3462	0.0000	0.0000
PWY.5103.L.isoleucinebiosynthesisIII	Streptococcus_oralis	site	oral	2.77	130	0.3462	0.0000	0.0000
ILEUSYN.PWY.L.isoleucinebiosynthesisI.fromthreonine.	Streptococcus_oralis	site	oral	2.67	130	0.3462	0.0000	0.0000
PWY.6897.thiaminediphosphatesalvageII	Streptococcus_oralis	site	oral	1.64	130	0.2462	0.0000	0.0000
PWY.6385.peptidoglycanbiosynthesisIII.mycobacteria.	Streptococcus_oralis	site	oral	1.22	130	0.2385	0.0000	0.0000
PEPTIDOGLYCANSYN.PWY.peptidoglycanbiosynthesisI.meso.diaminopimelat	Streptococcus_oralis	site	oral	1.11	130	0.2462	0.0000	0.0000
PWY.6936.seleno.aminoacidbiosynthesis.plants.	Streptococcus_oralis	site	oral	1.44	130	0.2538	0.0000	0.0000
PWY.7199.pyrimidinedeoxyribonucleosidessalvage	Streptococcus_oralis	site	oral	1.34	130	0.2462	0.0000	0.0001
PWY.6121.5.aminoimidazoleribonucleotidebiosynthesisI	Streptococcus_oralis	site	oral	1.58	130	0.2692	0.0000	0.0001
PWY.6700.queuosinebiosynthesisI.denovo.	Streptococcus_oralis	site	oral	1.29	130	0.2692	0.0000	0.0001
PWY0.1298.superpathwayofpyrimidinedeoxyribonucleosidesdegradation	Streptococcus_oralis	site	oral	0.87	130	0.2000	0.0000	0.0005
UDPNAGSYN.PWY.UDP.N.acetyl.D.glucosaminebiosynthesisI	Streptococcus_oralis	site	oral	1.16	130	0.2462	0.0000	0.0005
PWY.3841.folatetransformationsII.plants.	Streptococcus_oralis	site	oral	0.95	130	0.2385	0.0001	0.0009
HSERMETANA.PWY.L.methioninebiosynthesisIII	Streptococcus_oralis	site	oral	0.66	130	0.1385	0.0020	0.0091
PWY.5695.inosine5..phosphatedegradation	Streptococcus_oralis	site	oral	0.98	130	0.1923	0.0057	0.0221
PWY.6122.5.aminoimidazoleribonucleotidebiosynthesisII	Streptococcus_parasanguinis	site	oral	1.36	130	0.1615	0.0002	0.0012
PWY.6277.superpathwayof5.aminoimidazoleribonucleotidebiosynthesis	Streptococcus_parasanguinis	site	oral	1.36	130	0.1615	0.0002	0.0012
PWY.7111.pyruvatefermentationtoisobutanol.engineered.	Streptococcus_parasanguinis	site	oral	1.21	130	0.1769	0.0002	0.0017
PWY.7219.adenosineribonucleotidesdenovobiosynthesis	Streptococcus_parasanguinis	site	oral	1.04	130	0.1615	0.0002	0.0017
PWY.6123.inosine.5..phosphatebiosynthesisI	Streptococcus_parasanguinis	site	oral	1.04	130	0.1462	0.0004	0.0023
VALSYN.PWY.L.valinebiosynthesis	Streptococcus_parasanguinis	site	oral	1.07	130	0.1462	0.0008	0.0045
PWY.6124.inosine.5..phosphatebiosynthesisII	Streptococcus_parasanguinis	site	oral	1.00	130	0.1308	0.0009	0.0046
PWY.6121.5.aminoimidazoleribonucleotidebiosynthesisI	Streptococcus_parasanguinis	site	oral	1.07	130	0.1308	0.0009	0.0047
PWY.1042.glycolysisIV	Streptococcus_parasanguinis	site	oral	0.75	130	0.1462	0.0015	0.0071
ANAGLYCOLYSIS.PWY.glycolysisIII.fromglucose.	Streptococcus_parasanguinis	site	oral	0.60	130	0.1077	0.0051	0.0199
ILEUSYN.PWY.L.isoleucinebiosynthesisI.fromthreonine.	Streptococcus_parasanguinis	site	oral	0.81	130	0.1154	0.0085	0.0315
BRANCHED.CHAIN.AA.SYN.PWY.superpathwayofbranchedchainaminoacidbi	Streptococcus_parasanguinis	site	oral	0.58	130	0.1077	0.0123	0.0439
PWY.5103.L.isoleucinebiosynthesisIII	Streptococcus_parasanguinis	site	oral	0.59	130	0.1077	0.0123	0.0440
PWY.6897.thiaminediphosphatesalvageII	Streptococcus_pseudopneumoniae	site	oral	0.65	130	0.1077	0.0136	0.0480
PWY.7111.pyruvatefermentationtoisobutanol.engineered.	Streptococcus_sanguinis	site	oral	1.05	130	0.1615	0.0005	0.0028
PWY.7219.adenosineribonucleotidesdenovobiosynthesis	Streptococcus_sanguinis	site	oral	0.56	130	0.1154	0.0005	0.0030
PWY.1042.glycolysisIV	Streptococcus_sanguinis	site	oral	0.67	130	0.1077	0.0010	0.0053
PWY.7219.adenosineribonucleotidesdenovobiosynthesis	Veillonella_atypica	site	oral	0.58	130	0.1077	0.0090	0.0331
PWY.7219.adenosineribonucleotidesdenovobiosynthesis	Veillonella_parvula	site	oral	1.00	130	0.1462	0.0001	0.0011
VALSYN.PWY.L.valinebiosynthesis	Veillonella_parvula	site	oral	0.76	130	0.1077	0.0058	0.0224
PWY.7111.pyruvatefermentationtoisobutanol.engineered.	Veillonella_parvula	site	oral	0.71	130	0.1077	0.0072	0.0272
PWY.7219.adenosineribonucleotidesdenovobiosynthesis	Bacteroides_fragilis	site	oral	-1.18	130	0.1077	0.0053	0.0206
PWY.6123.inosine.5..phosphatebiosynthesisI	Bacteroides_vulgatus	site	oral	-0.58	130	0.1154	0.0125	0.0445
PWY.6124.inosine.5..phosphatebiosynthesisII	Bacteroides_vulgatus	site	oral	-0.86	130	0.1308	0.0042	0.0167
PWY.6700.queuosinebiosynthesisI.denovo.	Bacteroides_vulgatus	site	oral	-0.70	130	0.1154	0.0079	0.0295
PWY.7111.pyruvatefermentationtoisobutanol.engineered.	Bacteroides_vulgatus	site	oral	-0.62	130	0.1154	0.0086	0.0320
PWY.7219.adenosineribonucleotidesdenovobiosynthesis	Bacteroides_vulgatus	site	oral	-0.65	130	0.1462	0.0114	0.0411
PWY.7221.guanosineribonucleotidesdenovobiosynthesis	Bacteroides_vulgatus	site	oral	-0.91	130	0.1462	0.0074	0.0280
VALSYN.PWY.L.valinebiosynthesis	Bacteroides_vulgatus	site	oral	-0.62	130	0.1154	0.0086	0.0320
ARGSYNBSUB.PWY.L.argininebiosynthesisII.acetylcycle.	Catenibacterium_mitsuokai	site	oral	-0.64	130	0.1308	0.0039	0.0157
BRANCHED.CHAIN.AA.SYN.PWY.superpathwayofbranchedchainaminoacidbi	Catenibacterium_mitsuokai	site	oral	-0.73	130	0.1231	0.0004	0.0026
GLUTORN.PWY.L.ornithinebiosynthesisI	Catenibacterium_mitsuokai	site	oral	-0.47	130	0.1231	0.0088	0.0327
HSERMETANA.PWY.L.methioninebiosynthesisIII	Catenibacterium_mitsuokai	site	oral	-0.48	130	0.1077	0.0134	0.0472
ILEUSYN.PWY.L.isoleucinebiosynthesisI.fromthreonine.	Catenibacterium_mitsuokai	site	oral	-0.74	130	0.1231	0.0004	0.0023
NONOXIPENT.PWY.pentosephosphatepathway.non.oxidativebranch.I	Catenibacterium_mitsuokai	site	oral	-0.80	130	0.1385	0.0012	0.0061
PWY.3841.folatetransformationsII.plants.	Catenibacterium_mitsuokai	site	oral	-0.70	130	0.1231	0.0077	0.0290
PWY.5103.L.isoleucinebiosynthesisIII	Catenibacterium_mitsuokai	site	oral	-0.75	130	0.1231	0.0004	0.0026
PWY.6122.5.aminoimidazoleribonucleotidebiosynthesisII	Catenibacterium_mitsuokai	site	oral	-0.64	130	0.1077	0.0013	0.0062
PWY.6277.superpathwayof5.aminoimidazoleribonucleotidebiosynthesis	Catenibacterium_mitsuokai	site	oral	-0.64	130	0.1077	0.0013	0.0062
PWY.6317.D.galactosedegradationI.Leloirpathway.	Catenibacterium_mitsuokai	site	oral	-0.55	130	0.1077	0.0012	0.0060
PWY.6609.adenineandadenosinesalvageIII	Catenibacterium_mitsuokai	site	oral	-0.76	130	0.1308	0.0001	0.0012
PWY.6700.queuosinebiosynthesisI.denovo.	Catenibacterium_mitsuokai	site	oral	-0.91	130	0.1462	0.0009	0.0047
PWY.6737.starchdegradationV	Catenibacterium_mitsuokai	site	oral	-0.87	130	0.1385	0.0016	0.0075

Supplementary Table 6. (Continuation)

PWY	sps	metadata	value	coef	N	Prevalence	pval	qval
PWY.7111.pyruvatefermentationtoisobutanol.engineered.	Catenibacterium_mitsuokai	site	oral	-0.84	130	0.1538	0.0001	0.0007
PWY.7219.adenosineribonucleotidesdenovobiosynthesis	Catenibacterium_mitsuokai	site	oral	-1.10	130	0.1462	0.0009	0.0047
PWY.7221.guanosineribonucleotidesdenovobiosynthesis	Catenibacterium_mitsuokai	site	oral	-0.88	130	0.1231	0.0002	0.0017
UDPNAGSYN.PWY.UDP.N.acetyl.D.glucosaminebiosynthesisI	Catenibacterium_mitsuokai	site	oral	-0.88	130	0.1308	0.0002	0.0012
VALSYN.PWY.L.valinebiosynthesis	Catenibacterium_mitsuokai	site	oral	-0.78	130	0.1385	0.0002	0.0014
BRANCHED.CHAIN.AA.SYN.PWY.superpathwayofbranchedchainaminoacidbi	Eubacterium_rectale	site	oral	-0.89	130	0.1231	0.0004	0.0022
ILEUSYN.PWY.L.isoleucinebiosynthesisI.fromthreonine.	Eubacterium_rectale	site	oral	-0.97	130	0.1231	0.0003	0.0022
NONOXIPENT.PWY.pentosephosphatepathway.non.oxidativebranch.I	Eubacterium_rectale	site	oral	-0.81	130	0.1077	0.0012	0.0060
PWY.5103.L.isoleucinebiosynthesisIII	Eubacterium_rectale	site	oral	-0.93	130	0.1231	0.0003	0.0022
PWY.6121.5.aminoimidazoleribonucleotidebiosynthesisI	Eubacterium_rectale	site	oral	-1.09	130	0.1231	0.0003	0.0020
PWY.6122.5.aminoimidazoleribonucleotidebiosynthesisII	Eubacterium_rectale	site	oral	-1.05	130	0.1308	0.0015	0.0071
PWY.6277.superpathwayof5.aminoimidazoleribonucleotidebiosynthesis	Eubacterium_rectale	site	oral	-1.05	130	0.1308	0.0015	0.0071
PWY.7111.pyruvatefermentationtoisobutanol.engineered.	Eubacterium_rectale	site	oral	-1.35	130	0.1769	0.0000	0.0003
PWY.7219.adenosineribonucleotidesdenovobiosynthesis	Eubacterium_rectale	site	oral	-1.09	130	0.1462	0.0001	0.0007
PWY.7221.guanosineribonucleotidesdenovobiosynthesis	Eubacterium_rectale	site	oral	-0.76	130	0.1231	0.0007	0.0040
PWY.7357.thiaminephosphateformationfrompyrithiamineandoxythiamine.y	Eubacterium_rectale	site	oral	-0.93	130	0.1077	0.0009	0.0049
VALSYN.PWY.L.valinebiosynthesis	Eubacterium_rectale	site	oral	-1.16	130	0.1692	0.0002	0.0017
PWY.7111.pyruvatefermentationtoisobutanol.engineered.	Eubacterium_sp_CAG_180	site	oral	-0.69	130	0.1154	0.0003	0.0020
PWY.7219.adenosineribonucleotidesdenovobiosynthesis	Eubacterium_sp_CAG_180	site	oral	-0.78	130	0.1154	0.0003	0.0018
VALSYN.PWY.L.valinebiosynthesis	Eubacterium_sp_CAG_180	site	oral	-0.69	130	0.1154	0.0003	0.0020
ARGSYNBSUB.PWY.L.argininebiosynthesisII.acetylcycle.	Faecalibacterium_prausnitzii	site	oral	-1.22	130	0.2154	0.0000	0.0000
GLUTORN.PWY.L.ornithinebiosynthesisI	Faecalibacterium_prausnitzii	site	oral	-1.18	130	0.1923	0.0000	0.0000
HISTSYN.PWY.L.histidinebiosynthesis	Faecalibacterium_prausnitzii	site	oral	-0.94	130	0.1692	0.0000	0.0002
PEPTIDOGLYCANSYN.PWY.peptidoglycanbiosynthesisI.meso.diaminopimelat	Faecalibacterium_prausnitzii	site	oral	-0.78	130	0.1231	0.0005	0.0028
PWY.1042.glycolysisIV	Faecalibacterium_prausnitzii	site	oral	-1.44	130	0.2000	0.0000	0.0000
PWY.4242	Faecalibacterium_prausnitzii	site	oral	-1.39	130	0.1692	0.0000	0.0001
PWY.6121.5.aminoimidazoleribonucleotidebiosynthesisI	Faecalibacterium_prausnitzii	site	oral	-1.21	130	0.2000	0.0000	0.0001
PWY.6122.5.aminoimidazoleribonucleotidebiosynthesisII	Faecalibacterium_prausnitzii	site	oral	-1.32	130	0.2077	0.0000	0.0000
PWY.6123.inosine.5..phosphatebiosynthesisI	Faecalibacterium_prausnitzii	site	oral	-1.19	130	0.1769	0.0000	0.0001
PWY.6124.inosine.5..phosphatebiosynthesisII	Faecalibacterium_prausnitzii	site	oral	-1.29	130	0.1846	0.0000	0.0001
PWY.6151.S.adenosyl.L.methioninesalvageI	Faecalibacterium_prausnitzii	site	oral	-1.09	130	0.1846	0.0000	0.0001
PWY.6277.superpathwayof5.aminoimidazoleribonucleotidebiosynthesis	Faecalibacterium_prausnitzii	site	oral	-1.32	130	0.2077	0.0000	0.0000
PWY.6386.UDP.N.acetylmuramoyl.pentapeptidebiosynthesisII.lysine.contain	Faecalibacterium_prausnitzii	site	oral	-0.94	130	0.1385	0.0001	0.0012
PWY.6387.UDP.N.acetylmuramoyl.pentapeptidebiosynthesisI.meso.diamino	Faecalibacterium_prausnitzii	site	oral	-0.92	130	0.1385	0.0002	0.0012
PWY.6609.adenineandadenosinesalvageIII	Faecalibacterium_prausnitzii	site	oral	-1.47	130	0.2000	0.0000	0.0001
PWY.6700.queuosinebiosynthesisI.denovo.	Faecalibacterium_prausnitzii	site	oral	-0.65	130	0.1308	0.0003	0.0018
PWY.6737.starchdegradationV	Faecalibacterium_prausnitzii	site	oral	-1.69	130	0.2462	0.0000	0.0000
PWY.6897.thiaminediphosphatesalvageII	Faecalibacterium_prausnitzii	site	oral	-1.00	130	0.1538	0.0000	0.0004
PWY.7111.pyruvatefermentationtoisobutanol.engineered.	Faecalibacterium_prausnitzii	site	oral	-2.16	130	0.2923	0.0000	0.0000
PWY.7219.adenosineribonucleotidesdenovobiosynthesis	Faecalibacterium_prausnitzii	site	oral	-1.66	130	0.2923	0.0000	0.0000
PWY.7221.guanosineribonucleotidesdenovobiosynthesis	Faecalibacterium_prausnitzii	site	oral	-1.30	130	0.2077	0.0000	0.0000
PWY.7357.thiaminephosphateformationfrompyrithiamineandoxythiamine.y	Faecalibacterium_prausnitzii	site	oral	-1.32	130	0.2077	0.0000	0.0000
VALSYN.PWY.L.valinebiosynthesis	Faecalibacterium_prausnitzii	site	oral	-1.42	130	0.2538	0.0000	0.0000
PWY.7219.adenosineribonucleotidesdenovobiosynthesis	Finegoldia_magna	site	oral	-0.66	130	0.1308	0.0095	0.0347
NONMEVIPP.PWY.methylerythritolphosphatepathwayI	Fusobacterium_gonidiaformans	site	oral	-1.28	130	0.1846	0.0000	0.0001
PEPTIDOGLYCANSYN.PWY.peptidoglycanbiosynthesisI.meso.diaminopimelat	Fusobacterium_gonidiaformans	site	oral	-1.77	130	0.2077	0.0000	0.0001
PWY.1042.glycolysisIV	Fusobacterium_gonidiaformans	site	oral	-1.55	130	0.2154	0.0000	0.0001
PWY.4242	Fusobacterium_gonidiaformans	site	oral	-1.23	130	0.1846	0.0000	0.0005
PWY.6121.5.aminoimidazoleribonucleotidebiosynthesisI	Fusobacterium_gonidiaformans	site	oral	-1.21	130	0.1846	0.0002	0.0017
PWY.6122.5.aminoimidazoleribonucleotidebiosynthesisII	Fusobacterium_gonidiaformans	site	oral	-1.36	130	0.2231	0.0000	0.0005
PWY.6123.inosine.5..phosphatebiosynthesisI	Fusobacterium_gonidiaformans	site	oral	-1.04	130	0.1538	0.0001	0.0011
PWY.6124.inosine.5..phosphatebiosynthesisII	Fusobacterium_gonidiaformans	site	oral	-1.30	130	0.1923	0.0001	0.0005
PWY.6277.superpathwayof5.aminoimidazoleribonucleotidebiosynthesis	Fusobacterium_gonidiaformans	site	oral	-1.36	130	0.2231	0.0000	0.0005
PWY.6385.peptidoglycanbiosynthesisIII.mycobacteria.	Fusobacterium_gonidiaformans	site	oral	-1.86	130	0.2077	0.0000	0.0001
PWY.6386.UDP.N.acetylmuramoyl.pentapeptidebiosynthesisII.lysine.contain	Fusobacterium_gonidiaformans	site	oral	-1.59	130	0.1846	0.0000	0.0002
PWY.6387.UDP.N.acetylmuramoyl.pentapeptidebiosynthesisI.meso.diamino	Fusobacterium_gonidiaformans	site	oral	-1.87	130	0.2154	0.0000	0.0000
PWY.6609.adenineandadenosinesalvageIII	Fusobacterium_gonidiaformans	site	oral	-1.89	130	0.2308	0.0000	0.0001
PWY.6700.queuosinebiosynthesisI.denovo.	Fusobacterium_gonidiaformans	site	oral	-1.38	130	0.2462	0.0000	0.0002
PWY.6737.starchdegradationV	Fusobacterium_gonidiaformans	site	oral	-1.20	130	0.1846	0.0001	0.0007
PWY.7219.adenosineribonucleotidesdenovobiosynthesis	Fusobacterium_gonidiaformans	site	oral	-1.34	130	0.2385	0.0000	0.0002
PWY.7221.guanosineribonucleotidesdenovobiosynthesis	Fusobacterium_gonidiaformans	site	oral	-1.24	130	0.2000	0.0001	0.0010
PWY0.1586.peptidoglycanmaturation.meso.diaminopimelatecontaining.	Fusobacterium_gonidiaformans	site	oral	-1.29	130	0.2000	0.0001	0.0007
UDPNAGSYN.PWY.UDP.N.acetyl.D.glucosaminebiosynthesisI	Fusobacterium_gonidiaformans	site	oral	-1.90	130	0.2154	0.0000	0.0001
PWY.6609.adenineandadenosinesalvageIII	Megasphaera_elsdenii	site	oral	-0.64	130	0.1154	0.0005	0.0030
PWY.7111.pyruvatefermentationtoisobutanol.engineered.	Megasphaera_elsdenii	site	oral	-0.40	130	0.1154	0.0005	0.0029
PWY.7219.adenosineribonucleotidesdenovobiosynthesis	Megasphaera_elsdenii	site	oral	-0.69	130	0.1308	0.0023	0.0102
UDPNAGSYN.PWY.UDP.N.acetyl.D.glucosaminebiosynthesisI	Megasphaera_elsdenii	site	oral	-0.52	130	0.1154	0.0052	0.0206
VALSYN.PWY.L.valinebiosynthesis	Megasphaera_elsdenii	site	oral	-0.71	130	0.1385	0.0010	0.0050
PWY.6122.5.aminoimidazoleribonucleotidebiosynthesisII	Mitsuokella_jalaludinii	site	oral	-0.41	130	0.1077	0.0142	0.0496
PWY.6277.superpathwayof5.aminoimidazoleribonucleotidebiosynthesis	Mitsuokella_jalaludinii	site	oral	-0.41	130	0.1077	0.0142	0.0496
PWY.6385.peptidoglycanbiosynthesisIII.mycobacteria.	Mitsuokella_jalaludinii	site	oral	-0.42	130	0.1077	0.0143	0.0499
PWY.6387.UDP.N.acetylmuramoyl.pentapeptidebiosynthesisI.meso.diamino	Mitsuokella_jalaludinii	site	oral	-0.54	130	0.1154	0.0086	0.0320
PWY.6700.queuosinebiosynthesisI.denovo.	Mitsuokella_jalaludinii	site	oral	-0.58	130	0.1154	0.0069	0.0264
PWY.7111.pyruvatefermentationtoisobutanol.engineered.	Mitsuokella_jalaludinii	site	oral	-0.55	130	0.1231	0.0063	0.0240
PWY.7220.adenosinedeoxyribonucleotidesdenovobiosynthesisII	Mitsuokella_jalaludinii	site	oral	-0.49	130	0.1077	0.0141	0.0494
PWY.7221.guanosineribonucleotidesdenovobiosynthesis	Mitsuokella_jalaludinii	site	oral	-0.52	130	0.1231	0.0045	0.0179
PWY.7222.guanosinedeoxyribonucleotidesdenovobiosynthesisII	Mitsuokella_jalaludinii	site	oral	-0.49	130	0.1077	0.0141	0.0494
VALSYN.PWY.L.valinebiosynthesis	Mitsuokella_jalaludinii	site	oral	-0.59	130	0.1231	0.0063	0.0241

Supplementary Table 6. (Continuation)

PWY	sps	metadata	value	coef	N	Prevalence	pval	qval
PWY.6737.starchdegradationV	Mitsuokella_multacida	site	oral	-0.51	130	0.1077	0.0117	0.0420
PWY.6122.5.aminoimidazoleribonucleotidebiosynthesisII	Oscillibacter_sp_57_20	site	oral	-0.39	130	0.1077	0.0010	0.0050
PWY.6277.superpathwayof5.aminoimidazoleribonucleotidebiosynthesis	Oscillibacter_sp_57_20	site	oral	-0.39	130	0.1077	0.0010	0.0050
PWY.6609.adenineandadenosinesalvageIII	Oscillibacter_sp_57_20	site	oral	-0.86	130	0.1538	0.0000	0.0003
PWY.7219.adenosineribonucleotidesdenovobiosynthesis	Oscillibacter_sp_CAG_241	site	oral	-0.55	130	0.1077	0.0022	0.0096
UDPNAGSYN.PWY.UDP.N.acetyl.D.glucosaminebiosynthesisI	Parvimonas_sp_KA00067	site	oral	-0.51	130	0.1077	0.0026	0.0110
PWY.7111.pyruvatefermentationtoisobutanol.engineered.	Phascolarctobacterium_succinatutens	site	oral	-0.64	130	0.1077	0.0014	0.0069
PWY.7219.adenosineribonucleotidesdenovobiosynthesis	Phascolarctobacterium_succinatutens	site	oral	-0.62	130	0.1077	0.0017	0.0079
NONOXIPENT.PWY.pentosephosphatepathway.non.oxidativebranch.I	Porphyromonas_asaccharolytica	site	oral	-1.29	130	0.1692	0.0009	0.0049
PEPTIDOGLYCANSYN.PWY.peptidoglycanbiosynthesisI.meso.diaminopimelat	Porphyromonas_asaccharolytica	site	oral	-1.01	130	0.1462	0.0034	0.0142
PWY.3841.folatetransformationsII.plants.	Porphyromonas_asaccharolytica	site	oral	-0.90	130	0.1462	0.0038	0.0153
PWY.5695.inosine5..phosphatedegradation	Porphyromonas_asaccharolytica	site	oral	-1.47	130	0.1462	0.0003	0.0019
PWY.6122.5.aminoimidazoleribonucleotidebiosynthesisII	Porphyromonas_asaccharolytica	site	oral	-1.19	130	0.1692	0.0008	0.0043
PWY.6123.inosine.5..phosphatebiosynthesisI	Porphyromonas_asaccharolytica	site	oral	-1.08	130	0.1538	0.0027	0.0113
PWY.6124.inosine.5..phosphatebiosynthesisII	Porphyromonas_asaccharolytica	site	oral	-1.26	130	0.1692	0.0009	0.0047
PWY.6277.superpathwayof5.aminoimidazoleribonucleotidebiosynthesis	Porphyromonas_asaccharolytica	site	oral	-1.19	130	0.1692	0.0008	0.0043
PWY.6385.peptidoglycanbiosynthesisIII.mycobacteria.	Porphyromonas_asaccharolytica	site	oral	-1.30	130	0.1538	0.0021	0.0094
PWY.6386.UDP.N.acetylmuramoyl.pentapeptidebiosynthesisII.lysine.contair	Porphyromonas_asaccharolytica	site	oral	-1.28	130	0.1385	0.0011	0.0056
PWY.6387.UDP.N.acetylmuramoyl.pentapeptidebiosynthesisI.meso.diamino	Porphyromonas_asaccharolytica	site	oral	-1.32	130	0.1538	0.0020	0.0091
PWY.6609.adenineandadenosinesalvageIII	Porphyromonas_asaccharolytica	site	oral	-1.35	130	0.1615	0.0022	0.0096
PWY.6700.queuosinebiosynthesisI.denovo.	Porphyromonas_asaccharolytica	site	oral	-1.12	130	0.1231	0.0018	0.0084
PWY.7219.adenosineribonucleotidesdenovobiosynthesis	Porphyromonas_asaccharolytica	site	oral	-1.41	130	0.1769	0.0001	0.0007
PWY.7221.guanosineribonucleotidesdenovobiosynthesis	Porphyromonas_asaccharolytica	site	oral	-1.34	130	0.1462	0.0006	0.0035
NONOXIPENT.PWY.pentosephosphatepathway.non.oxidativebranch.I	Porphyromonas_uenonis	site	oral	-0.94	130	0.1385	0.0009	0.0047
PEPTIDOGLYCANSYN.PWY.peptidoglycanbiosynthesisI.meso.diaminopimelat	Porphyromonas_uenonis	site	oral	-1.25	130	0.1385	0.0002	0.0013
PWY.3841.folatetransformationsII.plants.	Porphyromonas_uenonis	site	oral	-1.07	130	0.1308	0.0003	0.0022
PWY.4242	Porphyromonas_uenonis	site	oral	-0.86	130	0.1077	0.0012	0.0060
PWY.5695.inosine5..phosphatedegradation	Porphyromonas_uenonis	site	oral	-2.01	130	0.1538	0.0000	0.0003
PWY.6121.5.aminoimidazoleribonucleotidebiosynthesisI	Porphyromonas_uenonis	site	oral	-0.82	130	0.1077	0.0009	0.0047
PWY.6122.5.aminoimidazoleribonucleotidebiosynthesisII	Porphyromonas_uenonis	site	oral	-1.11	130	0.1538	0.0003	0.0020
PWY.6123.inosine.5..phosphatebiosynthesisI	Porphyromonas_uenonis	site	oral	-1.33	130	0.1385	0.0002	0.0012
PWY.6124.inosine.5..phosphatebiosynthesisII	Porphyromonas_uenonis	site	oral	-1.37	130	0.1462	0.0001	0.0008
PWY.6151.S.adenosyl.L.methioninesalvageI	Porphyromonas_uenonis	site	oral	-0.84	130	0.1077	0.0014	0.0066
PWY.6277.superpathwayof5.aminoimidazoleribonucleotidebiosynthesis	Porphyromonas_uenonis	site	oral	-1.11	130	0.1538	0.0003	0.0020
PWY.6385.peptidoglycanbiosynthesisIII.mycobacteria.	Porphyromonas_uenonis	site	oral	-1.23	130	0.1385	0.0002	0.0013
PWY.6386.UDP.N.acetylmuramoyl.pentapeptidebiosynthesisII.lysine.contair	Porphyromonas_uenonis	site	oral	-1.35	130	0.1385	0.0001	0.0012
PWY.6387.UDP.N.acetylmuramoyl.pentapeptidebiosynthesisI.meso.diamino	Porphyromonas_uenonis	site	oral	-1.26	130	0.1385	0.0002	0.0013
PWY.6609.adenineandadenosinesalvageIII	Porphyromonas_uenonis	site	oral	-1.07	130	0.1538	0.0002	0.0017
PWY.6700.queuosinebiosynthesisI.denovo.	Porphyromonas_uenonis	site	oral	-1.07	130	0.1385	0.0002	0.0016
PWY.7219.adenosineribonucleotidesdenovobiosynthesis	Porphyromonas_uenonis	site	oral	-1.15	130	0.1462	0.0001	0.0010
PWY.7221.guanosineribonucleotidesdenovobiosynthesis	Porphyromonas_uenonis	site	oral	-1.84	130	0.1462	0.0001	0.0006
NAGLIPASYN.PWY.lipidIVAbiosynthesis.E.coli.	Prevotella_bivia	site	oral	-1.42	130	0.2000	0.0003	0.0020
NONMEVIPP.PWY.methylerythritolphosphatepathwayI	Prevotella_bivia	site	oral	-1.33	130	0.1846	0.0002	0.0015
PEPTIDOGLYCANSYN.PWY.peptidoglycanbiosynthesisI.meso.diaminopimelat	Prevotella_bivia	site	oral	-1.45	130	0.2154	0.0004	0.0023
PWY.1042.glycolysisIV	Prevotella_bivia	site	oral	-1.54	130	0.1923	0.0000	0.0005
PWY.3841.folatetransformationsII.plants.	Prevotella_bivia	site	oral	-1.55	130	0.2000	0.0000	0.0005
PWY.5695.inosine5..phosphatedegradation	Prevotella_bivia	site	oral	-1.43	130	0.1846	0.0001	0.0012
PWY.6151.S.adenosyl.L.methioninesalvageI	Prevotella_bivia	site	oral	-1.64	130	0.2231	0.0000	0.0003
PWY.6385.peptidoglycanbiosynthesisIII.mycobacteria.	Prevotella_bivia	site	oral	-1.34	130	0.2000	0.0001	0.0009
PWY.6386.UDP.N.acetylmuramoyl.pentapeptidebiosynthesisII.lysine.contair	Prevotella_bivia	site	oral	-1.25	130	0.2000	0.0001	0.0011
PWY.6387.UDP.N.acetylmuramoyl.pentapeptidebiosynthesisI.meso.diamino	Prevotella_bivia	site	oral	-1.65	130	0.2231	0.0002	0.0017
PWY.6609.adenineandadenosinesalvageIII	Prevotella_bivia	site	oral	-1.33	130	0.1846	0.0002	0.0013
PWY.6700.queuosinebiosynthesisI.denovo.	Prevotella_bivia	site	oral	-1.55	130	0.2154	0.0002	0.0015
PWY.7219.adenosineribonucleotidesdenovobiosynthesis	Prevotella_bivia	site	oral	-1.71	130	0.2538	0.0001	0.0011
PWY.7221.guanosineribonucleotidesdenovobiosynthesis	Prevotella_bivia	site	oral	-1.47	130	0.2154	0.0009	0.0047
BRANCHED.CHAIN.AA.SYN.PWY.superpathwayofbranchedchainaminoacidbi	Prevotella_copri	site	oral	-2.67	130	0.3615	0.0000	0.0000
HISTSYN.PWY.L.histidinebiosynthesis	Prevotella_copri	site	oral	-1.36	130	0.2923	0.0000	0.0000
ILEUSYN.PWY.L.isoleucinebiosynthesisI.fromthreonine.	Prevotella_copri	site	oral	-2.73	130	0.3615	0.0000	0.0000
PEPTIDOGLYCANSYN.PWY.peptidoglycanbiosynthesisI.meso.diaminopimelat	Prevotella_copri	site	oral	-1.98	130	0.3308	0.0000	0.0000
PWY.1042.glycolysisIV	Prevotella_copri	site	oral	-1.96	130	0.3308	0.0000	0.0000
PWY.5103.L.isoleucinebiosynthesisIII	Prevotella_copri	site	oral	-2.74	130	0.3615	0.0000	0.0000
PWY.5695.inosine5..phosphatedegradation	Prevotella_copri	site	oral	-1.34	130	0.2769	0.0000	0.0001
PWY.6122.5.aminoimidazoleribonucleotidebiosynthesisII	Prevotella_copri	site	oral	-2.77	130	0.3769	0.0000	0.0000
PWY.6151.S.adenosyl.L.methioninesalvageI	Prevotella_copri	site	oral	-1.64	130	0.3154	0.0000	0.0000
PWY.6277.superpathwayof5.aminoimidazoleribonucleotidebiosynthesis	Prevotella_copri	site	oral	-2.77	130	0.3769	0.0000	0.0000
PWY.6385.peptidoglycanbiosynthesisIII.mycobacteria.	Prevotella_copri	site	oral	-2.05	130	0.3308	0.0000	0.0000
PWY.6386.UDP.N.acetylmuramoyl.pentapeptidebiosynthesisII.lysine.contair	Prevotella_copri	site	oral	-2.14	130	0.3231	0.0000	0.0000
PWY.6387.UDP.N.acetylmuramoyl.pentapeptidebiosynthesisI.meso.diamino	Prevotella_copri	site	oral	-2.01	130	0.3308	0.0000	0.0000
PWY.6609.adenineandadenosinesalvageIII	Prevotella_copri	site	oral	-2.17	130	0.3385	0.0000	0.0000
PWY.6700.queuosinebiosynthesisI.denovo.	Prevotella_copri	site	oral	-1.95	130	0.3154	0.0000	0.0000
PWY.6897.thiaminediphosphatesalvageII	Prevotella_copri	site	oral	-1.38	130	0.2846	0.0000	0.0000
PWY.7111.pyruvatefermentationtoisobutanol.engineered.	Prevotella_copri	site	oral	-2.79	130	0.3615	0.0000	0.0000
PWY.7219.adenosineribonucleotidesdenovobiosynthesis	Prevotella_copri	site	oral	-2.70	130	0.3692	0.0000	0.0000
PWY.7221.guanosineribonucleotidesdenovobiosynthesis	Prevotella_copri	site	oral	-2.41	130	0.3462	0.0000	0.0000
PWY.7357.thiaminephosphateformationfrompyrithiamineandoxythiamine.ye	Prevotella_copri	site	oral	-1.47	130	0.2846	0.0000	0.0000
PWY0.1586.peptidoglycanmaturation.meso.diaminopimelatecontaining.	Prevotella_copri	site	oral	-1.83	130	0.3154	0.0000	0.0000
VALSYN.PWY.L.valinebiosynthesis	Prevotella_copri	site	oral	-2.73	130	0.3615	0.0000	0.0000
PWY.7219.adenosineribonucleotidesdenovobiosynthesis	Prevotella_corporis	site	oral	-1.16	130	0.1154	0.0004	0.0026

Supplementary Table 6. (Continuation)

PWY	sps	metadata	value	coef	N	Prevalence	pval	qval
PWY.1042.glycolysisIV	Prevotella_disiens	site	oral	-0.76	130	0.1692	0.0124	0.0443
PWY.6151.S.adenosyl.L.methioninesalvageI	Prevotella_disiens	site	oral	-0.90	130	0.1769	0.0080	0.0298
PWY.7219.adenosineribonucleotidesdenovobiosynthesis	Prevotella_disiens	site	oral	-0.85	130	0.1769	0.0069	0.0264
PWY.7221.guanosineribonucleotidesdenovobiosynthesis	Prevotella_disiens	site	oral	-0.77	130	0.1692	0.0118	0.0421
PWY0.1586.peptidoglycanmaturation.meso.diaminopimelatecontaining.	Prevotella_disiens	site	oral	-0.79	130	0.1692	0.0077	0.0289
NONMEVIPP.PWY.methylerythritolphosphatepathwayI	Prevotella_sp_885	site	oral	-0.62	130	0.1462	0.0011	0.0056
PEPTIDOGLYCANSYN.PWY.peptidoglycanbiosynthesisI.meso.diaminopimelat	Prevotella_sp_885	site	oral	-0.66	130	0.1692	0.0002	0.0016
PWY.3841.folatetransformationsII.plants.	Prevotella_sp_885	site	oral	-0.73	130	0.1769	0.0001	0.0010
PWY.6122.5.aminoimidazoleribonucleotidebiosynthesisII	Prevotella_sp_885	site	oral	-0.75	130	0.1692	0.0001	0.0011
PWY.6151.S.adenosyl.L.methioninesalvageI	Prevotella_sp_885	site	oral	-0.60	130	0.1692	0.0025	0.0107
PWY.6277.superpathwayof5.aminoimidazoleribonucleotidebiosynthesis	Prevotella_sp_885	site	oral	-0.75	130	0.1692	0.0001	0.0011
PWY.6385.peptidoglycanbiosynthesisIII.mycobacteria.	Prevotella_sp_885	site	oral	-0.63	130	0.1615	0.0003	0.0022
PWY.6386.UDP.N.acetylmuramoyl.pentapeptidebiosynthesisII.lysine.contair	Prevotella_sp_885	site	oral	-0.62	130	0.1615	0.0003	0.0022
PWY.6387.UDP.N.acetylmuramoyl.pentapeptidebiosynthesisI.meso.diamino	Prevotella_sp_885	site	oral	-0.67	130	0.1692	0.0002	0.0016
PWY.6700.queuosinebiosynthesisI.denovo.	Prevotella_sp_885	site	oral	-0.66	130	0.1846	0.0001	0.0009
PWY.7111.pyruvatefermentationtoisobutanol.engineered.	Prevotella_sp_885	site	oral	-0.70	130	0.1615	0.0003	0.0021
PWY.7219.adenosineribonucleotidesdenovobiosynthesis	Prevotella_sp_885	site	oral	-1.50	130	0.2077	0.0000	0.0001
PWY.7220.adenosinedeoxyribonucleotidesdenovobiosynthesisII	Prevotella_sp_885	site	oral	-0.85	130	0.1692	0.0001	0.0011
PWY.7221.guanosineribonucleotidesdenovobiosynthesis	Prevotella_sp_885	site	oral	-1.33	130	0.2000	0.0000	0.0002
PWY.7222.guanosinedeoxyribonucleotidesdenovobiosynthesisII	Prevotella_sp_885	site	oral	-0.85	130	0.1692	0.0001	0.0011
VALSYN.PWY.L.valinebiosynthesis	Prevotella_sp_885	site	oral	-0.70	130	0.1615	0.0003	0.0021
BRANCHED.CHAIN.AA.SYN.PWY.superpathwayofbranchedchainaminoacidbi	Prevotella_sp_AM42_24	site	oral	-1.23	130	0.2692	0.0000	0.0000
HISTSYN.PWY.L.histidinebiosynthesis	Prevotella_sp_AM42_24	site	oral	-1.48	130	0.2231	0.0000	0.0000
ILEUSYN.PWY.L.isoleucinebiosynthesisI.fromthreonine.	Prevotella_sp_AM42_24	site	oral	-1.28	130	0.2692	0.0000	0.0000
PEPTIDOGLYCANSYN.PWY.peptidoglycanbiosynthesisI.meso.diaminopimelat	Prevotella_sp_AM42_24	site	oral	-1.15	130	0.2000	0.0000	0.0001
PWY.1042.glycolysisIV	Prevotella_sp_AM42_24	site	oral	-1.09	130	0.2000	0.0000	0.0001
PWY.3841.folatetransformationsII.plants.	Prevotella_sp_AM42_24	site	oral	-0.98	130	0.1846	0.0000	0.0003
PWY.5103.L.isoleucinebiosynthesisIII	Prevotella_sp_AM42_24	site	oral	-1.23	130	0.2692	0.0000	0.0000
PWY.5695.inosine5..phosphatedegradation	Prevotella_sp_AM42_24	site	oral	-1.14	130	0.2000	0.0000	0.0001
PWY.6122.5.aminoimidazoleribonucleotidebiosynthesisII	Prevotella_sp_AM42_24	site	oral	-1.29	130	0.2769	0.0000	0.0000
PWY.6151.S.adenosyl.L.methioninesalvageI	Prevotella_sp_AM42_24	site	oral	-1.16	130	0.2154	0.0000	0.0000
PWY.6277.superpathwayof5.aminoimidazoleribonucleotidebiosynthesis	Prevotella_sp_AM42_24	site	oral	-1.29	130	0.2769	0.0000	0.0000
PWY.6385.peptidoglycanbiosynthesisIII.mycobacteria.	Prevotella_sp_AM42_24	site	oral	-1.14	130	0.2000	0.0000	0.0001
PWY.6386.UDP.N.acetylmuramoyl.pentapeptidebiosynthesisII.lysine.contair	Prevotella_sp_AM42_24	site	oral	-1.11	130	0.1923	0.0000	0.0002
PWY.6387.UDP.N.acetylmuramoyl.pentapeptidebiosynthesisI.meso.diamino	Prevotella_sp_AM42_24	site	oral	-1.18	130	0.2077	0.0000	0.0001
PWY.6609.adenineandadenosinesalvageIII	Prevotella_sp_AM42_24	site	oral	-1.20	130	0.2385	0.0000	0.0001
PWY.6700.queuosinebiosynthesisI.denovo.	Prevotella_sp_AM42_24	site	oral	-1.14	130	0.2077	0.0000	0.0001
PWY.7111.pyruvatefermentationtoisobutanol.engineered.	Prevotella_sp_AM42_24	site	oral	-1.27	130	0.2692	0.0000	0.0000
PWY.7219.adenosineribonucleotidesdenovobiosynthesis	Prevotella_sp_AM42_24	site	oral	-1.30	130	0.2615	0.0000	0.0000
PWY.7221.guanosineribonucleotidesdenovobiosynthesis	Prevotella_sp_AM42_24	site	oral	-1.51	130	0.2385	0.0000	0.0000
VALSYN.PWY.L.valinebiosynthesis	Prevotella_sp_AM42_24	site	oral	-1.37	130	0.2769	0.0000	0.0000
PWY.6277.superpathwayof5.aminoimidazoleribonucleotidebiosynthesis	Prevotella_sp_CAG_520	site	oral	-1.22	130	0.2385	0.0000	0.0000
PWY.6385.peptidoglycanbiosynthesisIII.mycobacteria.	Prevotella_sp_CAG_520	site	oral	-0.73	130	0.1692	0.0001	0.0011
PWY.6387.UDP.N.acetylmuramoyl.pentapeptidebiosynthesisI.meso.diamino	Prevotella_sp_CAG_520	site	oral	-0.71	130	0.1692	0.0001	0.0012
PWY.6700.queuosinebiosynthesisI.denovo.	Prevotella_sp_CAG_520	site	oral	-1.74	130	0.2154	0.0000	0.0000
PWY.7219.adenosineribonucleotidesdenovobiosynthesis	Prevotella_sp_CAG_520	site	oral	-2.57	130	0.2462	0.0000	0.0000
PWY.7220.adenosinedeoxyribonucleotidesdenovobiosynthesisII	Prevotella_sp_CAG_520	site	oral	-0.90	130	0.1923	0.0000	0.0002
PWY.7221.guanosineribonucleotidesdenovobiosynthesis	Prevotella_sp_CAG_520	site	oral	-1.98	130	0.2462	0.0000	0.0000
PWY.7222.guanosinedeoxyribonucleotidesdenovobiosynthesisII	Prevotella_sp_CAG_520	site	oral	-0.90	130	0.1923	0.0000	0.0002
PWY.6122.5.aminoimidazoleribonucleotidebiosynthesisII	Prevotella_stercorea	site	oral	-0.90	130	0.1385	0.0002	0.0013
PWY.6277.superpathwayof5.aminoimidazoleribonucleotidebiosynthesis	Prevotella_stercorea	site	oral	-0.90	130	0.1385	0.0002	0.0013
PWY.6700.queuosinebiosynthesisI.denovo.	Prevotella_stercorea	site	oral	-0.83	130	0.1154	0.0010	0.0052
PWY.7219.adenosineribonucleotidesdenovobiosynthesis	Prevotella_stercorea	site	oral	-0.81	130	0.1538	0.0003	0.0019
PWY.7220.adenosinedeoxyribonucleotidesdenovobiosynthesisII	Prevotella_stercorea	site	oral	-1.08	130	0.1154	0.0007	0.0038
PWY.7221.guanosineribonucleotidesdenovobiosynthesis	Prevotella_stercorea	site	oral	-1.07	130	0.1692	0.0001	0.0006
PWY.7222.guanosinedeoxyribonucleotidesdenovobiosynthesisII	Prevotella_stercorea	site	oral	-1.08	130	0.1154	0.0007	0.0038
PWY.7111.pyruvatefermentationtoisobutanol.engineered.	Roseburia_faecis	site	oral	-0.94	130	0.1462	0.0025	0.0109
PWY.7219.adenosineribonucleotidesdenovobiosynthesis	Roseburia_faecis	site	oral	-0.61	130	0.1231	0.0101	0.0365
PWY.7111.pyruvatefermentationtoisobutanol.engineered.	Roseburia_inulinivorans	site	oral	-1.07	130	0.1615	0.0001	0.0006
VALSYN.PWY.L.valinebiosynthesis	Roseburia_inulinivorans	site	oral	-0.81	130	0.1154	0.0009	0.0049
ARGSYNBSUB.PWY.L.argininebiosynthesisII.acetylcycle.	Ruminococcus_torques	site	oral	-0.74	130	0.1154	0.0009	0.0047
BRANCHED.CHAIN.AA.SYN.PWY.superpathwayofbranchedchainaminoacidbi	Ruminococcus_torques	site	oral	-0.95	130	0.1385	0.0002	0.0012
ILEUSYN.PWY.L.isoleucinebiosynthesisI.fromthreonine.	Ruminococcus_torques	site	oral	-0.96	130	0.1385	0.0002	0.0012
NONOXIPENT.PWY.pentosephosphatepathway.non.oxidativebranch.I	Ruminococcus_torques	site	oral	-0.94	130	0.1385	0.0002	0.0015
PWY.5103.L.isoleucinebiosynthesisIII	Ruminococcus_torques	site	oral	-1.04	130	0.1385	0.0001	0.0011
PWY.6121.5.aminoimidazoleribonucleotidebiosynthesisI	Ruminococcus_torques	site	oral	-0.97	130	0.1462	0.0002	0.0013
PWY.6122.5.aminoimidazoleribonucleotidebiosynthesisII	Ruminococcus_torques	site	oral	-0.95	130	0.1462	0.0001	0.0012
PWY.6151.S.adenosyl.L.methioninesalvageI	Ruminococcus_torques	site	oral	-1.09	130	0.1385	0.0001	0.0008
PWY.6277.superpathwayof5.aminoimidazoleribonucleotidebiosynthesis	Ruminococcus_torques	site	oral	-0.95	130	0.1462	0.0001	0.0012
PWY.6387.UDP.N.acetylmuramoyl.pentapeptidebiosynthesisI.meso.diamino	Ruminococcus_torques	site	oral	-0.85	130	0.1154	0.0005	0.0031
PWY.6609.adenineandadenosinesalvageIII	Ruminococcus_torques	site	oral	-0.79	130	0.1077	0.0012	0.0058
PWY.6737.starchdegradationV	Ruminococcus_torques	site	oral	-0.88	130	0.1077	0.0007	0.0040
PWY.7111.pyruvatefermentationtoisobutanol.engineered.	Ruminococcus_torques	site	oral	-1.42	130	0.1846	0.0001	0.0005
PWY.7219.adenosineribonucleotidesdenovobiosynthesis	Ruminococcus_torques	site	oral	-1.12	130	0.1692	0.0001	0.0011
PWY.7221.guanosineribonucleotidesdenovobiosynthesis	Ruminococcus_torques	site	oral	-1.08	130	0.1538	0.0001	0.0007
PWY.7357.thiaminephosphateformationfrompyrithiamineandoxythiamine.y	Ruminococcus_torques	site	oral	-1.00	130	0.1077	0.0009	0.0048
VALSYN.PWY.L.valinebiosynthesis	Ruminococcus_torques	site	oral	-1.02	130	0.1769	0.0001	0.0012

Supplementary Table 7. Significant associations among pathways and subjects´metadata

PWY	metadata	value	coef	N	prevalence	pval	qval
NAGLIPASYN.PWY.lipidIVAbiosynthesis.E.coli.	age_code	und33yo	1.794465602	130	0.623076923	0.001247902	0.008185654
PYRIDOXSYN.PWY.pyridoxal5..phosphatebiosynthesisI	age_code	und33yo	1.747023112	130	0.584615385	0.00026699	0.002441071
PENTOSE.P.PWY.pentosephosphatepathway	age_code	und33yo	1.633231036	130	0.653846154	0.007851369	0.038747908
PWY.7117.C4photosyntheticcarbonassimilationcycle.PEPCKtype	age_code	und33yo	1.556155364	130	0.615384615	0.021924601	0.092619138
PWY0.845.superpathwayofpyridoxal5..phosphatebiosynthesisandsalvage	age_code	und33yo	1.50920761	130	0.546153846	0.000411604	0.003447505
HEMESYN2.PWY.hemebbiosynthesisII.oxygen.independent.	age_code	und33yo	1.466848951	130	0.569230769	0.021909954	0.092615002
PWY.7219.adenosineribonucleotidesdenovobiosynthesis	age_code	und33yo	1.407536092	130	0.107692308	0.00157561	0.009959265
PWY.5189.tetrapyrrolebiosynthesisII.fromglycine.	age_code	und33yo	1.40697351	130	0.484615385	0.00970311	0.046139142
PWY.5913.partialTCAcycle.obligateautotrophs.	age_code	und33yo	1.387021523	130	0.653846154	0.044676768	0.165879332
GLYCOLYSIS.E.D.superpathwayofglycolysisandtheEntner.Doudoroffpathway	age_code	und33yo	1.305900554	130	0.453846154	0.012404512	0.057023728
HOMOSER.METSYN.PWY.L.methioninebiosynthesisI	age_code	und33yo	1.279544793	130	0.661538462	0.034370175	0.134697689
HISDEG.PWY.L.histidinedegradationI	age_code	und33yo	1.278118626	130	0.130769231	0.001280969	0.008370149
PWY0.162.superpathwayofpyrimidineribonucleotidesdenovobiosynthesis	age_code	und33yo	1.258862343	130	0.669230769	0.012940414	0.058848486
PWY.5484.glycolysisII.fromfructose6.phosphate.	age_code	und33yo	1.248546542	130	0.692307692	0.002662078	0.015349481
PWY66.399.gluconeogenesisIII	age_code	und33yo	1.227798851	130	0.384615385	0.010782233	0.050560836
UNINTEGRATED	age_code	und33yo	1.174537827	130	0.123076923	0.000274627	0.002487796
GLYCOLYSIS.glycolysisI.fromglucose6.phosphate.	age_code	und33yo	1.167763989	130	0.7	0.004957281	0.026222121
PWY.6588.pyruvatefermentationtoacetone	age_code	und33yo	1.166160021	130	0.276923077	0.005148472	0.027001583
GLUCONEO.PWY.gluconeogenesisI	age_code	und33yo	1.105366893	130	0.653846154	0.003069928	0.017291629
P441.PWY.superpathwayofN.acetylneuraminatedegradation	age_code	und33yo	1.102240021	130	0.5	0.032960753	0.130001201
PPGPPMET.PWY.ppGppmetabolism	age_code	und33yo	1.099441303	130	0.630769231	0.036827117	0.142868769
PWY.7383.anaerobicenergymetabolism.invertebrates.cytosol.	age_code	und33yo	1.050795603	130	0.553846154	0.027576644	0.112633718
PWY0.781.aspartatesuperpathway	age_code	und33yo	0.984974853	130	0.361538462	0.026109768	0.107680942
PWY0.1479.tRNAprocessing	age_code	und33yo	0.982153727	130	0.553846154	0.026000055	0.107490159
ANAEROFRUCAT.PWY.homolacticfermentation	age_code	und33yo	0.967162433	130	0.676923077	0.003969001	0.02172371
PWY.6608.guanosinenucleotidesdegradationIII	age_code	und33yo	0.949185045	130	0.476923077	0.031688026	0.126122778
HISDEG.PWY.L.histidinedegradationI	age_code	und33yo	0.947892548	130	0.115384615	0.00187693	0.01153044
P164.PWY.purinenucleobasesdegradationI.anaerobic.	age_code	und33yo	0.946237707	130	0.376923077	0.012690491	0.05802346
PWY.7221.guanosineribonucleotidesdenovobiosynthesis	age_code	und33yo	0.946208261	130	0.146153846	0.02134193	0.090780239
PWY.6803.phosphatidylcholineacylediting	age_code	und33yo	0.941461014	130	0.353846154	0.036232172	0.141178378
PWY.7220.adenosinedeoxyribonucleotidesdenovobiosynthesisII	age_code	und33yo	0.929073096	130	0.830769231	0.022058371	0.093068194
PWY.7222.guanosinedeoxyribonucleotidesdenovobiosynthesisII	age_code	und33yo	0.929073096	130	0.830769231	0.022058371	0.093068194
PWY.7197.pyrimidinedeoxyribonucleotidephosphorylation	age_code	und33yo	0.917477525	130	0.8	0.027121398	0.111243702
PWY.5695.inosine5..phosphatedegradation	age_code	und33yo	0.894101621	130	0.146153846	0.036459492	0.141738107
SER.GLYSYN.PWY.superpathwayofL.serineandglycinebiosynthesisI	age_code	und33yo	0.847706714	130	0.169230769	0.045583896	0.168508717
PWY.7282.4.amino.2.methyl.5.diphosphomethylpyrimidinebiosynthesisII	age_code	und33yo	0.846238143	130	0.692307692	0.009227366	0.044218269
PWY.6126.superpathwayofadenosinenucleotidesdenovobiosynthesisII	age_code	und33yo	0.824782714	130	0.830769231	0.017191435	0.075789956
PHOSLIPSYN.PWY.superpathwayofphospholipidbiosynthesisI.bacteria.	age_code	und33yo	0.820522822	130	0.746153846	0.037401035	0.144486554
TCA.TCAcycleI.prokaryotic.	age_code	und33yo	0.819053547	130	0.507692308	0.044384142	0.165245576
PWY0.1319.CDP.diacylglycerolbiosynthesisII	age_code	und33yo	0.818437507	130	0.861538462	0.032526344	0.128696337
GLCMANNANAUT.PWY.superpathwayofN.acetylglucosamine.N.acetylmannosamineandN.acetyln	age_code	und33yo	0.804331839	130	0.723076923	0.02996448	0.120928716
PWY.5667.CDP.diacylglycerolbiosynthesisI	age_code	und33yo	0.788653652	130	0.846153846	0.04189189	0.158669337
PWY.7663.gondoatebiosynthesis.anaerobic.	age_code	und33yo	0.787164443	130	0.884615385	0.005470302	0.028425433
PWY.7228.superpathwayofguanosinenucleotidesdenovobiosynthesisI	age_code	und33yo	0.786174484	130	0.815384615	0.031776331	0.126283951
UNINTEGRATED	age_code	und33yo	0.7758806	130	0.207692308	0.018831514	0.081379044
HEXITOLDEGSUPER.PWY.superpathwayofhexitoldegradation.bacteria.	age_code	und33yo	0.763387762	130	0.315384615	0.031031236	0.123905513
PWY.7229.superpathwayofadenosinenucleotidesdenovobiosynthesisI	age_code	und33yo	0.749433877	130	0.830769231	0.014515214	0.065308826
PYRIDOXSYN.PWY.pyridoxal5..phosphatebiosynthesisI	age_code	und33yo	0.747376361	130	0.146153846	0.039585593	0.15154349
THISYNARA.PWY.superpathwayofthiaminediphosphatebiosynthesisIII.eukaryotes.	age_code	und33yo	0.740330809	130	0.646153846	0.020114485	0.086247641
PWY.7219.adenosineribonucleotidesdenovobiosynthesis	age_code	und33yo	0.737513361	130	0.2	0.021372724	0.090797227
HISDEG.PWY.L.histidinedegradationI	age_code	und33yo	0.725824761	130	0.630769231	0.026708637	0.109749985
PWY.6163.chorismatebiosynthesisfrom3.dehydroquinate	age_code	und33yo	0.719385976	130	0.138461538	0.015658707	0.069850821
PWY.6168.flavinbiosynthesisIII.fungi.	age_code	und33yo	0.712342412	130	0.807692308	0.004014215	0.021900419
COLANSYN.PWY.colanicacidbuildingblocksbiosynthesis	age_code	und33yo	0.711488512	130	0.430769231	0.031702871	0.126122778
GLYOXYLATE.BYPASS.glyoxylatecycle	age_code	und33yo	0.709256624	130	0.184615385	0.032251056	0.127871947
SER.GLYSYN.PWY.superpathwayofL.serineandglycinebiosynthesisI	age_code	und33yo	0.706319679	130	0.8	0.04182747	0.158513945
PWY.6969.TCAcycleV.2.oxoglutaratesynthase.	age_code	und33yo	0.687177688	130	0.253846154	0.023972569	0.10014681
PWY.7560.methylerythritolphosphatepathwayII	age_code	und33yo	0.687162435	130	0.146153846	0.034265548	0.134443168
CITRULBIO.PWY.L.citrullinebiosynthesis	age_code	und33yo	0.679603261	130	0.292307692	0.032534951	0.128696337
NONOXIPENT.PWY.pentosephosphatepathway.non.oxidativebranch.I	age_code	und33yo	0.676353185	130	0.153846154	0.017450903	0.076634686
PYRIDNUCSYN.PWY.NADdenovobiosynthesisI.fromaspartate.	age_code	und33yo	0.670435626	130	0.738461538	0.026133817	0.107714563
PWY.7199.pyrimidinedeoxyribonucleosidessalvage	age_code	und33yo	0.669789141	130	0.830769231	0.005977184	0.030729437
PWY.7323.superpathwayofGDP.mannose.derivedO.antigenbuildingblocksbiosynthesis	age_code	und33yo	0.663901508	130	0.284615385	0.03331461	0.131320417
SER.GLYSYN.PWY.superpathwayofL.serineandglycinebiosynthesisI	age_code	und33yo	0.660364987	130	0.146153846	0.029529861	0.119530667
PWY.5686.UMPbiosynthesisI	age_code	und33yo	0.659281768	130	0.146153846	0.032489814	0.128668019
PWY.5367.petroselinatebiosynthesis	age_code	und33yo	0.649295049	130	0.223076923	0.045945425	0.169330004
PWY4LZ.257.superpathwayoffermentation.Chlamydomonasreinhardtii.	age_code	und33yo	0.645851679	130	0.192307692	0.021475141	0.091077147
P42.PWY.incompletereductiveTCAcycle	age_code	und33yo	0.641275706	130	0.415384615	0.048624739	0.175723323
PWY.4984.ureacycle	age_code	und33yo	0.632024683	130	0.284615385	0.048854814	0.176272746
PWY.3841.folatetransformationsII.plants.	age_code	und33yo	0.625126594	130	0.146153846	0.039403813	0.151018232
CALVIN.PWY.Calvin.Benson.Basshamcycle	age_code	und33yo	0.623038061	130	0.8	0.017248079	0.075984718
UNINTEGRATED	age_code	und33yo	0.622295011	130	0.115384615	0.003172473	0.017810007
PWY.6700.queuosinebiosynthesisI.denovo.	age_code	und33yo	0.618979804	130	0.153846154	0.046265047	0.170006484
PWY.5973.cis.vaccenatebiosynthesis	age_code	und33yo	0.610394354	130	0.869230769	0.024954544	0.103356963
UNINTEGRATED	age_code	und33yo	0.606338591	130	0.146153846	0.012520068	0.057335745
PWY.6124.inosine.5..phosphatebiosynthesisII.unclassified	age_code	und33yo	0.599248479	130	0.138461538	0.00649172	0.032847676
PWY.6703.preQ0biosynthesis	age_code	und33yo	0.589009607	130	0.130769231	0.03732485	0.14439464
PWY.6700.queuosinebiosynthesisI.denovo.	age_code	und33yo	0.577058081	130	0.876923077	0.005127254	0.026931996
SER.GLYSYN.PWY.superpathwayofL.serineandglycinebiosynthesisI	age_code	und33yo	0.576565386	130	0.146153846	0.041286166	0.157165766
SO4ASSIM.PWY.assimilatorysulfatereductionI	age_code	und33yo	0.573095267	130	0.123076923	0.037139488	0.143886317

Supplementary Table 7.(Continuation)

PWY	metadata	value	coef	N	prevalence	pval	qval
PWY0.1319.CDP.diacylglycerolbiosynthesisII	age_code	und33yo	0.568351051	130	0.130769231	0.031351569	0.125037216
UNINTEGRATED	age_code	und33yo	0.555010721	130	0.153846154	0.032699232	0.129195332
GLYCOGENSYNTH.PWY.glycogenbiosynthesisI.fromADP.D.Glucose.	age_code	und33yo	0.554310401	130	0.861538462	0.026657321	0.109605586
PWY.724.superpathwayofL.lysine.L.threonineandL.methioninebiosynthesisII	age_code	und33yo	0.553013773	130	0.784615385	0.037359155	0.144407094
PWY.6123.inosine.5..phosphatebiosynthesisI.unclassified	age_code	und33yo	0.532868589	130	0.130769231	0.008033473	0.039445518
TCA.GLYOX.BYPASS.superpathwayofglyoxylatebypassandTCA	age_code	und33yo	0.525722158	130	0.153846154	0.023738782	0.099415321
PWY.3841.folatetransformationsII.plants.	age_code	und33yo	0.520585321	130	0.884615385	0.012368621	0.056897336
RIBOSYN2.PWY.flavinbiosynthesisI.bacteriaandplants.	age_code	und33yo	0.512308862	130	0.861538462	0.020123601	0.086247641
GLYCOLYSIS.TCA.GLYOX.BYPASS.superpathwayofglycolysis.pyruvatedehydrogenase.TCA.andglyox	age_code	und33yo	0.508220612	130	0.138461538	0.027532884	0.112633718
UNINTEGRATED	age_code	und33yo	0.503998615	130	0.123076923	0.045536566	0.168425638
COA.PWY.1.superpathwayofcoenzymeAbiosynthesisIII.mammals.	age_code	und33yo	0.482340538	130	0.123076923	0.031716826	0.126122778
ARGSYN.PWY.L.argininebiosynthesisI.viaL.ornithine.	age_code	und33yo	0.457720357	130	0.115384615	0.04334694	0.162996042
PWY.6151.S.adenosyl.L.methioninesalvageI	age_code	und33yo	0.457700087	130	0.915384615	0.04311533	0.162395483
PWY.7400.L.argininebiosynthesisIV.archaebacteria.	age_code	und33yo	0.456992201	130	0.115384615	0.043432149	0.16304501
UNINTEGRATED	age_code	und33yo	0.451848289	130	0.130769231	0.0441165	0.164429814
PWY.5367.petroselinatebiosynthesis	age_code	und33yo	0.447651993	130	0.115384615	0.047826912	0.173673717
PWY.3841.folatetransformationsII.plants.	age_code	und33yo	0.442875494	130	0.130769231	0.033559378	0.132208342
COA.PWY.1.superpathwayofcoenzymeAbiosynthesisIII.mammals.	age_code	und33yo	0.437202004	130	0.9	0.015829762	0.070521016
PWY.7111.pyruvatefermentationtoisobutanol.engineered.	age_code	und33yo	0.413721278	130	0.107692308	0.027551988	0.112633718
PWY.6151.S.adenosyl.L.methioninesalvageI	age_code	und33yo	0.41125471	130	0.107692308	0.047220129	0.172301342
UNINTEGRATED	age_code	und33yo	0.304762847	130	0.976923077	0.00928092	0.044412084
PWY.7221.guanosineribonucleotidesdenovobiosynthesis	age_code	und33yo	-0.490484849	130	0.123076923	0.038501203	0.14806138
PWY.7219.adenosineribonucleotidesdenovobiosynthesis	age_code	und33yo	-0.50506207	130	0.123076923	0.04568473	0.168789384
CALVIN.PWY.Calvin.Benson.Basshamcycle	age_code	und33yo	-0.548473871	130	0.123076923	0.046648856	0.171045807
PWY.6163.chorismatebiosynthesisfrom3.dehydroquinate	age_code	und33yo	-0.579213883	130	0.246153846	0.047592736	0.173089794
HSERMETANA.PWY.L.methioninebiosynthesisIII.unclassified	age_code	und33yo	-0.648163709	130	0.223076923	0.021007569	0.089695835
PWY0.1586.peptidoglycanmaturation.meso.diaminopimelatecontaining.	age_code	und33yo	-0.776027914	130	0.315384615	0.034359868	0.134697689
GLYCOGENSYNTH.PWY.glycogenbiosynthesisI.fromADP.D.Glucose.	ART	yes	1.222021363	130	0.269230769	0.017272667	0.074642595
PWY.7219.adenosineribonucleotidesdenovobiosynthesis	ART	yes	1.179790403	130	0.261538462	0.036770401	0.129409404
PWY0.1296.purineribonucleosidesdegradation	ART	yes	1.157232528	130	0.176923077	0.039364491	0.136459987
ANAGLYCOLYSIS.PWY.glycolysisIII.fromglucose.	ART	yes	1.076401503	130	0.176923077	0.042220634	0.14536942
PWY.6386.UDP.N.acetylmuramoyl.pentapeptidebiosynthesisII.lysine.containing.	ART	yes	0.925003285	130	0.3	0.03540245	0.126504268
PWY.5686.UMPbiosynthesisI	ART	yes	-0.703851863	130	0.192307692	0.032380754	0.117667508
PWY.7220.adenosinedeoxyribonucleotidesdenovobiosynthesisII	ART	yes	-0.728240266	130	0.192307692	0.026466936	0.101055573
PWY.7222.guanosinedeoxyribonucleotidesdenovobiosynthesisII	ART	yes	-0.728240266	130	0.192307692	0.026466936	0.101055573
PWY.6737.starchdegradationV	ART	yes	-0.883627299	130	0.107692308	0.011519282	0.055331748
PWY.1042.glycolysisIV	ART	yes	-0.885565633	130	0.2	0.021025613	0.08568738
PWY.6123.inosine.5..phosphatebiosynthesisI	ART	yes	-0.92605321	130	0.153846154	0.034965191	0.125400917
PWY.6151.S.adenosyl.L.methioninesalvageI	ART	yes	-0.928487236	130	0.184615385	0.030014034	0.110851251
PWY.2942.L.lysinebiosynthesisIII	ART	yes	-1.064119456	130	0.207692308	0.007778345	0.042504892
HISTSYN.PWY.L.histidinebiosynthesis	ART	yes	-1.099162541	130	0.223076923	0.025165097	0.097446159
PWY.7221.guanosineribonucleotidesdenovobiosynthesis	ART	yes	-1.122435395	130	0.238461538	0.020710408	0.084606346
COA.PWY.coenzymeAbiosynthesisI.prokaryotic.	ART	yes	-1.251227016	130	0.153846154	0.035531845	0.126520297
HISDEG.PWY.L.histidinedegradationI	ART	yes	-1.361728011	130	0.161538462	0.024140202	0.094807192
PWY.5030.L.histidinedegradationIII	ART	yes	-1.365000038	130	0.161538462	0.025176467	0.097446159
PWY.6122.5.aminoimidazoleribonucleotidebiosynthesisII	ART	yes	-1.38458366	130	0.169230769	0.039148309	0.135835869
PWY.6277.superpathwayof5.aminoimidazoleribonucleotidebiosynthesis	ART	yes	-1.38458366	130	0.169230769	0.039148309	0.135835869
PWY.6124.inosine.5..phosphatebiosynthesisII	ART	yes	-1.459685455	130	0.169230769	0.042043408	0.144857356
PWY.5695.inosine5..phosphatedegradation	ART	yes	-1.618523519	130	0.146153846	0.029381885	0.109230971
SER.GLYSYN.PWY.superpathwayofL.serineandglycinebiosynthesisI	ART	yes	-1.638710497	130	0.169230769	0.028372717	0.106178312
PWY.5659.GDP.mannosebiosynthesis	ART	yes	-1.642586515	130	0.153846154	0.012476589	0.058492645
NONOXIPENT.PWY.pentosephosphatepathway.non.oxidativebranch.I	ART	yes	-1.683826546	130	0.169230769	0.020415277	0.083669709
PWY.6700.queuosinebiosynthesisI.denovo.	ART	yes	-1.749583873	130	0.123076923	0.009415871	0.047851456
PWY.6609.adenineandadenosinesalvageIII	ART	yes	-1.803629937	130	0.161538462	0.027463356	0.103691514
PWY.7219.adenosineribonucleotidesdenovobiosynthesis	CD4	under500	1.241925228	130	0.184615385	0.027443876	0.082022145
PWY.6123.inosine.5..phosphatebiosynthesisI	CD4	under500	0.732845942	130	0.115384615	0.023730048	0.073570784
PWY.5188.tetrapyrrolebiosynthesisI.fromglutamate.	CD4	under500	0.654708498	130	0.107692308	0.042884274	0.115558889
PWY0.1296.purineribonucleosidesdegradation	CD4	under500	-0.227559614	130	0.107692308	0.043157328	0.116795316
PWY.6609.adenineandadenosinesalvageIII	CD4	under500	-0.395817962	130	0.115384615	0.032011397	0.092458991
SER.GLYSYN.PWY.superpathwayofL.serineandglycinebiosynthesisI	CD4	under500	-0.528354751	130	0.115384615	0.035272325	0.101786833
PWY.2941.L.lysinebiosynthesisII	CD4	under500	-0.736406949	130	0.276923077	0.044392195	0.119255978
PWY.2942.L.lysinebiosynthesisIII	CD4	under500	-0.755508579	130	0.284615385	0.044123247	0.118373619
COA.PWY.coenzymeAbiosynthesisI.prokaryotic.	CD4	under500	-0.851116744	130	0.246153846	0.041620007	0.113401141
GLYCOGENSYNTH.PWY.glycogenbiosynthesisI.fromADP.D.Glucose.	CD4	under500	-1.059817134	130	0.346153846	0.040004934	0.109635974
PWY.5686.UMPbiosynthesisI	CD4	under500	-1.063984984	130	0.307692308	0.022782459	0.083113036
PWY.7220.adenosinedeoxyribonucleotidesdenovobiosynthesisII	gender	TGW	1.117914918	130	0.376923077	0.031941661	0.101201695
PWY.7222.guanosinedeoxyribonucleotidesdenovobiosynthesisII	gender	TGW	1.117914918	130	0.376923077	0.031941661	0.101201695
PWY.5695.inosine5..phosphatedegradation	gender	TGW	0.845978592	130	0.192307692	0.02356407	0.077509774
PWY.6897.thiaminediphosphatesalvageII	gender	TGW	0.801213807	130	0.184615385	0.021248054	0.070568967
PWY.6317.D.galactosedegradationI.Leloirpathway.	gender	TGW	0.796772479	130	0.207692308	0.029824439	0.095229579
PWY.7219.adenosineribonucleotidesdenovobiosynthesis	gender	TGW	0.781656748	130	0.107692308	0.000226808	0.001623435
PWY.7111.pyruvatefermentationtoisobutanol.engineered.	gender	TGW	0.722279335	130	0.107692308	0.000703062	0.003909148
HSERMETANA.PWY.L.methioninebiosynthesisIII	gender	TGW	0.696456478	130	0.176923077	0.029621436	0.094796056
PWY.7199.pyrimidinedeoxyribonucleosidessalvage	gender	TGW	0.590509582	130	0.246153846	0.044563958	0.133648689
PWY.6386.UDP.N.acetylmuramoyl.pentapeptidebiosynthesisII.lysine.containing.	gender	TGW	0.562772739	130	0.138461538	0.029166253	0.093516023
PWY0.1479.tRNAprocessing	gender	TGW	0.554087967	130	0.130769231	0.01809245	0.061174871
PWY.7199.pyrimidinedeoxyribonucleosidessalvage	gender	TGW	0.544958982	130	0.176923077	0.046739975	0.139070582
PWY.6387.UDP.N.acetylmuramoyl.pentapeptidebiosynthesisI.meso.diaminopimelatecontaining.	gender	TGW	0.542881302	130	0.138461538	0.031662331	0.100504662
PWY.6122.5.aminoimidazoleribonucleotidebiosynthesisII	gender	TGW	0.537892854	130	0.2	0.041849212	0.126859537
PWY.6277.superpathwayof5.aminoimidazoleribonucleotidebiosynthesis	gender	TGW	0.537892854	130	0.2	0.041849212	0.126859537

Supplementary Table 7.(Continuation)

PWY	metadata	value	coef	N	prevalence	pval	qval
PWY.6737.starchdegradationV	gender	TGW	0.537558446	130	0.184615385	0.047568621	0.141370603
DAPLYSINESYN.PWY.L.lysinebiosynthesisI	gender	TGW	0.536414198	130	0.169230769	0.033900756	0.106347927
PEPTIDOGLYCANSYN.PWY.peptidoglycanbiosynthesisI.meso.diaminopimelatecontaining.	gender	TGW	0.509043903	130	0.123076923	0.028203096	0.091059804
PEPTIDOGLYCANSYN.PWY.peptidoglycanbiosynthesisI.meso.diaminopimelatecontaining.	gender	TGW	0.485676082	130	0.115384615	0.039530407	0.121166181
PWY.6385.peptidoglycanbiosynthesisIII.mycobacteria.	gender	TGW	0.482638334	130	0.107692308	0.036453881	0.113060707
PWY.3841.folatetransformationsII.plants.	gender	TGW	0.447569082	130	0.130769231	0.02905984	0.093233652
PWY.5695.inosine5..phosphatedegradation	gender	TGW	0.429202383	130	0.138461538	0.043069988	0.129900106
PWY.6124.inosine.5..phosphatebiosynthesisII	gender	TGW	0.402570229	130	0.130769231	0.034768015	0.108452905
PWY.4242	gender	TGW	0.393685202	130	0.123076923	0.032386963	0.102484773
UDPNAGSYN.PWY.UDP.N.acetyl.D.glucosaminebiosynthesisI	gender	TGW	0.388309091	130	0.107692308	0.029372669	0.094103683
PWY.6121.5.aminoimidazoleribonucleotidebiosynthesisI	gender	TGW	0.387427225	130	0.176923077	0.046374098	0.138339232
PWY.6700.queuosinebiosynthesisI.denovo.	gender	TGW	0.378917149	130	0.130769231	0.04115268	0.125356801
PWY.6386.UDP.N.acetylmuramoyl.pentapeptidebiosynthesisII.lysine.containing.	gender	TGW	0.37242835	130	0.115384615	0.042607311	0.128810442
PWY.7219.adenosineribonucleotidesdenovobiosynthesis	HIV	Pos	1.116139386	130	0.2	0.026507879	0.086133658
PWY.6122.5.aminoimidazoleribonucleotidebiosynthesisII	HIV	Pos	1.024972698	130	0.161538462	0.008029148	0.030047223
PWY.6277.superpathwayof5.aminoimidazoleribonucleotidebiosynthesis	HIV	Pos	1.024972698	130	0.161538462	0.008029148	0.030047223
PWY.6123.inosine.5..phosphatebiosynthesisI	HIV	Pos	0.959421264	130	0.146153846	0.002392856	0.010447162
PWY.7111.pyruvatefermentationtoisobutanol.engineered.	HIV	Pos	0.944700935	130	0.176923077	0.007605127	0.028671555
PWY.6124.inosine.5..phosphatebiosynthesisII	HIV	Pos	0.935241958	130	0.130769231	0.004024684	0.016155961
VALSYN.PWY.L.valinebiosynthesis	HIV	Pos	0.920582988	130	0.146153846	0.007738454	0.029066389
ILEUSYN.PWY.L.isoleucinebiosynthesisI.fromthreonine.	HIV	Pos	0.869799549	130	0.115384615	0.009801686	0.035682069
PWY.7111.pyruvatefermentationtoisobutanol.engineered.	HIV	Pos	0.749137425	130	0.138461538	0.017346305	0.05892642
PWY.6121.5.aminoimidazoleribonucleotidebiosynthesisI	HIV	Pos	0.728310112	130	0.130769231	0.035604294	0.110735021
PWY.7219.adenosineribonucleotidesdenovobiosynthesis	HIV	Pos	0.623228542	130	0.161538462	0.04118926	0.125356801
PWY.5103.L.isoleucinebiosynthesisIII	HIV	Pos	0.62269187	130	0.107692308	0.016368622	0.056130458
BRANCHED.CHAIN.AA.SYN.PWY.superpathwayofbranchedchainaminoacidbiosynthesis	HIV	Pos	0.607701374	130	0.107692308	0.016550398	0.056587832
PWY.6124.inosine.5..phosphatebiosynthesisII	HIV	Pos	0.579242886	130	0.107692308	0.015521751	0.053588001
PWY.7219.adenosineribonucleotidesdenovobiosynthesis	HIV	Pos	0.518772001	130	0.107692308	0.032343992	0.102412566
ANAGLYCOLYSIS.PWY.glycolysisIII.fromglucose.	HIV	Pos	0.478347867	130	0.107692308	0.038522705	0.118578067
PWY.5855.ubiquinol.7biosynthesis.earlydecarboxylation.	HIV	Pos	0.476865209	130	0.123076923	0.020171554	0.067176828
PWY.5856.ubiquinol.9biosynthesis.earlydecarboxylation.	HIV	Pos	0.476865209	130	0.123076923	0.020171554	0.067176828
PWY.5857.ubiquinol.10biosynthesis.earlydecarboxylation.	HIV	Pos	0.476865209	130	0.123076923	0.020171554	0.067176828
PWY.6708.ubiquinol.8biosynthesis.earlydecarboxylation.	HIV	Pos	0.476865209	130	0.123076923	0.020171554	0.067176828
PWY.3841.folatetransformationsII.plants.	HIV	Pos	-0.308620425	130	0.107692308	0.03478517	0.108452905
PWY.6124.inosine.5..phosphatebiosynthesisII	HIV	Pos	-0.339851697	130	0.115384615	0.048917376	0.144786316
PWY.7199.pyrimidinedeoxyribonucleosidessalvage	HIV	Pos	-0.342728193	130	0.107692308	0.046733045	0.139070582
FERMENTATION.PWY.mixedacidfermentation	HIV	Pos	-0.346322305	130	0.107692308	0.017550523	0.059580334
PWY0.1298.superpathwayofpyrimidinedeoxyribonucleosidesdegradation	HIV	Pos	-0.356122102	130	0.107692308	0.046633237	0.138915658
PWY.1042.glycolysisIV	HIV	Pos	-0.387360665	130	0.130769231	0.030758213	0.097940626
DAPLYSINESYN.PWY.L.lysinebiosynthesisI	HIV	Pos	-0.397101637	130	0.130769231	0.048528691	0.143803387
GLYCOLYSIS.glycolysisI.fromglucose6.phosphate.	HIV	Pos	-0.421756135	130	0.115384615	0.024872377	0.081182671
PWY.6151.S.adenosyl.L.methioninesalvageI	HIV	Pos	-0.423041281	130	0.115384615	0.041208585	0.125356801
ANAGLYCOLYSIS.PWY.glycolysisIII.fromglucose.	HIV	Pos	-0.425232782	130	0.115384615	0.0242756	0.079541327
PWY.7219.adenosineribonucleotidesdenovobiosynthesis	HIV	Pos	-0.42779552	130	0.107692308	0.028520073	0.091791647
PWY.6122.5.aminoimidazoleribonucleotidebiosynthesisII	HIV	Pos	-0.435720253	130	0.107692308	0.042485196	0.128517718
PWY.6277.superpathwayof5.aminoimidazoleribonucleotidebiosynthesis	HIV	Pos	-0.435720253	130	0.107692308	0.042485196	0.128517718
PWY.6936.seleno.aminoacidbiosynthesis.plants.	HIV	Pos	-0.437592397	130	0.130769231	0.024561496	0.08037445
PWY.5484.glycolysisII.fromfructose6.phosphate.	HIV	Pos	-0.439475883	130	0.115384615	0.021462483	0.0711496
PWY.5861.superpathwayofdemethylmenaquinol.8biosynthesisI	HIV	Pos	-0.449120104	130	0.146153846	0.046378689	0.138339232
NONOXIPENT.PWY.pentosephosphatepathway.non.oxidativebranch.I	HIV	Pos	-0.464096952	130	0.138461538	0.038239319	0.117848525
PWY.3841.folatetransformationsII.plants.	HIV	Pos	-0.485295792	130	0.184615385	0.049038728	0.14506101
PWY0.1479.tRNAprocessing	HIV	Pos	-0.485306107	130	0.138461538	0.019388037	0.064950563
PWY.4242	HIV	Pos	-0.494812593	130	0.138461538	0.045896606	0.137284609
NAGLIPASYN.PWY.lipidIVAbiosynthesis.E.coli.	HIV	Pos	-0.496912754	130	0.146153846	0.049113007	0.145196221
PWY.6317.D.galactosedegradationI.Leloirpathway.	HIV	Pos	-0.508772506	130	0.107692308	0.005595354	0.021723138
PWY.6609.adenineandadenosinesalvageIII	HIV	Pos	-0.513565302	130	0.153846154	0.017270783	0.05870911
PWY.6386.UDP.N.acetylmuramoyl.pentapeptidebiosynthesisII.lysine.containing.	HIV	Pos	-0.516126393	130	0.115384615	0.006886136	0.026253069
PWY.6897.thiaminediphosphatesalvageII	HIV	Pos	-0.521144354	130	0.184615385	0.030298543	0.096537428
PEPTIDOGLYCANSYN.PWY.peptidoglycanbiosynthesisI.meso.diaminopimelatecontaining.	HIV	Pos	-0.532237893	130	0.146153846	0.036254936	0.112551976
PEPTIDOGLYCANSYN.PWY.peptidoglycanbiosynthesisI.meso.diaminopimelatecontaining.	HIV	Pos	-0.533613212	130	0.2	0.048425247	0.143580575
PWY.6385.peptidoglycanbiosynthesisIII.mycobacteria.	HIV	Pos	-0.537531602	130	0.146153846	0.03625382	0.112551976
PWY.6387.UDP.N.acetylmuramoyl.pentapeptidebiosynthesisI.meso.diaminopimelatecontaining.	HIV	Pos	-0.545678481	130	0.146153846	0.034618835	0.108199827
PWY.7221.guanosineribonucleotidesdenovobiosynthesis	HIV	Pos	-0.54573822	130	0.123076923	0.032975037	0.104086423
NONOXIPENT.PWY.pentosephosphatepathway.non.oxidativebranch.I	HIV	Pos	-0.552040937	130	0.253846154	0.037428852	0.115701597
PWY.3841.folatetransformationsII.plants.	HIV	Pos	-0.563939104	130	0.2	0.036789675	0.113933656
PWY.5695.inosine5..phosphatedegradation	HIV	Pos	-0.56734739	130	0.2	0.033056547	0.104214251
NONMEVIPP.PWY.methylerythritolphosphatepathwayI	HIV	Pos	-0.568978971	130	0.138461538	0.022858111	0.075431768
PWY.6387.UDP.N.acetylmuramoyl.pentapeptidebiosynthesisI.meso.diaminopimelatecontaining.	HIV	Pos	-0.569207302	130	0.207692308	0.035378873	0.110101305
ILEUSYN.PWY.L.isoleucinebiosynthesisI.fromthreonine.	HIV	Pos	-0.580617419	130	0.123076923	0.009264302	0.033969106
GLUTORN.PWY.L.ornithinebiosynthesisI	HIV	Pos	-0.587947473	130	0.123076923	0.002833304	0.01198905
BRANCHED.CHAIN.AA.SYN.PWY.superpathwayofbranchedchainaminoacidbiosynthesis	HIV	Pos	-0.589875729	130	0.123076923	0.00837819	0.031169809
PWY.7220.adenosinedeoxyribonucleotidesdenovobiosynthesisII	HIV	Pos	-0.591144399	130	0.161538462	0.044417613	0.133647312
PWY.7222.guanosinedeoxyribonucleotidesdenovobiosynthesisII	HIV	Pos	-0.591144399	130	0.161538462	0.044417613	0.133647312
PWY.7357.thiaminephosphateformationfrompyrithiamineandoxythiamine.yeast.	HIV	Pos	-0.606196855	130	0.207692308	0.037128515	0.11484304
PWY.5103.L.isoleucinebiosynthesisIII	HIV	Pos	-0.615013768	130	0.123076923	0.007674838	0.02891292
VALSYN.PWY.L.valinebiosynthesis	HIV	Pos	-0.637767158	130	0.138461538	0.004539379	0.017949251
PWY.7111.pyruvatefermentationtoisobutanol.engineered.	HIV	Pos	-0.639983938	130	0.115384615	0.002061561	0.009287992
VALSYN.PWY.L.valinebiosynthesis	HIV	Pos	-0.639983938	130	0.115384615	0.002061561	0.009287992
ARGSYNBSUB.PWY.L.argininebiosynthesisII.acetylcycle.	HIV	Pos	-0.643271551	130	0.130769231	0.008058596	0.030113078
HISTSYN.PWY.L.histidinebiosynthesis	HIV	Pos	-0.655246581	130	0.223076923	0.037671604	0.116310505

Supplementary Table 7.(Continuation)

PWY	metadata	value	coef	N	prevalence	pval	qval
HSERMETANA.PWY.L.methioninebiosynthesisIII	HIV	Pos	-0.668320249	130	0.107692308	0.00192305	0.008780719
PWY.6737.starchdegradationV	HIV	Pos	-0.688142167	130	0.138461538	0.021349184	0.070820206
PWY.7219.adenosineribonucleotidesdenovobiosynthesis	HIV	Pos	-0.712671572	130	0.146153846	0.044557768	0.133648689
PWY.7111.pyruvatefermentationtoisobutanol.engineered.	HIV	Pos	-0.71745483	130	0.153846154	0.001786134	0.008239425
PWY.7219.adenosineribonucleotidesdenovobiosynthesis	HIV	Pos	-0.728112852	130	0.115384615	0.001564635	0.007389844
PWY0.1586.peptidoglycanmaturation.meso.diaminopimelatecontaining.	HIV	Pos	-0.733451415	130	0.223076923	0.034184396	0.10717156
VALSYN.PWY.L.valinebiosynthesis	HIV	Pos	-0.753364748	130	0.330769231	0.015769304	0.054184991
UDPNAGSYN.PWY.UDP.N.acetyl.D.glucosaminebiosynthesisI	HIV	Pos	-0.785825739	130	0.130769231	0.001767367	0.008183335
PWY.7111.pyruvatefermentationtoisobutanol.engineered.	HIV	Pos	-0.789240464	130	0.307692308	0.012342843	0.044038882
PWY.6151.S.adenosyl.L.methioninesalvageI	HIV	Pos	-0.839445485	130	0.238461538	0.022968448	0.075697569
PWY.6700.queuosinebiosynthesisI.denovo.	HIV	Pos	-0.900907153	130	0.146153846	0.00242871	0.010558342
PWY.6609.adenineandadenosinesalvageIII	HIV	Pos	-0.93867104	130	0.130769231	2.01942E-05	0.000264503
PWY.6936.seleno.aminoacidbiosynthesis.plants.	HIV	Pos	-0.940383827	130	0.261538462	0.011006174	0.039725411
PWY.1042.glycolysisIV	HIV	Pos	-0.978059507	130	0.238461538	0.019620969	0.065644348
VALSYN.PWY.L.valinebiosynthesis	HIV	Pos	-1.130376587	130	0.315384615	0.038268424	0.117866747
COA.PWY.coenzymeAbiosynthesisI.prokaryotic.	lesions	yes	1.117051173	130	0.253846154	0.009998699	0.036166111
HISDEG.PWY.L.histidinedegradationI	lesions	yes	1.10271986	130	0.253846154	0.008160528	0.030697114
PWY.7111.pyruvatefermentationtoisobutanol.engineered.	lesions	yes	0.970560011	130	0.223076923	0.007074242	0.027208082
PWY.6123.inosine.5..phosphatebiosynthesisI	lesions	yes	0.961384777	130	0.2	0.005967229	0.023822041
COA.PWY.1.superpathwayofcoenzymeAbiosynthesisIII.mammals.	lesions	yes	0.946164604	130	0.238461538	0.022391243	0.069512704
PWY.7111.pyruvatefermentationtoisobutanol.engineered.	lesions	yes	0.939835149	130	0.130769231	0.030518263	0.088929939
PWY.6609.adenineandadenosinesalvageIII	lesions	yes	0.912322975	130	0.230769231	0.034019652	0.097291993
UDPNAGSYN.PWY.UDP.N.acetyl.D.glucosaminebiosynthesisI	lesions	yes	0.886116333	130	0.215384615	0.047201315	0.125262185
PWY.6387.UDP.N.acetylmuramoyl.pentapeptidebiosynthesisI.meso.diaminopimelatecontaining.	lesions	yes	0.883878712	130	0.215384615	0.03260823	0.093624307
UDPNAGSYN.PWY.UDP.N.acetyl.D.glucosaminebiosynthesisI	lesions	yes	0.879610432	130	0.238461538	0.027100383	0.081686921
PWY.7357.thiaminephosphateformationfrompyrithiamineandoxythiamine.yeast.	lesions	yes	0.865859819	130	0.246153846	0.038192483	0.105257158
PWY.6385.peptidoglycanbiosynthesisIII.mycobacteria.	lesions	yes	0.850553681	130	0.207692308	0.042817274	0.115558889
FASYN.INITIAL.PWY.superpathwayoffattyacidbiosynthesisinitiation.E.coli.	lesions	yes	0.846793122	130	0.169230769	0.009323942	0.03421247
PWY.6122.5.aminoimidazoleribonucleotidebiosynthesisII	lesions	yes	0.822751016	130	0.192307692	0.002431695	0.011485011
PWY.6277.superpathwayof5.aminoimidazoleribonucleotidebiosynthesis	lesions	yes	0.822751016	130	0.192307692	0.002431695	0.011485011
PEPTIDOGLYCANSYN.PWY.peptidoglycanbiosynthesisI.meso.diaminopimelatecontaining.	lesions	yes	0.821476538	130	0.207692308	0.043367328	0.116795316
DTDPRHAMSYN.PWY.dTDP..beta..L.rhamnosebiosynthesis	lesions	yes	0.805447481	130	0.230769231	0.049327577	0.130157188
NONOXIPENT.PWY.pentosephosphatepathway.non.oxidativebranch.I	lesions	yes	0.784116831	130	0.153846154	0.006760087	0.026205004
PWY.6124.inosine.5..phosphatebiosynthesisII	lesions	yes	0.782024667	130	0.169230769	0.017943631	0.05797173
VALSYN.PWY.L.valinebiosynthesis	lesions	yes	0.781812843	130	0.176923077	0.011932417	0.041736022
PWY.5173	lesions	yes	0.778266028	130	0.146153846	0.011135617	0.039491421
PWY.6700.queuosinebiosynthesisI.denovo.	lesions	yes	0.772035423	130	0.246153846	0.021545168	0.067380027
PWY.6123.inosine.5..phosphatebiosynthesisI	lesions	yes	0.770882508	130	0.169230769	0.005572149	0.022581867
NONMEVIPP.PWY.methylerythritolphosphatepathwayI	lesions	yes	0.768248337	130	0.184615385	0.047028475	0.124868709
PWY.6703.preQ0biosynthesis	lesions	yes	0.756536925	130	0.207692308	0.031731748	0.091625421
PWY.5913.partialTCAcycle.obligateautotrophs.	lesions	yes	0.755943393	130	0.146153846	0.008024467	0.030355511
PWY.5667.CDP.diacylglycerolbiosynthesisI	lesions	yes	0.744853598	130	0.146153846	0.037665467	0.104370723
PWY0.1319.CDP.diacylglycerolbiosynthesisII	lesions	yes	0.744853598	130	0.146153846	0.037665467	0.104370723
PWY.1042.glycolysisIV	lesions	yes	0.742719353	130	0.215384615	0.034470851	0.09808559
PWY.7219.adenosineribonucleotidesdenovobiosynthesis	lesions	yes	0.740666934	130	0.238461538	0.022322107	0.069425306
PWY.6386.UDP.N.acetylmuramoyl.pentapeptidebiosynthesisII.lysine.containing.	lesions	yes	0.740326297	130	0.138461538	0.018262651	0.058852753
TRPSYN.PWY.L.tryptophanbiosynthesis	lesions	yes	0.739323712	130	0.207692308	0.030213278	0.088410879
HEMESYN2.PWY.hemebbiosynthesisII.oxygen.independent.	lesions	yes	0.735264542	130	0.130769231	0.019578398	0.062498379
HSERMETANA.PWY.L.methioninebiosynthesisIII	lesions	yes	0.731658526	130	0.184615385	0.02089474	0.065669183
PWY.6122.5.aminoimidazoleribonucleotidebiosynthesisII	lesions	yes	0.729649176	130	0.223076923	0.041169024	0.112122713
PWY.6277.superpathwayof5.aminoimidazoleribonucleotidebiosynthesis	lesions	yes	0.729649176	130	0.223076923	0.041169024	0.112122713
VALSYN.PWY.L.valinebiosynthesis	lesions	yes	0.702350619	130	0.161538462	0.034422215	0.098002072
PWY.6151.S.adenosyl.L.methioninesalvageI	lesions	yes	0.702071604	130	0.215384615	0.035178854	0.09965381
PWY.5686.UMPbiosynthesisI	lesions	yes	0.700364836	130	0.169230769	0.005989075	0.023871749
PEPTIDOGLYCANSYN.PWY.peptidoglycanbiosynthesisI.meso.diaminopimelatecontaining.	lesions	yes	0.694059091	130	0.169230769	0.007001653	0.027017769
PWY.6387.UDP.N.acetylmuramoyl.pentapeptidebiosynthesisI.meso.diaminopimelatecontaining.	lesions	yes	0.688767451	130	0.169230769	0.006106596	0.024226167
PWY.6385.peptidoglycanbiosynthesisIII.mycobacteria.	lesions	yes	0.688325177	130	0.161538462	0.007320128	0.028033827
PWY.6803.phosphatidylcholineacylediting	lesions	yes	0.682657864	130	0.138461538	0.015102198	0.050213727
PWY.5913.partialTCAcycle.obligateautotrophs.	lesions	yes	0.676378967	130	0.161538462	0.009432012	0.03453421
PWY.6121.5.aminoimidazoleribonucleotidebiosynthesisI	lesions	yes	0.673748664	130	0.169230769	0.004441676	0.018624256
UDPNAGSYN.PWY.UDP.N.acetyl.D.glucosaminebiosynthesisI	lesions	yes	0.670992212	130	0.215384615	0.044023247	0.118373619
VALSYN.PWY.L.valinebiosynthesis	lesions	yes	0.65256109	130	0.184615385	0.021922256	0.06826526
PANTO.PWY.phosphopantothenatebiosynthesisI	lesions	yes	0.649721033	130	0.130769231	0.019798743	0.063082892
PWY.6700.queuosinebiosynthesisI.denovo.	lesions	yes	0.647557651	130	0.176923077	0.012985536	0.044619671
SER.GLYSYN.PWY.superpathwayofL.serineandglycinebiosynthesisI	lesions	yes	0.642644688	130	0.115384615	0.011202431	0.039617784
TCA.TCAcycleI.prokaryotic.	lesions	yes	0.638472275	130	0.123076923	0.036272325	0.101786833
PWY.7220.adenosinedeoxyribonucleotidesdenovobiosynthesisII	lesions	yes	0.63267135	130	0.161538462	0.032230305	0.092643896
PWY.7222.guanosinedeoxyribonucleotidesdenovobiosynthesisII	lesions	yes	0.63267135	130	0.161538462	0.032230305	0.092643896
PWY.5100.pyruvatefermentationtoacetateandlactateII	lesions	yes	0.627667312	130	0.130769231	0.044787263	0.119920375
PWY.7111.pyruvatefermentationtoisobutanol.engineered.	lesions	yes	0.627438621	130	0.2	0.027340986	0.082168475
PWY.1269.CMP.3.deoxy.D.manno.octulosonatebiosynthesis	lesions	yes	0.627426377	130	0.138461538	0.021831152	0.068023247
NAGLIPASYN.PWY.lipidIVAbiosynthesis.E.coli.	lesions	yes	0.626624271	130	0.146153846	0.013823508	0.046927902
PWY.7219.adenosineribonucleotidesdenovobiosynthesis	lesions	yes	0.622877864	130	0.223076923	0.019006479	0.060940648
SO4ASSIM.PWY.assimilatorysulfatereductionI	lesions	yes	0.620610073	130	0.123076923	0.027313681	0.082134986
PWY.6703.preQ0biosynthesis	lesions	yes	0.610567556	130	0.146153846	0.011651774	0.041035561
PWY.5173	lesions	yes	0.60413577	130	0.161538462	0.01129973	0.039906343
COA.PWY.coenzymeAbiosynthesisI.prokaryotic.	lesions	yes	0.604034913	130	0.107692308	0.041849477	0.113602482
PWY.6124.inosine.5..phosphatebiosynthesisII	lesions	yes	0.595895713	130	0.176923077	0.013606808	0.046254044
DTDPRHAMSYN.PWY.dTDP..beta..L.rhamnosebiosynthesis	lesions	yes	0.591470255	130	0.146153846	0.039564646	0.108450665
PWY.7234.inosine.5..phosphatebiosynthesisIII	lesions	yes	0.586735891	130	0.123076923	0.017286123	0.056130533

Supplementary Table 7.(Continuation)

PWY	metadata	value	coef	N	prevalence	pval	qval
PWY.7199.pyrimidinedeoxyribonucleosidessalvage	lesions	yes	0.580937583	130	0.176923077	0.044480238	0.119255978
PWY66.389.phytoldegradation	lesions	yes	0.579704516	130	0.115384615	0.045596907	0.121895572
PWY.5837.2.carboxy.1.4.naphthoquinolbiosynthesis	lesions	yes	0.570555497	130	0.153846154	0.016623933	0.054260005
PWY.6168.flavinbiosynthesisIII.fungi.	lesions	yes	0.567171562	130	0.138461538	0.023308318	0.071901207
PWY.5667.CDP.diacylglycerolbiosynthesisI	lesions	yes	0.562303709	130	0.176923077	0.037582651	0.104327383
PWY0.1319.CDP.diacylglycerolbiosynthesisII	lesions	yes	0.562303709	130	0.176923077	0.037582651	0.104327383
PWY.6122.5.aminoimidazoleribonucleotidebiosynthesisII	lesions	yes	0.56051146	130	0.2	0.044492195	0.119255978
PWY.6277.superpathwayof5.aminoimidazoleribonucleotidebiosynthesis	lesions	yes	0.56051146	130	0.2	0.044492195	0.119255978
PWY.7221.guanosineribonucleotidesdenovobiosynthesis	lesions	yes	0.557903264	130	0.123076923	0.027477854	0.082433562
PWY.7219.adenosineribonucleotidesdenovobiosynthesis	lesions	yes	0.557468916	130	0.115384615	0.040104934	0.109635974
PWY.7234.inosine.5..phosphatebiosynthesisIII	lesions	yes	0.557412373	130	0.123076923	0.037476975	0.104246299
PWY.7208.superpathwayofpyrimidinenucleobasessalvage	lesions	yes	0.556576263	130	0.115384615	0.013224123	0.045255887
THRESYN.PWY.superpathwayofL.threoninebiosynthesis	lesions	yes	0.55473988	130	0.161538462	0.031060742	0.089994692
NONMEVIPP.PWY.methylerythritolphosphatepathwayI	lesions	yes	0.553768241	130	0.138461538	0.028632111	0.085130302
RIBOSYN2.PWY.flavinbiosynthesisI.bacteriaandplants.	lesions	yes	0.551983979	130	0.192307692	0.044064732	0.118384405
PWY.6122.5.aminoimidazoleribonucleotidebiosynthesisII	lesions	yes	0.547797324	130	0.184615385	0.046738044	0.124162435
PWY.6277.superpathwayof5.aminoimidazoleribonucleotidebiosynthesis	lesions	yes	0.547797324	130	0.184615385	0.046738044	0.124162435
PWY.5861.superpathwayofdemethylmenaquinol.8biosynthesisI	lesions	yes	0.531590164	130	0.146153846	0.020452864	0.064720706
PWY.6700.queuosinebiosynthesisI.denovo.	lesions	yes	0.531481628	130	0.146153846	0.011886053	0.041629856
ARGSYNBSUB.PWY.L.argininebiosynthesisII.acetylcycle.	lesions	yes	0.527566672	130	0.107692308	0.035662988	0.100465248
THRESYN.PWY.superpathwayofL.threoninebiosynthesis	lesions	yes	0.526025079	130	0.146153846	0.045592523	0.121895572
PYRIDOXSYN.PWY.pyridoxal5..phosphatebiosynthesisI	lesions	yes	0.525686996	130	0.123076923	0.022665306	0.07027766
PWY.5973.cis.vaccenatebiosynthesis	lesions	yes	0.519108991	130	0.169230769	0.023930048	0.073570784
PWY.7663.gondoatebiosynthesis.anaerobic.	lesions	yes	0.514680175	130	0.130769231	0.021335109	0.066846501
PWY0.1586.peptidoglycanmaturation.meso.diaminopimelatecontaining.	lesions	yes	0.511492002	130	0.176923077	0.049192291	0.129935146
PWY0.1261.anhydromuropeptidesrecyclingI	lesions	yes	0.505999084	130	0.138461538	0.022468214	0.069709075
PWY.5913.partialTCAcycle.obligateautotrophs.	lesions	yes	0.505322374	130	0.138461538	0.029306685	0.086440263
PWY.5695.inosine5..phosphatedegradation	lesions	yes	0.502864996	130	0.130769231	0.049900013	0.13139475
PWY.3001.superpathwayofL.isoleucinebiosynthesisI	lesions	yes	0.50282644	130	0.115384615	0.023754152	0.073162788
PWY.5686.UMPbiosynthesisI	lesions	yes	0.501756325	130	0.153846154	0.025514369	0.077829547
DTDPRHAMSYN.PWY.dTDP..beta..L.rhamnosebiosynthesis	lesions	yes	0.486270079	130	0.130769231	0.041750007	0.113401141
ARGSYN.PWY.L.argininebiosynthesisI.viaL.ornithine.	lesions	yes	0.484827257	130	0.115384615	0.03559931	0.100341483
PWY.7400.L.argininebiosynthesisIV.archaebacteria.	lesions	yes	0.48453843	130	0.115384615	0.035486371	0.100138572
PWY.5686.UMPbiosynthesisI	lesions	yes	0.484517993	130	0.146153846	0.020438405	0.064715248
PWY.7220.adenosinedeoxyribonucleotidesdenovobiosynthesisII	lesions	yes	0.474884539	130	0.130769231	0.009953028	0.036077953
PWY.7222.guanosinedeoxyribonucleotidesdenovobiosynthesisII	lesions	yes	0.474884539	130	0.130769231	0.009953028	0.036077953
PWY.6123.inosine.5..phosphatebiosynthesisI	lesions	yes	0.472488817	130	0.146153846	0.038555506	0.106142515
PWY4FS.7.phosphatidylglycerolbiosynthesisI.plastidic.	lesions	yes	0.469849036	130	0.138461538	0.042530656	0.115029692
PWY4FS.8.phosphatidylglycerolbiosynthesisII.non.plastidic.	lesions	yes	0.469849036	130	0.138461538	0.042530656	0.115029692
CALVIN.PWY.Calvin.Benson.Basshamcycle	lesions	yes	0.466474856	130	0.123076923	0.040387033	0.110229271
PWY.7234.inosine.5..phosphatebiosynthesisIII	lesions	yes	0.459212543	130	0.169230769	0.039609224	0.108514326
NONOXIPENT.PWY.pentosephosphatepathway.non.oxidativebranch.I	lesions	yes	0.449420247	130	0.138461538	0.045805594	0.122196341
PWY.1269.CMP.3.deoxy.D.manno.octulosonatebiosynthesis	lesions	yes	0.442386884	130	0.107692308	0.03781192	0.104573804
PWY.6126.superpathwayofadenosinenucleotidesdenovobiosynthesisII	lesions	yes	0.434912031	130	0.115384615	0.027243876	0.082022145
PHOSLIPSYN.PWY.superpathwayofphospholipidbiosynthesisI.bacteria.	lesions	yes	0.432910907	130	0.138461538	0.049629019	0.130748924
PWY.1269.CMP.3.deoxy.D.manno.octulosonatebiosynthesis	lesions	yes	0.429347248	130	0.123076923	0.034325822	0.097782413
PWY.7229.superpathwayofadenosinenucleotidesdenovobiosynthesisI	lesions	yes	0.423288573	130	0.115384615	0.027178254	0.08187308
PWY.6936.seleno.aminoacidbiosynthesis.plants.	lesions	yes	0.418853672	130	0.130769231	0.032111397	0.092458991
PWY.5484.glycolysisII.fromfructose6.phosphate.	lesions	yes	0.404362977	130	0.115384615	0.03603191	0.101279959
PWY.6121.5.aminoimidazoleribonucleotidebiosynthesisI	lesions	yes	0.403637041	130	0.176923077	0.049236978	0.129985621
UDPNAGSYN.PWY.UDP.N.acetyl.D.glucosaminebiosynthesisI	lesions	yes	0.403063361	130	0.115384615	0.037608769	0.104327383
TCA.TCAcycleI.prokaryotic.	lesions	yes	0.398310992	130	0.138461538	0.039707799	0.108725773
GLYCOLYSIS.glycolysisI.fromglucose6.phosphate.	lesions	yes	0.39602078	130	0.115384615	0.036984497	0.103272096
ANAGLYCOLYSIS.PWY.glycolysisIII.fromglucose.	lesions	yes	0.39593543	130	0.115384615	0.037799195	0.104573804
PWY.6386.UDP.N.acetylmuramoyl.pentapeptidebiosynthesisII.lysine.containing.	lesions	yes	0.392984697	130	0.115384615	0.042590417	0.115130052
PWY.1042.glycolysisIV	lesions	yes	0.388336351	130	0.130769231	0.03091074	0.089647452
ARGSYNBSUB.PWY.L.argininebiosynthesisII.acetylcycle.	lesions	yes	0.383308238	130	0.138461538	0.031573982	0.091273594
X1CMET2.PWY.folatetransformationsIII.E.coli.	lesions	yes	0.381781354	130	0.107692308	0.016379431	0.053772784
PWY0.162.superpathwayofpyrimidineribonucleotidesdenovobiosynthesis	lesions	yes	0.370463931	130	0.107692308	0.018067372	0.058260396
TCA.TCAcycleI.prokaryotic.	lesions	yes	0.353596136	130	0.123076923	0.029628209	0.086984723
FERMENTATION.PWY.mixedacidfermentation	lesions	yes	0.298712186	130	0.107692308	0.043183358	0.116361518
SER.GLYSYN.PWY.superpathwayofL.serineandglycinebiosynthesisI	SW	Yes	0.909019816	130	0.230769231	0.007326393	0.028292347
PWY.6123.inosine.5..phosphatebiosynthesisI	SW	Yes	0.790379781	130	0.192307692	0.007477354	0.028727387
PWY0.862..5Z..dodecenoatebiosynthesisI	SW	Yes	0.771924341	130	0.207692308	0.003968634	0.01712105
PWY.7219.adenosineribonucleotidesdenovobiosynthesis	SW	Yes	0.770177446	130	0.207692308	0.018194868	0.05946387
PWY.5989.stearatebiosynthesisII.bacteriaandplants.	SW	Yes	0.761244952	130	0.192307692	0.007412912	0.028583017
TRPSYN.PWY.L.tryptophanbiosynthesis	SW	Yes	0.758961599	130	0.207692308	0.014617935	0.049932584
PWY.7221.guanosineribonucleotidesdenovobiosynthesis	SW	Yes	0.753750143	130	0.2	0.014359258	0.049339923
PWY.7208.superpathwayofpyrimidinenucleobasessalvage	SW	Yes	0.737955832	130	0.169230769	0.004486019	0.018982472
PWY.7199.pyrimidinedeoxyribonucleosidessalvage	SW	Yes	0.733671696	130	0.176923077	0.004601282	0.019373416
PWY.6124.inosine.5..phosphatebiosynthesisII	SW	Yes	0.71997349	130	0.230769231	0.005951512	0.023828582
PWY.7197.pyrimidinedeoxyribonucleotidephosphorylation	SW	Yes	0.715128655	130	0.153846154	0.00520322	0.021498183
PWY.6122.5.aminoimidazoleribonucleotidebiosynthesisII	SW	Yes	0.710119222	130	0.2	0.003558195	0.015615499
PWY.6277.superpathwayof5.aminoimidazoleribonucleotidebiosynthesis	SW	Yes	0.710119222	130	0.2	0.003558195	0.015615499
PWY.7388.octanoyl..acyl.carrierprotein.biosynthesis.mitochondria.yeast.	SW	Yes	0.704148663	130	0.176923077	0.005386561	0.021987552
PWY.6386.UDP.N.acetylmuramoyl.pentapeptidebiosynthesisII.lysine.containing.	SW	Yes	0.700058604	130	0.138461538	0.005420293	0.022089758
PWY.6700.queuosinebiosynthesisI.denovo.	SW	Yes	0.677952485	130	0.176923077	0.016971151	0.056004798
PWY.6387.UDP.N.acetylmuramoyl.pentapeptidebiosynthesisI.meso.diaminopimelatecontaining.	SW	Yes	0.677493188	130	0.138461538	0.005996929	0.023978278
PWY0.1586.peptidoglycanmaturation.meso.diaminopimelatecontaining.	SW	Yes	0.670772848	130	0.223076923	0.028836105	0.087804125
PWY.7663.gondoatebiosynthesis.anaerobic.	SW	Yes	0.661218034	130	0.253846154	0.007246852	0.028035987

Supplementary Table 7.(Continuation)

PWY	metadata	value	coef	N	prevalence	pval	qval
PWY.6527.stachyosedegradation	SW	Yes	0.659729333	130	0.2	0.015785951	0.052953271
MET.SAM.PWY.superpathwayofS.adenosyl.L.methioninebiosynthesis	SW	Yes	0.658402476	130	0.2	0.003808794	0.016515606
COA.PWY.coenzymeAbiosynthesisI.prokaryotic.	SW	Yes	0.657850554	130	0.107692308	0.007845142	0.029886816
PWY.5861.superpathwayofdemethylmenaquinol.8biosynthesisI	SW	Yes	0.657615946	130	0.146153846	0.001126374	0.006168355
PWY.6317.D.galactosedegradationI.Leloirpathway.	SW	Yes	0.653547841	130	0.161538462	0.003963326	0.01711268
PWY66.422	SW	Yes	0.653547841	130	0.161538462	0.003963326	0.01711268
PWY0.1296.purineribonucleosidesdegradation	SW	Yes	0.652850371	130	0.192307692	0.03438214	0.102541102
COA.PWY.1.superpathwayofcoenzymeAbiosynthesisIII.mammals.	SW	Yes	0.646965261	130	0.161538462	0.004756379	0.019894584
PWY.2942.L.lysinebiosynthesisIII	SW	Yes	0.646405186	130	0.146153846	0.006581995	0.025809954
PWY.7228.superpathwayofguanosinenucleotidesdenovobiosynthesisI	SW	Yes	0.643084869	130	0.161538462	0.01530135	0.051771945
PWY.5837.2.carboxy.1.4.naphthoquinolbiosynthesis	SW	Yes	0.641919661	130	0.153846154	0.001412554	0.007385391
PWY.7187.pyrimidinedeoxyribonucleotidesdenovobiosynthesisII	SW	Yes	0.640629763	130	0.153846154	0.004763865	0.01990951
FASYN.INITIAL.PWY.superpathwayoffattyacidbiosynthesisinitiation.E.coli.	SW	Yes	0.640368868	130	0.176923077	0.010481716	0.038212395
UDPNAGSYN.PWY.UDP.N.acetyl.D.glucosaminebiosynthesisI	SW	Yes	0.636702692	130	0.2	0.011587923	0.041367845
PWY.6609.adenineandadenosinesalvageIII	SW	Yes	0.630425331	130	0.161538462	0.005227317	0.021562683
HOMOSER.METSYN.PWY.L.methioninebiosynthesisI	SW	Yes	0.629805803	130	0.223076923	0.011718835	0.041763757
HSERMETANA.PWY.L.methioninebiosynthesisIII	SW	Yes	0.629324632	130	0.184615385	0.025095756	0.077435721
RIBOSYN2.PWY.flavinbiosynthesisI.bacteriaandplants.	SW	Yes	0.625777465	130	0.161538462	0.002890682	0.013093089
PWY.6126.superpathwayofadenosinenucleotidesdenovobiosynthesisII	SW	Yes	0.623564856	130	0.192307692	0.016266189	0.054029264
TRNA.CHARGING.PWY.tRNAcharging	SW	Yes	0.62353216	130	0.2	0.007186766	0.027837761
PWY4FS.7.phosphatidylglycerolbiosynthesisI.plastidic.	SW	Yes	0.621722069	130	0.138461538	0.002464999	0.01148224
PWY4FS.8.phosphatidylglycerolbiosynthesisII.non.plastidic.	SW	Yes	0.621722069	130	0.138461538	0.002464999	0.01148224
PWY.6282.palmitoleatebiosynthesisI.from.5Z..dodec.5.enoate.	SW	Yes	0.620714839	130	0.192307692	0.010341265	0.037754531
COA.PWY.coenzymeAbiosynthesisI.prokaryotic.	SW	Yes	0.62006414	130	0.169230769	0.002733189	0.012502308
PWY.6385.peptidoglycanbiosynthesisIII.mycobacteria.	SW	Yes	0.618046761	130	0.146153846	0.007698493	0.029505084
PWY.6387.UDP.N.acetylmuramoyl.pentapeptidebiosynthesisI.meso.diaminopimelatecontaining.	SW	Yes	0.617857076	130	0.146153846	0.008216416	0.031114624
GLUCONEO.PWY.gluconeogenesisI	SW	Yes	0.617376673	130	0.207692308	0.005666321	0.0229238
PWY.7219.adenosineribonucleotidesdenovobiosynthesis	SW	Yes	0.61732044	130	0.107692308	0.003303891	0.014689741
PEPTIDOGLYCANSYN.PWY.peptidoglycanbiosynthesisI.meso.diaminopimelatecontaining.	SW	Yes	0.614803754	130	0.146153846	0.007433062	0.028638987
PWY.7228.superpathwayofguanosinenucleotidesdenovobiosynthesisI	SW	Yes	0.614641652	130	0.130769231	0.034803706	0.103494694
METSYN.PWY.superpathwayofL.homoserineandL.methioninebiosynthesis	SW	Yes	0.61372562	130	0.223076923	0.010819861	0.039026849
PWY.7664.oleatebiosynthesisIV.anaerobic.	SW	Yes	0.613720882	130	0.192307692	0.010772996	0.038939093
THRESYN.PWY.superpathwayofL.threoninebiosynthesis	SW	Yes	0.607245604	130	0.153846154	0.004789975	0.020002179
PWY.6125.superpathwayofguanosinenucleotidesdenovobiosynthesisII	SW	Yes	0.606933486	130	0.161538462	0.015112255	0.051251568
THISYNARA.PWY.superpathwayofthiaminediphosphatebiosynthesisIII.eukaryotes.	SW	Yes	0.606242534	130	0.153846154	0.005745836	0.023174872
FASYN.ELONG.PWY.fattyacidelongation..saturated	SW	Yes	0.601288203	130	0.192307692	0.010213466	0.037395415
GLCMANNANAUT.PWY.superpathwayofN.acetylglucosamine.N.acetylmannosamineandN.acetyln	SW	Yes	0.599245234	130	0.184615385	0.006711161	0.026296161
HISDEG.PWY.L.histidinedegradationI	SW	Yes	0.596786309	130	0.115384615	0.008817902	0.032998954
PWY.5667.CDP.diacylglycerolbiosynthesisI	SW	Yes	0.596602076	130	0.169230769	0.020101677	0.064492882
PWY0.1319.CDP.diacylglycerolbiosynthesisII	SW	Yes	0.596602076	130	0.169230769	0.020101677	0.064492882
PWY.7282.4.amino.2.methyl.5.diphosphomethylpyrimidinebiosynthesisII	SW	Yes	0.592112368	130	0.153846154	0.004513176	0.019081497
PWY.6737.starchdegradationV	SW	Yes	0.590829818	130	0.153846154	0.007908859	0.030106982
PWY.7208.superpathwayofpyrimidinenucleobasessalvage	SW	Yes	0.588462624	130	0.107692308	0.023207877	0.072357319
PHOSLIPSYN.PWY.superpathwayofphospholipidbiosynthesisI.bacteria.	SW	Yes	0.587983488	130	0.138461538	0.002515098	0.011662161
PWY.5173	SW	Yes	0.586054577	130	0.176923077	0.003644588	0.015892684
PWY.5030.L.histidinedegradationIII	SW	Yes	0.585666962	130	0.115384615	0.006965551	0.027167253
PWY0.1241.ADP.L.glycero..beta..D.manno.heptosebiosynthesis	SW	Yes	0.584422998	130	0.123076923	0.023342075	0.072642025
PWY.7229.superpathwayofadenosinenucleotidesdenovobiosynthesisI	SW	Yes	0.582748958	130	0.192307692	0.016485562	0.05461514
PWY.5347.superpathwayofL.methioninebiosynthesis.transsulfuration.	SW	Yes	0.581882377	130	0.223076923	0.012738585	0.044797213
PWY.7221.guanosineribonucleotidesdenovobiosynthesis	SW	Yes	0.581129305	130	0.184615385	0.040251918	0.116492167
DENOVOPURINE2.PWY.superpathwayofpurinenucleotidesdenovobiosynthesisII	SW	Yes	0.579190084	130	0.153846154	0.003551392	0.015612609
PWY.1269.CMP.3.deoxy.D.manno.octulosonatebiosynthesis	SW	Yes	0.576466227	130	0.192307692	0.026427599	0.081200157
PWY.7219.adenosineribonucleotidesdenovobiosynthesis	SW	Yes	0.572112024	130	0.246153846	0.042243756	0.121358263
NONMEVIPP.PWY.methylerythritolphosphatepathwayI	SW	Yes	0.571369202	130	0.138461538	0.00710287	0.027575847
PWY.3001.superpathwayofL.isoleucinebiosynthesisI	SW	Yes	0.56830588	130	0.238461538	0.019716197	0.063417675
PWY.7228.superpathwayofguanosinenucleotidesdenovobiosynthesisI	SW	Yes	0.56613903	130	0.107692308	0.023616103	0.073404913
PWY.7111.pyruvatefermentationtoisobutanol.engineered.	SW	Yes	0.562320222	130	0.107692308	0.007716646	0.029530118
PWY.7221.guanosineribonucleotidesdenovobiosynthesis	SW	Yes	0.55915978	130	0.207692308	0.040876359	0.117963463
PENTOSE.P.PWY.pentosephosphatepathway	SW	Yes	0.551107128	130	0.238461538	0.022815965	0.071310416
PWY.7221.guanosineribonucleotidesdenovobiosynthesis	SW	Yes	0.548209633	130	0.130769231	0.031547821	0.095092541
NAGLIPASYN.PWY.lipidIVAbiosynthesis.E.coli.	SW	Yes	0.543612274	130	0.146153846	0.011436351	0.040929249
PWY.7115.C4photosyntheticcarbonassimilationcycle.NAD.MEtype	SW	Yes	0.543416493	130	0.169230769	0.009497119	0.035152483
PWY0.1261.anhydromuropeptidesrecyclingI	SW	Yes	0.543203807	130	0.153846154	0.005598115	0.022723338
PWY.5667.CDP.diacylglycerolbiosynthesisI	SW	Yes	0.53699676	130	0.176923077	0.020315312	0.064973202
PWY0.1319.CDP.diacylglycerolbiosynthesisII	SW	Yes	0.53699676	130	0.176923077	0.020315312	0.064973202
PWY0.1241.ADP.L.glycero..beta..D.manno.heptosebiosynthesis	SW	Yes	0.536774439	130	0.215384615	0.036983253	0.108640978
PWY.841.superpathwayofpurinenucleotidesdenovobiosynthesisI	SW	Yes	0.535618422	130	0.146153846	0.005323215	0.021816598
DTDPRHAMSYN.PWY.dTDP..beta..L.rhamnosebiosynthesis	SW	Yes	0.533781401	130	0.138461538	0.048584474	0.137094001
PWY.7219.adenosineribonucleotidesdenovobiosynthesis	SW	Yes	0.529284527	130	0.146153846	0.043534783	0.124434065
PEPTIDOGLYCANSYN.PWY.peptidoglycanbiosynthesisI.meso.diaminopimelatecontaining.	SW	Yes	0.525751596	130	0.123076923	0.020832969	0.066295021
THRESYN.PWY.superpathwayofL.threoninebiosynthesis	SW	Yes	0.521524378	130	0.161538462	0.039463377	0.114544257
PWY.5913.partialTCAcycle.obligateautotrophs.	SW	Yes	0.521480501	130	0.176923077	0.01053626	0.038356214
PWY.7220.adenosinedeoxyribonucleotidesdenovobiosynthesisII	SW	Yes	0.521198894	130	0.169230769	0.018432337	0.060085398
PWY.7222.guanosinedeoxyribonucleotidesdenovobiosynthesisII	SW	Yes	0.521198894	130	0.169230769	0.018432337	0.060085398
PPGPPMET.PWY.ppGppmetabolism	SW	Yes	0.520117208	130	0.161538462	0.014404896	0.049377303
NONMEVIPP.PWY.methylerythritolphosphatepathwayI	SW	Yes	0.516875896	130	0.146153846	0.007038552	0.0274099
HISDEG.PWY.L.histidinedegradationI	SW	Yes	0.514949632	130	0.115384615	0.038240585	0.111944054
PWY.6121.5.aminoimidazoleribonucleotidebiosynthesisI	SW	Yes	0.51342518	130	0.176923077	0.005761655	0.023207681
PWY.7234.inosine.5..phosphatebiosynthesisIII	SW	Yes	0.502815486	130	0.169230769	0.008543001	0.032231277
PWY.6897.thiaminediphosphatesalvageII	SW	Yes	0.501244326	130	0.184615385	0.023288123	0.072518529

Supplementary Table 7.(Continuation)

PWY	metadata	value	coef	N	prevalence	pval	qval
PWY.4242	SW	Yes	0.499367685	130	0.138461538	0.024604163	0.076150034
PWY.5686.UMPbiosynthesisI	SW	Yes	0.497610924	130	0.153846154	0.011180528	0.040126725
NONOXIPENT.PWY.pentosephosphatepathway.non.oxidativebranch.I	SW	Yes	0.49596274	130	0.253846154	0.044817462	0.127669473
PWY.7117.C4photosyntheticcarbonassimilationcycle.PEPCKtype	SW	Yes	0.49567825	130	0.176923077	0.009772856	0.036068013
PWY.6125.superpathwayofguanosinenucleotidesdenovobiosynthesisII	SW	Yes	0.495633558	130	0.169230769	0.019763449	0.063528052
PWY.6385.peptidoglycanbiosynthesisIII.mycobacteria.	SW	Yes	0.48914455	130	0.115384615	0.045630646	0.129705489
PWY.7228.superpathwayofguanosinenucleotidesdenovobiosynthesisI	SW	Yes	0.48677271	130	0.169230769	0.020888433	0.066429924
X1CMET2.PWY.folatetransformationsIII.E.coli.	SW	Yes	0.486056473	130	0.184615385	0.029589751	0.089829818
PWY.6387.UDP.N.acetylmuramoyl.pentapeptidebiosynthesisI.meso.diaminopimelatecontaining.	SW	Yes	0.483253163	130	0.115384615	0.047839083	0.135215917
HISTSYN.PWY.L.histidinebiosynthesis	SW	Yes	0.482472224	130	0.169230769	0.026469162	0.081229639
PWY.6700.queuosinebiosynthesisI.denovo.	SW	Yes	0.474190883	130	0.130769231	0.008868359	0.033163357
PWY.6126.superpathwayofadenosinenucleotidesdenovobiosynthesisII	SW	Yes	0.473752365	130	0.169230769	0.019988279	0.064210135
PWY0.1479.tRNAprocessing	SW	Yes	0.473155602	130	0.138461538	0.012062115	0.042807032
PEPTIDOGLYCANSYN.PWY.peptidoglycanbiosynthesisI.meso.diaminopimelatecontaining.	SW	Yes	0.470291761	130	0.115384615	0.034534666	0.102814981
PWY.2942.L.lysinebiosynthesisIII	SW	Yes	0.469746608	130	0.146153846	0.042868564	0.122736924
PWY.5097.L.lysinebiosynthesisVI	SW	Yes	0.467003416	130	0.130769231	0.015721377	0.052845099
COMPLETE.ARO.PWY.superpathwayofaromaticaminoacidbiosynthesis	SW	Yes	0.462182682	130	0.107692308	0.009125521	0.033975016
ARO.PWY.chorismatebiosynthesisI	SW	Yes	0.459907436	130	0.107692308	0.009084104	0.033845614
PWY.6163.chorismatebiosynthesisfrom3.dehydroquinate	SW	Yes	0.458669319	130	0.115384615	0.020820995	0.066295021
PWY0.862..5Z..dodecenoatebiosynthesisI	SW	Yes	0.45759253	130	0.107692308	0.015739153	0.052866079
PWY.7229.superpathwayofadenosinenucleotidesdenovobiosynthesisI	SW	Yes	0.454959001	130	0.169230769	0.021174138	0.067108377
PWY.7219.adenosineribonucleotidesdenovobiosynthesis	SW	Yes	0.453882691	130	0.107692308	0.041922787	0.120523291
PWY.3841.folatetransformationsII.plants.	SW	Yes	0.44565812	130	0.176923077	0.018551021	0.060278956
COA.PWY.coenzymeAbiosynthesisI.prokaryotic.	SW	Yes	0.443536358	130	0.153846154	0.014425005	0.049398837
DAPLYSINESYN.PWY.L.lysinebiosynthesisI	SW	Yes	0.442082986	130	0.130769231	0.015575233	0.052545321
PWY.6386.UDP.N.acetylmuramoyl.pentapeptidebiosynthesisII.lysine.containing.	SW	Yes	0.441956703	130	0.115384615	0.010420474	0.038016402
PWY.6122.5.aminoimidazoleribonucleotidebiosynthesisII	SW	Yes	0.44080932	130	0.169230769	0.024330736	0.075349666
PWY.6277.superpathwayof5.aminoimidazoleribonucleotidebiosynthesis	SW	Yes	0.44080932	130	0.169230769	0.024330736	0.075349666
X1CMET2.PWY.folatetransformationsIII.E.coli.	SW	Yes	0.438549766	130	0.169230769	0.027262184	0.083512006
THRESYN.PWY.superpathwayofL.threoninebiosynthesis	SW	Yes	0.434248464	130	0.253846154	0.041929393	0.120523291
PWY.6385.peptidoglycanbiosynthesisIII.mycobacteria.	SW	Yes	0.434097555	130	0.161538462	0.015052003	0.051166741
PWY.5097.L.lysinebiosynthesisVI	SW	Yes	0.433706946	130	0.130769231	0.012604367	0.044390433
PWY.6168.flavinbiosynthesisIII.fungi.	SW	Yes	0.432537006	130	0.138461538	0.040844716	0.117939119
PWY.724.superpathwayofL.lysine.L.threonineandL.methioninebiosynthesisII	SW	Yes	0.431736698	130	0.130769231	0.018286196	0.05968558
PWY.5695.inosine5..phosphatedegradation	SW	Yes	0.428270742	130	0.138461538	0.035008227	0.103981185
PWY.6387.UDP.N.acetylmuramoyl.pentapeptidebiosynthesisI.meso.diaminopimelatecontaining.	SW	Yes	0.420760813	130	0.115384615	0.044847927	0.127684685
PWY.6386.UDP.N.acetylmuramoyl.pentapeptidebiosynthesisII.lysine.containing.	SW	Yes	0.419717195	130	0.161538462	0.016187791	0.053839236
PWY.6387.UDP.N.acetylmuramoyl.pentapeptidebiosynthesisI.meso.diaminopimelatecontaining.	SW	Yes	0.417923067	130	0.169230769	0.022178906	0.069834698
PEPTIDOGLYCANSYN.PWY.peptidoglycanbiosynthesisI.meso.diaminopimelatecontaining.	SW	Yes	0.412794322	130	0.169230769	0.02223677	0.069843797
PWY.7111.pyruvatefermentationtoisobutanol.engineered.	SW	Yes	0.409850772	130	0.161538462	0.036071371	0.106331035
VALSYN.PWY.L.valinebiosynthesis	SW	Yes	0.409850772	130	0.161538462	0.036071371	0.106331035
PWY.2942.L.lysinebiosynthesisIII	SW	Yes	0.407303947	130	0.169230769	0.020136141	0.064562692
PWY.5484.glycolysisII.fromfructose6.phosphate.	SW	Yes	0.401275066	130	0.115384615	0.012932734	0.045358283
GLCMANNANAUT.PWY.superpathwayofN.acetylglucosamine.N.acetylmannosamineandN.acetyln	SW	Yes	0.399615573	130	0.138461538	0.022803886	0.071310416
PWY.5097.L.lysinebiosynthesisVI	SW	Yes	0.39908859	130	0.169230769	0.020153208	0.064576672
ANAGLYCOLYSIS.PWY.glycolysisIII.fromglucose.	SW	Yes	0.394402746	130	0.115384615	0.013169346	0.045934535
PWY.6703.preQ0biosynthesis	SW	Yes	0.393509229	130	0.169230769	0.0256952	0.079045402
GLYCOLYSIS.glycolysisI.fromglucose6.phosphate.	SW	Yes	0.39293539	130	0.115384615	0.013025894	0.045653512
PWY.1269.CMP.3.deoxy.D.manno.octulosonatebiosynthesis	SW	Yes	0.39288257	130	0.107692308	0.026083052	0.080190002
PWY.724.superpathwayofL.lysine.L.threonineandL.methioninebiosynthesisII	SW	Yes	0.391831776	130	0.130769231	0.010232999	0.037439956
HISDEG.PWY.L.histidinedegradationI	SW	Yes	0.390320868	130	0.161538462	0.033592308	0.100598769
PWY.6126.superpathwayofadenosinenucleotidesdenovobiosynthesisII	SW	Yes	0.387285455	130	0.115384615	0.018257747	0.059631021
PWY.6151.S.adenosyl.L.methioninesalvageI	SW	Yes	0.384681041	130	0.115384615	0.044732404	0.127498641
UDPNAGSYN.PWY.UDP.N.acetyl.D.glucosaminebiosynthesisI	SW	Yes	0.383912375	130	0.115384615	0.018484701	0.060217469
PWY.7663.gondoatebiosynthesis.anaerobic.	SW	Yes	0.382130101	130	0.130769231	0.044533187	0.127144749
PWY.6700.queuosinebiosynthesisI.denovo.	SW	Yes	0.381783846	130	0.184615385	0.023886465	0.07410929
PWY.7229.superpathwayofadenosinenucleotidesdenovobiosynthesisI	SW	Yes	0.376963575	130	0.115384615	0.017659673	0.058013225
PWY.6163.chorismatebiosynthesisfrom3.dehydroquinate	SW	Yes	0.366732199	130	0.138461538	0.034951453	0.103873266
FERMENTATION.PWY.mixedacidfermentation	SW	Yes	0.357692647	130	0.107692308	0.006202235	0.024586395
PEPTIDOGLYCANSYN.PWY.peptidoglycanbiosynthesisI.meso.diaminopimelatecontaining.	SW	Yes	0.349224665	130	0.107692308	0.046796719	0.13260666
PWY.6385.peptidoglycanbiosynthesisIII.mycobacteria.	SW	Yes	0.345893921	130	0.107692308	0.049089735	0.138059786
PWY.6124.inosine.5..phosphatebiosynthesisII	SW	Yes	0.338412814	130	0.107692308	0.01554587	0.052494425
PWY0.1479.tRNAprocessing	SW	Yes	0.309321045	130	0.107692308	0.039755096	0.115251225
PWY.7219.adenosineribonucleotidesdenovobiosynthesis	SW	Yes	-0.516362569	130	0.130769231	0.046502998	0.132026947
PWY.7221.guanosineribonucleotidesdenovobiosynthesis	VL	Yes	1.258149971	130	0.146153846	0.031673982	0.091273594
PWY.6700.queuosinebiosynthesisI.denovo.	VL	Yes	1.159266595	130	0.123076923	0.032230305	0.092643896
PWY.5659.GDP.mannosebiosynthesis	VL	Yes	1.15291477	130	0.153846154	0.042561777	0.131090274
PWY.6123.inosine.5..phosphatebiosynthesisI	VL	Yes	1.147105151	130	0.153846154	0.046478527	0.139600399
PWY.5686.UMPbiosynthesisI	VL	Yes	1.137518267	130	0.138461538	0.043446316	0.133088715
PWY.7237.myo..chiro.andscyllo.inositoldegradation	VL	Yes	0.676522887	130	0.146153846	0.025401944	0.094150161
PWY.7539.6.hydroxymethyl.dihydropterindiphosphatebiosynthesisIII.Chlamydia.	VL	Yes	0.535254525	130	0.107692308	0.047063201	0.140488932
TCA.TCAcycleI.prokaryotic.	VL	Yes	-0.509117782	130	0.123076923	0.046735942	0.140125108
PWY.3841.folatetransformationsII.plants.	VL	Yes	-0.54193479	130	0.123076923	0.048093494	0.141842622
PWY.7197.pyrimidinedeoxyribonucleotidephosphorylation	VL	Yes	-0.562649622	130	0.130769231	0.049674901	0.144214708
PWY.6700.queuosinebiosynthesisI.denovo.	VL	Yes	-0.57582146	130	0.146153846	0.04253128	0.131090274
COA.PWY.coenzymeAbiosynthesisI.prokaryotic.	VL	Yes	-0.614327133	130	0.115384615	0.048881099	0.142889702
COA.PWY.1.superpathwayofcoenzymeAbiosynthesisIII.mammals.	VL	Yes	-0.61708276	130	0.115384615	0.048775171	0.142826516
PPGPPMET.PWY.ppGppmetabolism	VL	Yes	-0.633552731	130	0.115384615	0.047481533	0.140912696
PEPTIDOGLYCANSYN.PWY.peptidoglycanbiosynthesisI.meso.diaminopimelatecontaining.	VL	Yes	-0.654421374	130	0.153846154	0.047623782	0.141039662
PWY.6385.peptidoglycanbiosynthesisIII.mycobacteria.	VL	Yes	-0.658502504	130	0.153846154	0.0498119	0.144397828

Supplementary Table 7.(Continuation)

PWY	metadata	value	coef	N	prevalence	pval	qval
PWY.3841.folatetransformationsII.plants.	VL	Yes	-0.678754158	130	0.123076923	0.029095058	0.103003192
PWY.5855.ubiquinol.7biosynthesis.earlydecarboxylation.	VL	Yes	-0.682689819	130	0.123076923	0.0399233	0.126292353
PWY.5856.ubiquinol.9biosynthesis.earlydecarboxylation.	VL	Yes	-0.682689819	130	0.123076923	0.039922996	0.126292353
PWY.5857.ubiquinol.10biosynthesis.earlydecarboxylation.	VL	Yes	-0.682689819	130	0.123076923	0.039922996	0.126292353
PWY.6708.ubiquinol.8biosynthesis.earlydecarboxylation.	VL	Yes	-0.682689819	130	0.123076923	0.039922996	0.126292353
PWY.6124.inosine.5..phosphatebiosynthesisII	VL	Yes	-0.731892876	130	0.176923077	0.034961615	0.116013664
DTDPRHAMSYN.PWY.dTDP..beta..L.rhamnosebiosynthesis	VL	Yes	-0.745479013	130	0.130769231	0.044313957	0.134771712
NONOXIPENT.PWY.pentosephosphatepathway.non.oxidativebranch.I	VL	Yes	-0.756532405	130	0.138461538	0.049983217	0.14444105
PWY.6700.queuosinebiosynthesisI.denovo.	VL	Yes	-0.786970737	130	0.161538462	0.029985275	0.105056993
PWY.6936.seleno.aminoacidbiosynthesis.plants.	VL	Yes	-0.817932403	130	0.253846154	0.044423313	0.134983126
PWY.7219.adenosineribonucleotidesdenovobiosynthesis	VL	Yes	-0.853563828	130	0.161538462	0.041520345	0.129890054
PWY.6703.preQ0biosynthesis	VL	Yes	-0.855343399	130	0.146153846	0.019648167	0.07938994
CALVIN.PWY.Calvin.Benson.Basshamcycle	VL	Yes	-0.8663963	130	0.123076923	0.035730545	0.117187887
PWY.6122.5.aminoimidazoleribonucleotidebiosynthesisII	VL	Yes	-0.867927922	130	0.184615385	0.027554577	0.099951721
PWY.6277.superpathwayof5.aminoimidazoleribonucleotidebiosynthesis	VL	Yes	-0.867927922	130	0.184615385	0.027554577	0.099951721
PWY.5695.inosine5..phosphatedegradation	VL	Yes	-0.870115017	130	0.115384615	0.048535996	0.142619214
ANAGLYCOLYSIS.PWY.glycolysisIII.fromglucose.	VL	Yes	-0.881958575	130	0.176923077	0.038835655	0.1236609
PWY.7111.pyruvatefermentationtoisobutanol.engineered.	VL	Yes	-0.882026823	130	0.176923077	0.045773972	0.138096365
PWY.6609.adenineandadenosinesalvageIII	VL	Yes	-0.948622987	130	0.115384615	0.047595297	0.141039662
PWY.6123.inosine.5..phosphatebiosynthesisI	VL	Yes	-0.968541961	130	0.169230769	0.018452105	0.075869647
PANTO.PWY.phosphopantothenatebiosynthesisI	VL	Yes	-0.969043433	130	0.130769231	0.022728439	0.087703819
PWY.7111.pyruvatefermentationtoisobutanol.engineered.	VL	Yes	-0.97512005	130	0.2	0.021276556	0.083238996
FASYN.INITIAL.PWY.superpathwayoffattyacidbiosynthesisinitiation.E.coli.	VL	Yes	-0.982268781	130	0.153846154	0.04139088	0.129725193
UDPNAGSYN.PWY.UDP.N.acetyl.D.glucosaminebiosynthesisI	VL	Yes	-0.99560991	130	0.215384615	0.04186189	0.130236992
GLYCOGENSYNTH.PWY.glycogenbiosynthesisI.fromADP.D.Glucose.	VL	Yes	-1.002821537	130	0.269230769	0.017786923	0.074032057
VALSYN.PWY.L.valinebiosynthesis	VL	Yes	-1.050076749	130	0.184615385	0.018643698	0.076410589
PWY.6897.thiaminediphosphatesalvageII	VL	Yes	-1.094019749	130	0.246153846	0.008764584	0.050414956
PWY0.1586.peptidoglycanmaturation.meso.diaminopimelatecontaining.	VL	Yes	-1.114670494	130	0.161538462	0.027174562	0.099085924
PWY0.1296.purineribonucleosidesdegradation	VL	Yes	-1.14468603	130	0.176923077	0.012396775	0.059510342

Supplementary Table 8.
Significant associations between gene families and HIV, viral load, and the presence of lesions

feature	metadata	value	coef	N	Prevalence	pval	qval
UniRef90_A0A015WBF1_Prevotella_bivia	HIV	Pos	0.992	130	0.377	0.031	0.090
UniRef90_A0A060QNB5_Streptococcus_oralis	HIV	Pos	1.394	130	0.246	0.015	0.043
UniRef90_A0A081Q966_Streptococcus_mitis	HIV	Pos	1.383	130	0.277	0.025	0.071
UniRef90_A0A081R0J5_Streptococcus_oralis	HIV	Pos	0.811	130	0.238	0.030	0.087
UniRef90_A0A088F5Q4_Prevotella_timonensis	HIV	Pos	0.847	130	0.254	0.022	0.065
UniRef90_A0A095ZH15_Prevotella_sp_AM42_24	HIV	Pos	-0.610	130	0.231	0.006	0.019
UniRef90_A0A096B5B1_Ruminococcus_torques	HIV	Pos	-1.000	130	0.300	0.030	0.086
UniRef90_A0A0B7LB97_Streptococcus_infantis	HIV	Pos	0.506	130	0.246	0.030	0.086
UniRef90_A0A0D0ISJ7_Prevotella_sp_AM42_24	HIV	Pos	-0.421	130	0.246	0.023	0.068
UniRef90_A0A0E2H0J4_Dorea_formicigenerans	HIV	Pos	-0.727	130	0.231	0.023	0.067
UniRef90_A0A0E2H2A6_Dorea_formicigenerans	HIV	Pos	-0.942	130	0.238	0.039	0.111
UniRef90_A0A0E2H2A6_Faecalibacterium_prausnitzii	HIV	Pos	-0.794	130	0.246	0.029	0.084
UniRef90_A0A0E9FLH2_Prevotella_sp_AM42_24	HIV	Pos	-0.657	130	0.246	0.038	0.107
UniRef90_A0A0E9G918_Haemophilus_haemolyticus	HIV	Pos	-1.175	130	0.246	0.028	0.081
UniRef90_A0A0G8GBQ2_Faecalibacterium_prausnitzii	HIV	Pos	-0.937	130	0.238	0.017	0.049
UniRef90_A0A0K9NBB8_Coprococcus_comes	HIV	Pos	-0.847	130	0.300	0.001	0.005
UniRef90_A0A0K9NBB8_Roseburia_inulinivorans	HIV	Pos	-0.803	130	0.300	0.025	0.071
UniRef90_A0A0M6WB40_Faecalibacterium_prausnitzii	HIV	Pos	-0.859	130	0.238	0.036	0.103
UniRef90_A0A0M9FJG7_Streptococcus_parasanguinis	HIV	Pos	1.356	130	0.246	0.018	0.052
UniRef90_A0A0S2W734_Coprococcus_comes	HIV	Pos	-0.708	130	0.254	0.014	0.042
UniRef90_A0A0S2W734_Fusicatenibacter_saccharivorans	HIV	Pos	-0.724	130	0.254	0.032	0.091
UniRef90_A0A0S2W764_Faecalibacterium_prausnitzii	HIV	Pos	-0.815	130	0.238	0.025	0.073
UniRef90_A0A0T8QTS3_Streptococcus_parasanguinis	HIV	Pos	1.240	130	0.300	0.030	0.085
UniRef90_A0A0T8YH35_Streptococcus_parasanguinis	HIV	Pos	0.722	130	0.246	0.046	0.129
UniRef90_A0A127SMK3_Dorea_longicatena	HIV	Pos	-0.863	130	0.308	0.049	0.136
UniRef90_A0A133Z424_Prevotella_melaninogenica	HIV	Pos	1.591	130	0.246	0.036	0.104
UniRef90_A0A135YTH6_Coprococcus_comes	HIV	Pos	-0.890	130	0.246	0.020	0.057
UniRef90_A0A135YTH6_Fusicatenibacter_saccharivorans	HIV	Pos	-0.626	130	0.269	0.044	0.124
UniRef90_A0A136WGK3_Eubacterium_rectale	HIV	Pos	-1.264	130	0.231	0.007	0.022
UniRef90_A0A136WGM7_Coprococcus_comes	HIV	Pos	-0.899	130	0.262	0.004	0.013
UniRef90_A0A136WGM7_Roseburia_inulinivorans	HIV	Pos	-0.677	130	0.285	0.034	0.098
UniRef90_A0A139PMN9_Streptococcus_mitis	HIV	Pos	-1.436	130	0.254	0.006	0.019
UniRef90_A0A173QTZ7_Faecalibacterium_prausnitzii	HIV	Pos	-0.840	130	0.231	0.008	0.025
UniRef90_A0A173QU89_Faecalibacterium_prausnitzii	HIV	Pos	-0.665	130	0.238	0.040	0.112
UniRef90_A0A173QWR1_Coprococcus_comes	HIV	Pos	-0.977	130	0.277	0.018	0.053
UniRef90_A0A173QWR1_Ruminococcus_torques	HIV	Pos	-0.617	130	0.277	0.046	0.128
UniRef90_A0A173S9W5_Coprococcus_comes	HIV	Pos	-0.943	130	0.254	0.006	0.019
UniRef90_A0A173SHZ4_Roseburia_inulinivorans	HIV	Pos	-0.706	130	0.246	0.027	0.077
UniRef90_A0A173SXT0_Coprococcus_comes	HIV	Pos	-0.765	130	0.231	0.019	0.055
UniRef90_A0A173T4W8_Faecalibacterium_prausnitzii	HIV	Pos	-0.743	130	0.262	0.029	0.084
UniRef90_A0A173TP13_Faecalibacterium_prausnitzii	HIV	Pos	-0.670	130	0.238	0.033	0.094
UniRef90_A0A173TP98_Faecalibacterium_prausnitzii	HIV	Pos	-0.672	130	0.254	0.037	0.106
UniRef90_A0A173U256_Faecalibacterium_prausnitzii	HIV	Pos	-0.605	130	0.246	0.050	0.138
UniRef90_A0A173UNL4_Ruminococcus_torques	HIV	Pos	-1.580	130	0.254	0.003	0.009
UniRef90_A0A173V6Z4_Dorea_longicatena	HIV	Pos	-0.636	130	0.308	0.018	0.053
UniRef90_A0A173VH24_Roseburia_inulinivorans	HIV	Pos	-0.918	130	0.262	0.006	0.018
UniRef90_A0A173VIP0_Faecalibacterium_prausnitzii	HIV	Pos	-0.684	130	0.246	0.038	0.107
UniRef90_A0A173X6V6_Fusicatenibacter_saccharivorans	HIV	Pos	-0.575	130	0.277	0.050	0.138
UniRef90_A0A173X6V6_Ruminococcus_torques	HIV	Pos	-0.675	130	0.285	0.026	0.075
UniRef90_A0A173YA79_Coprococcus_comes	HIV	Pos	-0.686	130	0.246	0.024	0.069
UniRef90_A0A173YA79_Eubacterium_rectale	HIV	Pos	-0.655	130	0.277	0.049	0.135
UniRef90_A0A173Z9H9_Roseburia_inulinivorans	HIV	Pos	-0.557	130	0.238	0.034	0.096
UniRef90_A0A174A414_Faecalibacterium_prausnitzii	HIV	Pos	-0.619	130	0.238	0.032	0.091

UniRef90_A0A174ARN5_Faecalibacterium_prausnitzii	HIV	Pos	-0.886	130	0.285	0.032	0.092
UniRef90_A0A174C1Q1_Dorea_longicatena	HIV	Pos	-0.722	130	0.269	0.030	0.087
UniRef90_A0A174C1Q1_Roseburia_inulinivorans	HIV	Pos	-0.904	130	0.262	0.007	0.020
UniRef90_A0A174CTY8_Coprococcus_comes	HIV	Pos	-1.007	130	0.300	0.002	0.006
UniRef90_A0A174CTY8_Roseburia_inulinivorans	HIV	Pos	-0.603	130	0.300	0.047	0.131
UniRef90_A0A174CTY8_Ruminococcus_torques	HIV	Pos	-0.730	130	0.292	0.027	0.077
UniRef90_A0A174DTK3_Coprococcus_comes	HIV	Pos	-1.018	130	0.231	0.010	0.029
UniRef90_A0A174EIN5_Coprococcus_comes	HIV	Pos	-1.025	130	0.231	0.017	0.049
UniRef90_A0A174ES80_Catenibacterium_mitsuokai	HIV	Pos	-0.880	130	0.300	0.040	0.112
UniRef90_A0A174FJF4_Faecalibacterium_prausnitzii	HIV	Pos	-0.781	130	0.315	0.040	0.113
UniRef90_A0A174FJF4_Ruminococcus_torques	HIV	Pos	-0.738	130	0.262	0.011	0.031
UniRef90_A0A174GBZ5_Fusicatenibacter_saccharivorans	HIV	Pos	-0.567	130	0.254	0.050	0.138
UniRef90_A0A174GBZ5_Roseburia_inulinivorans	HIV	Pos	-0.898	130	0.254	0.009	0.026
UniRef90_A0A174HEF7_Coprococcus_comes	HIV	Pos	-0.563	130	0.238	0.026	0.075
UniRef90_A0A174HEF7_Dorea_formicigenerans	HIV	Pos	-0.644	130	0.269	0.050	0.137
UniRef90_A0A174HEF7_Dorea_longicatena	HIV	Pos	-0.698	130	0.254	0.008	0.024
UniRef90_A0A174HEF7_Eubacterium_rectale	HIV	Pos	-0.661	130	0.262	0.026	0.075
UniRef90_A0A174HEF7_Roseburia_inulinivorans	HIV	Pos	-0.662	130	0.254	0.017	0.049
UniRef90_A0A174HP74_Catenibacterium_mitsuokai	HIV	Pos	-0.750	130	0.338	0.012	0.034
UniRef90_A0A174I2N9_Coprococcus_comes	HIV	Pos	-0.959	130	0.231	0.004	0.012
UniRef90_A0A174I2N9_Dorea_formicigenerans	HIV	Pos	-0.744	130	0.254	0.018	0.052
UniRef90_A0A174ISG2_Catenibacterium_mitsuokai	HIV	Pos	-1.003	130	0.238	0.007	0.021
UniRef90_A0A174JLN6_Prevotella_sp_AM42_24	HIV	Pos	-0.586	130	0.254	0.048	0.134
UniRef90_A0A174KJ41_Dorea_longicatena	HIV	Pos	-0.702	130	0.231	0.041	0.114
UniRef90_A0A174KSJ1_Catenibacterium_mitsuokai	HIV	Pos	-0.708	130	0.262	0.021	0.060
UniRef90_A0A174LAB2_Dorea_longicatena	HIV	Pos	-0.919	130	0.254	0.008	0.025
UniRef90_A0A174N166_Catenibacterium_mitsuokai	HIV	Pos	-0.696	130	0.262	0.048	0.133
UniRef90_A0A174N610_Prevotella_sp_AM42_24	HIV	Pos	-0.670	130	0.238	0.020	0.057
UniRef90_A0A174N8T0_Catenibacterium_mitsuokai	HIV	Pos	-0.720	130	0.292	0.037	0.105
UniRef90_A0A174PVT9_Eubacterium_rectale	HIV	Pos	-0.948	130	0.238	0.023	0.066
UniRef90_A0A174QW60_Prevotella_sp_AM42_24	HIV	Pos	-0.510	130	0.246	0.043	0.122
UniRef90_A0A174RDH8_Coprococcus_comes	HIV	Pos	-0.781	130	0.238	0.007	0.021
UniRef90_A0A174SA26_Prevotella_sp_AM42_24	HIV	Pos	-0.473	130	0.277	0.034	0.098
UniRef90_A0A174TQZ1_Coprococcus_comes	HIV	Pos	-0.684	130	0.254	0.034	0.098
UniRef90_A0A174TSL2_Eubacterium_rectale	HIV	Pos	-0.974	130	0.246	0.025	0.072
UniRef90_A0A174TSL2_Faecalibacterium_prausnitzii	HIV	Pos	-0.712	130	0.262	0.044	0.123
UniRef90_A0A174UAR2_Prevotella_sp_AM42_24	HIV	Pos	-1.063	130	0.231	0.004	0.011
UniRef90_A0A174UWU8_Prevotella_sp_AM42_24	HIV	Pos	-0.567	130	0.246	0.037	0.105
UniRef90_A0A174VDA7_Fusicatenibacter_saccharivorans	HIV	Pos	-1.034	130	0.254	0.006	0.019
UniRef90_A0A174VDA7_Ruminococcus_torques	HIV	Pos	-0.840	130	0.292	0.034	0.098
UniRef90_A0A174X3E1_Prevotella_sp_AM42_24	HIV	Pos	-1.045	130	0.254	0.025	0.072
UniRef90_A0A174XHC2_Prevotella_sp_AM42_24	HIV	Pos	-0.537	130	0.246	0.033	0.093
UniRef90_A0A174XK61_Faecalibacterium_prausnitzii	HIV	Pos	-0.893	130	0.238	0.024	0.070
UniRef90_A0A174Y9Z1_Coprococcus_comes	HIV	Pos	-1.057	130	0.246	0.003	0.010
UniRef90_A0A174YAF1_Ruminococcus_torques	HIV	Pos	-0.553	130	0.238	0.037	0.106
UniRef90_A0A174ZD99_Dorea_longicatena	HIV	Pos	-0.938	130	0.231	0.044	0.124
UniRef90_A0A174ZJF4_Coprococcus_comes	HIV	Pos	-0.729	130	0.308	0.030	0.085
UniRef90_A0A174ZJF4_Fusicatenibacter_saccharivorans	HIV	Pos	-0.845	130	0.285	0.026	0.075
UniRef90_A0A174ZJF4_Ruminococcus_torques	HIV	Pos	-0.749	130	0.285	0.015	0.044
UniRef90_A0A174ZSN8_Coprococcus_comes	HIV	Pos	-0.767	130	0.246	0.037	0.106
UniRef90_A0A176U3W6_Coprococcus_comes	HIV	Pos	-0.979	130	0.285	0.005	0.016
UniRef90_A0A176U3W6_Fusicatenibacter_saccharivorans	HIV	Pos	-0.833	130	0.277	0.025	0.073
UniRef90_A0A176U3W6_Ruminococcus_torques	HIV	Pos	-0.770	130	0.285	0.009	0.026
UniRef90_A0A176U927_Fusicatenibacter_saccharivorans	HIV	Pos	-0.754	130	0.231	0.031	0.090
UniRef90_A0A1B1I9V9_Bacteroides_vulgatus	HIV	Pos	-0.522	130	0.238	0.043	0.119
UniRef90_A0A1B1IAF4_Prevotella_copri	HIV	Pos	-1.158	130	0.231	0.032	0.093

UniRef90_A0A1E3AIV3_Ruminococcus_torques	HIV	Pos	-0.892	130	0.292	0.045	0.126
UniRef90_A0A1L5L421_Coprococcus_comes	HIV	Pos	-0.719	130	0.254	0.013	0.038
UniRef90_A0A1L5L540_Prevotella_sp_AM42_24	HIV	Pos	-0.674	130	0.262	0.038	0.107
UniRef90_A0A1X1KB82_Streptococcus_mitis	HIV	Pos	1.187	130	0.369	0.030	0.085
UniRef90_A0A1Y4MFI1_Coprococcus_comes	HIV	Pos	-0.617	130	0.254	0.039	0.109
UniRef90_A0A229I1V0_Prevotella_sp_AM42_24	HIV	Pos	-0.403	130	0.246	0.031	0.090
UniRef90_A0A229I2S7_Prevotella_sp_AM42_24	HIV	Pos	-0.730	130	0.238	0.018	0.053
UniRef90_A0A229I383_Prevotella_copri	HIV	Pos	-0.499	130	0.231	0.025	0.073
UniRef90_A0A229I3N1_Prevotella_copri	HIV	Pos	-0.613	130	0.246	0.020	0.059
UniRef90_A0A229I4I5_Prevotella_sp_AM42_24	HIV	Pos	-0.647	130	0.231	0.029	0.085
UniRef90_A0A229I4M0_Prevotella_sp_AM42_24	HIV	Pos	-0.570	130	0.246	0.046	0.128
UniRef90_A0A229I513_Prevotella_copri	HIV	Pos	-0.456	130	0.231	0.043	0.120
UniRef90_A0A229I5Y2_Prevotella_copri	HIV	Pos	-0.823	130	0.254	0.020	0.059
UniRef90_A0A229I5Y9_Prevotella_sp_AM42_24	HIV	Pos	-0.747	130	0.238	0.004	0.011
UniRef90_A0A229I616_Prevotella_sp_AM42_24	HIV	Pos	-0.602	130	0.238	0.039	0.110
UniRef90_A0A229I6C0_Prevotella_copri	HIV	Pos	-0.767	130	0.277	0.039	0.110
UniRef90_A0A229I7D9_Prevotella_sp_AM42_24	HIV	Pos	-0.590	130	0.269	0.039	0.111
UniRef90_A0A229I901_Prevotella_sp_AM42_24	HIV	Pos	-0.794	130	0.231	0.011	0.034
UniRef90_A0A229I939_Bacteroides_vulgatus	HIV	Pos	-0.499	130	0.238	0.050	0.138
UniRef90_A0A229I944_Prevotella_sp_AM42_24	HIV	Pos	-0.622	130	0.254	0.038	0.107
UniRef90_A0A229I9S0_Prevotella_sp_AM42_24	HIV	Pos	-0.367	130	0.254	0.044	0.122
UniRef90_A0A229IA06_Prevotella_sp_AM42_24	HIV	Pos	-1.078	130	0.246	0.020	0.058
UniRef90_A0A285PPQ3_Faecalibacterium_prausnitzii	HIV	Pos	-0.847	130	0.269	0.026	0.075
UniRef90_A0A2A6Z957_Faecalibacterium_prausnitzii	HIV	Pos	-0.816	130	0.238	0.005	0.016
UniRef90_A0A2A6ZAF7_Faecalibacterium_prausnitzii	HIV	Pos	-0.883	130	0.308	0.030	0.086
UniRef90_A0A2A6ZB25_Faecalibacterium_prausnitzii	HIV	Pos	-1.203	130	0.277	0.011	0.032
UniRef90_A0A2A6ZDF3_Faecalibacterium_prausnitzii	HIV	Pos	-0.599	130	0.231	0.046	0.128
UniRef90_A0A2A6ZE59_Faecalibacterium_prausnitzii	HIV	Pos	-0.759	130	0.315	0.027	0.077
UniRef90_A0A2A6ZFI5_Faecalibacterium_prausnitzii	HIV	Pos	-0.858	130	0.262	0.018	0.053
UniRef90_A0A2A6ZL01_Faecalibacterium_prausnitzii	HIV	Pos	-0.676	130	0.231	0.046	0.128
UniRef90_A0A2A6ZL20_Faecalibacterium_prausnitzii	HIV	Pos	-0.806	130	0.238	0.015	0.045
UniRef90_A0A2A6ZL21_Faecalibacterium_prausnitzii	HIV	Pos	-0.925	130	0.231	0.016	0.048
UniRef90_A0A2A6ZL80_Coprococcus_comes	HIV	Pos	-0.695	130	0.277	0.036	0.102
UniRef90_A0A2A6ZL80_Fusicatenibacter_saccharivorans	HIV	Pos	-0.721	130	0.262	0.039	0.111
UniRef90_A0A2A6ZL80_Roseburia_inulinivorans	HIV	Pos	-0.722	130	0.254	0.023	0.066
UniRef90_A0A2A7AG21_Faecalibacterium_prausnitzii	HIV	Pos	-0.686	130	0.262	0.048	0.134
UniRef90_A0A2A7ALX5_Faecalibacterium_prausnitzii	HIV	Pos	-0.549	130	0.277	0.046	0.127
UniRef90_A0A2A7APA7_Faecalibacterium_prausnitzii	HIV	Pos	-0.921	130	0.262	0.027	0.077
UniRef90_A0A2A7ATS6_Faecalibacterium_prausnitzii	HIV	Pos	-0.735	130	0.246	0.038	0.107
UniRef90_A0A2A7AZZ8_Faecalibacterium_prausnitzii	HIV	Pos	-0.624	130	0.246	0.027	0.079
UniRef90_A0A2A7B088_Faecalibacterium_prausnitzii	HIV	Pos	-0.661	130	0.254	0.026	0.075
UniRef90_A0A2A7B688_Faecalibacterium_prausnitzii	HIV	Pos	-0.806	130	0.246	0.049	0.135
UniRef90_A0A2I1TT40_Streptococcus_parasanguinis	HIV	Pos	2.392	130	0.246	0.001	0.002
UniRef90_A0A2I1TTC0_Streptococcus_parasanguinis	HIV	Pos	1.764	130	0.315	0.012	0.037
UniRef90_A0A2I7ZEY3_Coprococcus_comes	HIV	Pos	-1.059	130	0.254	0.002	0.007
UniRef90_A0A2J4JLE3_Faecalibacterium_prausnitzii	HIV	Pos	-0.806	130	0.238	0.022	0.063
UniRef90_A0A2J4JNX1_Faecalibacterium_prausnitzii	HIV	Pos	-0.839	130	0.246	0.037	0.105
UniRef90_A0A2U1C1B7_Faecalibacterium_prausnitzii	HIV	Pos	-0.719	130	0.246	0.044	0.123
UniRef90_A0A329U974_Faecalibacterium_prausnitzii	HIV	Pos	-0.731	130	0.231	0.017	0.049
UniRef90_A0A329UC40_Faecalibacterium_prausnitzii	HIV	Pos	-0.686	130	0.254	0.039	0.110
UniRef90_A0A329UIW4_Ruminococcus_torques	HIV	Pos	-0.597	130	0.308	0.019	0.056
UniRef90_A0A346FUW5_Coprococcus_comes	HIV	Pos	-0.648	130	0.285	0.043	0.120
UniRef90_A0A346FUW7_Coprococcus_comes	HIV	Pos	-0.760	130	0.285	0.005	0.014
UniRef90_A0A350TLZ2_Oscillibacter_sp_CAG_241	HIV	Pos	-0.818	130	0.231	0.042	0.117
UniRef90_A0A367G075_Faecalibacterium_prausnitzii	HIV	Pos	-0.571	130	0.231	0.047	0.131
UniRef90_A0A367G562_Faecalibacterium_prausnitzii	HIV	Pos	-0.767	130	0.246	0.037	0.106

UniRef90_A0A374BMQ1_Prevotella_sp_AM42_24	HIV	Pos	-0.561	130	0.238	0.048	0.134
UniRef90_A0A374BPV1_Prevotella_sp_AM42_24	HIV	Pos	-0.512	130	0.246	0.040	0.113
UniRef90_A0A374BQD1_Prevotella_sp_AM42_24	HIV	Pos	-0.679	130	0.231	0.011	0.032
UniRef90_A0A374BQR7_Prevotella_sp_AM42_24	HIV	Pos	-0.536	130	0.246	0.034	0.098
UniRef90_A0A374BWN3_Prevotella_sp_AM42_24	HIV	Pos	-0.392	130	0.246	0.036	0.102
UniRef90_A0A374C2I3_Prevotella_sp_AM42_24	HIV	Pos	-0.554	130	0.231	0.044	0.123
UniRef90_A0A374C8F4_Prevotella_sp_AM42_24	HIV	Pos	-0.495	130	0.246	0.030	0.087
UniRef90_A0A374CBG3_Prevotella_sp_AM42_24	HIV	Pos	-0.614	130	0.246	0.028	0.081
UniRef90_A0A374CIC9_Prevotella_sp_AM42_24	HIV	Pos	-0.488	130	0.238	0.028	0.080
UniRef90_A0A374NP07_Eubacterium_rectale	HIV	Pos	-0.988	130	0.231	0.016	0.047
UniRef90_A0A395UT51_Bacteroides_vulgatus	HIV	Pos	-0.387	130	0.238	0.047	0.132
UniRef90_A0A395V4X9_Roseburia_inulinivorans	HIV	Pos	-0.972	130	0.238	0.008	0.023
UniRef90_A0A395V6C3_Faecalibacterium_prausnitzii	HIV	Pos	-0.914	130	0.238	0.005	0.016
UniRef90_A0A395X5I7_Ruminococcus_torques	HIV	Pos	-0.717	130	0.254	0.014	0.041
UniRef90_A0A395X7M9_Coprococcus_comes	HIV	Pos	-0.510	130	0.231	0.032	0.091
UniRef90_A0A395X7M9_Dorea_formicigenerans	HIV	Pos	-0.756	130	0.238	0.012	0.035
UniRef90_A0A395X7M9_Dorea_longicatena	HIV	Pos	-0.816	130	0.246	0.020	0.057
UniRef90_A0A395X7M9_Eubacterium_rectale	HIV	Pos	-0.698	130	0.238	0.017	0.049
UniRef90_A0A395XBK7_Dorea_longicatena	HIV	Pos	-0.648	130	0.277	0.022	0.063
UniRef90_A0A395XBK7_Eubacterium_rectale	HIV	Pos	-0.801	130	0.277	0.012	0.035
UniRef90_A0A395ZWL7_Dorea_longicatena	HIV	Pos	-0.596	130	0.254	0.037	0.104
UniRef90_A0A396A1S7_Coprococcus_comes	HIV	Pos	-0.707	130	0.231	0.003	0.008
UniRef90_A0A396A1S7_Dorea_formicigenerans	HIV	Pos	-0.795	130	0.254	0.040	0.112
UniRef90_A0A396A9Y4_Coprococcus_comes	HIV	Pos	-0.747	130	0.262	0.011	0.034
UniRef90_A0A396ADT9_Dorea_formicigenerans	HIV	Pos	-0.842	130	0.285	0.030	0.087
UniRef90_A0A396ADT9_Roseburia_inulinivorans	HIV	Pos	-0.981	130	0.246	0.023	0.067
UniRef90_A0A396AE43_Eubacterium_rectale	HIV	Pos	-1.105	130	0.254	0.020	0.059
UniRef90_A0A396AE43_Ruminococcus_torques	HIV	Pos	-1.481	130	0.246	0.004	0.013
UniRef90_A0A396AFP6_Coprococcus_comes	HIV	Pos	-0.872	130	0.262	0.004	0.013
UniRef90_A0A396AFP6_Eubacterium_rectale	HIV	Pos	-0.852	130	0.292	0.028	0.081
UniRef90_A0A396AFP6_Ruminococcus_torques	HIV	Pos	-0.996	130	0.269	0.013	0.038
UniRef90_A0A396GB52_Dorea_formicigenerans	HIV	Pos	-1.047	130	0.231	0.024	0.070
UniRef90_A0A396GB52_Eubacterium_rectale	HIV	Pos	-0.966	130	0.246	0.047	0.130
UniRef90_A0A396GB52_Roseburia_inulinivorans	HIV	Pos	-0.927	130	0.238	0.001	0.004
UniRef90_A0A396GCB6_Coprococcus_comes	HIV	Pos	-0.755	130	0.254	0.015	0.043
UniRef90_A0A396GCB6_Eubacterium_rectale	HIV	Pos	-0.701	130	0.277	0.016	0.047
UniRef90_A0A396GCB6_Faecalibacterium_prausnitzii	HIV	Pos	-0.887	130	0.231	0.015	0.045
UniRef90_A0A396GCB6_Ruminococcus_torques	HIV	Pos	-0.706	130	0.246	0.035	0.101
UniRef90_A0A396GFV9_Roseburia_inulinivorans	HIV	Pos	-1.121	130	0.285	0.006	0.019
UniRef90_A0A396GFV9_Ruminococcus_torques	HIV	Pos	-1.257	130	0.292	0.024	0.070
UniRef90_A0A3E2UIN4_Faecalibacterium_prausnitzii	HIV	Pos	-0.595	130	0.238	0.022	0.065
UniRef90_A0A3E4GL34_Coprococcus_comes	HIV	Pos	-0.558	130	0.331	0.037	0.105
UniRef90_A0A3E4JFD6_Bacteroides_vulgatus	HIV	Pos	-0.590	130	0.254	0.018	0.052
UniRef90_A0A3E4S954_Prevotella_sp_AM42_24	HIV	Pos	-0.666	130	0.246	0.008	0.022
UniRef90_A0A3E4S9D1_Prevotella_sp_AM42_24	HIV	Pos	-0.513	130	0.238	0.035	0.101
UniRef90_A0A3E4SA83_Prevotella_copri	HIV	Pos	-0.688	130	0.308	0.040	0.114
UniRef90_A0A3E4SB08_Prevotella_copri	HIV	Pos	-0.912	130	0.262	0.039	0.109
UniRef90_A0A3E4SB18_Prevotella_copri	HIV	Pos	-0.933	130	0.254	0.030	0.086
UniRef90_A0A3E4SH63_Prevotella_sp_AM42_24	HIV	Pos	-0.414	130	0.254	0.047	0.130
UniRef90_A0A3E4SID5_Prevotella_sp_AM42_24	HIV	Pos	-0.608	130	0.238	0.036	0.102
UniRef90_A0A3E4SIW2_Prevotella_sp_AM42_24	HIV	Pos	-0.617	130	0.254	0.049	0.137
UniRef90_A0A3E4SKT8_Prevotella_sp_AM42_24	HIV	Pos	-0.504	130	0.238	0.038	0.107
UniRef90_A0A3E4SL07_Prevotella_sp_AM42_24	HIV	Pos	-0.758	130	0.238	0.015	0.043
UniRef90_A0A3E4SLM0_Prevotella_copri	HIV	Pos	-0.621	130	0.262	0.042	0.119
UniRef90_A0A3E4SLM6_Prevotella_sp_AM42_24	HIV	Pos	-0.640	130	0.231	0.027	0.079
UniRef90_A0A3E4TMC8_Faecalibacterium_prausnitzii	HIV	Pos	-1.041	130	0.292	0.029	0.084

UniRef90_A0A3E4VCI8_Dorea_formicigenerans	HIV	Pos	-0.693	130	0.254	0.037	0.104
UniRef90_A0A3E4VCI8_Dorea_longicatena	HIV	Pos	-0.729	130	0.246	0.036	0.103
UniRef90_A0A3E4VCI8_Eubacterium_rectale	HIV	Pos	-0.661	130	0.231	0.035	0.100
UniRef90_A0A3E4VCI8_Roseburia_inulinivorans	HIV	Pos	-0.773	130	0.238	0.006	0.019
UniRef90_A0A3E5A3B4_Prevotella_sp_AM42_24	HIV	Pos	-0.384	130	0.254	0.030	0.087
UniRef90_A0A3E5A5M2_Prevotella_sp_AM42_24	HIV	Pos	-0.607	130	0.254	0.045	0.125
UniRef90_A0A3E5ADY2_Prevotella_sp_AM42_24	HIV	Pos	-0.662	130	0.246	0.017	0.051
UniRef90_A0A3E5AFC8_Prevotella_sp_AM42_24	HIV	Pos	-0.898	130	0.262	0.041	0.116
UniRef90_A0A3E5AFK5_Prevotella_sp_AM42_24	HIV	Pos	-0.536	130	0.254	0.034	0.098
UniRef90_A0A3E5AG13_Prevotella_sp_AM42_24	HIV	Pos	-0.354	130	0.262	0.037	0.106
UniRef90_A0A3E5AH61_Prevotella_sp_AM42_24	HIV	Pos	-0.809	130	0.254	0.031	0.088
UniRef90_A0A3E5AHG7_Prevotella_sp_AM42_24	HIV	Pos	-0.563	130	0.269	0.030	0.086
UniRef90_A0A3E5AS08_Coprococcus_comes	HIV	Pos	-0.569	130	0.231	0.026	0.076
UniRef90_A0A3E5AS08_Dorea_formicigenerans	HIV	Pos	-0.758	130	0.254	0.011	0.032
UniRef90_A0A3E5AS10_Coprococcus_comes	HIV	Pos	-0.711	130	0.231	0.015	0.043
UniRef90_A0A3E5AS10_Dorea_formicigenerans	HIV	Pos	-0.648	130	0.246	0.006	0.018
UniRef90_A0A3E5AS10_Dorea_longicatena	HIV	Pos	-0.718	130	0.238	0.007	0.020
UniRef90_A0A3E5AS10_Eubacterium_rectale	HIV	Pos	-0.830	130	0.246	0.008	0.025
UniRef90_A0A3E5AS10_Roseburia_inulinivorans	HIV	Pos	-0.881	130	0.246	0.007	0.021
UniRef90_A0A3E5AS22_Dorea_formicigenerans	HIV	Pos	-0.605	130	0.262	0.035	0.099
UniRef90_A0A3E5AS22_Dorea_longicatena	HIV	Pos	-1.040	130	0.269	0.006	0.019
UniRef90_A0A3E5AS22_Eubacterium_rectale	HIV	Pos	-0.747	130	0.262	0.012	0.037
UniRef90_A0A3E5AS22_Ruminococcus_torques	HIV	Pos	-0.888	130	0.246	0.004	0.011
UniRef90_A0A3E5DLS5_Prevotella_bivia	HIV	Pos	1.168	130	0.315	0.029	0.084
UniRef90_A0A3E5DXX1_Prevotella_sp_AM42_24	HIV	Pos	-0.746	130	0.262	0.041	0.114
UniRef90_A0A3E5DYL0_Prevotella_sp_AM42_24	HIV	Pos	-1.017	130	0.254	0.018	0.053
UniRef90_A0A3E5DZ02_Prevotella_sp_AM42_24	HIV	Pos	-0.548	130	0.246	0.031	0.088
UniRef90_A0A3E5E212_Prevotella_sp_AM42_24	HIV	Pos	-0.754	130	0.231	0.011	0.033
UniRef90_A0A3E5E3Y2_Prevotella_sp_AM42_24	HIV	Pos	-1.188	130	0.238	0.011	0.033
UniRef90_A0A3E5E475_Prevotella_sp_AM42_24	HIV	Pos	-1.036	130	0.238	0.008	0.025
UniRef90_A0A3E5E6M0_Prevotella_sp_AM42_24	HIV	Pos	-0.596	130	0.254	0.035	0.100
UniRef90_A0A3E5E6R6_Prevotella_sp_AM42_24	HIV	Pos	-0.565	130	0.246	0.048	0.132
UniRef90_A0A3E5E8I4_Prevotella_sp_AM42_24	HIV	Pos	-0.522	130	0.254	0.021	0.061
UniRef90_A0A3E5E9L2_Prevotella_sp_AM42_24	HIV	Pos	-0.645	130	0.246	0.043	0.122
UniRef90_A0A3E5EB88_Prevotella_sp_AM42_24	HIV	Pos	-0.812	130	0.254	0.041	0.116
UniRef90_A0A3E5ECA2_Prevotella_sp_AM42_24	HIV	Pos	-0.595	130	0.231	0.008	0.025
UniRef90_A0A3E5EDH2_Prevotella_sp_AM42_24	HIV	Pos	-0.820	130	0.254	0.041	0.116
UniRef90_A0A3F3K0I6_Faecalibacterium_prausnitzii	HIV	Pos	-0.768	130	0.308	0.043	0.119
UniRef90_A0A3F3K661_Faecalibacterium_prausnitzii	HIV	Pos	-0.772	130	0.308	0.020	0.058
UniRef90_A3CR42_Streptococcus_parasanguinis	HIV	Pos	1.365	130	0.254	0.006	0.018
UniRef90_A4W4F3_Streptococcus_parasanguinis	HIV	Pos	1.371	130	0.277	0.021	0.062
UniRef90_A5ZAM4_Dorea_longicatena	HIV	Pos	-1.077	130	0.238	0.009	0.028
UniRef90_A5ZAM4_Eubacterium_rectale	HIV	Pos	-0.891	130	0.231	0.044	0.122
UniRef90_A6BD89_Coprococcus_comes	HIV	Pos	-0.508	130	0.231	0.048	0.133
UniRef90_A6BD89_Dorea_longicatena	HIV	Pos	-0.751	130	0.262	0.032	0.092
UniRef90_A6BEX3_Coprococcus_comes	HIV	Pos	-0.736	130	0.262	0.016	0.048
UniRef90_A6BEX3_Dorea_formicigenerans	HIV	Pos	-1.125	130	0.238	0.013	0.040
UniRef90_A6BEX3_Dorea_longicatena	HIV	Pos	-0.981	130	0.277	0.025	0.073
UniRef90_A6BEX3_Ruminococcus_torques	HIV	Pos	-1.287	130	0.246	0.003	0.008
UniRef90_A6BGA7_Coprococcus_comes	HIV	Pos	-0.624	130	0.238	0.049	0.137
UniRef90_A6BGA7_Dorea_formicigenerans	HIV	Pos	-0.618	130	0.254	0.047	0.131
UniRef90_A6BGA7_Eubacterium_rectale	HIV	Pos	-0.632	130	0.254	0.031	0.088
UniRef90_A6BGA7_Roseburia_inulinivorans	HIV	Pos	-0.721	130	0.238	0.014	0.042
UniRef90_A6BGC7_Coprococcus_comes	HIV	Pos	-0.534	130	0.238	0.049	0.137
UniRef90_A6BGC7_Dorea_formicigenerans	HIV	Pos	-0.681	130	0.254	0.031	0.089
UniRef90_A6BGC7_Dorea_longicatena	HIV	Pos	-0.767	130	0.254	0.016	0.048

UniRef90_A6BGC7_Eubacterium_rectale	HIV	Pos	-0.774	130	0.238	0.011	0.031
UniRef90_A6BKX9_Dorea_longicatena	HIV	Pos	-0.724	130	0.246	0.026	0.075
UniRef90_A6BKY6_Dorea_longicatena	HIV	Pos	-0.870	130	0.292	0.012	0.035
UniRef90_A6NW75_Eubacterium_rectale	HIV	Pos	-0.925	130	0.231	0.037	0.104
UniRef90_A6NW75_Roseburia_inulinivorans	HIV	Pos	-0.718	130	0.246	0.008	0.023
UniRef90_A6NXA0_Roseburia_inulinivorans	HIV	Pos	-0.833	130	0.238	0.007	0.020
UniRef90_A6VL57_Haemophilus_parainfluenzae	HIV	Pos	-1.054	130	0.400	0.044	0.122
UniRef90_A7AZM6_Roseburia_inulinivorans	HIV	Pos	-1.024	130	0.246	0.018	0.052
UniRef90_A7AZN6_Dorea_formicigenerans	HIV	Pos	-0.635	130	0.331	0.033	0.093
UniRef90_A7AZN6_Fusicatenibacter_saccharivorans	HIV	Pos	-0.698	130	0.277	0.025	0.073
UniRef90_A7VCV6_Dorea_formicigenerans	HIV	Pos	-0.756	130	0.238	0.029	0.083
UniRef90_A7VCV6_Eubacterium_rectale	HIV	Pos	-0.710	130	0.238	0.038	0.109
UniRef90_A7VCV6_Faecalibacterium_prausnitzii	HIV	Pos	-0.761	130	0.262	0.041	0.114
UniRef90_A8AUR6_Streptococcus_parasanguinis	HIV	Pos	1.630	130	0.277	0.035	0.098
UniRef90_A8AZI5_Streptococcus_parasanguinis	HIV	Pos	1.466	130	0.254	0.006	0.017
UniRef90_A8AZP0_Streptococcus_parasanguinis	HIV	Pos	1.382	130	0.246	0.012	0.036
UniRef90_A8RRJ5_Coprococcus_comes	HIV	Pos	-1.028	130	0.292	0.011	0.032
UniRef90_A8RRJ5_Roseburia_inulinivorans	HIV	Pos	-0.687	130	0.269	0.033	0.095
UniRef90_A8RRJ5_Ruminococcus_torques	HIV	Pos	-1.178	130	0.269	0.001	0.004
UniRef90_A8S7R8_Faecalibacterium_prausnitzii	HIV	Pos	-0.936	130	0.269	0.017	0.051
UniRef90_A8SC38_Faecalibacterium_prausnitzii	HIV	Pos	-0.909	130	0.254	0.029	0.084
UniRef90_A8SEJ3_Faecalibacterium_prausnitzii	HIV	Pos	-1.355	130	0.246	0.001	0.003
UniRef90_A8SFH4_Faecalibacterium_prausnitzii	HIV	Pos	-0.710	130	0.292	0.020	0.058
UniRef90_A8SHC7_Coprococcus_comes	HIV	Pos	-0.865	130	0.231	0.014	0.041
UniRef90_A8SHC7_Dorea_formicigenerans	HIV	Pos	-0.814	130	0.238	0.015	0.044
UniRef90_A8SHC7_Dorea_longicatena	HIV	Pos	-0.665	130	0.254	0.050	0.137
UniRef90_A8SHC7_Eubacterium_rectale	HIV	Pos	-0.758	130	0.254	0.035	0.099
UniRef90_A8SHC7_Roseburia_inulinivorans	HIV	Pos	-0.713	130	0.254	0.022	0.065
UniRef90_A8SHK3_Faecalibacterium_prausnitzii	HIV	Pos	-0.655	130	0.262	0.029	0.083
UniRef90_A8SV00_Coprococcus_comes	HIV	Pos	-1.038	130	0.285	0.002	0.006
UniRef90_A8SV00_Dorea_longicatena	HIV	Pos	-1.097	130	0.300	0.010	0.028
UniRef90_A8SV00_Fusicatenibacter_saccharivorans	HIV	Pos	-0.712	130	0.277	0.016	0.046
UniRef90_A8SV00_Ruminococcus_torques	HIV	Pos	-0.708	130	0.262	0.017	0.049
UniRef90_A8SVC3_Ruminococcus_torques	HIV	Pos	-0.707	130	0.246	0.032	0.091
UniRef90_B0GAB5_Coprococcus_comes	HIV	Pos	-0.830	130	0.292	0.016	0.047
UniRef90_B0GAB5_Ruminococcus_torques	HIV	Pos	-0.871	130	0.246	0.025	0.073
UniRef90_B0GAE7_Dorea_longicatena	HIV	Pos	-0.900	130	0.231	0.009	0.027
UniRef90_B0NE07_Ruminococcus_torques	HIV	Pos	-1.133	130	0.254	0.017	0.051
UniRef90_B0P5G9_Eubacterium_rectale	HIV	Pos	-1.201	130	0.254	0.011	0.033
UniRef90_B0P879_Faecalibacterium_prausnitzii	HIV	Pos	-1.013	130	0.231	0.023	0.068
UniRef90_B0PDH4_Coprococcus_comes	HIV	Pos	-1.129	130	0.231	0.000	0.000
UniRef90_B1IA36_Streptococcus_parasanguinis	HIV	Pos	2.058	130	0.300	0.008	0.024
UniRef90_B3XSP2_Streptococcus_oralis	HIV	Pos	0.986	130	0.254	0.020	0.059
UniRef90_B5CLP5_Ruminococcus_torques	HIV	Pos	-0.775	130	0.308	0.021	0.060
UniRef90_B5CNA7_Fusicatenibacter_saccharivorans	HIV	Pos	-0.610	130	0.285	0.036	0.103
UniRef90_B5E2L2_Streptococcus_parasanguinis	HIV	Pos	1.031	130	0.238	0.027	0.078
UniRef90_B6FQE1_Fusicatenibacter_saccharivorans	HIV	Pos	-0.840	130	0.231	0.035	0.100
UniRef90_B6FSS6_Coprococcus_comes	HIV	Pos	-0.802	130	0.292	0.008	0.025
UniRef90_B6FSS6_Fusicatenibacter_saccharivorans	HIV	Pos	-0.720	130	0.262	0.021	0.060
UniRef90_B6FSS6_Ruminococcus_torques	HIV	Pos	-0.815	130	0.292	0.018	0.052
UniRef90_B6FU96_Ruminococcus_torques	HIV	Pos	-0.821	130	0.238	0.009	0.026
UniRef90_B7ANM9_Dorea_formicigenerans	HIV	Pos	-0.962	130	0.254	0.005	0.015
UniRef90_B7ANM9_Dorea_longicatena	HIV	Pos	-1.113	130	0.246	0.002	0.007
UniRef90_B7ARW3_Coprococcus_comes	HIV	Pos	-0.664	130	0.300	0.032	0.091
UniRef90_B7CBS9_Holdemanella_biformis	HIV	Pos	-0.862	130	0.231	0.004	0.012
UniRef90_B7CEA2_Holdemanella_biformis	HIV	Pos	-0.862	130	0.231	0.010	0.028

UniRef90_B7CEC9_Holdemanella_biformis	HIV	Pos	-0.858	130	0.231	0.006	0.017
UniRef90_B9DSV1_Streptococcus_parasanguinis	HIV	Pos	0.767	130	0.254	0.033	0.095
UniRef90_B9Y5Q4_Faecalibacterium_prausnitzii	HIV	Pos	-0.812	130	0.238	0.032	0.091
UniRef90_B9Y5S8_Faecalibacterium_prausnitzii	HIV	Pos	-0.743	130	0.246	0.043	0.121
UniRef90_B9Y5T5_Faecalibacterium_prausnitzii	HIV	Pos	-1.019	130	0.231	0.013	0.039
UniRef90_B9Y5T7_Faecalibacterium_prausnitzii	HIV	Pos	-1.094	130	0.231	0.005	0.015
UniRef90_C0B5T7_Fusicatenibacter_saccharivorans	HIV	Pos	-0.527	130	0.262	0.038	0.107
UniRef90_C0B7X0_Faecalibacterium_prausnitzii	HIV	Pos	-0.798	130	0.246	0.038	0.108
UniRef90_C0B7X5_Ruminococcus_torques	HIV	Pos	-0.859	130	0.262	0.033	0.094
UniRef90_C0B9Z9_Coprococcus_comes	HIV	Pos	-0.831	130	0.269	0.006	0.018
UniRef90_C0B9Z9_Fusicatenibacter_saccharivorans	HIV	Pos	-0.747	130	0.269	0.016	0.048
UniRef90_C0B9Z9_Roseburia_inulinivorans	HIV	Pos	-0.815	130	0.254	0.024	0.071
UniRef90_C0B9Z9_Ruminococcus_torques	HIV	Pos	-0.747	130	0.285	0.034	0.097
UniRef90_C0BDI2_Roseburia_inulinivorans	HIV	Pos	-0.669	130	0.292	0.024	0.069
UniRef90_C0BDI2_Ruminococcus_torques	HIV	Pos	-0.785	130	0.238	0.007	0.022
UniRef90_C0CLV9_Coprococcus_comes	HIV	Pos	-0.608	130	0.254	0.038	0.107
UniRef90_C0CLV9_Fusicatenibacter_saccharivorans	HIV	Pos	-0.571	130	0.246	0.031	0.089
UniRef90_C0EVZ0_Fusicatenibacter_saccharivorans	HIV	Pos	-0.694	130	0.277	0.043	0.121
UniRef90_C0EWA4_Coprococcus_comes	HIV	Pos	-1.111	130	0.269	0.002	0.007
UniRef90_C0EWA4_Dorea_longicatena	HIV	Pos	-0.696	130	0.285	0.040	0.114
UniRef90_C0EWA4_Ruminococcus_torques	HIV	Pos	-0.717	130	0.246	0.033	0.095
UniRef90_C0FPL5_Roseburia_inulinivorans	HIV	Pos	-0.873	130	0.238	0.009	0.028
UniRef90_C0FQ15_Ruminococcus_torques	HIV	Pos	-0.790	130	0.269	0.048	0.133
UniRef90_C0FUE1_Coprococcus_comes	HIV	Pos	-0.980	130	0.254	0.006	0.018
UniRef90_C0FUE1_Dorea_longicatena	HIV	Pos	-0.675	130	0.269	0.038	0.107
UniRef90_C0FUE1_Eubacterium_rectale	HIV	Pos	-1.052	130	0.254	0.003	0.008
UniRef90_C0FUE1_Roseburia_inulinivorans	HIV	Pos	-0.611	130	0.254	0.020	0.057
UniRef90_C0FV99_Roseburia_inulinivorans	HIV	Pos	-0.852	130	0.254	0.015	0.045
UniRef90_C0FVR2_Faecalibacterium_prausnitzii	HIV	Pos	-0.598	130	0.269	0.042	0.118
UniRef90_C0FVR2_Roseburia_inulinivorans	HIV	Pos	-0.713	130	0.246	0.046	0.129
UniRef90_C0FW47_Coprococcus_comes	HIV	Pos	-0.833	130	0.246	0.014	0.040
UniRef90_C0FW47_Dorea_longicatena	HIV	Pos	-0.750	130	0.262	0.024	0.070
UniRef90_C0FW47_Faecalibacterium_prausnitzii	HIV	Pos	-1.066	130	0.269	0.036	0.102
UniRef90_C0FW50_Coprococcus_comes	HIV	Pos	-0.885	130	0.269	0.015	0.044
UniRef90_C0FW50_Eubacterium_rectale	HIV	Pos	-0.840	130	0.254	0.014	0.042
UniRef90_C0FW50_Roseburia_inulinivorans	HIV	Pos	-0.988	130	0.269	0.008	0.023
UniRef90_C0FWL4_Fusicatenibacter_saccharivorans	HIV	Pos	-0.816	130	0.254	0.017	0.049
UniRef90_C0FWL4_Ruminococcus_torques	HIV	Pos	-0.833	130	0.254	0.016	0.046
UniRef90_C0FXK6_Faecalibacterium_prausnitzii	HIV	Pos	-0.954	130	0.254	0.038	0.107
UniRef90_C4Z731_Fusicatenibacter_saccharivorans	HIV	Pos	-0.665	130	0.269	0.021	0.062
UniRef90_C4ZEM7_Coprococcus_comes	HIV	Pos	-1.012	130	0.246	0.007	0.020
UniRef90_C4ZEP3_Faecalibacterium_prausnitzii	HIV	Pos	-0.860	130	0.246	0.019	0.056
UniRef90_C4ZES9_Coprococcus_comes	HIV	Pos	-0.751	130	0.246	0.030	0.087
UniRef90_C4ZES9_Dorea_formicigenerans	HIV	Pos	-0.871	130	0.238	0.023	0.068
UniRef90_C4ZES9_Eubacterium_rectale	HIV	Pos	-0.930	130	0.231	0.034	0.098
UniRef90_C4ZES9_Roseburia_inulinivorans	HIV	Pos	-0.896	130	0.254	0.034	0.096
UniRef90_C4ZFE1_Eubacterium_rectale	HIV	Pos	-0.629	130	0.292	0.049	0.136
UniRef90_C4ZFG5_Eubacterium_rectale	HIV	Pos	-0.731	130	0.262	0.045	0.126
UniRef90_C5EWP5_Roseburia_inulinivorans	HIV	Pos	-0.750	130	0.238	0.027	0.077
UniRef90_C6LD83_Eubacterium_rectale	HIV	Pos	-0.726	130	0.231	0.045	0.126
UniRef90_C7H3I1_Roseburia_inulinivorans	HIV	Pos	-1.005	130	0.254	0.004	0.012
UniRef90_C7H604_Faecalibacterium_prausnitzii	HIV	Pos	-0.916	130	0.285	0.011	0.033
UniRef90_C7H653_Faecalibacterium_prausnitzii	HIV	Pos	-0.469	130	0.262	0.031	0.088
UniRef90_C7H697_Faecalibacterium_prausnitzii	HIV	Pos	-0.722	130	0.262	0.050	0.138
UniRef90_C7H6T0_Faecalibacterium_prausnitzii	HIV	Pos	-0.884	130	0.231	0.031	0.089
UniRef90_C7H6T9_Roseburia_inulinivorans	HIV	Pos	-0.879	130	0.254	0.006	0.019

UniRef90_C7H7K4_Coprococcus_comes	HIV	Pos	-0.647	130	0.238	0.021	0.061
UniRef90_C7H7M1_Faecalibacterium_prausnitzii	HIV	Pos	-0.793	130	0.262	0.017	0.050
UniRef90_C7HAG3_Faecalibacterium_prausnitzii	HIV	Pos	-0.694	130	0.300	0.035	0.100
UniRef90_C7HB24_Faecalibacterium_prausnitzii	HIV	Pos	-0.696	130	0.254	0.032	0.092
UniRef90_C9LBR4_Dorea_longicatena	HIV	Pos	-0.803	130	0.231	0.014	0.041
UniRef90_D1P8W7_Prevotella_sp_AM42_24	HIV	Pos	-0.489	130	0.277	0.048	0.135
UniRef90_D1P8Z7_Prevotella_copri	HIV	Pos	-0.426	130	0.285	0.048	0.134
UniRef90_D1P966_Prevotella_sp_AM42_24	HIV	Pos	-0.635	130	0.246	0.028	0.080
UniRef90_D1P979_Prevotella_sp_AM42_24	HIV	Pos	-0.496	130	0.254	0.043	0.122
UniRef90_D1P9M5_Prevotella_sp_AM42_24	HIV	Pos	-0.964	130	0.231	0.011	0.032
UniRef90_D1P9M7_Prevotella_copri	HIV	Pos	-0.610	130	0.231	0.005	0.014
UniRef90_D1PA74_Prevotella_sp_AM42_24	HIV	Pos	-0.725	130	0.262	0.049	0.136
UniRef90_D1PA97_Prevotella_copri	HIV	Pos	-0.550	130	0.254	0.016	0.047
UniRef90_D1PB34_Prevotella_sp_AM42_24	HIV	Pos	-0.527	130	0.238	0.023	0.067
UniRef90_D1PBF0_Prevotella_sp_AM42_24	HIV	Pos	-0.716	130	0.277	0.048	0.134
UniRef90_D1PBR2_Prevotella_copri	HIV	Pos	-0.646	130	0.285	0.018	0.053
UniRef90_D1PC21_Prevotella_sp_AM42_24	HIV	Pos	-0.699	130	0.262	0.045	0.125
UniRef90_D1PCL1_Prevotella_copri	HIV	Pos	-0.589	130	0.292	0.012	0.034
UniRef90_D1PCN7_Prevotella_copri	HIV	Pos	-0.427	130	0.231	0.040	0.113
UniRef90_D1PD84_Prevotella_sp_AM42_24	HIV	Pos	-0.409	130	0.254	0.038	0.106
UniRef90_D1PDT1_Prevotella_copri	HIV	Pos	-0.775	130	0.238	0.039	0.111
UniRef90_D1PE23_Prevotella_sp_AM42_24	HIV	Pos	-1.048	130	0.246	0.012	0.035
UniRef90_D1PEH8_Prevotella_sp_AM42_24	HIV	Pos	-0.600	130	0.246	0.023	0.068
UniRef90_D1PF53_Prevotella_sp_AM42_24	HIV	Pos	-0.500	130	0.262	0.032	0.091
UniRef90_D1PGX1_Prevotella_sp_AM42_24	HIV	Pos	-0.635	130	0.238	0.022	0.064
UniRef90_D1PGX2_Prevotella_sp_AM42_24	HIV	Pos	-0.666	130	0.238	0.020	0.059
UniRef90_D1PH49_Prevotella_copri	HIV	Pos	-0.468	130	0.254	0.045	0.126
UniRef90_D3I8L8_Prevotella_sp_AM42_24	HIV	Pos	-0.452	130	0.254	0.042	0.117
UniRef90_D4C6N2_Roseburia_inulinivorans	HIV	Pos	-0.728	130	0.285	0.045	0.126
UniRef90_D4J5W9_Dorea_longicatena	HIV	Pos	-0.923	130	0.246	0.021	0.060
UniRef90_D4J5W9_Eubacterium_rectale	HIV	Pos	-0.942	130	0.238	0.009	0.028
UniRef90_D4J5X4_Roseburia_inulinivorans	HIV	Pos	-0.549	130	0.238	0.032	0.090
UniRef90_D4J5X8_Coprococcus_comes	HIV	Pos	-0.610	130	0.231	0.025	0.073
UniRef90_D4J5X8_Ruminococcus_torques	HIV	Pos	-0.659	130	0.238	0.015	0.045
UniRef90_D4J5Y5_Ruminococcus_torques	HIV	Pos	-0.803	130	0.262	0.017	0.049
UniRef90_D4J5Z0_Coprococcus_comes	HIV	Pos	-0.612	130	0.246	0.031	0.089
UniRef90_D4J5Z0_Roseburia_inulinivorans	HIV	Pos	-0.645	130	0.262	0.030	0.085
UniRef90_D4JMB0_Eubacterium_rectale	HIV	Pos	-0.819	130	0.231	0.026	0.076
UniRef90_D4JS14_Coprococcus_comes	HIV	Pos	-0.716	130	0.269	0.019	0.054
UniRef90_D4JZP9_Faecalibacterium_prausnitzii	HIV	Pos	-0.665	130	0.231	0.017	0.051
UniRef90_D4K2V6_Faecalibacterium_prausnitzii	HIV	Pos	-0.642	130	0.238	0.041	0.114
UniRef90_D4K413_Faecalibacterium_prausnitzii	HIV	Pos	-0.575	130	0.262	0.044	0.124
UniRef90_D4K4J9_Coprococcus_comes	HIV	Pos	-0.834	130	0.231	0.024	0.070
UniRef90_D4K4K4_Faecalibacterium_prausnitzii	HIV	Pos	-0.682	130	0.254	0.048	0.134
UniRef90_D4K4V0_Faecalibacterium_prausnitzii	HIV	Pos	-0.627	130	0.231	0.023	0.066
UniRef90_D4K5A7_Faecalibacterium_prausnitzii	HIV	Pos	-0.649	130	0.231	0.027	0.077
UniRef90_D4K6T2_Fusicatenibacter_saccharivorans	HIV	Pos	-0.603	130	0.238	0.043	0.121
UniRef90_D4K6T2_Roseburia_inulinivorans	HIV	Pos	-0.887	130	0.246	0.006	0.018
UniRef90_D4K7N2_Faecalibacterium_prausnitzii	HIV	Pos	-0.623	130	0.285	0.034	0.097
UniRef90_D4K8Y0_Faecalibacterium_prausnitzii	HIV	Pos	-0.667	130	0.292	0.041	0.116
UniRef90_D4KAK2_Faecalibacterium_prausnitzii	HIV	Pos	-0.853	130	0.292	0.035	0.100
UniRef90_D4KBC6_Faecalibacterium_prausnitzii	HIV	Pos	-0.679	130	0.254	0.040	0.113
UniRef90_D4KCU0_Faecalibacterium_prausnitzii	HIV	Pos	-0.720	130	0.262	0.027	0.077
UniRef90_D4KV53_Roseburia_inulinivorans	HIV	Pos	-0.809	130	0.254	0.032	0.091
UniRef90_D4KYI8_Roseburia_inulinivorans	HIV	Pos	-1.070	130	0.262	0.017	0.050
UniRef90_D4L1Y4_Roseburia_inulinivorans	HIV	Pos	-1.509	130	0.246	0.002	0.005

UniRef90_D4L236_Roseburia_inulinivorans	HIV	Pos	-1.114	130	0.254	0.011	0.032
UniRef90_D4LY41_Coprococcus_comes	HIV	Pos	-0.890	130	0.300	0.017	0.050
UniRef90_D4ML19_Coprococcus_comes	HIV	Pos	-0.720	130	0.238	0.017	0.050
UniRef90_D4ML19_Dorea_formicigenerans	HIV	Pos	-0.762	130	0.246	0.040	0.113
UniRef90_D4ML19_Dorea_longicatena	HIV	Pos	-1.029	130	0.269	0.036	0.103
UniRef90_D4ML19_Roseburia_inulinivorans	HIV	Pos	-1.123	130	0.262	0.003	0.010
UniRef90_D4MQP8_Faecalibacterium_prausnitzii	HIV	Pos	-0.964	130	0.238	0.022	0.064
UniRef90_D5HH09_Roseburia_inulinivorans	HIV	Pos	-0.678	130	0.246	0.025	0.073
UniRef90_D5HH13_Roseburia_inulinivorans	HIV	Pos	-0.766	130	0.238	0.010	0.029
UniRef90_D6DF46_Faecalibacterium_prausnitzii	HIV	Pos	-0.873	130	0.254	0.045	0.126
UniRef90_D7GT53_Faecalibacterium_prausnitzii	HIV	Pos	-0.806	130	0.246	0.025	0.073
UniRef90_D8E003_Prevotella_copri	HIV	Pos	-0.852	130	0.292	0.041	0.116
UniRef90_D8E003_Prevotella_sp_885	HIV	Pos	-0.804	130	0.231	0.032	0.092
UniRef90_D8E003_Prevotella_sp_AM42_24	HIV	Pos	-1.021	130	0.238	0.009	0.026
UniRef90_D9RTK4_Prevotella_melaninogenica	HIV	Pos	1.069	130	0.238	0.022	0.063
UniRef90_E0Q3M9_Streptococcus_parasanguinis	HIV	Pos	0.858	130	0.238	0.048	0.134
UniRef90_E1LRI1_Streptococcus_mitis	HIV	Pos	-0.978	130	0.277	0.023	0.066
UniRef90_E1W2Q1_Haemophilus_parainfluenzae	HIV	Pos	-0.848	130	0.300	0.043	0.120
UniRef90_E1W2V9_Haemophilus_parainfluenzae	HIV	Pos	-1.185	130	0.362	0.037	0.106
UniRef90_E1W4G0_Haemophilus_parainfluenzae	HIV	Pos	-0.807	130	0.300	0.043	0.119
UniRef90_E2NP50_Catenibacterium_mitsuokai	HIV	Pos	-0.688	130	0.354	0.040	0.112
UniRef90_E2NWJ2_Catenibacterium_mitsuokai	HIV	Pos	-1.089	130	0.262	0.004	0.011
UniRef90_E2NWP6_Catenibacterium_mitsuokai	HIV	Pos	-0.811	130	0.269	0.014	0.042
UniRef90_E2SL03_Faecalibacterium_prausnitzii	HIV	Pos	-0.565	130	0.231	0.050	0.138
UniRef90_E2ZJS2_Faecalibacterium_prausnitzii	HIV	Pos	-0.707	130	0.254	0.023	0.068
UniRef90_E2ZJT1_Faecalibacterium_prausnitzii	HIV	Pos	-0.702	130	0.254	0.020	0.060
UniRef90_E2ZLP5_Faecalibacterium_prausnitzii	HIV	Pos	-0.730	130	0.238	0.030	0.085
UniRef90_E2ZM22_Coprococcus_comes	HIV	Pos	-0.922	130	0.246	0.011	0.032
UniRef90_E2ZM22_Dorea_longicatena	HIV	Pos	-1.010	130	0.269	0.030	0.086
UniRef90_E2ZM22_Eubacterium_rectale	HIV	Pos	-0.737	130	0.254	0.041	0.116
UniRef90_E2ZN31_Faecalibacterium_prausnitzii	HIV	Pos	-0.868	130	0.254	0.027	0.077
UniRef90_E4M058_Roseburia_inulinivorans	HIV	Pos	-0.819	130	0.277	0.044	0.123
UniRef90_E4M058_Ruminococcus_torques	HIV	Pos	-0.837	130	0.269	0.031	0.089
UniRef90_E6J0Q4_Streptococcus_infantis	HIV	Pos	0.718	130	0.269	0.018	0.052
UniRef90_E6KB58_Prevotella_sp_AM42_24	HIV	Pos	-0.601	130	0.246	0.027	0.079
UniRef90_E6KN52_Streptococcus_parasanguinis	HIV	Pos	1.069	130	0.238	0.012	0.036
UniRef90_E6MLK4_Prevotella_sp_AM42_24	HIV	Pos	-0.708	130	0.254	0.020	0.060
UniRef90_E7GMB4_Coprococcus_comes	HIV	Pos	-1.035	130	0.262	0.008	0.025
UniRef90_E7GMB4_Dorea_longicatena	HIV	Pos	-0.862	130	0.308	0.040	0.113
UniRef90_E7GMB4_Eubacterium_rectale	HIV	Pos	-0.801	130	0.285	0.020	0.057
UniRef90_E7GMB4_Faecalibacterium_prausnitzii	HIV	Pos	-0.893	130	0.292	0.017	0.049
UniRef90_E7GMB4_Roseburia_inulinivorans	HIV	Pos	-1.002	130	0.254	0.012	0.036
UniRef90_E7RLS8_Prevotella_sp_AM42_24	HIV	Pos	-0.841	130	0.254	0.034	0.096
UniRef90_E8JVJ0_Streptococcus_oralis	HIV	Pos	0.812	130	0.315	0.048	0.132
UniRef90_E8KA54_Streptococcus_parasanguinis	HIV	Pos	1.069	130	0.262	0.020	0.057
UniRef90_F0ES37_Haemophilus_parainfluenzae	HIV	Pos	-0.786	130	0.292	0.038	0.108
UniRef90_F0EU44_Haemophilus_parainfluenzae	HIV	Pos	-0.776	130	0.315	0.044	0.122
UniRef90_F0H671_Prevotella_sp_AM42_24	HIV	Pos	-0.379	130	0.254	0.030	0.085
UniRef90_F3UMU1_Streptococcus_parasanguinis	HIV	Pos	1.009	130	0.262	0.034	0.097
UniRef90_F7JK95_Roseburia_inulinivorans	HIV	Pos	-0.813	130	0.262	0.017	0.050
UniRef90_F9CZV1_Prevotella_sp_AM42_24	HIV	Pos	-0.897	130	0.254	0.032	0.091
UniRef90_F9D4Y6_Prevotella_sp_AM42_24	HIV	Pos	-0.612	130	0.246	0.026	0.076
UniRef90_F9D663_Prevotella_sp_AM42_24	HIV	Pos	-0.640	130	0.246	0.019	0.055
UniRef90_G1WD84_Prevotella_melaninogenica	HIV	Pos	1.157	130	0.246	0.039	0.111
UniRef90_G1WUV0_Dorea_formicigenerans	HIV	Pos	-1.070	130	0.231	0.004	0.013
UniRef90_G2T0F8_Coprococcus_comes	HIV	Pos	-0.843	130	0.315	0.012	0.035

UniRef90_G2T3B6_Faecalibacterium_prausnitzii	HIV	Pos	-0.923	130	0.238	0.028	0.081
UniRef90_G2T5K3_Coprococcus_comes	HIV	Pos	-0.804	130	0.315	0.005	0.015
UniRef90_G2T5K3_Ruminococcus_torques	HIV	Pos	-0.705	130	0.308	0.025	0.072
UniRef90_G4KVU7_Oscillibacter_sp_CAG_241	HIV	Pos	-0.818	130	0.246	0.021	0.062
UniRef90_G4KVX5_Oscillibacter_sp_CAG_241	HIV	Pos	-0.855	130	0.246	0.014	0.041
UniRef90_G6AY33_Prevotella_sp_AM42_24	HIV	Pos	-0.643	130	0.262	0.047	0.131
UniRef90_G6AYE0_Prevotella_sp_CAG_279	HIV	Pos	-0.350	130	0.238	0.040	0.114
UniRef90_G6C7N6_Streptococcus_oralis	HIV	Pos	0.592	130	0.269	0.018	0.053
UniRef90_G8FXM5_Roseburia_inulinivorans	HIV	Pos	-0.588	130	0.285	0.026	0.075
UniRef90_G9RR38_Faecalibacterium_prausnitzii	HIV	Pos	-0.694	130	0.231	0.025	0.073
UniRef90_G9RSQ0_Coprococcus_comes	HIV	Pos	-0.995	130	0.300	0.009	0.028
UniRef90_G9RSQ0_Ruminococcus_torques	HIV	Pos	-0.790	130	0.292	0.046	0.127
UniRef90_G9YLS6_Roseburia_inulinivorans	HIV	Pos	-0.946	130	0.254	0.007	0.021
UniRef90_I1ZJ37_Streptococcus_parasanguinis	HIV	Pos	1.349	130	0.277	0.013	0.039
UniRef90_J5UNM3_Roseburia_inulinivorans	HIV	Pos	-0.733	130	0.246	0.036	0.101
UniRef90_J7T504_Streptococcus_parasanguinis	HIV	Pos	1.000	130	0.238	0.023	0.068
UniRef90_L1PB72_Prevotella_melaninogenica	HIV	Pos	0.716	130	0.262	0.028	0.081
UniRef90_L7VSY8_Streptococcus_oralis	HIV	Pos	1.332	130	0.269	0.010	0.031
UniRef90_O06942_Streptococcus_parasanguinis	HIV	Pos	1.446	130	0.269	0.015	0.045
UniRef90_O52836_Roseburia_inulinivorans	HIV	Pos	-0.564	130	0.300	0.042	0.118
UniRef90_P0DA82_Streptococcus_parasanguinis	HIV	Pos	1.375	130	0.277	0.011	0.032
UniRef90_P10324_Haemophilus_haemolyticus	HIV	Pos	-0.821	130	0.285	0.013	0.038
UniRef90_P10952_Coprococcus_comes	HIV	Pos	-0.875	130	0.315	0.044	0.124
UniRef90_P22886_Streptococcus_oralis	HIV	Pos	0.941	130	0.238	0.040	0.114
UniRef90_P29849_Streptococcus_parasanguinis	HIV	Pos	0.912	130	0.254	0.045	0.126
UniRef90_P33950_Haemophilus_parainfluenzae	HIV	Pos	-1.605	130	0.354	0.042	0.118
UniRef90_P37247_Prevotella_buccalis	HIV	Pos	-0.841	130	0.231	0.038	0.108
UniRef90_P43812_Haemophilus_haemolyticus	HIV	Pos	-0.974	130	0.262	0.040	0.114
UniRef90_P43836_Haemophilus_parainfluenzae	HIV	Pos	-0.870	130	0.331	0.035	0.101
UniRef90_P43861_Haemophilus_haemolyticus	HIV	Pos	-0.660	130	0.300	0.048	0.133
UniRef90_P43955_Haemophilus_parainfluenzae	HIV	Pos	-1.556	130	0.354	0.042	0.117
UniRef90_P44630_Haemophilus_haemolyticus	HIV	Pos	-0.890	130	0.300	0.026	0.074
UniRef90_P44744_Haemophilus_parainfluenzae	HIV	Pos	-0.797	130	0.338	0.043	0.119
UniRef90_P44786_Haemophilus_parainfluenzae	HIV	Pos	-1.664	130	0.369	0.021	0.060
UniRef90_P44808_Haemophilus_haemolyticus	HIV	Pos	-1.050	130	0.315	0.033	0.093
UniRef90_P44862_Haemophilus_haemolyticus	HIV	Pos	-0.802	130	0.246	0.037	0.106
UniRef90_P44894_Haemophilus_haemolyticus	HIV	Pos	-0.761	130	0.338	0.038	0.108
UniRef90_P45049_Haemophilus_parainfluenzae	HIV	Pos	-1.881	130	0.354	0.047	0.130
UniRef90_P45180_Haemophilus_haemolyticus	HIV	Pos	-0.775	130	0.231	0.026	0.074
UniRef90_P45308_Haemophilus_parainfluenzae	HIV	Pos	-0.995	130	0.285	0.041	0.116
UniRef90_P66648_Streptococcus_parasanguinis	HIV	Pos	1.347	130	0.254	0.044	0.124
UniRef90_P71355_Haemophilus_parainfluenzae	HIV	Pos	-0.960	130	0.338	0.030	0.086
UniRef90_P72478_Streptococcus_parasanguinis	HIV	Pos	1.825	130	0.315	0.021	0.062
UniRef90_P95765_Streptococcus_parasanguinis	HIV	Pos	1.348	130	0.277	0.027	0.078
UniRef90_Q03L20_Streptococcus_parasanguinis	HIV	Pos	1.256	130	0.254	0.020	0.059
UniRef90_Q04IA2_Streptococcus_parasanguinis	HIV	Pos	0.939	130	0.238	0.010	0.031
UniRef90_Q04IK6_Streptococcus_parasanguinis	HIV	Pos	1.407	130	0.300	0.013	0.040
UniRef90_Q04IK7_Streptococcus_parasanguinis	HIV	Pos	0.991	130	0.246	0.022	0.064
UniRef90_Q04KG2_Streptococcus_parasanguinis	HIV	Pos	1.151	130	0.262	0.044	0.122
UniRef90_Q04LT4_Streptococcus_parasanguinis	HIV	Pos	1.010	130	0.262	0.011	0.033
UniRef90_Q04LT6_Streptococcus_parasanguinis	HIV	Pos	0.913	130	0.269	0.044	0.123
UniRef90_Q04MV1_Streptococcus_parasanguinis	HIV	Pos	1.230	130	0.254	0.014	0.042
UniRef90_Q3K2Y7_Streptococcus_parasanguinis	HIV	Pos	1.911	130	0.269	0.004	0.011
UniRef90_Q47733_Streptococcus_oralis	HIV	Pos	1.296	130	0.277	0.014	0.042
UniRef90_Q4QLH6_Haemophilus_parainfluenzae	HIV	Pos	-0.759	130	0.246	0.028	0.080
UniRef90_Q4QLZ6_Haemophilus_parainfluenzae	HIV	Pos	-1.065	130	0.377	0.033	0.095

UniRef90_Q59623_Haemophilus_haemolyticus	HIV	Pos	-0.718	130	0.308	0.040	0.112
UniRef90_Q5LXZ6_Streptococcus_parasanguinis	HIV	Pos	1.447	130	0.246	0.022	0.063
UniRef90_Q8DRA8_Streptococcus_parasanguinis	HIV	Pos	1.120	130	0.238	0.031	0.088
UniRef90_Q97SE7_Streptococcus_parasanguinis	HIV	Pos	1.419	130	0.246	0.002	0.005
UniRef90_Q97TA2_Streptococcus_parasanguinis	HIV	Pos	2.177	130	0.262	0.002	0.006
UniRef90_Q9CK94_Haemophilus_haemolyticus	HIV	Pos	-0.971	130	0.231	0.041	0.115
UniRef90_Q9F0R4_Streptococcus_parasanguinis	HIV	Pos	1.144	130	0.246	0.024	0.070
UniRef90_R0CK04_Dorea_longicatena	HIV	Pos	-0.965	130	0.238	0.035	0.100
UniRef90_R0CK04_Roseburia_inulinivorans	HIV	Pos	-0.758	130	0.269	0.029	0.084
UniRef90_R0CK04_Ruminococcus_torques	HIV	Pos	-0.677	130	0.238	0.037	0.105
UniRef90_R0P2S7_Streptococcus_parasanguinis	HIV	Pos	1.260	130	0.254	0.011	0.033
UniRef90_R5CYJ5_Oscillibacter_sp_CAG_241	HIV	Pos	-0.655	130	0.238	0.050	0.138
UniRef90_R5D0Y4_Oscillibacter_sp_CAG_241	HIV	Pos	-0.924	130	0.231	0.036	0.102
UniRef90_R5D154_Oscillibacter_sp_CAG_241	HIV	Pos	-0.841	130	0.231	0.040	0.112
UniRef90_R5D372_Oscillibacter_sp_CAG_241	HIV	Pos	-0.673	130	0.231	0.027	0.079
UniRef90_R5SME4_Oscillibacter_sp_CAG_241	HIV	Pos	-0.901	130	0.238	0.020	0.059
UniRef90_R6AN06_Prevotella_sp_AM42_24	HIV	Pos	-0.620	130	0.254	0.034	0.097
UniRef90_R6AQ06_Prevotella_sp_AM42_24	HIV	Pos	-0.579	130	0.254	0.031	0.089
UniRef90_R6ARD8_Prevotella_sp_AM42_24	HIV	Pos	-0.595	130	0.246	0.029	0.084
UniRef90_R6ARE3_Prevotella_sp_AM42_24	HIV	Pos	-0.662	130	0.231	0.035	0.100
UniRef90_R6AT20_Prevotella_sp_AM42_24	HIV	Pos	-0.476	130	0.262	0.047	0.130
UniRef90_R6ATL0_Prevotella_sp_AM42_24	HIV	Pos	-0.784	130	0.254	0.034	0.097
UniRef90_R6AUU5_Prevotella_sp_AM42_24	HIV	Pos	-0.889	130	0.246	0.013	0.039
UniRef90_R6AXL4_Prevotella_sp_AM42_24	HIV	Pos	-0.616	130	0.254	0.046	0.129
UniRef90_R6B083_Prevotella_sp_AM42_24	HIV	Pos	-0.634	130	0.254	0.027	0.077
UniRef90_R6B3C0_Prevotella_sp_AM42_24	HIV	Pos	-0.418	130	0.254	0.046	0.128
UniRef90_R6B3G2_Prevotella_sp_AM42_24	HIV	Pos	-0.832	130	0.238	0.017	0.050
UniRef90_R6B4W3_Prevotella_sp_AM42_24	HIV	Pos	-0.774	130	0.238	0.017	0.050
UniRef90_R6B7G3_Prevotella_sp_AM42_24	HIV	Pos	-0.697	130	0.262	0.040	0.113
UniRef90_R6B9C0_Prevotella_sp_AM42_24	HIV	Pos	-0.618	130	0.262	0.031	0.087
UniRef90_R6BB76_Prevotella_sp_AM42_24	HIV	Pos	-0.626	130	0.246	0.037	0.106
UniRef90_R6BB81_Prevotella_sp_AM42_24	HIV	Pos	-0.548	130	0.246	0.037	0.105
UniRef90_R6BKA9_Prevotella_sp_AM42_24	HIV	Pos	-0.595	130	0.262	0.041	0.115
UniRef90_R6BN06_Prevotella_sp_AM42_24	HIV	Pos	-0.516	130	0.262	0.048	0.133
UniRef90_R6C291_Prevotella_sp_AM42_24	HIV	Pos	-0.645	130	0.254	0.037	0.106
UniRef90_R6CHM8_Prevotella_sp_AM42_24	HIV	Pos	-0.496	130	0.238	0.016	0.047
UniRef90_R6CZN9_Oscillibacter_sp_CAG_241	HIV	Pos	-0.704	130	0.238	0.035	0.099
UniRef90_R6DD67_Oscillibacter_sp_CAG_241	HIV	Pos	-0.677	130	0.231	0.043	0.121
UniRef90_R6E9H0_Prevotella_sp_AM42_24	HIV	Pos	-0.598	130	0.246	0.013	0.037
UniRef90_R6EH73_Prevotella_copri	HIV	Pos	-0.625	130	0.262	0.021	0.062
UniRef90_R6EPT0_Dorea_formicigenerans	HIV	Pos	-0.925	130	0.246	0.049	0.137
UniRef90_R6EPT0_Fusicatenibacter_saccharivorans	HIV	Pos	-1.079	130	0.246	0.005	0.016
UniRef90_R6GPU4_Oscillibacter_sp_CAG_241	HIV	Pos	-0.693	130	0.231	0.036	0.102
UniRef90_R6GZD8_Oscillibacter_sp_CAG_241	HIV	Pos	-0.715	130	0.231	0.036	0.102
UniRef90_R6GZK0_Oscillibacter_sp_CAG_241	HIV	Pos	-0.712	130	0.231	0.040	0.112
UniRef90_R6HGF7_Oscillibacter_sp_CAG_241	HIV	Pos	-0.718	130	0.231	0.027	0.077
UniRef90_R6HMP3_Oscillibacter_sp_CAG_241	HIV	Pos	-1.199	130	0.231	0.015	0.043
UniRef90_R6PLX8_Prevotella_sp_AM42_24	HIV	Pos	-0.885	130	0.254	0.027	0.078
UniRef90_R6PMU4_Prevotella_sp_AM42_24	HIV	Pos	-0.715	130	0.254	0.025	0.072
UniRef90_R6PNS9_Prevotella_sp_AM42_24	HIV	Pos	-0.710	130	0.246	0.015	0.044
UniRef90_R6PPT7_Prevotella_sp_AM42_24	HIV	Pos	-0.702	130	0.269	0.040	0.114
UniRef90_R6PRR8_Prevotella_sp_AM42_24	HIV	Pos	-0.530	130	0.246	0.022	0.063
UniRef90_R6PX24_Prevotella_sp_AM42_24	HIV	Pos	-0.717	130	0.262	0.045	0.127
UniRef90_R6Q8L0_Faecalibacterium_prausnitzii	HIV	Pos	-0.701	130	0.277	0.026	0.075
UniRef90_R6QDH9_Prevotella_sp_AM42_24	HIV	Pos	-0.628	130	0.254	0.022	0.065
UniRef90_R6QE29_Prevotella_sp_AM42_24	HIV	Pos	-1.011	130	0.246	0.018	0.054

UniRef90_R6QTW6_Catenibacterium_mitsuokai	HIV	Pos	-1.044	130	0.238	0.027	0.079
UniRef90_R6VTN4_Prevotella_sp_AM42_24	HIV	Pos	-0.476	130	0.238	0.037	0.104
UniRef90_R6X078_Prevotella_sp_AM42_24	HIV	Pos	-0.868	130	0.262	0.031	0.090
UniRef90_R6X3T9_Prevotella_copri	HIV	Pos	-0.530	130	0.254	0.042	0.117
UniRef90_R6X420_Prevotella_sp_AM42_24	HIV	Pos	-0.647	130	0.254	0.039	0.111
UniRef90_R6X4X0_Prevotella_sp_AM42_24	HIV	Pos	-0.644	130	0.238	0.012	0.036
UniRef90_R6X5M0_Prevotella_sp_AM42_24	HIV	Pos	-0.729	130	0.246	0.042	0.118
UniRef90_R6X5Z9_Prevotella_sp_AM42_24	HIV	Pos	-0.543	130	0.254	0.027	0.079
UniRef90_R6X6A2_Prevotella_sp_AM42_24	HIV	Pos	-0.614	130	0.238	0.034	0.096
UniRef90_R6X8Q0_Prevotella_sp_AM42_24	HIV	Pos	-0.679	130	0.277	0.046	0.129
UniRef90_R6X8Z2_Prevotella_sp_AM42_24	HIV	Pos	-0.483	130	0.277	0.047	0.130
UniRef90_R6XA66_Prevotella_sp_AM42_24	HIV	Pos	-0.802	130	0.246	0.045	0.127
UniRef90_R6XAC5_Prevotella_sp_AM42_24	HIV	Pos	-0.534	130	0.238	0.033	0.094
UniRef90_R6XBE1_Prevotella_sp_AM42_24	HIV	Pos	-0.625	130	0.254	0.039	0.111
UniRef90_R6XCD8_Prevotella_sp_AM42_24	HIV	Pos	-0.829	130	0.254	0.027	0.079
UniRef90_R6XCU6_Prevotella_sp_AM42_24	HIV	Pos	-0.563	130	0.238	0.026	0.076
UniRef90_R6XD85_Prevotella_sp_AM42_24	HIV	Pos	-0.830	130	0.262	0.022	0.064
UniRef90_R6XEM9_Prevotella_sp_AM42_24	HIV	Pos	-0.462	130	0.246	0.024	0.070
UniRef90_R6XF64_Prevotella_sp_AM42_24	HIV	Pos	-0.979	130	0.262	0.048	0.134
UniRef90_R6XFB4_Prevotella_sp_AM42_24	HIV	Pos	-0.583	130	0.262	0.046	0.127
UniRef90_R6XMA3_Prevotella_sp_AM42_24	HIV	Pos	-0.381	130	0.254	0.043	0.120
UniRef90_R6XN93_Prevotella_sp_AM42_24	HIV	Pos	-0.832	130	0.246	0.029	0.083
UniRef90_R6XNA1_Prevotella_sp_AM42_24	HIV	Pos	-0.637	130	0.246	0.031	0.088
UniRef90_R6XNW2_Prevotella_sp_AM42_24	HIV	Pos	-0.661	130	0.262	0.033	0.095
UniRef90_R6XPA8_Prevotella_sp_AM42_24	HIV	Pos	-0.571	130	0.246	0.049	0.135
UniRef90_R6XPP6_Prevotella_sp_AM42_24	HIV	Pos	-0.937	130	0.246	0.016	0.048
UniRef90_R6XRJ6_Prevotella_sp_AM42_24	HIV	Pos	-0.664	130	0.238	0.019	0.055
UniRef90_R6XSY0_Prevotella_sp_AM42_24	HIV	Pos	-0.510	130	0.262	0.050	0.138
UniRef90_R6XTG2_Prevotella_sp_AM42_24	HIV	Pos	-0.645	130	0.262	0.047	0.130
UniRef90_R6XU16_Prevotella_sp_AM42_24	HIV	Pos	-0.624	130	0.262	0.046	0.129
UniRef90_R6XUE1_Prevotella_sp_AM42_24	HIV	Pos	-0.644	130	0.262	0.040	0.113
UniRef90_R6XVE5_Prevotella_sp_AM42_24	HIV	Pos	-0.568	130	0.254	0.032	0.091
UniRef90_R6XVK0_Prevotella_sp_AM42_24	HIV	Pos	-0.828	130	0.262	0.042	0.119
UniRef90_R6XXB0_Prevotella_sp_AM42_24	HIV	Pos	-0.595	130	0.269	0.048	0.135
UniRef90_R6YEY2_Prevotella_sp_AM42_24	HIV	Pos	-0.520	130	0.246	0.044	0.124
UniRef90_R6YF93_Prevotella_sp_AM42_24	HIV	Pos	-0.408	130	0.246	0.036	0.102
UniRef90_R6YIG0_Prevotella_sp_AM42_24	HIV	Pos	-0.760	130	0.238	0.016	0.047
UniRef90_R6YLZ0_Prevotella_sp_AM42_24	HIV	Pos	-0.764	130	0.254	0.020	0.059
UniRef90_R6YN86_Prevotella_sp_AM42_24	HIV	Pos	-0.747	130	0.246	0.022	0.064
UniRef90_R6YPZ8_Prevotella_sp_AM42_24	HIV	Pos	-0.704	130	0.269	0.046	0.128
UniRef90_R6YQM3_Prevotella_sp_AM42_24	HIV	Pos	-0.636	130	0.254	0.019	0.056
UniRef90_R6YQY3_Prevotella_sp_AM42_24	HIV	Pos	-0.402	130	0.262	0.041	0.114
UniRef90_R6YRU8_Prevotella_sp_AM42_24	HIV	Pos	-0.512	130	0.262	0.047	0.132
UniRef90_R9HXJ2_Catenibacterium_mitsuokai	HIV	Pos	-0.952	130	0.300	0.032	0.091
UniRef90_R9LK49_Faecalibacterium_prausnitzii	HIV	Pos	-0.975	130	0.238	0.006	0.018
UniRef90_S0FD81_Bacteroides_vulgatus	HIV	Pos	-0.450	130	0.254	0.041	0.114
UniRef90_S0FDJ3_Prevotella_sp_AM42_24	HIV	Pos	-0.549	130	0.238	0.012	0.035
UniRef90_U2BAG9_Roseburia_inulinivorans	HIV	Pos	-1.015	130	0.246	0.011	0.031
UniRef90_U2DKD6_Coprococcus_comes	HIV	Pos	-0.845	130	0.254	0.013	0.039
UniRef90_U2J131_Prevotella_sp_AM42_24	HIV	Pos	-0.465	130	0.254	0.049	0.136
UniRef90_U2KQU1_Dorea_formicigenerans	HIV	Pos	-0.712	130	0.269	0.050	0.137
UniRef90_U2KQU1_Dorea_longicatena	HIV	Pos	-0.665	130	0.246	0.035	0.099
UniRef90_U5F4Y0_Ruminococcus_torques	HIV	Pos	-0.710	130	0.262	0.049	0.136
UniRef90_UPI000DE266AC_Faecalibacterium_prausnitzii	HIV	Pos	-1.096	130	0.238	0.005	0.015
UniRef90_V8BHM6_Streptococcus_parasanguinis	HIV	Pos	1.545	130	0.254	0.007	0.020
UniRef90_A0A3E4WLB1_Prevotella_bivia	lesions	yes	1.761	130	0.331	0.002	0.007

UniRef90_H1DBY5_Fusobacterium_gonidiaformans	lesions	yes	1.847	130	0.271	0.003	0.008
UniRef90_D4IQJ2_Prevotella_bivia	lesions	yes	1.340	130	0.346	0.003	0.009
UniRef90_A0A162IIZ1_Fusobacterium_gonidiaformans	lesions	yes	1.665	130	0.263	0.003	0.010
UniRef90_A0A3E2TDA3_Faecalibacterium_prausnitzii	lesions	yes	1.418	130	0.241	0.004	0.011
UniRef90_A0A396E3S1_Prevotella_bivia	lesions	yes	1.478	130	0.308	0.004	0.012
UniRef90_H1D624_Fusobacterium_gonidiaformans	lesions	yes	1.573	130	0.263	0.004	0.012
UniRef90_H1DA99_Fusobacterium_gonidiaformans	lesions	yes	2.146	130	0.248	0.004	0.012
UniRef90_H1DC92_Fusobacterium_gonidiaformans	lesions	yes	1.686	130	0.263	0.004	0.012
UniRef90_E5BH56_Fusobacterium_gonidiaformans	lesions	yes	1.779	130	0.263	0.004	0.012
UniRef90_E5BEG0_Fusobacterium_gonidiaformans	lesions	yes	1.891	130	0.271	0.004	0.012
UniRef90_J6GQZ0_Fusobacterium_gonidiaformans	lesions	yes	1.689	130	0.248	0.004	0.013
UniRef90_E5BIB0_Fusobacterium_gonidiaformans	lesions	yes	1.432	130	0.241	0.004	0.013
UniRef90_H1DC86_Fusobacterium_gonidiaformans	lesions	yes	1.679	130	0.256	0.004	0.013
UniRef90_A0A133NCY1_Fusobacterium_gonidiaformans	lesions	yes	1.442	130	0.263	0.004	0.013
UniRef90_UPI000D13A76A_Fusobacterium_gonidiaformans	lesions	yes	1.488	130	0.286	0.004	0.013
UniRef90_A0A133N6Q2_Fusobacterium_gonidiaformans	lesions	yes	1.640	130	0.256	0.004	0.013
UniRef90_D4CVQ3_Fusobacterium_gonidiaformans	lesions	yes	1.747	130	0.301	0.005	0.014
UniRef90_H1D670_Fusobacterium_gonidiaformans	lesions	yes	1.915	130	0.263	0.005	0.014
UniRef90_H1DBV4_Fusobacterium_gonidiaformans	lesions	yes	1.691	130	0.263	0.005	0.014
UniRef90_E5BG54_Fusobacterium_gonidiaformans	lesions	yes	1.884	130	0.263	0.005	0.014
UniRef90_E5BHQ1_Fusobacterium_gonidiaformans	lesions	yes	1.407	130	0.241	0.005	0.014
UniRef90_A0A170MWV6_Fusobacterium_gonidiaformans	lesions	yes	1.625	130	0.263	0.005	0.014
UniRef90_A0A162II68_Fusobacterium_gonidiaformans	lesions	yes	1.611	130	0.263	0.005	0.015
UniRef90_H1D4C7_Fusobacterium_gonidiaformans	lesions	yes	1.622	130	0.263	0.005	0.015
UniRef90_H1D5A3_Fusobacterium_gonidiaformans	lesions	yes	1.647	130	0.263	0.005	0.015
UniRef90_E5BF78_Fusobacterium_gonidiaformans	lesions	yes	1.544	130	0.256	0.005	0.015
UniRef90_H1D597_Fusobacterium_gonidiaformans	lesions	yes	1.568	130	0.263	0.005	0.015
UniRef90_H1DC80_Fusobacterium_gonidiaformans	lesions	yes	1.492	130	0.263	0.005	0.015
UniRef90_A0A133NEM1_Fusobacterium_gonidiaformans	lesions	yes	1.576	130	0.263	0.005	0.015
UniRef90_A0A133NCV6_Fusobacterium_gonidiaformans	lesions	yes	1.553	130	0.263	0.005	0.015
UniRef90_A0A170MVI6_Fusobacterium_gonidiaformans	lesions	yes	1.695	130	0.263	0.005	0.015
UniRef90_A0A017H379_Fusobacterium_gonidiaformans	lesions	yes	1.795	130	0.241	0.005	0.016
UniRef90_A0A161QTS1_Fusobacterium_gonidiaformans	lesions	yes	1.712	130	0.263	0.005	0.016
UniRef90_E5BHU5_Fusobacterium_gonidiaformans	lesions	yes	1.630	130	0.233	0.005	0.016
UniRef90_E5BHH3_Fusobacterium_gonidiaformans	lesions	yes	1.515	130	0.263	0.005	0.016
UniRef90_A0A133NFF1_Fusobacterium_gonidiaformans	lesions	yes	1.640	130	0.263	0.005	0.016
UniRef90_H1DBY3_Fusobacterium_gonidiaformans	lesions	yes	1.528	130	0.263	0.005	0.016
UniRef90_E5BDT0_Fusobacterium_gonidiaformans	lesions	yes	1.631	130	0.256	0.005	0.016
UniRef90_E5BHM9_Fusobacterium_gonidiaformans	lesions	yes	1.595	130	0.263	0.005	0.016
UniRef90_A0A133NHY7_Fusobacterium_gonidiaformans	lesions	yes	1.681	130	0.263	0.005	0.016
UniRef90_H1DC43_Fusobacterium_gonidiaformans	lesions	yes	1.637	130	0.263	0.005	0.016
UniRef90_J6GPI0_Fusobacterium_gonidiaformans	lesions	yes	1.493	130	0.263	0.006	0.017
UniRef90_E5BDI5_Fusobacterium_gonidiaformans	lesions	yes	1.372	130	0.241	0.006	0.017
UniRef90_E5BHR0_Fusobacterium_gonidiaformans	lesions	yes	1.533	130	0.263	0.006	0.017
UniRef90_H1DBV0_Fusobacterium_gonidiaformans	lesions	yes	1.473	130	0.263	0.006	0.017
UniRef90_A0A017H4U5_Fusobacterium_gonidiaformans	lesions	yes	1.478	130	0.248	0.006	0.018
UniRef90_E5BIC7_Fusobacterium_gonidiaformans	lesions	yes	1.808	130	0.263	0.006	0.018
UniRef90_E5BFX7_Fusobacterium_gonidiaformans	lesions	yes	1.541	130	0.241	0.006	0.018
UniRef90_E5BHH2_Fusobacterium_gonidiaformans	lesions	yes	1.507	130	0.263	0.006	0.018
UniRef90_A0A2P1RHT4_Fusobacterium_gonidiaformans	lesions	yes	1.202	130	0.263	0.006	0.018
UniRef90_E5BHM4_Fusobacterium_gonidiaformans	lesions	yes	1.396	130	0.256	0.006	0.018
UniRef90_A0A3E4TAH0_Prevotella_bivia	lesions	yes	1.439	130	0.383	0.006	0.018
UniRef90_H1DBM5_Fusobacterium_gonidiaformans	lesions	yes	1.519	130	0.256	0.006	0.018
UniRef90_H1DC73_Fusobacterium_gonidiaformans	lesions	yes	1.474	130	0.256	0.006	0.018
UniRef90_E5BI88_Fusobacterium_gonidiaformans	lesions	yes	1.908	130	0.248	0.006	0.018
UniRef90_J5TR32_Fusobacterium_gonidiaformans	lesions	yes	1.569	130	0.263	0.006	0.018

UniRef90_E5BIE0_Fusobacterium_gonidiaformans	lesions	yes	1.598	130	0.263	0.006	0.018
UniRef90_H1DBC9_Fusobacterium_gonidiaformans	lesions	yes	1.417	130	0.263	0.006	0.018
UniRef90_A0A133NF18_Fusobacterium_gonidiaformans	lesions	yes	1.564	130	0.263	0.006	0.018
UniRef90_E5BHV4_Fusobacterium_gonidiaformans	lesions	yes	1.888	130	0.256	0.006	0.018
UniRef90_H1DB58_Fusobacterium_gonidiaformans	lesions	yes	1.857	130	0.263	0.006	0.018
UniRef90_H1D518_Fusobacterium_gonidiaformans	lesions	yes	1.510	130	0.256	0.006	0.019
UniRef90_A0A133NBK4_Fusobacterium_gonidiaformans	lesions	yes	1.481	130	0.263	0.006	0.019
UniRef90_H1PY35_Fusobacterium_gonidiaformans	lesions	yes	1.538	130	0.256	0.006	0.019
UniRef90_E5BHE3_Fusobacterium_gonidiaformans	lesions	yes	1.528	130	0.248	0.006	0.019
UniRef90_H1D909_Fusobacterium_gonidiaformans	lesions	yes	1.592	130	0.256	0.006	0.019
UniRef90_E5BGP8_Fusobacterium_gonidiaformans	lesions	yes	1.828	130	0.248	0.006	0.019
UniRef90_E5BG80_Fusobacterium_gonidiaformans	lesions	yes	1.666	130	0.241	0.006	0.019
UniRef90_E5BDY5_Fusobacterium_gonidiaformans	lesions	yes	1.472	130	0.233	0.006	0.019
UniRef90_H1DAS6_Fusobacterium_gonidiaformans	lesions	yes	1.567	130	0.256	0.007	0.020
UniRef90_A0A133N740_Fusobacterium_gonidiaformans	lesions	yes	1.857	130	0.256	0.007	0.020
UniRef90_A0A133NJW0_Fusobacterium_gonidiaformans	lesions	yes	1.543	130	0.241	0.007	0.020
UniRef90_A0A133N730_Fusobacterium_gonidiaformans	lesions	yes	1.457	130	0.263	0.007	0.020
UniRef90_E5BHN1_Fusobacterium_gonidiaformans	lesions	yes	1.564	130	0.256	0.007	0.020
UniRef90_H1D415_Fusobacterium_gonidiaformans	lesions	yes	1.759	130	0.256	0.007	0.020
UniRef90_A0A2I1YY65_Streptococcus_oralis	lesions	yes	1.555	130	0.398	0.007	0.020
UniRef90_E5BIM3_Fusobacterium_gonidiaformans	lesions	yes	1.644	130	0.248	0.007	0.020
UniRef90_A0A133PU47_Prevotella_bivia	lesions	yes	1.825	130	0.376	0.007	0.020
UniRef90_H1D9A8_Fusobacterium_gonidiaformans	lesions	yes	1.692	130	0.256	0.007	0.020
UniRef90_E5BGA2_Fusobacterium_gonidiaformans	lesions	yes	1.569	130	0.256	0.007	0.021
UniRef90_A0A017H4K1_Fusobacterium_gonidiaformans	lesions	yes	1.507	130	0.256	0.007	0.021
UniRef90_E5BIE5_Fusobacterium_gonidiaformans	lesions	yes	1.677	130	0.256	0.007	0.021
UniRef90_J5TX02_Fusobacterium_gonidiaformans	lesions	yes	1.220	130	0.256	0.007	0.021
UniRef90_A0A133NKW7_Fusobacterium_gonidiaformans	lesions	yes	1.703	130	0.256	0.007	0.021
UniRef90_A0A162IKM2_Fusobacterium_gonidiaformans	lesions	yes	1.413	130	0.263	0.007	0.021
UniRef90_E5BE63_Fusobacterium_gonidiaformans	lesions	yes	1.692	130	0.248	0.007	0.021
UniRef90_A0A133NFJ5_Fusobacterium_gonidiaformans	lesions	yes	1.555	130	0.256	0.007	0.021
UniRef90_H1D510_Fusobacterium_gonidiaformans	lesions	yes	1.369	130	0.256	0.007	0.021
UniRef90_E5BIG1_Fusobacterium_gonidiaformans	lesions	yes	1.873	130	0.256	0.007	0.021
UniRef90_E5BDS6_Fusobacterium_gonidiaformans	lesions	yes	1.554	130	0.263	0.007	0.021
UniRef90_J4VSC9_Fusobacterium_gonidiaformans	lesions	yes	1.400	130	0.256	0.007	0.022
UniRef90_E5BF39_Fusobacterium_gonidiaformans	lesions	yes	1.856	130	0.248	0.007	0.022
UniRef90_A0A133NHX4_Fusobacterium_gonidiaformans	lesions	yes	1.678	130	0.256	0.007	0.022
UniRef90_E5BIA9_Fusobacterium_gonidiaformans	lesions	yes	1.474	130	0.263	0.007	0.022
UniRef90_E5BG84_Fusobacterium_gonidiaformans	lesions	yes	1.379	130	0.248	0.007	0.022
UniRef90_A0A017H4L3_Fusobacterium_gonidiaformans	lesions	yes	1.672	130	0.233	0.007	0.022
UniRef90_E5BEK0_Fusobacterium_gonidiaformans	lesions	yes	1.271	130	0.248	0.007	0.022
UniRef90_E5BGL0_Fusobacterium_gonidiaformans	lesions	yes	1.578	130	0.256	0.007	0.022
UniRef90_H1D5L6_Fusobacterium_gonidiaformans	lesions	yes	1.571	130	0.256	0.008	0.022
UniRef90_A0A134BDT6_Prevotella_bivia	lesions	yes	1.240	130	0.248	0.008	0.022
UniRef90_E5BHK0_Fusobacterium_gonidiaformans	lesions	yes	1.739	130	0.256	0.008	0.022
UniRef90_H1DB02_Fusobacterium_gonidiaformans	lesions	yes	1.821	130	0.248	0.008	0.022
UniRef90_C6M392_Neisseria_sicca	lesions	yes	0.957	130	0.241	0.008	0.022
UniRef90_A0A017H413_Fusobacterium_gonidiaformans	lesions	yes	1.334	130	0.256	0.008	0.023
UniRef90_H1DC91_Fusobacterium_gonidiaformans	lesions	yes	1.515	130	0.256	0.008	0.023
UniRef90_J5U0S5_Fusobacterium_gonidiaformans	lesions	yes	1.345	130	0.256	0.008	0.023
UniRef90_E5BEF2_Fusobacterium_gonidiaformans	lesions	yes	1.608	130	0.233	0.008	0.023
UniRef90_H1D557_Fusobacterium_gonidiaformans	lesions	yes	1.699	130	0.256	0.008	0.023
UniRef90_H1DAZ8_Fusobacterium_gonidiaformans	lesions	yes	1.485	130	0.256	0.008	0.023
UniRef90_E5BG53_Fusobacterium_gonidiaformans	lesions	yes	1.524	130	0.256	0.008	0.023
UniRef90_A0A133NFK6_Fusobacterium_gonidiaformans	lesions	yes	1.492	130	0.256	0.008	0.023
UniRef90_E5BE96_Fusobacterium_gonidiaformans	lesions	yes	1.612	130	0.256	0.008	0.023

UniRef90_A0A133N8A6_Fusobacterium_gonidiaformans	lesions	yes	1.482	130	0.256	0.008	0.023
UniRef90_E5BFX8_Fusobacterium_gonidiaformans	lesions	yes	1.315	130	0.241	0.008	0.023
UniRef90_E5BH43_Fusobacterium_gonidiaformans	lesions	yes	1.421	130	0.256	0.008	0.023
UniRef90_K6ADG5_Prevotella_bivia	lesions	yes	1.347	130	0.248	0.008	0.023
UniRef90_E5BHR3_Fusobacterium_gonidiaformans	lesions	yes	1.441	130	0.256	0.008	0.023
UniRef90_A0A161QVQ1_Fusobacterium_gonidiaformans	lesions	yes	1.354	130	0.256	0.008	0.024
UniRef90_E5BE99_Fusobacterium_gonidiaformans	lesions	yes	1.448	130	0.256	0.008	0.024
UniRef90_A0A133NBJ9_Fusobacterium_gonidiaformans	lesions	yes	1.171	130	0.248	0.008	0.024
UniRef90_E5BFQ2_Fusobacterium_gonidiaformans	lesions	yes	1.232	130	0.248	0.008	0.024
UniRef90_E5BDP6_Fusobacterium_gonidiaformans	lesions	yes	1.479	130	0.248	0.008	0.024
UniRef90_E5BGF1_Fusobacterium_gonidiaformans	lesions	yes	1.532	130	0.248	0.008	0.024
UniRef90_E5BIB7_Fusobacterium_gonidiaformans	lesions	yes	1.428	130	0.263	0.008	0.024
UniRef90_E5BDT6_Fusobacterium_gonidiaformans	lesions	yes	1.473	130	0.256	0.008	0.024
UniRef90_J5U0V7_Fusobacterium_gonidiaformans	lesions	yes	1.527	130	0.248	0.008	0.024
UniRef90_E5BEE9_Fusobacterium_gonidiaformans	lesions	yes	1.401	130	0.241	0.008	0.024
UniRef90_A0A162JFV4_Fusobacterium_gonidiaformans	lesions	yes	1.555	130	0.248	0.008	0.025
UniRef90_E5BHC1_Fusobacterium_gonidiaformans	lesions	yes	1.512	130	0.256	0.008	0.025
UniRef90_A0A170MXP9_Fusobacterium_gonidiaformans	lesions	yes	1.506	130	0.256	0.008	0.025
UniRef90_E5BI58_Fusobacterium_gonidiaformans	lesions	yes	1.542	130	0.256	0.008	0.025
UniRef90_E5BHP0_Fusobacterium_gonidiaformans	lesions	yes	1.283	130	0.248	0.008	0.025
UniRef90_E5BI68_Fusobacterium_gonidiaformans	lesions	yes	1.480	130	0.233	0.008	0.025
UniRef90_H1DB47_Fusobacterium_gonidiaformans	lesions	yes	1.289	130	0.263	0.008	0.025
UniRef90_H1DA00_Fusobacterium_gonidiaformans	lesions	yes	1.525	130	0.256	0.009	0.025
UniRef90_H1DAK6_Fusobacterium_gonidiaformans	lesions	yes	1.490	130	0.256	0.009	0.025
UniRef90_E5BH83_Fusobacterium_gonidiaformans	lesions	yes	1.146	130	0.256	0.009	0.025
UniRef90_A0A161PQP8_Fusobacterium_gonidiaformans	lesions	yes	1.545	130	0.256	0.009	0.025
UniRef90_J5TZL9_Fusobacterium_gonidiaformans	lesions	yes	1.568	130	0.256	0.009	0.026
UniRef90_E5BF27_Fusobacterium_gonidiaformans	lesions	yes	1.503	130	0.233	0.009	0.026
UniRef90_A0A017H8L6_Fusobacterium_gonidiaformans	lesions	yes	1.405	130	0.256	0.009	0.026
UniRef90_G6C1L6_Fusobacterium_gonidiaformans	lesions	yes	1.774	130	0.263	0.009	0.026
UniRef90_E5BDF8_Fusobacterium_gonidiaformans	lesions	yes	1.529	130	0.248	0.009	0.026
UniRef90_E5BIE2_Fusobacterium_gonidiaformans	lesions	yes	1.461	130	0.256	0.009	0.026
UniRef90_A0A133NC54_Fusobacterium_gonidiaformans	lesions	yes	1.240	130	0.241	0.009	0.026
UniRef90_H1DAB1_Fusobacterium_gonidiaformans	lesions	yes	1.552	130	0.256	0.009	0.026
UniRef90_J6H211_Fusobacterium_gonidiaformans	lesions	yes	1.433	130	0.263	0.009	0.026
UniRef90_A0A133NA98_Fusobacterium_gonidiaformans	lesions	yes	1.542	130	0.256	0.009	0.026
UniRef90_E5BG47_Fusobacterium_gonidiaformans	lesions	yes	1.589	130	0.256	0.009	0.026
UniRef90_A0A0B4FLQ4_Fusobacterium_gonidiaformans	lesions	yes	1.773	130	0.256	0.009	0.026
UniRef90_D1JU46_Prevotella_bivia	lesions	yes	1.007	130	0.233	0.009	0.027
UniRef90_J5TTA1_Fusobacterium_gonidiaformans	lesions	yes	1.469	130	0.256	0.009	0.027
UniRef90_J8VXQ7_Fusobacterium_gonidiaformans	lesions	yes	1.333	130	0.263	0.009	0.027
UniRef90_A0A017H3T2_Fusobacterium_gonidiaformans	lesions	yes	1.300	130	0.248	0.009	0.027
UniRef90_A0A133NH95_Fusobacterium_gonidiaformans	lesions	yes	1.332	130	0.256	0.009	0.027
UniRef90_H1DC45_Fusobacterium_gonidiaformans	lesions	yes	1.563	130	0.248	0.009	0.027
UniRef90_A0A133NBL1_Fusobacterium_gonidiaformans	lesions	yes	1.536	130	0.256	0.009	0.027
UniRef90_E5BEB7_Fusobacterium_gonidiaformans	lesions	yes	1.387	130	0.248	0.009	0.027
UniRef90_A0A015YJI0_Prevotella_bivia	lesions	yes	1.286	130	0.368	0.009	0.028
UniRef90_E5BIC8_Fusobacterium_gonidiaformans	lesions	yes	1.359	130	0.256	0.009	0.028
UniRef90_E5BH65_Fusobacterium_gonidiaformans	lesions	yes	1.529	130	0.241	0.009	0.028
UniRef90_A0A170MWD0_Fusobacterium_gonidiaformans	lesions	yes	1.619	130	0.248	0.009	0.028
UniRef90_A0A162ISQ1_Fusobacterium_gonidiaformans	lesions	yes	1.492	130	0.256	0.009	0.028
UniRef90_A0A0E2V4K6_Fusobacterium_gonidiaformans	lesions	yes	1.669	130	0.248	0.009	0.028
UniRef90_J5VTW9_Fusobacterium_gonidiaformans	lesions	yes	1.723	130	0.248	0.009	0.028
UniRef90_H1D5A8_Fusobacterium_gonidiaformans	lesions	yes	1.525	130	0.256	0.009	0.028
UniRef90_H1DCB8_Fusobacterium_gonidiaformans	lesions	yes	1.426	130	0.248	0.009	0.028
UniRef90_A0A161PSC6_Fusobacterium_gonidiaformans	lesions	yes	1.347	130	0.256	0.009	0.028

UniRef90_Q8RH45_Fusobacterium_gonidiaformans	lesions	yes	1.381	130	0.256	0.009	0.028
UniRef90_H1DAU1_Fusobacterium_gonidiaformans	lesions	yes	1.079	130	0.248	0.009	0.028
UniRef90_E5BGA8_Fusobacterium_gonidiaformans	lesions	yes	1.603	130	0.248	0.009	0.028
UniRef90_A0A064AN44_Fusobacterium_gonidiaformans	lesions	yes	1.456	130	0.256	0.009	0.028
UniRef90_A0A133NBL9_Fusobacterium_gonidiaformans	lesions	yes	1.653	130	0.256	0.009	0.028
UniRef90_C6MAB4_Neisseria_sicca	lesions	yes	0.842	130	0.226	0.010	0.028
UniRef90_H1DAH4_Fusobacterium_gonidiaformans	lesions	yes	1.380	130	0.256	0.010	0.028
UniRef90_A0A137SQU0_Prevotella_bivia	lesions	yes	1.289	130	0.226	0.010	0.028
UniRef90_E5BI91_Fusobacterium_gonidiaformans	lesions	yes	1.400	130	0.256	0.010	0.028
UniRef90_E5BIC3_Fusobacterium_gonidiaformans	lesions	yes	1.392	130	0.248	0.010	0.028
UniRef90_J6GS77_Fusobacterium_gonidiaformans	lesions	yes	1.548	130	0.233	0.010	0.028
UniRef90_E5BHT1_Fusobacterium_gonidiaformans	lesions	yes	1.007	130	0.248	0.010	0.029
UniRef90_E5BI85_Fusobacterium_gonidiaformans	lesions	yes	1.409	130	0.256	0.010	0.029
UniRef90_E5BGZ9_Fusobacterium_gonidiaformans	lesions	yes	1.534	130	0.248	0.010	0.029
UniRef90_E6K3S4_Prevotella_bivia	lesions	yes	1.337	130	0.278	0.010	0.029
UniRef90_A0A133NFI2_Fusobacterium_gonidiaformans	lesions	yes	1.592	130	0.256	0.010	0.029
UniRef90_E5BFQ1_Fusobacterium_gonidiaformans	lesions	yes	1.448	130	0.241	0.010	0.029
UniRef90_D6H9T7_Neisseria_flavescens	lesions	yes	1.021	130	0.233	0.010	0.029
UniRef90_E5BHI5_Fusobacterium_gonidiaformans	lesions	yes	1.439	130	0.248	0.010	0.029
UniRef90_E5BGF3_Fusobacterium_gonidiaformans	lesions	yes	1.831	130	0.248	0.010	0.029
UniRef90_E5BEK7_Fusobacterium_gonidiaformans	lesions	yes	1.181	130	0.248	0.010	0.029
UniRef90_E5BIM0_Fusobacterium_gonidiaformans	lesions	yes	1.410	130	0.256	0.010	0.029
UniRef90_A0A170MVM8_Fusobacterium_gonidiaformans	lesions	yes	1.495	130	0.248	0.010	0.029
UniRef90_E5BDI2_Fusobacterium_gonidiaformans	lesions	yes	1.284	130	0.248	0.010	0.029
UniRef90_I4Z7E0_Prevotella_bivia	lesions	yes	1.116	130	0.233	0.010	0.029
UniRef90_E5BDS4_Fusobacterium_gonidiaformans	lesions	yes	1.442	130	0.248	0.010	0.029
UniRef90_A0A162IJ86_Fusobacterium_gonidiaformans	lesions	yes	1.679	130	0.248	0.010	0.029
UniRef90_E5BEE8_Fusobacterium_gonidiaformans	lesions	yes	1.276	130	0.248	0.010	0.029
UniRef90_E5BH91_Fusobacterium_gonidiaformans	lesions	yes	1.345	130	0.248	0.010	0.030
UniRef90_E5BHS5_Fusobacterium_gonidiaformans	lesions	yes	1.592	130	0.248	0.010	0.030
UniRef90_Q8RIL9_Fusobacterium_gonidiaformans	lesions	yes	1.348	130	0.248	0.010	0.030
UniRef90_J4VNY5_Fusobacterium_gonidiaformans	lesions	yes	1.638	130	0.248	0.010	0.030
UniRef90_E5BG50_Fusobacterium_gonidiaformans	lesions	yes	1.601	130	0.256	0.010	0.030
UniRef90_Q8RIF8_Fusobacterium_gonidiaformans	lesions	yes	1.358	130	0.256	0.010	0.030
UniRef90_A0A0B4EN82_Fusobacterium_gonidiaformans	lesions	yes	1.370	130	0.233	0.010	0.030
UniRef90_E5BH66_Fusobacterium_gonidiaformans	lesions	yes	1.330	130	0.256	0.010	0.030
UniRef90_E5BGT0_Fusobacterium_gonidiaformans	lesions	yes	1.528	130	0.256	0.010	0.030
UniRef90_E5BH23_Fusobacterium_gonidiaformans	lesions	yes	1.243	130	0.248	0.010	0.030
UniRef90_E7NSS8_Fusobacterium_gonidiaformans	lesions	yes	1.441	130	0.263	0.010	0.030
UniRef90_E0N9D3_Neisseria_sicca	lesions	yes	0.682	130	0.256	0.010	0.030
UniRef90_E5BE56_Fusobacterium_gonidiaformans	lesions	yes	1.192	130	0.248	0.010	0.030
UniRef90_A0A154TFR0_Neisseria_sicca	lesions	yes	0.929	130	0.248	0.010	0.030
UniRef90_Q8RHI5_Fusobacterium_gonidiaformans	lesions	yes	1.294	130	0.233	0.010	0.031
UniRef90_J6H6C5_Fusobacterium_gonidiaformans	lesions	yes	1.389	130	0.248	0.010	0.031
UniRef90_E5BDZ1_Fusobacterium_gonidiaformans	lesions	yes	1.441	130	0.248	0.010	0.031
UniRef90_J5TPL8_Fusobacterium_gonidiaformans	lesions	yes	1.201	130	0.248	0.010	0.031
UniRef90_H1D614_Fusobacterium_gonidiaformans	lesions	yes	1.117	130	0.241	0.010	0.031
UniRef90_Q8RIN1_Fusobacterium_gonidiaformans	lesions	yes	1.571	130	0.263	0.010	0.031
UniRef90_E5BHM2_Fusobacterium_gonidiaformans	lesions	yes	1.404	130	0.248	0.011	0.031
UniRef90_E5BHG6_Fusobacterium_gonidiaformans	lesions	yes	1.455	130	0.248	0.011	0.031
UniRef90_E5BHT4_Fusobacterium_gonidiaformans	lesions	yes	1.189	130	0.248	0.011	0.031
UniRef90_E5BF90_Fusobacterium_gonidiaformans	lesions	yes	1.091	130	0.241	0.011	0.031
UniRef90_E5BIF8_Fusobacterium_gonidiaformans	lesions	yes	1.375	130	0.256	0.011	0.031
UniRef90_E5BIC5_Fusobacterium_gonidiaformans	lesions	yes	1.483	130	0.248	0.011	0.031
UniRef90_H1DCI6_Fusobacterium_gonidiaformans	lesions	yes	1.572	130	0.241	0.011	0.031
UniRef90_E5BG77_Fusobacterium_gonidiaformans	lesions	yes	1.132	130	0.241	0.011	0.031

UniRef90_J5VY99_Fusobacterium_gonidiaformans	lesions	yes	1.692	130	0.241	0.011	0.031
UniRef90_E5BHU4_Fusobacterium_gonidiaformans	lesions	yes	1.107	130	0.263	0.011	0.031
UniRef90_A0A396DXA9_Prevotella_bivia	lesions	yes	1.333	130	0.308	0.011	0.031
UniRef90_E5BEG4_Fusobacterium_gonidiaformans	lesions	yes	1.294	130	0.241	0.011	0.031
UniRef90_A0A3E4SC61_Prevotella_copri	lesions	yes	0.979	130	0.271	0.011	0.031
UniRef90_E5BDK2_Fusobacterium_gonidiaformans	lesions	yes	1.132	130	0.241	0.011	0.031
UniRef90_E5BI87_Fusobacterium_gonidiaformans	lesions	yes	1.417	130	0.256	0.011	0.032
UniRef90_H1D9K1_Fusobacterium_gonidiaformans	lesions	yes	1.486	130	0.241	0.011	0.032
UniRef90_A0A133NJ77_Fusobacterium_gonidiaformans	lesions	yes	1.419	130	0.256	0.011	0.032
UniRef90_E5BGS7_Fusobacterium_gonidiaformans	lesions	yes	1.478	130	0.248	0.011	0.032
UniRef90_A0A017H597_Fusobacterium_gonidiaformans	lesions	yes	1.519	130	0.248	0.011	0.032
UniRef90_A0A162J1E2_Fusobacterium_gonidiaformans	lesions	yes	1.473	130	0.241	0.011	0.032
UniRef90_H1D983_Fusobacterium_gonidiaformans	lesions	yes	1.567	130	0.248	0.011	0.032
UniRef90_E5BHM6_Fusobacterium_gonidiaformans	lesions	yes	1.253	130	0.248	0.011	0.032
UniRef90_A0A174ES80_Prevotella_bivia	lesions	yes	1.121	130	0.293	0.011	0.032
UniRef90_A0A133NFA4_Fusobacterium_gonidiaformans	lesions	yes	1.424	130	0.248	0.011	0.032
UniRef90_UPI000673CEC5_Fusobacterium_gonidiaformans	lesions	yes	1.402	130	0.271	0.011	0.032
UniRef90_A0A133ND89_Fusobacterium_gonidiaformans	lesions	yes	1.059	130	0.241	0.011	0.032
UniRef90_E5BG99_Fusobacterium_gonidiaformans	lesions	yes	1.227	130	0.248	0.011	0.033
UniRef90_E5BDI3_Fusobacterium_gonidiaformans	lesions	yes	1.424	130	0.248	0.011	0.033
UniRef90_A0A133NKG8_Fusobacterium_gonidiaformans	lesions	yes	1.279	130	0.256	0.011	0.033
UniRef90_Q8RIG2_Fusobacterium_gonidiaformans	lesions	yes	1.231	130	0.248	0.011	0.033
UniRef90_J5VR53_Fusobacterium_gonidiaformans	lesions	yes	1.543	130	0.248	0.011	0.033
UniRef90_H1D6H5_Fusobacterium_gonidiaformans	lesions	yes	1.680	130	0.248	0.011	0.033
UniRef90_A0A133NAV3_Fusobacterium_gonidiaformans	lesions	yes	1.479	130	0.248	0.011	0.033
UniRef90_E5BHM7_Fusobacterium_gonidiaformans	lesions	yes	1.710	130	0.241	0.011	0.033
UniRef90_E5BHV3_Fusobacterium_gonidiaformans	lesions	yes	1.272	130	0.233	0.011	0.033
UniRef90_H1D9Y7_Fusobacterium_gonidiaformans	lesions	yes	1.409	130	0.241	0.011	0.033
UniRef90_A0A133NGG4_Fusobacterium_gonidiaformans	lesions	yes	1.349	130	0.248	0.011	0.033
UniRef90_E5BHG2_Fusobacterium_gonidiaformans	lesions	yes	1.494	130	0.248	0.011	0.033
UniRef90_A0A064A2P7_Fusobacterium_gonidiaformans	lesions	yes	0.965	130	0.256	0.011	0.034
UniRef90_E5BHD8_Fusobacterium_gonidiaformans	lesions	yes	1.739	130	0.241	0.011	0.034
UniRef90_E5BF20_Fusobacterium_gonidiaformans	lesions	yes	1.394	130	0.241	0.011	0.034
UniRef90_A0A3E4SCF4_Prevotella_copri	lesions	yes	0.912	130	0.256	0.011	0.034
UniRef90_E5BHL8_Fusobacterium_gonidiaformans	lesions	yes	1.460	130	0.248	0.011	0.034
UniRef90_E5BHN9_Fusobacterium_gonidiaformans	lesions	yes	1.482	130	0.256	0.011	0.034
UniRef90_A0A133N6R1_Fusobacterium_gonidiaformans	lesions	yes	1.158	130	0.248	0.012	0.034
UniRef90_J5TWY1_Fusobacterium_gonidiaformans	lesions	yes	1.469	130	0.248	0.012	0.034
UniRef90_E5BHF8_Fusobacterium_gonidiaformans	lesions	yes	1.048	130	0.248	0.012	0.034
UniRef90_E5BHC9_Fusobacterium_gonidiaformans	lesions	yes	1.557	130	0.248	0.012	0.034
UniRef90_A0A133NKU8_Fusobacterium_gonidiaformans	lesions	yes	1.663	130	0.248	0.012	0.034
UniRef90_H1DBF7_Fusobacterium_gonidiaformans	lesions	yes	1.343	130	0.256	0.012	0.034
UniRef90_E5BHV6_Fusobacterium_gonidiaformans	lesions	yes	1.320	130	0.248	0.012	0.034
UniRef90_H1D6C3_Fusobacterium_gonidiaformans	lesions	yes	1.262	130	0.256	0.012	0.034
UniRef90_A0A133NAF9_Fusobacterium_gonidiaformans	lesions	yes	1.460	130	0.248	0.012	0.034
UniRef90_E5BHS6_Fusobacterium_gonidiaformans	lesions	yes	1.439	130	0.248	0.012	0.034
UniRef90_J6H0P2_Fusobacterium_gonidiaformans	lesions	yes	1.407	130	0.233	0.012	0.034
UniRef90_E5BHF3_Fusobacterium_gonidiaformans	lesions	yes	1.459	130	0.241	0.012	0.035
UniRef90_A0A133N8F7_Fusobacterium_gonidiaformans	lesions	yes	1.352	130	0.248	0.012	0.035
UniRef90_E5BGW4_Fusobacterium_gonidiaformans	lesions	yes	1.328	130	0.248	0.012	0.035
UniRef90_E5BGR0_Fusobacterium_gonidiaformans	lesions	yes	1.218	130	0.241	0.012	0.035
UniRef90_A0A133NA47_Fusobacterium_gonidiaformans	lesions	yes	1.471	130	0.248	0.012	0.035
UniRef90_E5BID2_Fusobacterium_gonidiaformans	lesions	yes	1.506	130	0.241	0.012	0.035
UniRef90_E5BHI0_Fusobacterium_gonidiaformans	lesions	yes	1.381	130	0.248	0.012	0.035
UniRef90_E5BH93_Fusobacterium_gonidiaformans	lesions	yes	1.379	130	0.256	0.012	0.035
UniRef90_A0A162J420_Fusobacterium_gonidiaformans	lesions	yes	1.124	130	0.256	0.012	0.035

UniRef90_E5BGX2_Fusobacterium_gonidiaformans	lesions	yes	1.026	130	0.248	0.012	0.035
UniRef90_A0A133NCV1_Fusobacterium_gonidiaformans	lesions	yes	1.376	130	0.241	0.012	0.035
UniRef90_A0A133NH66_Fusobacterium_gonidiaformans	lesions	yes	1.406	130	0.256	0.012	0.035
UniRef90_J5TRB5_Fusobacterium_gonidiaformans	lesions	yes	1.442	130	0.241	0.012	0.035
UniRef90_E5BH41_Fusobacterium_gonidiaformans	lesions	yes	1.529	130	0.248	0.012	0.035
UniRef90_E5BHP4_Fusobacterium_gonidiaformans	lesions	yes	1.461	130	0.241	0.012	0.035
UniRef90_E5BFU2_Fusobacterium_gonidiaformans	lesions	yes	1.233	130	0.233	0.012	0.035
UniRef90_H1D4M9_Fusobacterium_gonidiaformans	lesions	yes	1.368	130	0.248	0.012	0.036
UniRef90_E5BH95_Fusobacterium_gonidiaformans	lesions	yes	1.467	130	0.241	0.012	0.036
UniRef90_A0A0N7J6W0_Prevotella_bivia	lesions	yes	1.316	130	0.361	0.012	0.036
UniRef90_E7NST5_Fusobacterium_gonidiaformans	lesions	yes	1.368	130	0.248	0.012	0.036
UniRef90_H1DBQ3_Fusobacterium_gonidiaformans	lesions	yes	1.325	130	0.241	0.012	0.036
UniRef90_E5BI55_Fusobacterium_gonidiaformans	lesions	yes	1.128	130	0.241	0.012	0.036
UniRef90_A0A064A2B4_Fusobacterium_gonidiaformans	lesions	yes	1.321	130	0.233	0.012	0.036
UniRef90_E5BG96_Fusobacterium_gonidiaformans	lesions	yes	1.172	130	0.241	0.012	0.036
UniRef90_E5BEK1_Fusobacterium_gonidiaformans	lesions	yes	1.297	130	0.233	0.012	0.036
UniRef90_E5BHQ2_Fusobacterium_gonidiaformans	lesions	yes	1.474	130	0.241	0.012	0.036
UniRef90_H1D450_Fusobacterium_gonidiaformans	lesions	yes	1.670	130	0.233	0.012	0.036
UniRef90_E5BE51_Fusobacterium_gonidiaformans	lesions	yes	1.098	130	0.241	0.012	0.037
UniRef90_Q5F973_Neisseria_sicca	lesions	yes	0.786	130	0.233	0.013	0.037
UniRef90_E5BH12_Fusobacterium_gonidiaformans	lesions	yes	1.391	130	0.248	0.013	0.037
UniRef90_A0A096BRN9_Prevotella_bivia	lesions	yes	1.170	130	0.226	0.013	0.037
UniRef90_E5BHF5_Fusobacterium_gonidiaformans	lesions	yes	1.115	130	0.248	0.013	0.037
UniRef90_H1DAW4_Fusobacterium_gonidiaformans	lesions	yes	1.163	130	0.241	0.013	0.037
UniRef90_R6F7H6_Prevotella_sp_CAG_520	lesions	yes	0.644	130	0.233	0.013	0.037
UniRef90_E5BHR4_Fusobacterium_gonidiaformans	lesions	yes	1.318	130	0.248	0.013	0.037
UniRef90_E5BE97_Fusobacterium_gonidiaformans	lesions	yes	1.407	130	0.248	0.013	0.038
UniRef90_E5BFZ6_Fusobacterium_gonidiaformans	lesions	yes	1.660	130	0.241	0.013	0.038
UniRef90_A0A162J1U5_Fusobacterium_gonidiaformans	lesions	yes	1.231	130	0.241	0.013	0.038
UniRef90_E5BE11_Fusobacterium_gonidiaformans	lesions	yes	1.496	130	0.248	0.013	0.038
UniRef90_D8DX56_Prevotella_copri	lesions	yes	0.847	130	0.233	0.013	0.038
UniRef90_A0A162IGY5_Fusobacterium_gonidiaformans	lesions	yes	1.134	130	0.233	0.013	0.038
UniRef90_A0A133N7F0_Fusobacterium_gonidiaformans	lesions	yes	1.148	130	0.233	0.013	0.038
UniRef90_E5BGD5_Fusobacterium_gonidiaformans	lesions	yes	1.347	130	0.248	0.013	0.038
UniRef90_J8W720_Fusobacterium_gonidiaformans	lesions	yes	1.377	130	0.256	0.013	0.038
UniRef90_A0A017H3N9_Fusobacterium_gonidiaformans	lesions	yes	1.296	130	0.226	0.013	0.038
UniRef90_E5BHM5_Fusobacterium_gonidiaformans	lesions	yes	1.395	130	0.248	0.013	0.039
UniRef90_E5BGZ4_Fusobacterium_gonidiaformans	lesions	yes	1.133	130	0.233	0.013	0.039
UniRef90_A0A0E0TNL9_Neisseria_sicca	lesions	yes	0.809	130	0.278	0.013	0.039
UniRef90_E5BF22_Fusobacterium_gonidiaformans	lesions	yes	1.335	130	0.248	0.013	0.039
UniRef90_H1D5V7_Fusobacterium_gonidiaformans	lesions	yes	1.386	130	0.226	0.013	0.039
UniRef90_E5BHP1_Fusobacterium_gonidiaformans	lesions	yes	1.390	130	0.241	0.013	0.039
UniRef90_A0A154TSM8_Neisseria_sicca	lesions	yes	0.685	130	0.226	0.013	0.039
UniRef90_E5BGC0_Fusobacterium_gonidiaformans	lesions	yes	1.357	130	0.256	0.013	0.039
UniRef90_UPI00066454A0_Fusobacterium_gonidiaformans	lesions	yes	1.571	130	0.278	0.013	0.039
UniRef90_U7UPK0_Prevotella_bivia	lesions	yes	1.420	130	0.353	0.013	0.039
UniRef90_H1DA75_Fusobacterium_gonidiaformans	lesions	yes	1.255	130	0.248	0.013	0.039
UniRef90_Q8RIH0_Fusobacterium_gonidiaformans	lesions	yes	1.029	130	0.241	0.013	0.039
UniRef90_E5BGN9_Fusobacterium_gonidiaformans	lesions	yes	1.425	130	0.248	0.013	0.039
UniRef90_H1DAY1_Fusobacterium_gonidiaformans	lesions	yes	1.538	130	0.241	0.014	0.040
UniRef90_E5BE90_Fusobacterium_gonidiaformans	lesions	yes	1.321	130	0.241	0.014	0.040
UniRef90_R6EY10_Prevotella_stercorea	lesions	yes	1.017	130	0.233	0.014	0.040
UniRef90_E5BED5_Fusobacterium_gonidiaformans	lesions	yes	1.346	130	0.233	0.014	0.040
UniRef90_I1ZJ40_Streptococcus_mitis	lesions	yes	1.354	130	0.271	0.014	0.040
UniRef90_E5BEF0_Fusobacterium_gonidiaformans	lesions	yes	1.059	130	0.248	0.014	0.040
UniRef90_A0A133NB60_Fusobacterium_gonidiaformans	lesions	yes	1.571	130	0.263	0.014	0.040

UniRef90_H1D6L0_Fusobacterium_gonidiaformans	lesions	yes	1.415	130	0.241	0.014	0.040
UniRef90_E5BEB8_Fusobacterium_gonidiaformans	lesions	yes	1.277	130	0.256	0.014	0.040
UniRef90_E5BDE4_Fusobacterium_gonidiaformans	lesions	yes	1.405	130	0.256	0.014	0.040
UniRef90_E5BID1_Fusobacterium_gonidiaformans	lesions	yes	1.405	130	0.241	0.014	0.040
UniRef90_E5BGS4_Fusobacterium_gonidiaformans	lesions	yes	1.010	130	0.241	0.014	0.040
UniRef90_E5BDZ0_Fusobacterium_gonidiaformans	lesions	yes	1.325	130	0.241	0.014	0.040
UniRef90_D4IQJ1_Prevotella_bivia	lesions	yes	1.262	130	0.308	0.014	0.040
UniRef90_A0A154TSG0_Neisseria_sicca	lesions	yes	0.844	130	0.241	0.014	0.041
UniRef90_E5BGG3_Fusobacterium_gonidiaformans	lesions	yes	1.074	130	0.241	0.014	0.041
UniRef90_H1D4N2_Fusobacterium_gonidiaformans	lesions	yes	1.337	130	0.241	0.014	0.041
UniRef90_E5BGH0_Fusobacterium_gonidiaformans	lesions	yes	1.111	130	0.233	0.014	0.041
UniRef90_J8VWY4_Fusobacterium_gonidiaformans	lesions	yes	1.233	130	0.248	0.014	0.041
UniRef90_A0A3E4SDY1_Prevotella_copri	lesions	yes	0.851	130	0.241	0.014	0.041
UniRef90_E5BHP6_Fusobacterium_gonidiaformans	lesions	yes	1.293	130	0.248	0.014	0.041
UniRef90_E5BE16_Fusobacterium_gonidiaformans	lesions	yes	1.441	130	0.248	0.014	0.041
UniRef90_E5BDK7_Fusobacterium_gonidiaformans	lesions	yes	1.344	130	0.226	0.014	0.041
UniRef90_E5BH59_Fusobacterium_gonidiaformans	lesions	yes	1.204	130	0.241	0.014	0.041
UniRef90_E5BIL0_Fusobacterium_gonidiaformans	lesions	yes	1.338	130	0.256	0.014	0.041
UniRef90_E5BHD9_Fusobacterium_gonidiaformans	lesions	yes	1.034	130	0.241	0.014	0.041
UniRef90_E5BGH9_Fusobacterium_gonidiaformans	lesions	yes	1.503	130	0.226	0.014	0.041
UniRef90_E5BGQ1_Fusobacterium_gonidiaformans	lesions	yes	1.366	130	0.241	0.014	0.041
UniRef90_H1D9B8_Fusobacterium_gonidiaformans	lesions	yes	1.151	130	0.248	0.014	0.042
UniRef90_E5BE71_Fusobacterium_gonidiaformans	lesions	yes	1.359	130	0.241	0.014	0.042
UniRef90_J5VY32_Fusobacterium_gonidiaformans	lesions	yes	1.224	130	0.241	0.014	0.042
UniRef90_A0A0B4EJM6_Fusobacterium_gonidiaformans	lesions	yes	1.291	130	0.248	0.014	0.042
UniRef90_E5BHU9_Fusobacterium_gonidiaformans	lesions	yes	1.456	130	0.248	0.014	0.042
UniRef90_J6H6V7_Fusobacterium_gonidiaformans	lesions	yes	1.082	130	0.241	0.015	0.042
UniRef90_E5BHA8_Fusobacterium_gonidiaformans	lesions	yes	1.291	130	0.271	0.015	0.042
UniRef90_E5BH24_Fusobacterium_gonidiaformans	lesions	yes	1.172	130	0.248	0.015	0.042
UniRef90_E5BHC6_Fusobacterium_gonidiaformans	lesions	yes	1.015	130	0.248	0.015	0.042
UniRef90_E5BI98_Fusobacterium_gonidiaformans	lesions	yes	1.226	130	0.233	0.015	0.043
UniRef90_J5TZB1_Fusobacterium_gonidiaformans	lesions	yes	1.745	130	0.241	0.015	0.043
UniRef90_E5BHT3_Fusobacterium_gonidiaformans	lesions	yes	1.396	130	0.248	0.015	0.043
UniRef90_Q4QLX6_Haemophilus_parainfluenzae	lesions	yes	0.961	130	0.346	0.015	0.043
UniRef90_H1DCB5_Fusobacterium_gonidiaformans	lesions	yes	1.327	130	0.233	0.015	0.043
UniRef90_E5BGD4_Fusobacterium_gonidiaformans	lesions	yes	1.460	130	0.241	0.015	0.043
UniRef90_E5BI86_Fusobacterium_gonidiaformans	lesions	yes	1.362	130	0.241	0.015	0.043
UniRef90_J8W6C6_Fusobacterium_gonidiaformans	lesions	yes	1.436	130	0.241	0.015	0.043
UniRef90_E5BH13_Fusobacterium_gonidiaformans	lesions	yes	1.193	130	0.248	0.015	0.044
UniRef90_E5BHM8_Fusobacterium_gonidiaformans	lesions	yes	1.232	130	0.241	0.015	0.044
UniRef90_A0A133ND81_Fusobacterium_gonidiaformans	lesions	yes	1.005	130	0.248	0.015	0.044
UniRef90_E5BDS5_Fusobacterium_gonidiaformans	lesions	yes	1.204	130	0.241	0.015	0.044
UniRef90_E5BHL4_Fusobacterium_gonidiaformans	lesions	yes	0.910	130	0.248	0.015	0.044
UniRef90_A0A133NJ69_Fusobacterium_gonidiaformans	lesions	yes	0.924	130	0.241	0.015	0.044
UniRef90_J4VL88_Fusobacterium_gonidiaformans	lesions	yes	0.891	130	0.233	0.015	0.044
UniRef90_E5BDL8_Fusobacterium_gonidiaformans	lesions	yes	1.196	130	0.233	0.015	0.044
UniRef90_A0A133NKC1_Fusobacterium_gonidiaformans	lesions	yes	1.097	130	0.233	0.015	0.044
UniRef90_E5BHN5_Fusobacterium_gonidiaformans	lesions	yes	1.246	130	0.248	0.015	0.044
UniRef90_A0A0E2V4L1_Fusobacterium_gonidiaformans	lesions	yes	0.979	130	0.241	0.015	0.044
UniRef90_E5BGW3_Fusobacterium_gonidiaformans	lesions	yes	1.014	130	0.226	0.015	0.045
UniRef90_E5BGI5_Fusobacterium_gonidiaformans	lesions	yes	0.964	130	0.241	0.015	0.045
UniRef90_H1D667_Fusobacterium_gonidiaformans	lesions	yes	1.117	130	0.233	0.015	0.045
UniRef90_A0A3E5DK23_Prevotella_bivia	lesions	yes	1.508	130	0.338	0.015	0.045
UniRef90_Q5F8T6_Neisseria_sicca	lesions	yes	0.730	130	0.226	0.015	0.045
UniRef90_E5BGD1_Fusobacterium_gonidiaformans	lesions	yes	1.260	130	0.271	0.015	0.045
UniRef90_A0A154U8W3_Neisseria_sicca	lesions	yes	0.938	130	0.256	0.016	0.045

UniRef90_E5BIA1_Fusobacterium_gonidiaformans	lesions	yes	0.988	130	0.233	0.016	0.045
UniRef90_A9M173_Neisseria_sicca	lesions	yes	0.726	130	0.248	0.016	0.045
UniRef90_H1DAD3_Fusobacterium_gonidiaformans	lesions	yes	1.427	130	0.248	0.016	0.045
UniRef90_E5BE08_Fusobacterium_gonidiaformans	lesions	yes	1.307	130	0.241	0.016	0.045
UniRef90_J6H0E7_Fusobacterium_gonidiaformans	lesions	yes	1.375	130	0.241	0.016	0.045
UniRef90_E5BH72_Fusobacterium_gonidiaformans	lesions	yes	1.153	130	0.226	0.016	0.045
UniRef90_E5BDU6_Fusobacterium_gonidiaformans	lesions	yes	1.270	130	0.241	0.016	0.045
UniRef90_I2NX17_Haemophilus_parainfluenzae	lesions	yes	0.854	130	0.263	0.016	0.045
UniRef90_E5BHJ2_Fusobacterium_gonidiaformans	lesions	yes	1.256	130	0.241	0.016	0.045
UniRef90_E5BFY6_Fusobacterium_gonidiaformans	lesions	yes	1.257	130	0.233	0.016	0.046
UniRef90_E5BGN6_Fusobacterium_gonidiaformans	lesions	yes	1.201	130	0.248	0.016	0.046
UniRef90_E5BDN8_Fusobacterium_gonidiaformans	lesions	yes	1.211	130	0.241	0.016	0.046
UniRef90_H1DC48_Fusobacterium_gonidiaformans	lesions	yes	1.060	130	0.226	0.016	0.046
UniRef90_H1D938_Fusobacterium_gonidiaformans	lesions	yes	1.354	130	0.233	0.016	0.046
UniRef90_A0A133N9Z3_Fusobacterium_gonidiaformans	lesions	yes	1.036	130	0.248	0.016	0.046
UniRef90_H1DBG3_Fusobacterium_gonidiaformans	lesions	yes	1.259	130	0.226	0.016	0.046
UniRef90_G6C7I4_Streptococcus_mitis	lesions	yes	1.355	130	0.376	0.016	0.046
UniRef90_D6H7T8_Neisseria_sicca	lesions	yes	0.849	130	0.256	0.016	0.046
UniRef90_E5BIN8_Fusobacterium_gonidiaformans	lesions	yes	1.387	130	0.248	0.016	0.046
UniRef90_Q8RIH3_Fusobacterium_gonidiaformans	lesions	yes	0.996	130	0.241	0.016	0.046
UniRef90_J4VND1_Fusobacterium_gonidiaformans	lesions	yes	1.240	130	0.233	0.016	0.046
UniRef90_E5BE64_Fusobacterium_gonidiaformans	lesions	yes	1.297	130	0.241	0.016	0.046
UniRef90_A0A133NKV0_Fusobacterium_gonidiaformans	lesions	yes	1.278	130	0.241	0.016	0.046
UniRef90_A0A017H261_Fusobacterium_gonidiaformans	lesions	yes	1.458	130	0.271	0.016	0.046
UniRef90_A0A162IJU1_Fusobacterium_gonidiaformans	lesions	yes	1.015	130	0.226	0.016	0.046
UniRef90_H1D570_Fusobacterium_gonidiaformans	lesions	yes	1.477	130	0.241	0.016	0.047
UniRef90_E5BGI3_Fusobacterium_gonidiaformans	lesions	yes	1.315	130	0.233	0.016	0.047
UniRef90_A0A0F2CNW2_Streptococcus_oralis	lesions	yes	1.456	130	0.308	0.016	0.047
UniRef90_A0A133NHI1_Fusobacterium_gonidiaformans	lesions	yes	1.483	130	0.271	0.016	0.047
UniRef90_E4VR83_Prevotella_bivia	lesions	yes	1.192	130	0.256	0.016	0.047
UniRef90_H1D7X0_Fusobacterium_gonidiaformans	lesions	yes	1.304	130	0.248	0.016	0.047
UniRef90_H1D870_Fusobacterium_gonidiaformans	lesions	yes	1.261	130	0.241	0.016	0.047
UniRef90_E5BIJ7_Fusobacterium_gonidiaformans	lesions	yes	1.161	130	0.241	0.016	0.047
UniRef90_E5BE22_Fusobacterium_gonidiaformans	lesions	yes	1.406	130	0.241	0.016	0.047
UniRef90_A0A139LMC3_Prevotella_bivia	lesions	yes	1.250	130	0.308	0.016	0.047
UniRef90_J4VL59_Fusobacterium_gonidiaformans	lesions	yes	0.975	130	0.248	0.016	0.047
UniRef90_E5BI53_Fusobacterium_gonidiaformans	lesions	yes	1.362	130	0.241	0.016	0.047
UniRef90_J6H0L2_Fusobacterium_gonidiaformans	lesions	yes	1.198	130	0.241	0.016	0.047
UniRef90_E5BG58_Fusobacterium_gonidiaformans	lesions	yes	1.153	130	0.233	0.016	0.047
UniRef90_L1PB72_Prevotella_bivia	lesions	yes	0.791	130	0.368	0.016	0.048
UniRef90_A0A133N8B6_Fusobacterium_gonidiaformans	lesions	yes	1.253	130	0.248	0.017	0.048
UniRef90_A0A3E4SCJ8_Prevotella_copri	lesions	yes	1.187	130	0.248	0.017	0.048
UniRef90_H1DAV3_Fusobacterium_gonidiaformans	lesions	yes	1.341	130	0.241	0.017	0.048
UniRef90_A0A133NAZ2_Fusobacterium_gonidiaformans	lesions	yes	1.320	130	0.248	0.017	0.048
UniRef90_A0A133NEZ7_Fusobacterium_gonidiaformans	lesions	yes	1.117	130	0.241	0.017	0.048
UniRef90_E5BG67_Fusobacterium_gonidiaformans	lesions	yes	1.256	130	0.241	0.017	0.049
UniRef90_D7N3U2_Neisseria_sicca	lesions	yes	0.753	130	0.233	0.017	0.049
UniRef90_A0A0B4FQ23_Fusobacterium_gonidiaformans	lesions	yes	1.187	130	0.248	0.017	0.049
UniRef90_A0A162IHJ1_Fusobacterium_gonidiaformans	lesions	yes	0.980	130	0.241	0.017	0.049
UniRef90_H1D5W4_Fusobacterium_gonidiaformans	lesions	yes	1.414	130	0.248	0.017	0.049
UniRef90_E5BGG1_Fusobacterium_gonidiaformans	lesions	yes	1.002	130	0.241	0.017	0.049
UniRef90_H1Q2D5_Prevotella_bivia	lesions	yes	0.983	130	0.271	0.017	0.049
UniRef90_H1D3Z1_Fusobacterium_gonidiaformans	lesions	yes	1.318	130	0.233	0.017	0.049
UniRef90_F2B9R9_Neisseria_flavescens	lesions	yes	0.984	130	0.241	0.017	0.049
UniRef90_E5BG18_Fusobacterium_gonidiaformans	lesions	yes	1.473	130	0.241	0.017	0.049
UniRef90_E5BHI2_Fusobacterium_gonidiaformans	lesions	yes	1.307	130	0.241	0.017	0.049

UniRef90_E5BG60_Fusobacterium_gonidiaformans	lesions	yes	1.337	130	0.241	0.017	0.049
UniRef90_E5BEB6_Fusobacterium_gonidiaformans	lesions	yes	1.024	130	0.233	0.017	0.049
UniRef90_E5BE15_Fusobacterium_gonidiaformans	lesions	yes	1.413	130	0.226	0.017	0.049
UniRef90_E5BGF6_Fusobacterium_gonidiaformans	lesions	yes	1.252	130	0.241	0.017	0.049
UniRef90_E5BH15_Fusobacterium_gonidiaformans	lesions	yes	1.203	130	0.241	0.017	0.049
UniRef90_Q04HV7_Streptococcus_oralis	lesions	yes	0.962	130	0.376	0.017	0.049
UniRef90_H1D434_Fusobacterium_gonidiaformans	lesions	yes	1.352	130	0.233	0.017	0.049
UniRef90_E5BHU0_Fusobacterium_gonidiaformans	lesions	yes	1.244	130	0.248	0.017	0.050
UniRef90_A0A170MVJ3_Fusobacterium_gonidiaformans	lesions	yes	0.973	130	0.241	0.017	0.050
UniRef90_Q8RE45_Fusobacterium_gonidiaformans	lesions	yes	1.130	130	0.226	0.017	0.050
UniRef90_E5BHB2_Fusobacterium_gonidiaformans	lesions	yes	1.027	130	0.233	0.017	0.050
UniRef90_E5BG21_Fusobacterium_gonidiaformans	lesions	yes	1.456	130	0.241	0.017	0.050
UniRef90_A0A133NH60_Fusobacterium_gonidiaformans	lesions	yes	0.919	130	0.241	0.017	0.050
UniRef90_H1D620_Fusobacterium_gonidiaformans	lesions	yes	1.229	130	0.241	0.017	0.050
UniRef90_H1D8M9_Fusobacterium_gonidiaformans	lesions	yes	1.014	130	0.241	0.017	0.050
UniRef90_E5BE52_Fusobacterium_gonidiaformans	lesions	yes	1.108	130	0.233	0.017	0.050
UniRef90_A0A017H5N7_Fusobacterium_gonidiaformans	lesions	yes	1.327	130	0.263	0.017	0.050
UniRef90_E5BH87_Fusobacterium_gonidiaformans	lesions	yes	1.270	130	0.241	0.017	0.050
UniRef90_D4WMR9_Prevotella_bivia	lesions	yes	1.311	130	0.338	0.017	0.050
UniRef90_A0A133N875_Fusobacterium_gonidiaformans	lesions	yes	1.216	130	0.226	0.017	0.050
UniRef90_A0A096BR03_Prevotella_bivia	lesions	yes	0.944	130	0.226	0.018	0.051
UniRef90_E5BDU1_Fusobacterium_gonidiaformans	lesions	yes	1.281	130	0.233	0.018	0.051
UniRef90_E5BDF5_Fusobacterium_gonidiaformans	lesions	yes	1.250	130	0.241	0.018	0.051
UniRef90_H1D3T8_Fusobacterium_gonidiaformans	lesions	yes	1.247	130	0.241	0.018	0.051
UniRef90_E5BDR3_Fusobacterium_gonidiaformans	lesions	yes	1.248	130	0.241	0.018	0.051
UniRef90_E5BG23_Fusobacterium_gonidiaformans	lesions	yes	0.968	130	0.241	0.018	0.051
UniRef90_H1D985_Fusobacterium_gonidiaformans	lesions	yes	1.396	130	0.233	0.018	0.051
UniRef90_E5BG51_Fusobacterium_gonidiaformans	lesions	yes	1.248	130	0.248	0.018	0.051
UniRef90_I2NX17_Neisseria_flavescens	lesions	yes	0.632	130	0.233	0.018	0.051
UniRef90_E5BH90_Fusobacterium_gonidiaformans	lesions	yes	1.461	130	0.233	0.018	0.051
UniRef90_E0N9D3_Neisseria_flavescens	lesions	yes	1.061	130	0.226	0.018	0.052
UniRef90_A0A133N6L0_Fusobacterium_gonidiaformans	lesions	yes	1.317	130	0.241	0.018	0.052
UniRef90_A0A096CIE9_Prevotella_bivia	lesions	yes	1.006	130	0.233	0.018	0.052
UniRef90_A0A2X1UHB2_Neisseria_sicca	lesions	yes	0.799	130	0.248	0.018	0.052
UniRef90_H1DA32_Fusobacterium_gonidiaformans	lesions	yes	1.241	130	0.241	0.018	0.052
UniRef90_E5BGN7_Fusobacterium_gonidiaformans	lesions	yes	1.396	130	0.241	0.018	0.052
UniRef90_E5BG81_Fusobacterium_gonidiaformans	lesions	yes	0.871	130	0.248	0.018	0.052
UniRef90_H1DBL0_Fusobacterium_gonidiaformans	lesions	yes	1.266	130	0.248	0.018	0.052
UniRef90_E5BH27_Fusobacterium_gonidiaformans	lesions	yes	0.980	130	0.233	0.018	0.052
UniRef90_H1D4N1_Fusobacterium_gonidiaformans	lesions	yes	1.316	130	0.241	0.018	0.052
UniRef90_Q9K0M3_Neisseria_sicca	lesions	yes	0.775	130	0.241	0.018	0.052
UniRef90_A0A133NBL8_Fusobacterium_gonidiaformans	lesions	yes	1.689	130	0.241	0.018	0.052
UniRef90_H1D807_Fusobacterium_gonidiaformans	lesions	yes	1.158	130	0.248	0.018	0.052
UniRef90_E5BDD2_Fusobacterium_gonidiaformans	lesions	yes	1.275	130	0.233	0.018	0.052
UniRef90_H1DBF3_Fusobacterium_gonidiaformans	lesions	yes	1.270	130	0.241	0.018	0.052
UniRef90_E5BI65_Fusobacterium_gonidiaformans	lesions	yes	1.287	130	0.271	0.018	0.053
UniRef90_A0A133NKH9_Fusobacterium_gonidiaformans	lesions	yes	1.390	130	0.233	0.018	0.053
UniRef90_A0A133NBK2_Fusobacterium_gonidiaformans	lesions	yes	1.262	130	0.241	0.018	0.053
UniRef90_H1D6J2_Fusobacterium_gonidiaformans	lesions	yes	1.331	130	0.248	0.018	0.053
UniRef90_A0A174UG67_Prevotella_bivia	lesions	yes	0.956	130	0.361	0.018	0.053
UniRef90_H1DBU3_Fusobacterium_gonidiaformans	lesions	yes	0.956	130	0.233	0.018	0.053
UniRef90_E5BIL5_Fusobacterium_gonidiaformans	lesions	yes	0.893	130	0.248	0.019	0.053
UniRef90_H1DC90_Fusobacterium_gonidiaformans	lesions	yes	1.212	130	0.241	0.019	0.053
UniRef90_A0A0B4EXB7_Fusobacterium_gonidiaformans	lesions	yes	1.023	130	0.233	0.019	0.054
UniRef90_H1DA89_Fusobacterium_gonidiaformans	lesions	yes	1.623	130	0.271	0.019	0.054
UniRef90_E5BDF7_Fusobacterium_gonidiaformans	lesions	yes	0.962	130	0.233	0.019	0.054

UniRef90_E5BHH4_Fusobacterium_gonidiaformans	lesions	yes	1.330	130	0.241	0.019	0.054
UniRef90_H1D5A4_Fusobacterium_gonidiaformans	lesions	yes	1.235	130	0.241	0.019	0.054
UniRef90_E5BF31_Fusobacterium_gonidiaformans	lesions	yes	1.120	130	0.226	0.019	0.054
UniRef90_E5BGH8_Fusobacterium_gonidiaformans	lesions	yes	1.153	130	0.233	0.019	0.055
UniRef90_E5BGK5_Fusobacterium_gonidiaformans	lesions	yes	1.217	130	0.226	0.019	0.055
UniRef90_E5BF79_Fusobacterium_gonidiaformans	lesions	yes	1.183	130	0.241	0.019	0.055
UniRef90_H1DAE9_Fusobacterium_gonidiaformans	lesions	yes	0.917	130	0.241	0.019	0.055
UniRef90_A0A064A5D7_Fusobacterium_gonidiaformans	lesions	yes	1.258	130	0.241	0.019	0.055
UniRef90_A0A133NJV2_Fusobacterium_gonidiaformans	lesions	yes	1.296	130	0.233	0.019	0.055
UniRef90_H1DBG0_Fusobacterium_gonidiaformans	lesions	yes	1.154	130	0.248	0.019	0.055
UniRef90_E5BIF9_Fusobacterium_gonidiaformans	lesions	yes	1.351	130	0.241	0.019	0.055
UniRef90_E5BF72_Fusobacterium_gonidiaformans	lesions	yes	1.283	130	0.241	0.019	0.056
UniRef90_E5BH94_Fusobacterium_gonidiaformans	lesions	yes	1.251	130	0.233	0.019	0.056
UniRef90_E5BH22_Fusobacterium_gonidiaformans	lesions	yes	0.880	130	0.241	0.019	0.056
UniRef90_A0A133NKZ8_Fusobacterium_gonidiaformans	lesions	yes	1.245	130	0.241	0.019	0.056
UniRef90_A0A133N850_Fusobacterium_gonidiaformans	lesions	yes	1.132	130	0.233	0.019	0.056
UniRef90_H1DCH8_Fusobacterium_gonidiaformans	lesions	yes	1.194	130	0.241	0.019	0.056
UniRef90_E5BGU4_Fusobacterium_gonidiaformans	lesions	yes	1.180	130	0.233	0.019	0.056
UniRef90_E5BGL4_Fusobacterium_gonidiaformans	lesions	yes	1.254	130	0.233	0.019	0.056
UniRef90_A0A170MVS8_Fusobacterium_gonidiaformans	lesions	yes	1.137	130	0.241	0.019	0.056
UniRef90_E5BGT1_Fusobacterium_gonidiaformans	lesions	yes	1.581	130	0.233	0.020	0.056
UniRef90_E5BI71_Fusobacterium_gonidiaformans	lesions	yes	1.309	130	0.241	0.020	0.056
UniRef90_E5BDK0_Fusobacterium_gonidiaformans	lesions	yes	0.967	130	0.233	0.020	0.056
UniRef90_P30899_Prevotella_bivia	lesions	yes	0.655	130	0.361	0.020	0.056
UniRef90_E5BHU6_Fusobacterium_gonidiaformans	lesions	yes	1.018	130	0.248	0.020	0.056
UniRef90_A0A016FI57_Prevotella_bivia	lesions	yes	0.954	130	0.376	0.020	0.056
UniRef90_E5BEH6_Fusobacterium_gonidiaformans	lesions	yes	1.124	130	0.241	0.020	0.057
UniRef90_E5BGG5_Fusobacterium_gonidiaformans	lesions	yes	0.928	130	0.241	0.020	0.057
UniRef90_E5BHE6_Fusobacterium_gonidiaformans	lesions	yes	0.968	130	0.241	0.020	0.057
UniRef90_E5BGY0_Fusobacterium_gonidiaformans	lesions	yes	1.234	130	0.241	0.020	0.057
UniRef90_E5BH82_Fusobacterium_gonidiaformans	lesions	yes	1.357	130	0.233	0.020	0.057
UniRef90_F8N8L8_Prevotella_sp_CAG_520	lesions	yes	0.540	130	0.233	0.020	0.057
UniRef90_E5BHK1_Fusobacterium_gonidiaformans	lesions	yes	1.430	130	0.263	0.020	0.057
UniRef90_E5BF77_Fusobacterium_gonidiaformans	lesions	yes	1.255	130	0.233	0.020	0.057
UniRef90_E5BH86_Fusobacterium_gonidiaformans	lesions	yes	1.253	130	0.233	0.020	0.057
UniRef90_J4VWE2_Fusobacterium_gonidiaformans	lesions	yes	0.991	130	0.248	0.020	0.057
UniRef90_H1D3Q1_Fusobacterium_gonidiaformans	lesions	yes	1.225	130	0.241	0.020	0.057
UniRef90_A0A017H4D1_Fusobacterium_gonidiaformans	lesions	yes	1.270	130	0.241	0.020	0.058
UniRef90_E5BGZ8_Fusobacterium_gonidiaformans	lesions	yes	1.408	130	0.241	0.020	0.058
UniRef90_E5BE70_Fusobacterium_gonidiaformans	lesions	yes	1.047	130	0.233	0.020	0.058
UniRef90_E5BDK3_Fusobacterium_gonidiaformans	lesions	yes	0.928	130	0.226	0.020	0.058
UniRef90_E5BDP8_Fusobacterium_gonidiaformans	lesions	yes	1.165	130	0.241	0.020	0.058
UniRef90_E5BHK8_Fusobacterium_gonidiaformans	lesions	yes	0.910	130	0.226	0.020	0.058
UniRef90_E5BGS1_Fusobacterium_gonidiaformans	lesions	yes	1.197	130	0.241	0.020	0.058
UniRef90_H1DB92_Fusobacterium_gonidiaformans	lesions	yes	1.379	130	0.256	0.020	0.058
UniRef90_E5BEF7_Fusobacterium_gonidiaformans	lesions	yes	0.841	130	0.233	0.020	0.058
UniRef90_E5BH92_Fusobacterium_gonidiaformans	lesions	yes	1.203	130	0.226	0.020	0.058
UniRef90_E5BG12_Fusobacterium_gonidiaformans	lesions	yes	0.975	130	0.233	0.020	0.059
UniRef90_E5BDZ4_Fusobacterium_gonidiaformans	lesions	yes	1.370	130	0.241	0.021	0.059
UniRef90_H1DC01_Fusobacterium_gonidiaformans	lesions	yes	1.409	130	0.248	0.021	0.059
UniRef90_H1D3R0_Fusobacterium_gonidiaformans	lesions	yes	1.278	130	0.233	0.021	0.059
UniRef90_E5BEA7_Fusobacterium_gonidiaformans	lesions	yes	1.177	130	0.241	0.021	0.059
UniRef90_H1D827_Fusobacterium_gonidiaformans	lesions	yes	1.252	130	0.233	0.021	0.059
UniRef90_H1D6J1_Fusobacterium_gonidiaformans	lesions	yes	0.953	130	0.241	0.021	0.059
UniRef90_E5BHM1_Fusobacterium_gonidiaformans	lesions	yes	1.215	130	0.248	0.021	0.059
UniRef90_H1D3R4_Fusobacterium_gonidiaformans	lesions	yes	1.275	130	0.248	0.021	0.060

UniRef90_E5BEF1_Fusobacterium_gonidiaformans	lesions	yes	1.325	130	0.241	0.021	0.060
UniRef90_E5BDJ0_Fusobacterium_gonidiaformans	lesions	yes	1.342	130	0.233	0.021	0.060
UniRef90_E5BF85_Fusobacterium_gonidiaformans	lesions	yes	1.130	130	0.241	0.021	0.060
UniRef90_H1D4A6_Fusobacterium_gonidiaformans	lesions	yes	1.186	130	0.248	0.021	0.060
UniRef90_E5BGA1_Fusobacterium_gonidiaformans	lesions	yes	1.253	130	0.241	0.021	0.060
UniRef90_A0A133NIN2_Fusobacterium_gonidiaformans	lesions	yes	1.511	130	0.226	0.021	0.060
UniRef90_D4K4L9_Faecalibacterium_prausnitzii	lesions	yes	-0.822	130	0.241	0.021	0.060
UniRef90_E5BGQ5_Fusobacterium_gonidiaformans	lesions	yes	0.891	130	0.241	0.021	0.060
UniRef90_E5BE25_Fusobacterium_gonidiaformans	lesions	yes	1.425	130	0.241	0.021	0.060
UniRef90_E5BHB9_Fusobacterium_gonidiaformans	lesions	yes	1.058	130	0.233	0.021	0.061
UniRef90_E5BG34_Fusobacterium_gonidiaformans	lesions	yes	1.465	130	0.263	0.021	0.061
UniRef90_H1DBZ6_Fusobacterium_gonidiaformans	lesions	yes	1.258	130	0.241	0.021	0.061
UniRef90_P44569_Haemophilus_parainfluenzae	lesions	yes	0.814	130	0.286	0.021	0.061
UniRef90_P10337_Prevotella_bivia	lesions	yes	0.880	130	0.368	0.021	0.061
UniRef90_W6NZ48_Prevotella_bivia	lesions	yes	1.228	130	0.331	0.021	0.061
UniRef90_H1D3M2_Fusobacterium_gonidiaformans	lesions	yes	1.290	130	0.241	0.021	0.061
UniRef90_E5BDH4_Fusobacterium_gonidiaformans	lesions	yes	1.237	130	0.233	0.021	0.061
UniRef90_H1DC14_Fusobacterium_gonidiaformans	lesions	yes	0.908	130	0.241	0.021	0.061
UniRef90_E5BDX3_Fusobacterium_gonidiaformans	lesions	yes	1.645	130	0.233	0.021	0.061
UniRef90_E5BID3_Fusobacterium_gonidiaformans	lesions	yes	0.966	130	0.248	0.021	0.061
UniRef90_A0A264Y4J1_Prevotella_sp_885	lesions	yes	1.035	130	0.233	0.021	0.061
UniRef90_E5BH88_Fusobacterium_gonidiaformans	lesions	yes	1.226	130	0.241	0.021	0.061
UniRef90_H1DBB0_Fusobacterium_gonidiaformans	lesions	yes	0.890	130	0.233	0.021	0.061
UniRef90_J4VMW5_Fusobacterium_gonidiaformans	lesions	yes	0.917	130	0.241	0.022	0.062
UniRef90_H1D527_Fusobacterium_gonidiaformans	lesions	yes	1.389	130	0.233	0.022	0.062
UniRef90_A0A0B4ENE3_Fusobacterium_gonidiaformans	lesions	yes	0.979	130	0.226	0.022	0.062
UniRef90_E5BIC4_Fusobacterium_gonidiaformans	lesions	yes	1.042	130	0.233	0.022	0.062
UniRef90_E5BDR6_Fusobacterium_gonidiaformans	lesions	yes	1.250	130	0.226	0.022	0.062
UniRef90_A0A133NBG9_Fusobacterium_gonidiaformans	lesions	yes	1.215	130	0.241	0.022	0.062
UniRef90_E5BDD7_Fusobacterium_gonidiaformans	lesions	yes	1.181	130	0.233	0.022	0.062
UniRef90_E5BEB9_Fusobacterium_gonidiaformans	lesions	yes	1.257	130	0.248	0.022	0.063
UniRef90_UPI00050F3A5A_Prevotella_bivia	lesions	yes	0.823	130	0.316	0.022	0.063
UniRef90_Q8RG80_Fusobacterium_gonidiaformans	lesions	yes	1.214	130	0.233	0.022	0.063
UniRef90_P70718_Haemophilus_parainfluenzae	lesions	yes	0.921	130	0.361	0.022	0.063
UniRef90_H1D9X7_Fusobacterium_gonidiaformans	lesions	yes	0.898	130	0.233	0.022	0.063
UniRef90_E5BDQ1_Fusobacterium_gonidiaformans	lesions	yes	1.162	130	0.233	0.022	0.063
UniRef90_E5BHK6_Fusobacterium_gonidiaformans	lesions	yes	1.490	130	0.263	0.022	0.063
UniRef90_E5BGT9_Fusobacterium_gonidiaformans	lesions	yes	1.167	130	0.226	0.022	0.063
UniRef90_H1DA85_Fusobacterium_gonidiaformans	lesions	yes	1.161	130	0.233	0.022	0.063
UniRef90_E5BGI4_Fusobacterium_gonidiaformans	lesions	yes	1.123	130	0.233	0.022	0.064
UniRef90_A0A133NGH9_Fusobacterium_gonidiaformans	lesions	yes	1.327	130	0.271	0.022	0.064
UniRef90_A0A017H554_Fusobacterium_gonidiaformans	lesions	yes	1.277	130	0.226	0.022	0.064
UniRef90_E5UIQ8_Neisseria_sicca	lesions	yes	0.878	130	0.248	0.022	0.064
UniRef90_A0A133NA53_Fusobacterium_gonidiaformans	lesions	yes	1.171	130	0.241	0.022	0.064
UniRef90_E5BE24_Fusobacterium_gonidiaformans	lesions	yes	1.253	130	0.233	0.023	0.064
UniRef90_J5W3B7_Fusobacterium_gonidiaformans	lesions	yes	0.962	130	0.241	0.023	0.064
UniRef90_H1DC42_Fusobacterium_gonidiaformans	lesions	yes	1.173	130	0.241	0.023	0.064
UniRef90_H1D969_Fusobacterium_gonidiaformans	lesions	yes	1.234	130	0.226	0.023	0.065
UniRef90_A0A133NE39_Fusobacterium_gonidiaformans	lesions	yes	0.919	130	0.233	0.023	0.065
UniRef90_E5BGE3_Fusobacterium_gonidiaformans	lesions	yes	1.144	130	0.241	0.023	0.065
UniRef90_E5BIL4_Fusobacterium_gonidiaformans	lesions	yes	1.321	130	0.241	0.023	0.065
UniRef90_J8SR01_Fusobacterium_gonidiaformans	lesions	yes	1.221	130	0.233	0.023	0.065
UniRef90_P44891_Haemophilus_haemolyticus	lesions	yes	1.008	130	0.233	0.023	0.065
UniRef90_E5BFQ8_Fusobacterium_gonidiaformans	lesions	yes	1.276	130	0.226	0.023	0.065
UniRef90_E0NQW5_Prevotella_bivia	lesions	yes	1.389	130	0.286	0.023	0.065
UniRef90_A0A3A5BTL4_Neisseria_sicca	lesions	yes	0.700	130	0.248	0.023	0.065

UniRef90_E5BG90_Fusobacterium_gonidiaformans	lesions	yes	0.977	130	0.226	0.023	0.065
UniRef90_A0A0E9FAA4_Fusobacterium_gonidiaformans	lesions	yes	1.049	130	0.248	0.023	0.065
UniRef90_R6EWV8_Prevotella_stercorea	lesions	yes	0.716	130	0.233	0.023	0.065
UniRef90_E5BGE9_Fusobacterium_gonidiaformans	lesions	yes	0.925	130	0.241	0.023	0.066
UniRef90_H1D4M0_Fusobacterium_gonidiaformans	lesions	yes	0.899	130	0.226	0.023	0.066
UniRef90_A0A133NDP0_Fusobacterium_gonidiaformans	lesions	yes	1.227	130	0.233	0.023	0.066
UniRef90_A0A017H4L5_Fusobacterium_gonidiaformans	lesions	yes	1.215	130	0.241	0.023	0.066
UniRef90_E5BED6_Fusobacterium_gonidiaformans	lesions	yes	1.038	130	0.256	0.023	0.066
UniRef90_E5BEI8_Fusobacterium_gonidiaformans	lesions	yes	0.938	130	0.226	0.023	0.066
UniRef90_H1D9N9_Fusobacterium_gonidiaformans	lesions	yes	1.232	130	0.241	0.023	0.067
UniRef90_E5BG75_Fusobacterium_gonidiaformans	lesions	yes	1.081	130	0.226	0.024	0.067
UniRef90_A0A3E4SDA8_Prevotella_copri	lesions	yes	0.852	130	0.226	0.024	0.067
UniRef90_E5BG27_Fusobacterium_gonidiaformans	lesions	yes	1.160	130	0.233	0.024	0.067
UniRef90_E5BDZ9_Fusobacterium_gonidiaformans	lesions	yes	1.361	130	0.241	0.024	0.067
UniRef90_H1DA83_Fusobacterium_gonidiaformans	lesions	yes	0.927	130	0.241	0.024	0.067
UniRef90_A0A133N6K6_Fusobacterium_gonidiaformans	lesions	yes	1.141	130	0.226	0.024	0.067
UniRef90_E5BEC0_Fusobacterium_gonidiaformans	lesions	yes	0.961	130	0.248	0.024	0.067
UniRef90_I4ZBD6_Prevotella_bivia	lesions	yes	1.149	130	0.233	0.024	0.067
UniRef90_E5BFU6_Fusobacterium_gonidiaformans	lesions	yes	0.972	130	0.241	0.024	0.067
UniRef90_C5TK40_Neisseria_sicca	lesions	yes	0.973	130	0.233	0.024	0.067
UniRef90_E5BDY1_Fusobacterium_gonidiaformans	lesions	yes	1.263	130	0.248	0.024	0.067
UniRef90_A0A3E4SE83_Prevotella_copri	lesions	yes	0.660	130	0.248	0.024	0.067
UniRef90_H1D3Q8_Fusobacterium_gonidiaformans	lesions	yes	0.969	130	0.233	0.024	0.067
UniRef90_E5BEF6_Fusobacterium_gonidiaformans	lesions	yes	0.921	130	0.226	0.024	0.067
UniRef90_A0A170MY26_Fusobacterium_gonidiaformans	lesions	yes	0.906	130	0.233	0.024	0.068
UniRef90_E5BHK5_Fusobacterium_gonidiaformans	lesions	yes	1.003	130	0.226	0.024	0.068
UniRef90_A0A017H4G1_Fusobacterium_gonidiaformans	lesions	yes	1.358	130	0.263	0.024	0.068
UniRef90_E5BH19_Fusobacterium_gonidiaformans	lesions	yes	0.857	130	0.233	0.024	0.068
UniRef90_E5BGP1_Fusobacterium_gonidiaformans	lesions	yes	1.396	130	0.233	0.024	0.068
UniRef90_E5BF41_Fusobacterium_gonidiaformans	lesions	yes	1.335	130	0.233	0.024	0.068
UniRef90_H1DC72_Fusobacterium_gonidiaformans	lesions	yes	1.184	130	0.233	0.024	0.068
UniRef90_E5BE20_Fusobacterium_gonidiaformans	lesions	yes	1.118	130	0.233	0.024	0.068
UniRef90_A0A133NAS6_Fusobacterium_gonidiaformans	lesions	yes	0.885	130	0.226	0.024	0.068
UniRef90_E5BIL9_Fusobacterium_gonidiaformans	lesions	yes	1.333	130	0.271	0.024	0.068
UniRef90_H1D629_Fusobacterium_gonidiaformans	lesions	yes	1.038	130	0.233	0.024	0.069
UniRef90_E5BED0_Fusobacterium_gonidiaformans	lesions	yes	0.837	130	0.241	0.024	0.069
UniRef90_E5BFV0_Fusobacterium_gonidiaformans	lesions	yes	1.006	130	0.241	0.024	0.069
UniRef90_E5BGP7_Fusobacterium_gonidiaformans	lesions	yes	1.179	130	0.233	0.024	0.069
UniRef90_E5BHG7_Fusobacterium_gonidiaformans	lesions	yes	1.046	130	0.233	0.024	0.069
UniRef90_A0A137T0C1_Prevotella_bivia	lesions	yes	1.143	130	0.226	0.024	0.069
UniRef90_E5BGE1_Fusobacterium_gonidiaformans	lesions	yes	1.107	130	0.226	0.025	0.069
UniRef90_A0A017H5G0_Fusobacterium_gonidiaformans	lesions	yes	1.267	130	0.233	0.025	0.070
UniRef90_A0A133N9U3_Fusobacterium_gonidiaformans	lesions	yes	0.854	130	0.226	0.025	0.070
UniRef90_A0A0B4EP98_Fusobacterium_gonidiaformans	lesions	yes	0.886	130	0.233	0.025	0.070
UniRef90_A0A2P1RLY9_Fusobacterium_gonidiaformans	lesions	yes	0.994	130	0.226	0.025	0.070
UniRef90_Q5F5T4_Neisseria_sicca	lesions	yes	0.751	130	0.241	0.025	0.070
UniRef90_E4ZEH5_Haemophilus_parainfluenzae	lesions	yes	0.841	130	0.293	0.025	0.070
UniRef90_E5BE55_Fusobacterium_gonidiaformans	lesions	yes	0.875	130	0.241	0.025	0.070
UniRef90_A0A3E4SHN4_Prevotella_copri	lesions	yes	0.631	130	0.248	0.025	0.070
UniRef90_E5BHD1_Fusobacterium_gonidiaformans	lesions	yes	1.210	130	0.226	0.025	0.071
UniRef90_A0A133NC71_Fusobacterium_gonidiaformans	lesions	yes	1.442	130	0.233	0.025	0.071
UniRef90_E5WY79_Prevotella_bivia	lesions	yes	1.265	130	0.286	0.025	0.071
UniRef90_E5BH36_Fusobacterium_gonidiaformans	lesions	yes	1.347	130	0.263	0.025	0.071
UniRef90_H1D901_Fusobacterium_gonidiaformans	lesions	yes	1.163	130	0.226	0.025	0.071
UniRef90_I4ZBE6_Prevotella_bivia	lesions	yes	0.917	130	0.226	0.025	0.071
UniRef90_A0A162IXT3_Fusobacterium_gonidiaformans	lesions	yes	1.095	130	0.226	0.025	0.071

UniRef90_E5BFX9_Fusobacterium_gonidiaformans	lesions	yes	1.198	130	0.233	0.025	0.071
UniRef90_C6GFR2_Prevotella_bivia	lesions	yes	0.813	130	0.316	0.025	0.072
UniRef90_A0A133N879_Fusobacterium_gonidiaformans	lesions	yes	1.140	130	0.233	0.025	0.072
UniRef90_J4VWH0_Fusobacterium_gonidiaformans	lesions	yes	1.164	130	0.241	0.025	0.072
UniRef90_H1D9X4_Fusobacterium_gonidiaformans	lesions	yes	1.211	130	0.226	0.025	0.072
UniRef90_D1QNX9_Prevotella_bivia	lesions	yes	1.118	130	0.338	0.025	0.072
UniRef90_Q9JZ44_Neisseria_sicca	lesions	yes	0.763	130	0.241	0.025	0.072
UniRef90_J8SS39_Fusobacterium_gonidiaformans	lesions	yes	1.537	130	0.226	0.026	0.072
UniRef90_A0A017H339_Fusobacterium_gonidiaformans	lesions	yes	1.108	130	0.226	0.026	0.072
UniRef90_E5BEJ4_Fusobacterium_gonidiaformans	lesions	yes	1.267	130	0.226	0.026	0.073
UniRef90_E5BDU4_Fusobacterium_gonidiaformans	lesions	yes	1.209	130	0.226	0.026	0.073
UniRef90_A0A133NA71_Fusobacterium_gonidiaformans	lesions	yes	1.273	130	0.233	0.026	0.073
UniRef90_A0A145X6I1_Neisseria_flavescens	lesions	yes	0.867	130	0.226	0.026	0.073
UniRef90_A0A139PC68_Streptococcus_oralis	lesions	yes	0.806	130	0.248	0.026	0.073
UniRef90_A0A133NGJ8_Fusobacterium_gonidiaformans	lesions	yes	1.315	130	0.263	0.026	0.073
UniRef90_E5BEF5_Fusobacterium_gonidiaformans	lesions	yes	1.105	130	0.226	0.026	0.073
UniRef90_I4Z716_Prevotella_bivia	lesions	yes	1.021	130	0.226	0.026	0.073
UniRef90_E1KN21_Prevotella_bivia	lesions	yes	1.182	130	0.338	0.026	0.073
UniRef90_Q65V08_Haemophilus_parainfluenzae	lesions	yes	1.067	130	0.353	0.026	0.074
UniRef90_H1DC11_Fusobacterium_gonidiaformans	lesions	yes	1.142	130	0.226	0.026	0.074
UniRef90_E5BH10_Fusobacterium_gonidiaformans	lesions	yes	0.973	130	0.226	0.026	0.074
UniRef90_H1DAF6_Fusobacterium_gonidiaformans	lesions	yes	1.158	130	0.233	0.026	0.074
UniRef90_A0A137SSL8_Prevotella_bivia	lesions	yes	1.530	130	0.338	0.026	0.074
UniRef90_E5BDS1_Fusobacterium_gonidiaformans	lesions	yes	0.783	130	0.226	0.026	0.074
UniRef90_E5BDJ9_Fusobacterium_gonidiaformans	lesions	yes	1.046	130	0.226	0.026	0.074
UniRef90_H1DA90_Fusobacterium_gonidiaformans	lesions	yes	1.288	130	0.233	0.026	0.074
UniRef90_E5BGR9_Fusobacterium_gonidiaformans	lesions	yes	1.167	130	0.233	0.026	0.074
UniRef90_Q9S358_Neisseria_sicca	lesions	yes	0.765	130	0.241	0.026	0.075
UniRef90_Q47810_Streptococcus_mitis	lesions	yes	1.379	130	0.286	0.026	0.075
UniRef90_E5BGW7_Fusobacterium_gonidiaformans	lesions	yes	0.990	130	0.226	0.026	0.075
UniRef90_A0A161QVN3_Fusobacterium_gonidiaformans	lesions	yes	0.928	130	0.233	0.026	0.075
UniRef90_A0A133NEU0_Fusobacterium_gonidiaformans	lesions	yes	1.121	130	0.226	0.027	0.075
UniRef90_E5BG69_Fusobacterium_gonidiaformans	lesions	yes	0.934	130	0.226	0.027	0.075
UniRef90_E5BGT3_Fusobacterium_gonidiaformans	lesions	yes	1.244	130	0.233	0.027	0.075
UniRef90_E5BE23_Fusobacterium_gonidiaformans	lesions	yes	1.234	130	0.241	0.027	0.075
UniRef90_R6YP67_Prevotella_bivia	lesions	yes	1.017	130	0.278	0.027	0.075
UniRef90_A0A133NF92_Fusobacterium_gonidiaformans	lesions	yes	0.964	130	0.233	0.027	0.075
UniRef90_H1D5A2_Fusobacterium_gonidiaformans	lesions	yes	1.176	130	0.233	0.027	0.075
UniRef90_Q8RIG5_Fusobacterium_gonidiaformans	lesions	yes	0.898	130	0.233	0.027	0.075
UniRef90_A0A1R0EDS1_Haemophilus_parainfluenzae	lesions	yes	0.976	130	0.248	0.027	0.076
UniRef90_E5BF38_Fusobacterium_gonidiaformans	lesions	yes	0.863	130	0.226	0.027	0.076
UniRef90_E5BGV2_Fusobacterium_gonidiaformans	lesions	yes	0.854	130	0.226	0.027	0.076
UniRef90_H1DBC2_Fusobacterium_gonidiaformans	lesions	yes	1.243	130	0.226	0.027	0.076
UniRef90_A0A0T7ANX4_Prevotella_bivia	lesions	yes	1.306	130	0.278	0.027	0.076
UniRef90_E5BDU7_Fusobacterium_gonidiaformans	lesions	yes	1.089	130	0.233	0.027	0.076
UniRef90_E5BDI7_Fusobacterium_gonidiaformans	lesions	yes	0.917	130	0.233	0.027	0.077
UniRef90_E5BIN0_Fusobacterium_gonidiaformans	lesions	yes	1.280	130	0.241	0.027	0.077
UniRef90_A0A137STD7_Prevotella_bivia	lesions	yes	1.173	130	0.233	0.027	0.077
UniRef90_A0A133NA78_Fusobacterium_gonidiaformans	lesions	yes	1.117	130	0.226	0.028	0.077
UniRef90_A0A0B4E5L2_Fusobacterium_gonidiaformans	lesions	yes	1.086	130	0.226	0.028	0.077
UniRef90_E5BG68_Fusobacterium_gonidiaformans	lesions	yes	0.898	130	0.226	0.028	0.077
UniRef90_E5BE54_Fusobacterium_gonidiaformans	lesions	yes	1.162	130	0.233	0.028	0.077
UniRef90_E5BHE5_Fusobacterium_gonidiaformans	lesions	yes	1.504	130	0.256	0.028	0.078
UniRef90_A0A133NC73_Fusobacterium_gonidiaformans	lesions	yes	1.062	130	0.233	0.028	0.078
UniRef90_E5BGT7_Fusobacterium_gonidiaformans	lesions	yes	1.282	130	0.241	0.028	0.078
UniRef90_E5BEJ3_Fusobacterium_gonidiaformans	lesions	yes	1.145	130	0.233	0.028	0.078

UniRef90_A0A133NKH3_Fusobacterium_gonidiaformans	lesions	yes	1.215	130	0.233	0.028	0.078
UniRef90_E5BDK6_Fusobacterium_gonidiaformans	lesions	yes	0.758	130	0.226	0.028	0.078
UniRef90_P95379_Neisseria_flavescens	lesions	yes	0.818	130	0.263	0.028	0.078
UniRef90_Q04K52_Streptococcus_oralis	lesions	yes	1.033	130	0.263	0.028	0.078
UniRef90_E5BDH0_Fusobacterium_gonidiaformans	lesions	yes	1.323	130	0.233	0.028	0.078
UniRef90_J5VQN1_Fusobacterium_gonidiaformans	lesions	yes	0.858	130	0.226	0.028	0.079
UniRef90_E5BGQ3_Fusobacterium_gonidiaformans	lesions	yes	0.957	130	0.226	0.028	0.079
UniRef90_A0A096CIP2_Prevotella_bivia	lesions	yes	1.085	130	0.226	0.028	0.079
UniRef90_H1D840_Fusobacterium_gonidiaformans	lesions	yes	0.839	130	0.233	0.028	0.079
UniRef90_I4Z7V0_Prevotella_bivia	lesions	yes	1.194	130	0.226	0.028	0.079
UniRef90_A0A017H783_Fusobacterium_gonidiaformans	lesions	yes	0.938	130	0.226	0.028	0.079
UniRef90_E5BGR1_Fusobacterium_gonidiaformans	lesions	yes	1.096	130	0.226	0.028	0.079
UniRef90_E5BHT7_Fusobacterium_gonidiaformans	lesions	yes	1.226	130	0.233	0.028	0.080
UniRef90_A0A133NB08_Fusobacterium_gonidiaformans	lesions	yes	1.321	130	0.233	0.028	0.080
UniRef90_E5BGJ3_Fusobacterium_gonidiaformans	lesions	yes	1.048	130	0.226	0.028	0.080
UniRef90_E5BGA3_Fusobacterium_gonidiaformans	lesions	yes	0.822	130	0.241	0.029	0.080
UniRef90_P45127_Haemophilus_parainfluenzae	lesions	yes	0.766	130	0.353	0.029	0.080
UniRef90_A0A173YA79_Coprococcus_comes	lesions	yes	0.677	130	0.241	0.029	0.080
UniRef90_A0A133NIU1_Fusobacterium_gonidiaformans	lesions	yes	1.087	130	0.233	0.029	0.080
UniRef90_H1D925_Fusobacterium_gonidiaformans	lesions	yes	1.415	130	0.233	0.029	0.081
UniRef90_A0A133ZPE5_Fusobacterium_gonidiaformans	lesions	yes	1.448	130	0.293	0.029	0.081
UniRef90_E5BGU9_Fusobacterium_gonidiaformans	lesions	yes	0.889	130	0.226	0.029	0.081
UniRef90_J6GS25_Fusobacterium_gonidiaformans	lesions	yes	1.201	130	0.233	0.029	0.081
UniRef90_H1DA92_Fusobacterium_gonidiaformans	lesions	yes	1.248	130	0.248	0.029	0.081
UniRef90_E5BDW8_Fusobacterium_gonidiaformans	lesions	yes	1.062	130	0.226	0.029	0.081
UniRef90_E5BE47_Fusobacterium_gonidiaformans	lesions	yes	1.220	130	0.233	0.029	0.081
UniRef90_H1D806_Fusobacterium_gonidiaformans	lesions	yes	1.168	130	0.226	0.029	0.081
UniRef90_A0A137T0C8_Prevotella_bivia	lesions	yes	1.006	130	0.241	0.029	0.081
UniRef90_F9EV04_Neisseria_sicca	lesions	yes	0.720	130	0.248	0.029	0.081
UniRef90_E5BGX5_Fusobacterium_gonidiaformans	lesions	yes	1.333	130	0.226	0.029	0.081
UniRef90_H1D685_Fusobacterium_gonidiaformans	lesions	yes	1.348	130	0.248	0.029	0.081
UniRef90_E5BH55_Fusobacterium_gonidiaformans	lesions	yes	1.431	130	0.226	0.029	0.081
UniRef90_A0A017H329_Fusobacterium_gonidiaformans	lesions	yes	0.886	130	0.233	0.029	0.082
UniRef90_UPI000D11C76C_Fusobacterium_gonidiaformans	lesions	yes	1.411	130	0.271	0.029	0.082
UniRef90_H1D9K8_Fusobacterium_gonidiaformans	lesions	yes	0.934	130	0.233	0.029	0.082
UniRef90_H1D7Z7_Fusobacterium_gonidiaformans	lesions	yes	1.212	130	0.233	0.029	0.082
UniRef90_A0A0T9GWU8_Streptococcus_sanguinis	lesions	yes	0.955	130	0.278	0.029	0.082
UniRef90_H1D8F6_Fusobacterium_gonidiaformans	lesions	yes	0.863	130	0.226	0.029	0.082
UniRef90_E5BDM5_Fusobacterium_gonidiaformans	lesions	yes	1.207	130	0.226	0.029	0.082
UniRef90_E5BH52_Fusobacterium_gonidiaformans	lesions	yes	1.228	130	0.226	0.029	0.082
UniRef90_K9E6V7_Prevotella_bivia	lesions	yes	1.158	130	0.316	0.029	0.082
UniRef90_H1D7W6_Fusobacterium_gonidiaformans	lesions	yes	1.108	130	0.226	0.029	0.082
UniRef90_E5BH47_Fusobacterium_gonidiaformans	lesions	yes	0.897	130	0.233	0.029	0.082
UniRef90_R6XXN4_Prevotella_bivia	lesions	yes	0.792	130	0.323	0.030	0.083
UniRef90_E5BGU0_Fusobacterium_gonidiaformans	lesions	yes	1.212	130	0.226	0.030	0.083
UniRef90_E5BE14_Fusobacterium_gonidiaformans	lesions	yes	1.007	130	0.233	0.030	0.083
UniRef90_G1VEQ7_Prevotella_bivia	lesions	yes	1.115	130	0.293	0.030	0.083
UniRef90_A0A133NKD5_Fusobacterium_gonidiaformans	lesions	yes	1.261	130	0.226	0.030	0.083
UniRef90_P37247_Prevotella_bivia	lesions	yes	0.930	130	0.376	0.030	0.083
UniRef90_A0A133NEM0_Fusobacterium_gonidiaformans	lesions	yes	1.078	130	0.226	0.030	0.083
UniRef90_E5BGJ8_Fusobacterium_gonidiaformans	lesions	yes	1.157	130	0.233	0.030	0.083
UniRef90_A0A133NFH3_Fusobacterium_gonidiaformans	lesions	yes	0.883	130	0.233	0.030	0.083
UniRef90_A0A064ACK2_Fusobacterium_gonidiaformans	lesions	yes	1.087	130	0.233	0.030	0.083
UniRef90_E5BEB2_Fusobacterium_gonidiaformans	lesions	yes	0.827	130	0.233	0.030	0.083
UniRef90_X5F517_Neisseria_sicca	lesions	yes	0.822	130	0.233	0.030	0.083
UniRef90_A0A133NA08_Fusobacterium_gonidiaformans	lesions	yes	1.130	130	0.233	0.030	0.084

UniRef90_A0A0E2V5R7_Fusobacterium_gonidiaformans	lesions	yes	1.103	130	0.226	0.030	0.084
UniRef90_A0A133NLM8_Fusobacterium_gonidiaformans	lesions	yes	0.886	130	0.233	0.030	0.084
UniRef90_E5BEE2_Fusobacterium_gonidiaformans	lesions	yes	0.808	130	0.233	0.030	0.084
UniRef90_E5UT96_Prevotella_copri	lesions	yes	0.809	130	0.248	0.030	0.084
UniRef90_E5BH89_Fusobacterium_gonidiaformans	lesions	yes	0.880	130	0.233	0.030	0.084
UniRef90_Q3K3Q0_Streptococcus_mitis	lesions	yes	1.353	130	0.331	0.030	0.085
UniRef90_A0A133N9R8_Fusobacterium_gonidiaformans	lesions	yes	1.142	130	0.263	0.030	0.085
UniRef90_H1D489_Fusobacterium_gonidiaformans	lesions	yes	0.824	130	0.241	0.030	0.085
UniRef90_E5BG39_Fusobacterium_gonidiaformans	lesions	yes	1.062	130	0.233	0.030	0.085
UniRef90_Q04HZ9_Streptococcus_oralis	lesions	yes	0.655	130	0.256	0.030	0.085
UniRef90_A0A017H7G9_Fusobacterium_gonidiaformans	lesions	yes	1.246	130	0.226	0.030	0.085
UniRef90_E5BF95_Fusobacterium_gonidiaformans	lesions	yes	1.165	130	0.233	0.030	0.085
UniRef90_A0A062WP34_Streptococcus_mitis	lesions	yes	1.091	130	0.429	0.030	0.085
UniRef90_C3WFW9_Fusobacterium_gonidiaformans	lesions	yes	0.917	130	0.226	0.030	0.085
UniRef90_E5BDI9_Fusobacterium_gonidiaformans	lesions	yes	0.902	130	0.241	0.031	0.085
UniRef90_P0DB88_Streptococcus_sanguinis	lesions	yes	0.761	130	0.263	0.031	0.086
UniRef90_H1DAB0_Fusobacterium_gonidiaformans	lesions	yes	0.854	130	0.233	0.031	0.086
UniRef90_E5BG11_Fusobacterium_gonidiaformans	lesions	yes	0.841	130	0.226	0.031	0.086
UniRef90_E5BH58_Fusobacterium_gonidiaformans	lesions	yes	1.177	130	0.263	0.031	0.086
UniRef90_H1DBF6_Fusobacterium_gonidiaformans	lesions	yes	0.762	130	0.226	0.031	0.086
UniRef90_E5BEK6_Fusobacterium_gonidiaformans	lesions	yes	0.855	130	0.226	0.031	0.086
UniRef90_A0A162J162_Fusobacterium_gonidiaformans	lesions	yes	1.220	130	0.233	0.031	0.087
UniRef90_A0A137SRB8_Prevotella_bivia	lesions	yes	1.210	130	0.301	0.031	0.087
UniRef90_A0A133YAZ9_Prevotella_bivia	lesions	yes	1.322	130	0.308	0.031	0.087
UniRef90_F9DH44_Prevotella_bivia	lesions	yes	1.151	130	0.293	0.031	0.087
UniRef90_A0A137T1C0_Prevotella_bivia	lesions	yes	1.256	130	0.233	0.031	0.087
UniRef90_H1D4Z0_Fusobacterium_gonidiaformans	lesions	yes	1.185	130	0.233	0.031	0.087
UniRef90_A0A133NCX7_Fusobacterium_gonidiaformans	lesions	yes	1.173	130	0.233	0.031	0.087
UniRef90_I4Z7P1_Prevotella_bivia	lesions	yes	1.248	130	0.316	0.031	0.087
UniRef90_E5BDP1_Fusobacterium_gonidiaformans	lesions	yes	0.854	130	0.226	0.031	0.087
UniRef90_A0A017H4J1_Fusobacterium_gonidiaformans	lesions	yes	0.881	130	0.226	0.031	0.087
UniRef90_A0A137T0X5_Prevotella_bivia	lesions	yes	0.637	130	0.241	0.031	0.087
UniRef90_G1WD84_Prevotella_bivia	lesions	yes	1.002	130	0.293	0.031	0.087
UniRef90_H1D9Y6_Fusobacterium_gonidiaformans	lesions	yes	0.787	130	0.241	0.031	0.087
UniRef90_A0A133NC81_Fusobacterium_gonidiaformans	lesions	yes	1.151	130	0.226	0.031	0.088
UniRef90_H1DBZ5_Fusobacterium_gonidiaformans	lesions	yes	1.128	130	0.263	0.031	0.088
UniRef90_E5BHR6_Fusobacterium_gonidiaformans	lesions	yes	1.084	130	0.241	0.032	0.088
UniRef90_A0A133NLM3_Fusobacterium_gonidiaformans	lesions	yes	1.089	130	0.226	0.032	0.088
UniRef90_E5BGB7_Fusobacterium_gonidiaformans	lesions	yes	0.863	130	0.233	0.032	0.088
UniRef90_A0A396DV75_Prevotella_bivia	lesions	yes	1.134	130	0.331	0.032	0.088
UniRef90_S0F6D4_Prevotella_bivia	lesions	yes	0.969	130	0.271	0.032	0.088
UniRef90_H1D430_Fusobacterium_gonidiaformans	lesions	yes	1.436	130	0.226	0.032	0.088
UniRef90_E5BH07_Fusobacterium_gonidiaformans	lesions	yes	0.858	130	0.226	0.032	0.089
UniRef90_J5TQA9_Fusobacterium_gonidiaformans	lesions	yes	0.840	130	0.226	0.032	0.089
UniRef90_Q8DS23_Streptococcus_oralis	lesions	yes	1.161	130	0.331	0.032	0.089
UniRef90_E5BHB1_Fusobacterium_gonidiaformans	lesions	yes	1.185	130	0.226	0.032	0.090
UniRef90_A0A133Y188_Prevotella_bivia	lesions	yes	0.695	130	0.398	0.032	0.090
UniRef90_E5BI67_Fusobacterium_gonidiaformans	lesions	yes	1.260	130	0.233	0.032	0.090
UniRef90_E5BHW2_Fusobacterium_gonidiaformans	lesions	yes	1.053	130	0.226	0.032	0.090
UniRef90_E5BFR0_Fusobacterium_gonidiaformans	lesions	yes	1.371	130	0.241	0.032	0.090
UniRef90_A0A096AEJ9_Prevotella_bivia	lesions	yes	1.177	130	0.233	0.032	0.090
UniRef90_J8W633_Fusobacterium_gonidiaformans	lesions	yes	1.202	130	0.248	0.032	0.090
UniRef90_A0A133NJX0_Fusobacterium_gonidiaformans	lesions	yes	0.851	130	0.226	0.032	0.090
UniRef90_A0A3E5DLS5_Prevotella_bivia	lesions	yes	1.150	130	0.308	0.033	0.090
UniRef90_E5BGU1_Fusobacterium_gonidiaformans	lesions	yes	1.068	130	0.226	0.033	0.091
UniRef90_E5BGC4_Fusobacterium_gonidiaformans	lesions	yes	0.897	130	0.226	0.033	0.091

UniRef90_A0A133NE17_Fusobacterium_gonidiaformans	lesions	yes	0.885	130	0.241	0.033	0.091
UniRef90_E5BGS9_Fusobacterium_gonidiaformans	lesions	yes	1.233	130	0.233	0.033	0.091
UniRef90_D2ZSG6_Neisseria_flavescens	lesions	yes	0.799	130	0.271	0.033	0.091
UniRef90_H1DC23_Fusobacterium_gonidiaformans	lesions	yes	1.372	130	0.271	0.033	0.092
UniRef90_E5BDM0_Fusobacterium_gonidiaformans	lesions	yes	1.203	130	0.256	0.033	0.092
UniRef90_E5BHF4_Fusobacterium_gonidiaformans	lesions	yes	1.087	130	0.248	0.033	0.092
UniRef90_P43799_Haemophilus_parainfluenzae	lesions	yes	1.158	130	0.376	0.033	0.092
UniRef90_E5BGJ1_Fusobacterium_gonidiaformans	lesions	yes	1.168	130	0.226	0.033	0.092
UniRef90_A0A154U400_Neisseria_sicca	lesions	yes	0.655	130	0.256	0.033	0.092
UniRef90_A0A3E5E455_Prevotella_bivia	lesions	yes	1.061	130	0.248	0.033	0.092
UniRef90_A0A0J0YSM7_Neisseria_sicca	lesions	yes	0.714	130	0.226	0.033	0.092
UniRef90_E5BG89_Fusobacterium_gonidiaformans	lesions	yes	0.923	130	0.233	0.033	0.092
UniRef90_J6GZC8_Fusobacterium_gonidiaformans	lesions	yes	1.110	130	0.226	0.033	0.093
UniRef90_H1D562_Fusobacterium_gonidiaformans	lesions	yes	1.254	130	0.256	0.033	0.093
UniRef90_A0A229I3E2_Prevotella_copri	lesions	yes	-0.699	130	0.286	0.033	0.093
UniRef90_E5BGW8_Fusobacterium_gonidiaformans	lesions	yes	1.350	130	0.233	0.034	0.093
UniRef90_P0A3Q8_Streptococcus_oralis	lesions	yes	1.116	130	0.346	0.034	0.093
UniRef90_F9EST7_Neisseria_sicca	lesions	yes	0.750	130	0.233	0.034	0.094
UniRef90_A0A133NKS1_Fusobacterium_gonidiaformans	lesions	yes	1.212	130	0.241	0.034	0.094
UniRef90_E5BIM9_Fusobacterium_gonidiaformans	lesions	yes	1.176	130	0.233	0.034	0.094
UniRef90_A0A096AFQ5_Prevotella_bivia	lesions	yes	1.159	130	0.248	0.034	0.094
UniRef90_H1D989_Fusobacterium_gonidiaformans	lesions	yes	0.803	130	0.233	0.034	0.094
UniRef90_E5BEA5_Fusobacterium_gonidiaformans	lesions	yes	1.119	130	0.226	0.034	0.094
UniRef90_E5BFU7_Fusobacterium_gonidiaformans	lesions	yes	0.900	130	0.226	0.034	0.094
UniRef90_E5BDD6_Fusobacterium_gonidiaformans	lesions	yes	0.753	130	0.226	0.034	0.094
UniRef90_E1KPP4_Prevotella_bivia	lesions	yes	1.250	130	0.256	0.034	0.094
UniRef90_A0A3E4SIC7_Prevotella_copri	lesions	yes	0.756	130	0.226	0.034	0.094
UniRef90_E5BIA6_Fusobacterium_gonidiaformans	lesions	yes	0.777	130	0.233	0.034	0.095
UniRef90_H1DA14_Fusobacterium_gonidiaformans	lesions	yes	1.124	130	0.226	0.034	0.095
UniRef90_E5BGT8_Fusobacterium_gonidiaformans	lesions	yes	1.151	130	0.226	0.034	0.095
UniRef90_P44390_Neisseria_mucosa	lesions	yes	0.718	130	0.226	0.034	0.095
UniRef90_U2KQU1_Dorea_formicigenerans	lesions	yes	0.778	130	0.263	0.034	0.095
UniRef90_W6NZG1_Prevotella_bivia	lesions	yes	1.132	130	0.331	0.034	0.095
UniRef90_E5BGI2_Fusobacterium_gonidiaformans	lesions	yes	0.838	130	0.233	0.034	0.095
UniRef90_A0A064AKW2_Fusobacterium_gonidiaformans	lesions	yes	1.021	130	0.256	0.034	0.095
UniRef90_E5BHJ5_Fusobacterium_gonidiaformans	lesions	yes	0.844	130	0.226	0.035	0.096
UniRef90_A0A134BKG4_Prevotella_bivia	lesions	yes	1.064	130	0.226	0.035	0.096
UniRef90_A0A137SVJ6_Prevotella_bivia	lesions	yes	1.116	130	0.226	0.035	0.096
UniRef90_H1D3Q5_Fusobacterium_gonidiaformans	lesions	yes	0.885	130	0.241	0.035	0.096
UniRef90_E5BFX6_Fusobacterium_gonidiaformans	lesions	yes	1.140	130	0.226	0.035	0.096
UniRef90_E5BGD2_Fusobacterium_gonidiaformans	lesions	yes	1.182	130	0.256	0.035	0.096
UniRef90_G6AW31_Prevotella_stercorea	lesions	yes	0.884	130	0.241	0.035	0.097
UniRef90_E5BE26_Fusobacterium_gonidiaformans	lesions	yes	1.133	130	0.226	0.035	0.097
UniRef90_E5BHG1_Fusobacterium_gonidiaformans	lesions	yes	1.121	130	0.263	0.035	0.097
UniRef90_P66539_Neisseria_sicca	lesions	yes	0.656	130	0.226	0.035	0.097
UniRef90_A0A137T0Q9_Prevotella_bivia	lesions	yes	0.412	130	0.241	0.035	0.097
UniRef90_E5UJV0_Neisseria_sicca	lesions	yes	0.633	130	0.226	0.035	0.097
UniRef90_A0A133NJQ1_Fusobacterium_gonidiaformans	lesions	yes	0.981	130	0.226	0.035	0.098
UniRef90_A0A174YBK6_Ruminococcus_torques	lesions	yes	-0.721	130	0.301	0.035	0.098
UniRef90_R6F6Y3_Prevotella_sp_CAG_520	lesions	yes	0.768	130	0.263	0.035	0.098
UniRef90_E5BDL6_Fusobacterium_gonidiaformans	lesions	yes	0.869	130	0.233	0.035	0.098
UniRef90_E5BF10_Fusobacterium_gonidiaformans	lesions	yes	1.015	130	0.241	0.035	0.098
UniRef90_H1DB17_Fusobacterium_gonidiaformans	lesions	yes	0.926	130	0.226	0.035	0.098
UniRef90_E5BI57_Fusobacterium_gonidiaformans	lesions	yes	0.889	130	0.226	0.035	0.098
UniRef90_P43822_Haemophilus_parainfluenzae	lesions	yes	0.774	130	0.346	0.036	0.098
UniRef90_H1DAZ9_Fusobacterium_gonidiaformans	lesions	yes	1.124	130	0.256	0.036	0.098

UniRef90_A0A096CL16_Prevotella_bivia	lesions	yes	1.039	130	0.226	0.036	0.098
UniRef90_E5BE12_Fusobacterium_gonidiaformans	lesions	yes	1.093	130	0.233	0.036	0.098
UniRef90_Q9K0M5_Neisseria_sicca	lesions	yes	0.623	130	0.226	0.036	0.098
UniRef90_A0A162J9A8_Fusobacterium_gonidiaformans	lesions	yes	1.125	130	0.226	0.036	0.099
UniRef90_G6AZK7_Prevotella_stercorea	lesions	yes	0.744	130	0.263	0.036	0.099
UniRef90_A0A137T1C5_Prevotella_bivia	lesions	yes	1.251	130	0.256	0.036	0.099
UniRef90_R6EVZ0_Prevotella_sp_CAG_520	lesions	yes	0.854	130	0.233	0.036	0.099
UniRef90_A0A139RMF9_Streptococcus_oralis	lesions	yes	1.044	130	0.263	0.036	0.099
UniRef90_A0A3A5CDW0_Neisseria_sicca	lesions	yes	0.613	130	0.226	0.036	0.099
UniRef90_A0A3E4MVM5_Prevotella_sp_885	lesions	yes	0.631	130	0.248	0.036	0.099
UniRef90_E5BEA2_Fusobacterium_gonidiaformans	lesions	yes	1.122	130	0.256	0.036	0.100
UniRef90_D1W0P9_Prevotella_stercorea	lesions	yes	0.678	130	0.248	0.036	0.100
UniRef90_A0A173YA79_Roseburia_inulinivorans	lesions	yes	0.790	130	0.256	0.036	0.100
UniRef90_Q5F5S8_Neisseria_sicca	lesions	yes	0.798	130	0.248	0.036	0.100
UniRef90_C7XCI1_Prevotella_bivia	lesions	yes	1.035	130	0.301	0.036	0.100
UniRef90_P44803_Neisseria_flavescens	lesions	yes	0.730	130	0.263	0.036	0.100
UniRef90_P66854_Streptococcus_oralis	lesions	yes	1.036	130	0.353	0.036	0.100
UniRef90_Q9JYQ8_Neisseria_sicca	lesions	yes	0.560	130	0.233	0.037	0.101
UniRef90_E5BDW6_Fusobacterium_gonidiaformans	lesions	yes	1.245	130	0.241	0.037	0.101
UniRef90_E5BGY6_Fusobacterium_gonidiaformans	lesions	yes	0.859	130	0.226	0.037	0.101
UniRef90_E5BF42_Fusobacterium_gonidiaformans	lesions	yes	1.018	130	0.226	0.037	0.101
UniRef90_A0A096C242_Prevotella_bivia	lesions	yes	0.839	130	0.248	0.037	0.101
UniRef90_D9RWG1_Prevotella_bivia	lesions	yes	0.835	130	0.233	0.037	0.102
UniRef90_A0A133NAY9_Fusobacterium_gonidiaformans	lesions	yes	1.021	130	0.233	0.037	0.102
UniRef90_I4ZAU8_Prevotella_bivia	lesions	yes	1.019	130	0.241	0.037	0.102
UniRef90_E4MAW9_Prevotella_bivia	lesions	yes	1.001	130	0.331	0.037	0.102
UniRef90_A0A3E4SC61_Prevotella_sp_885	lesions	yes	0.758	130	0.233	0.037	0.102
UniRef90_G1VFX1_Prevotella_bivia	lesions	yes	0.938	130	0.338	0.037	0.102
UniRef90_J5W2G7_Fusobacterium_gonidiaformans	lesions	yes	1.085	130	0.263	0.037	0.103
UniRef90_A0A2X1UHB2_Neisseria_flavescens	lesions	yes	0.897	130	0.256	0.037	0.103
UniRef90_I4Z854_Prevotella_bivia	lesions	yes	1.403	130	0.331	0.037	0.103
UniRef90_E5BG93_Fusobacterium_gonidiaformans	lesions	yes	1.138	130	0.226	0.038	0.103
UniRef90_Q97TA6_Streptococcus_oralis	lesions	yes	1.157	130	0.398	0.038	0.103
UniRef90_A0A3E4SCK7_Prevotella_copri	lesions	yes	1.074	130	0.271	0.038	0.104
UniRef90_I4ZA31_Prevotella_bivia	lesions	yes	0.814	130	0.233	0.038	0.104
UniRef90_A0A017H6N6_Fusobacterium_gonidiaformans	lesions	yes	1.214	130	0.226	0.038	0.104
UniRef90_E5BEK8_Fusobacterium_gonidiaformans	lesions	yes	0.790	130	0.233	0.038	0.104
UniRef90_A0A374N425_Prevotella_bivia	lesions	yes	0.841	130	0.391	0.038	0.104
UniRef90_I4Z6S2_Prevotella_bivia	lesions	yes	0.944	130	0.226	0.038	0.104
UniRef90_A0A2P1RIU7_Fusobacterium_gonidiaformans	lesions	yes	1.070	130	0.226	0.038	0.104
UniRef90_E5BEL3_Fusobacterium_gonidiaformans	lesions	yes	0.776	130	0.233	0.038	0.104
UniRef90_Q8RIG8_Fusobacterium_gonidiaformans	lesions	yes	1.317	130	0.263	0.038	0.105
UniRef90_Q9XBG8_Prevotella_bivia	lesions	yes	0.890	130	0.398	0.038	0.105
UniRef90_A0A133NFH9_Fusobacterium_gonidiaformans	lesions	yes	1.325	130	0.263	0.038	0.105
UniRef90_Q8RHI2_Fusobacterium_gonidiaformans	lesions	yes	1.135	130	0.233	0.038	0.105
UniRef90_F0H553_Prevotella_bivia	lesions	yes	0.876	130	0.256	0.038	0.105
UniRef90_H1DB16_Fusobacterium_gonidiaformans	lesions	yes	0.889	130	0.233	0.038	0.106
UniRef90_E5BG42_Fusobacterium_gonidiaformans	lesions	yes	1.218	130	0.241	0.038	0.106
UniRef90_E5BGC9_Fusobacterium_gonidiaformans	lesions	yes	0.821	130	0.233	0.039	0.106
UniRef90_E5BGA6_Fusobacterium_gonidiaformans	lesions	yes	1.201	130	0.226	0.039	0.106
UniRef90_A0A0E2V6S2_Fusobacterium_gonidiaformans	lesions	yes	0.849	130	0.226	0.039	0.106
UniRef90_A0A2R4ATN8_Neisseria_sicca	lesions	yes	0.654	130	0.226	0.039	0.106
UniRef90_C3R2Z0_Prevotella_bivia	lesions	yes	0.774	130	0.316	0.039	0.107
UniRef90_U2K6B1_Prevotella_bivia	lesions	yes	1.373	130	0.361	0.039	0.107
UniRef90_E5BH02_Fusobacterium_gonidiaformans	lesions	yes	0.890	130	0.226	0.039	0.107
UniRef90_J4VVL6_Fusobacterium_gonidiaformans	lesions	yes	0.808	130	0.226	0.039	0.107

UniRef90_U2II21_Prevotella_bivia	lesions	yes	1.135	130	0.301	0.039	0.107
UniRef90_E5BF93_Fusobacterium_gonidiaformans	lesions	yes	0.747	130	0.226	0.039	0.107
UniRef90_I4Z6Q1_Prevotella_bivia	lesions	yes	0.811	130	0.241	0.039	0.107
UniRef90_E5BFV7_Fusobacterium_gonidiaformans	lesions	yes	0.846	130	0.241	0.039	0.108
UniRef90_A0A064BX67_Streptococcus_oralis	lesions	yes	0.710	130	0.271	0.039	0.108
UniRef90_E5BGH1_Fusobacterium_gonidiaformans	lesions	yes	0.864	130	0.226	0.040	0.108
UniRef90_E5BFX5_Fusobacterium_gonidiaformans	lesions	yes	0.963	130	0.233	0.040	0.109
UniRef90_B0UX32_Haemophilus_parainfluenzae	lesions	yes	1.340	130	0.256	0.040	0.109
UniRef90_D3A1Q6_Neisseria_flavescens	lesions	yes	0.914	130	0.226	0.040	0.109
UniRef90_Q8RE42_Fusobacterium_gonidiaformans	lesions	yes	1.008	130	0.248	0.040	0.109
UniRef90_A0A137T063_Prevotella_bivia	lesions	yes	0.735	130	0.226	0.040	0.110
UniRef90_E6KJT7_Streptococcus_oralis	lesions	yes	1.066	130	0.346	0.040	0.110
UniRef90_I2NX17_Neisseria_sicca	lesions	yes	0.745	130	0.241	0.040	0.110
UniRef90_E5WU41_Prevotella_bivia	lesions	yes	0.680	130	0.368	0.040	0.110
UniRef90_W0ERK2_Prevotella_bivia	lesions	yes	1.069	130	0.338	0.040	0.110
UniRef90_E5BHF2_Fusobacterium_gonidiaformans	lesions	yes	0.773	130	0.226	0.040	0.110
UniRef90_E5BGV3_Fusobacterium_gonidiaformans	lesions	yes	0.921	130	0.233	0.040	0.110
UniRef90_A0A017H736_Fusobacterium_gonidiaformans	lesions	yes	0.765	130	0.226	0.040	0.110
UniRef90_E1KNK1_Prevotella_bivia	lesions	yes	0.910	130	0.241	0.040	0.110
UniRef90_E5BDV4_Fusobacterium_gonidiaformans	lesions	yes	1.101	130	0.241	0.040	0.110
UniRef90_A0A3D1H1R8_Prevotella_sp_885	lesions	yes	0.512	130	0.256	0.041	0.111
UniRef90_A0A173S9W5_Coprococcus_comes	lesions	yes	-0.713	130	0.248	0.041	0.111
UniRef90_R5C7B6_Prevotella_stercorea	lesions	yes	0.860	130	0.241	0.041	0.111
UniRef90_D7NG96_Prevotella_bivia	lesions	yes	0.630	130	0.391	0.041	0.111
UniRef90_P0A4M9_Streptococcus_oralis	lesions	yes	1.254	130	0.338	0.041	0.112
UniRef90_L1NWJ9_Neisseria_sicca	lesions	yes	0.606	130	0.241	0.041	0.112
UniRef90_E5BH61_Fusobacterium_gonidiaformans	lesions	yes	0.903	130	0.241	0.041	0.112
UniRef90_P56990_Neisseria_sicca	lesions	yes	0.638	130	0.233	0.041	0.112
UniRef90_A0A0D0HAG6_Prevotella_sp_CAG_520	lesions	yes	0.837	130	0.248	0.041	0.112
UniRef90_E5BEK3_Fusobacterium_gonidiaformans	lesions	yes	0.836	130	0.233	0.041	0.113
UniRef90_D5EUS1_Prevotella_bivia	lesions	yes	1.305	130	0.316	0.041	0.113
UniRef90_A0A0B4E8T3_Fusobacterium_gonidiaformans	lesions	yes	1.044	130	0.226	0.041	0.113
UniRef90_J6GS20_Fusobacterium_gonidiaformans	lesions	yes	1.286	130	0.226	0.041	0.113
UniRef90_A0A133NAV9_Fusobacterium_gonidiaformans	lesions	yes	1.101	130	0.226	0.041	0.113
UniRef90_E5BFA7_Fusobacterium_gonidiaformans	lesions	yes	0.873	130	0.226	0.041	0.113
UniRef90_E5BEI9_Fusobacterium_gonidiaformans	lesions	yes	1.127	130	0.226	0.042	0.113
UniRef90_K6A0H4_Prevotella_bivia	lesions	yes	1.211	130	0.301	0.042	0.113
UniRef90_E5BH74_Fusobacterium_gonidiaformans	lesions	yes	1.036	130	0.256	0.042	0.114
UniRef90_D9RWP7_Prevotella_bivia	lesions	yes	1.388	130	0.278	0.042	0.114
UniRef90_D7N4P3_Neisseria_sicca	lesions	yes	0.623	130	0.233	0.042	0.114
UniRef90_E1W4F8_Haemophilus_parainfluenzae	lesions	yes	0.844	130	0.263	0.042	0.114
UniRef90_Q9K0M4_Neisseria_sicca	lesions	yes	0.640	130	0.256	0.042	0.114
UniRef90_F0H8Q7_Prevotella_sp_CAG_520	lesions	yes	0.526	130	0.248	0.042	0.114
UniRef90_A0A133NGZ6_Fusobacterium_gonidiaformans	lesions	yes	1.056	130	0.226	0.042	0.114
UniRef90_D0WCC3_Neisseria_sicca	lesions	yes	0.698	130	0.256	0.042	0.115
UniRef90_H1D4N0_Fusobacterium_gonidiaformans	lesions	yes	1.016	130	0.256	0.042	0.115
UniRef90_E5BI59_Fusobacterium_gonidiaformans	lesions	yes	1.096	130	0.256	0.042	0.115
UniRef90_A7VCS4_Eubacterium_rectale	lesions	yes	1.018	130	0.241	0.042	0.115
UniRef90_Q3K2D0_Streptococcus_mitis	lesions	yes	1.103	130	0.376	0.042	0.115
UniRef90_E4ZEH5_Neisseria_sicca	lesions	yes	0.661	130	0.271	0.042	0.116
UniRef90_A0A0X8UU30_Streptococcus_mitis	lesions	yes	0.820	130	0.233	0.043	0.116
UniRef90_E5BHG9_Fusobacterium_gonidiaformans	lesions	yes	0.997	130	0.226	0.043	0.116
UniRef90_H1DC87_Fusobacterium_gonidiaformans	lesions	yes	1.162	130	0.241	0.043	0.117
UniRef90_A0A133NFI0_Fusobacterium_gonidiaformans	lesions	yes	0.759	130	0.241	0.043	0.117
UniRef90_H1D6P7_Fusobacterium_gonidiaformans	lesions	yes	0.981	130	0.233	0.043	0.117
UniRef90_C0DTL8_Neisseria_flavescens	lesions	yes	0.667	130	0.226	0.043	0.117

UniRef90_A0A134BGT4_Prevotella_bivia	lesions	yes	0.945	130	0.226	0.043	0.117
UniRef90_I4ZAN1_Prevotella_bivia	lesions	yes	0.983	130	0.256	0.043	0.117
UniRef90_E5BHN0_Fusobacterium_gonidiaformans	lesions	yes	1.075	130	0.226	0.043	0.118
UniRef90_D2ZSG6_Neisseria_sicca	lesions	yes	0.754	130	0.256	0.043	0.118
UniRef90_G6AG08_Prevotella_bivia	lesions	yes	1.246	130	0.271	0.043	0.118
UniRef90_I4Z6Y8_Prevotella_bivia	lesions	yes	1.185	130	0.323	0.043	0.118
UniRef90_A0A0F2DYF7_Streptococcus_oralis	lesions	yes	0.725	130	0.271	0.044	0.119
UniRef90_H1DAW7_Fusobacterium_gonidiaformans	lesions	yes	1.023	130	0.226	0.044	0.119
UniRef90_F0F606_Prevotella_bivia	lesions	yes	0.875	130	0.256	0.044	0.119
UniRef90_E5BF94_Fusobacterium_gonidiaformans	lesions	yes	1.331	130	0.226	0.044	0.119
UniRef90_E5BGK4_Fusobacterium_gonidiaformans	lesions	yes	0.803	130	0.233	0.044	0.119
UniRef90_E5BGF7_Fusobacterium_gonidiaformans	lesions	yes	1.208	130	0.241	0.044	0.119
UniRef90_R7H0N4_Prevotella_stercorea	lesions	yes	0.991	130	0.241	0.044	0.119
UniRef90_Q97Q16_Streptococcus_mitis	lesions	yes	1.132	130	0.391	0.044	0.120
UniRef90_R7H1W0_Prevotella_sp_885	lesions	yes	0.740	130	0.263	0.044	0.120
UniRef90_Q04I78_Streptococcus_oralis	lesions	yes	1.279	130	0.429	0.044	0.120
UniRef90_Q5F623_Neisseria_sicca	lesions	yes	0.541	130	0.226	0.044	0.120
UniRef90_A0A0D9LZZ7_Neisseria_sicca	lesions	yes	0.547	130	0.233	0.044	0.120
UniRef90_Q4QMB4_Haemophilus_parainfluenzae	lesions	yes	1.036	130	0.271	0.044	0.120
UniRef90_E5BIH1_Fusobacterium_gonidiaformans	lesions	yes	0.660	130	0.233	0.044	0.121
UniRef90_Q9JY99_Neisseria_sicca	lesions	yes	0.646	130	0.226	0.044	0.121
UniRef90_H1D5B6_Fusobacterium_gonidiaformans	lesions	yes	0.891	130	0.241	0.045	0.121
UniRef90_E5BGD7_Fusobacterium_gonidiaformans	lesions	yes	0.963	130	0.233	0.045	0.122
UniRef90_G6AWH8_Prevotella_stercorea	lesions	yes	0.878	130	0.256	0.045	0.122
UniRef90_E5BFV5_Fusobacterium_gonidiaformans	lesions	yes	0.817	130	0.226	0.045	0.122
UniRef90_P45015_Haemophilus_parainfluenzae	lesions	yes	1.000	130	0.353	0.045	0.122
UniRef90_A0A162IL18_Fusobacterium_gonidiaformans	lesions	yes	1.026	130	0.233	0.045	0.122
UniRef90_A0A1B8QXA5_Haemophilus_parainfluenzae	lesions	yes	0.552	130	0.226	0.045	0.122
UniRef90_F0H4Y7_Prevotella_bivia	lesions	yes	1.181	130	0.316	0.045	0.122
UniRef90_R6F5A5_Prevotella_sp_CAG_520	lesions	yes	0.394	130	0.233	0.045	0.122
UniRef90_E5BDR2_Fusobacterium_gonidiaformans	lesions	yes	1.033	130	0.226	0.045	0.122
UniRef90_A0A3E4SCH5_Prevotella_copri	lesions	yes	1.016	130	0.226	0.045	0.122
UniRef90_A0A3E4SBR7_Prevotella_copri	lesions	yes	0.595	130	0.233	0.045	0.123
UniRef90_E1MAP3_Streptococcus_oralis	lesions	yes	0.744	130	0.301	0.045	0.123
UniRef90_G6AEN9_Prevotella_bivia	lesions	yes	0.685	130	0.226	0.045	0.123
UniRef90_P63384_Streptococcus_sanguinis	lesions	yes	0.832	130	0.248	0.045	0.123
UniRef90_E2PDT5_Neisseria_flavescens	lesions	yes	0.783	130	0.226	0.046	0.123
UniRef90_A0A0T8AUD9_Streptococcus_oralis	lesions	yes	1.212	130	0.286	0.046	0.124
UniRef90_Q04JN5_Streptococcus_mitis	lesions	yes	0.872	130	0.406	0.046	0.124
UniRef90_P44390_Haemophilus_parainfluenzae	lesions	yes	0.874	130	0.376	0.046	0.124
UniRef90_G6B0G1_Prevotella_sp_CAG_520	lesions	yes	0.786	130	0.248	0.046	0.124
UniRef90_I4Z7D5_Prevotella_bivia	lesions	yes	1.015	130	0.271	0.046	0.124
UniRef90_E5BDJ5_Fusobacterium_gonidiaformans	lesions	yes	1.141	130	0.226	0.046	0.124
UniRef90_D3LUR1_Fusobacterium_gonidiaformans	lesions	yes	1.007	130	0.293	0.046	0.124
UniRef90_A0A134BNA3_Prevotella_bivia	lesions	yes	0.790	130	0.226	0.046	0.124
UniRef90_H1D406_Fusobacterium_gonidiaformans	lesions	yes	0.674	130	0.226	0.046	0.124
UniRef90_J0UXN8_Streptococcus_oralis	lesions	yes	1.150	130	0.406	0.046	0.125
UniRef90_Q8RIM0_Fusobacterium_gonidiaformans	lesions	yes	0.808	130	0.226	0.046	0.125
UniRef90_A0A1B8T524_Haemophilus_parainfluenzae	lesions	yes	0.818	130	0.278	0.046	0.125
UniRef90_A0A062WP34_Streptococcus_oralis	lesions	yes	1.079	130	0.346	0.046	0.126
UniRef90_A0A096CHD4_Prevotella_bivia	lesions	yes	1.071	130	0.226	0.046	0.126
UniRef90_E5BHR5_Fusobacterium_gonidiaformans	lesions	yes	0.987	130	0.226	0.047	0.126
UniRef90_E5BDU3_Fusobacterium_gonidiaformans	lesions	yes	0.724	130	0.226	0.047	0.126
UniRef90_E5BID0_Fusobacterium_gonidiaformans	lesions	yes	1.021	130	0.256	0.047	0.126
UniRef90_A0A096CJE9_Prevotella_bivia	lesions	yes	1.029	130	0.248	0.047	0.127
UniRef90_A0A081QAX6_Streptococcus_oralis	lesions	yes	1.076	130	0.263	0.047	0.127

UniRef90_E5BHU2_Fusobacterium_gonidiaformans	lesions	yes	0.835	130	0.226	0.047	0.127
UniRef90_I4ZAY3_Prevotella_bivia	lesions	yes	1.011	130	0.241	0.047	0.127
UniRef90_I4Z8P5_Prevotella_bivia	lesions	yes	0.918	130	0.226	0.047	0.127
UniRef90_A0A1X1IA35_Streptococcus_sanguinis	lesions	yes	0.569	130	0.256	0.047	0.128
UniRef90_E5BGY4_Fusobacterium_gonidiaformans	lesions	yes	1.004	130	0.226	0.047	0.128
UniRef90_R9HWQ8_Prevotella_bivia	lesions	yes	0.736	130	0.263	0.048	0.128
UniRef90_A9M490_Neisseria_sicca	lesions	yes	0.689	130	0.256	0.048	0.129
UniRef90_Q9JZ07_Neisseria_sicca	lesions	yes	0.642	130	0.226	0.048	0.129
UniRef90_Q4QP33_Haemophilus_parainfluenzae	lesions	yes	0.647	130	0.233	0.048	0.129
UniRef90_Q5FAC0_Neisseria_flavescens	lesions	yes	0.698	130	0.256	0.048	0.129
UniRef90_H1DBB2_Fusobacterium_gonidiaformans	lesions	yes	1.049	130	0.241	0.048	0.129
UniRef90_Q5F5T3_Neisseria_sicca	lesions	yes	0.572	130	0.241	0.048	0.129
UniRef90_B1IA36_Streptococcus_sanguinis	lesions	yes	0.588	130	0.263	0.048	0.129
UniRef90_E5BHF1_Fusobacterium_gonidiaformans	lesions	yes	0.808	130	0.248	0.048	0.130
UniRef90_R7H9W1_Prevotella_sp_CAG_520	lesions	yes	0.838	130	0.233	0.048	0.130
UniRef90_H1DAK4_Fusobacterium_gonidiaformans	lesions	yes	0.895	130	0.226	0.048	0.130
UniRef90_E5BGK8_Fusobacterium_gonidiaformans	lesions	yes	1.101	130	0.263	0.048	0.130
UniRef90_A0A369YHC1_Haemophilus_parainfluenzae	lesions	yes	1.011	130	0.256	0.048	0.130
UniRef90_E5BGC6_Fusobacterium_gonidiaformans	lesions	yes	0.722	130	0.226	0.048	0.130
UniRef90_A0A134B303_Prevotella_bivia	lesions	yes	0.973	130	0.233	0.048	0.130
UniRef90_A0A096BQU1_Prevotella_bivia	lesions	yes	0.775	130	0.226	0.048	0.130
UniRef90_E5BFB4_Fusobacterium_gonidiaformans	lesions	yes	0.986	130	0.241	0.048	0.130
UniRef90_A0A1Y4VSM1_Prevotella_sp_885	lesions	yes	0.546	130	0.271	0.048	0.130
UniRef90_J5W3I5_Fusobacterium_gonidiaformans	lesions	yes	1.082	130	0.248	0.048	0.130
UniRef90_A0A329U7R9_Faecalibacterium_prausnitzii	lesions	yes	0.798	130	0.248	0.048	0.131
UniRef90_I4Z6U5_Prevotella_bivia	lesions	yes	1.245	130	0.421	0.048	0.131
UniRef90_A0A3E4R8K2_Prevotella_sp_AM42_24	lesions	yes	0.720	130	0.248	0.049	0.131
UniRef90_A0A174IFE2_Prevotella_copri	lesions	yes	0.872	130	0.346	0.049	0.131
UniRef90_R7GV62_Prevotella_sp_CAG_520	lesions	yes	0.923	130	0.233	0.049	0.131
UniRef90_A0A154TN48_Neisseria_flavescens	lesions	yes	0.734	130	0.233	0.049	0.132
UniRef90_P45096_Haemophilus_parainfluenzae	lesions	yes	1.058	130	0.323	0.049	0.132
UniRef90_A0A3E4SCB3_Prevotella_copri	lesions	yes	0.601	130	0.263	0.049	0.132
UniRef90_E5BHL0_Fusobacterium_gonidiaformans	lesions	yes	1.056	130	0.263	0.049	0.132
UniRef90_P0A3Q8_Streptococcus_mitis	lesions	yes	1.001	130	0.436	0.049	0.133
UniRef90_I4Z9E3_Prevotella_bivia	lesions	yes	1.022	130	0.233	0.049	0.133
UniRef90_J6H0G7_Fusobacterium_gonidiaformans	lesions	yes	0.977	130	0.226	0.049	0.133
UniRef90_C0M8R8_Streptococcus_oralis	lesions	yes	1.306	130	0.414	0.049	0.133
UniRef90_I4Z8J4_Prevotella_bivia	lesions	yes	0.931	130	0.256	0.049	0.133
UniRef90_Q65RW3_Haemophilus_haemolyticus	lesions	yes	0.714	130	0.248	0.049	0.133
UniRef90_R7HMC5_Prevotella_bivia	lesions	yes	0.608	130	0.271	0.049	0.133
UniRef90_A0A229I9W1_Prevotella_stercorea	lesions	yes	0.560	130	0.241	0.049	0.133
UniRef90_Q5F5R2_Neisseria_flavescens	lesions	yes	0.615	130	0.241	0.049	0.133
UniRef90_A0A264Y466_Prevotella_sp_885	lesions	yes	0.646	130	0.233	0.050	0.133
UniRef90_A0A096BF88_Prevotella_bivia	lesions	yes	1.381	130	0.248	0.050	0.134
UniRef90_A0A133NA10_Fusobacterium_gonidiaformans	lesions	yes	0.977	130	0.226	0.050	0.134
UniRef90_A0A3E4SDB7_Prevotella_copri	lesions	yes	0.756	130	0.271	0.050	0.134
feature	metadata	value	coef	N	Prevalence	pval	qval
feature	metadata	value	coef	N	Prevalence	pval	qval
feature	metadata	value	coef	N	Prevalence	pval	qval
feature	metadata	value	coef	N	Prevalence	pval	qval
feature	metadata	value	coef	N	Prevalence	pval	qval
feature	metadata	value	coef	N	Prevalence	pval	qval
feature	metadata	value	coef	N	Prevalence	pval	qval
feature	metadata	value	coef	N	Prevalence	pval	qval
feature	metadata	value	coef	N	Prevalence	pval	qval
feature	metadata	value	coef	N	Prevalence	pval	qval

feature	metadata	value	coef	N	Prevalence	pval	qval
UniRef90_A0A3E4V1D1_Dorea_longicatena	VL	detectable	1.797	130	0.233	0.001	0.004
UniRef90_G2T3B2_Dorea_longicatena	VL	detectable	1.619	130	0.248	0.002	0.005
UniRef90_D4K4J9_Dorea_longicatena	VL	detectable	1.243	130	0.241	0.002	0.006
UniRef90_D4J5Y4_Dorea_longicatena	VL	detectable	1.708	130	0.241	0.002	0.006
UniRef90_A6NW75_Blautia_wexlerae	VL	detectable	1.119	130	0.226	0.002	0.007
UniRef90_A0A2J4JPV9_Faecalibacterium_prausnitzii	VL	detectable	1.252	130	0.241	0.003	0.007
UniRef90_A0A3E4USV0_Bacteroides_vulgatus	VL	detectable	1.755	130	0.233	0.003	0.009
UniRef90_A0A174T1T5_Bacteroides_vulgatus	VL	detectable	1.402	130	0.233	0.003	0.009
UniRef90_E2ZGT6_Faecalibacterium_prausnitzii	VL	detectable	1.152	130	0.248	0.004	0.009
UniRef90_C4ZEM7_Dorea_longicatena	VL	detectable	1.749	130	0.271	0.004	0.009
UniRef90_A0A174FN26_Blautia_wexlerae	VL	detectable	1.177	130	0.248	0.004	0.010
UniRef90_A8SVC3_Coprococcus_comes	VL	detectable	1.602	130	0.248	0.004	0.011
UniRef90_A0A173VH24_Dorea_longicatena	VL	detectable	1.673	130	0.323	0.005	0.013
UniRef90_J6HFP1_Dorea_formicigenerans	VL	detectable	1.529	130	0.263	0.005	0.013
UniRef90_D6DLD6_Eubacterium_rectale	VL	detectable	1.584	130	0.263	0.005	0.013
UniRef90_C0FXK6_Faecalibacterium_prausnitzii	VL	detectable	1.660	130	0.248	0.005	0.013
UniRef90_C9YMF1_Faecalibacterium_prausnitzii	VL	detectable	1.482	130	0.241	0.005	0.013
UniRef90_A0A2A6Z957_Faecalibacterium_prausnitzii	VL	detectable	0.999	130	0.233	0.006	0.014
UniRef90_R9HXJ2_Bacteroides_vulgatus	VL	detectable	1.756	130	0.241	0.006	0.014
UniRef90_B7ARW3_Dorea_longicatena	VL	detectable	1.355	130	0.301	0.006	0.014
UniRef90_A0A174C1Q1_Blautia_wexlerae	VL	detectable	1.576	130	0.233	0.006	0.015
UniRef90_D4K6T2_Dorea_longicatena	VL	detectable	1.411	130	0.271	0.007	0.016
UniRef90_C5EWP5_Dorea_longicatena	VL	detectable	1.774	130	0.263	0.007	0.016
UniRef90_B0NE07_Faecalibacterium_prausnitzii	VL	detectable	1.495	130	0.316	0.007	0.017
UniRef90_A0A396E717_Bacteroides_vulgatus	VL	detectable	1.479	130	0.233	0.007	0.018
UniRef90_A0A173X477_Dorea_formicigenerans	VL	detectable	1.238	130	0.263	0.008	0.019
UniRef90_A6BEX3_Dorea_longicatena	VL	detectable	1.593	130	0.271	0.008	0.019
UniRef90_R6Q7U2_Prevotella_sp_885	VL	detectable	0.931	130	0.241	0.008	0.019
UniRef90_A0A3E4JCZ2_Bacteroides_vulgatus	VL	detectable	0.900	130	0.233	0.008	0.019
UniRef90_B0PDJ0_Dorea_longicatena	VL	detectable	1.530	130	0.226	0.008	0.020
UniRef90_A0A016CI08_Bacteroides_vulgatus	VL	detectable	1.536	130	0.241	0.009	0.020
UniRef90_G6AXT9_Prevotella_sp_885	VL	detectable	1.519	130	0.256	0.009	0.020
UniRef90_A6BD33_Dorea_formicigenerans	VL	detectable	1.402	130	0.233	0.009	0.020
UniRef90_D1PEN5_Prevotella_copri	VL	detectable	1.383	130	0.263	0.009	0.021
UniRef90_C5EWP5_Dorea_formicigenerans	VL	detectable	1.515	130	0.256	0.009	0.021
UniRef90_Q01911_Bacteroides_vulgatus	VL	detectable	1.568	130	0.248	0.010	0.022
UniRef90_A0A3E2VW53_Faecalibacterium_prausnitzii	VL	detectable	1.604	130	0.263	0.010	0.023
UniRef90_A7AZN6_Coprococcus_comes	VL	detectable	0.946	130	0.308	0.010	0.023
UniRef90_A0A396E3S1_Bacteroides_vulgatus	VL	detectable	1.369	130	0.226	0.010	0.024
UniRef90_A0A174C1Q1_Dorea_longicatena	VL	detectable	1.075	130	0.263	0.011	0.025
UniRef90_A0A2X2VRZ5_Coprococcus_comes	VL	detectable	1.309	130	0.263	0.011	0.025
UniRef90_A0A3E4WLB1_Bacteroides_vulgatus	VL	detectable	1.619	130	0.226	0.011	0.025
UniRef90_A0A173XT59_Bacteroides_vulgatus	VL	detectable	0.521	130	0.271	0.012	0.026
UniRef90_H1LZ69_Dorea_formicigenerans	VL	detectable	1.446	130	0.271	0.012	0.027
UniRef90_A0A3E2TD88_Faecalibacterium_prausnitzii	VL	detectable	1.482	130	0.271	0.012	0.027
UniRef90_C9YMF1_Dorea_longicatena	VL	detectable	1.510	130	0.241	0.012	0.028
UniRef90_E4M058_Faecalibacterium_prausnitzii	VL	detectable	1.405	130	0.308	0.012	0.028
UniRef90_J5UNM3_Dorea_longicatena	VL	detectable	2.129	130	0.263	0.012	0.028
UniRef90_A0A173UXL2_Dorea_longicatena	VL	detectable	1.309	130	0.301	0.013	0.028
UniRef90_A0A174C1Q1_Faecalibacterium_prausnitzii	VL	detectable	1.318	130	0.293	0.013	0.028
UniRef90_E5WU41_Bacteroides_vulgatus	VL	detectable	0.842	130	0.256	0.013	0.028
UniRef90_A0A367FUD5_Faecalibacterium_prausnitzii	VL	detectable	0.916	130	0.248	0.013	0.030
UniRef90_I0TC27_Prevotella_sp_885	VL	detectable	1.298	130	0.308	0.014	0.030
UniRef90_A0A174SPG7_Faecalibacterium_prausnitzii	VL	detectable	1.046	130	0.248	0.015	0.032
UniRef90_A6BKY9_Dorea_longicatena	VL	detectable	1.228	130	0.316	0.015	0.033

UniRef90_A0A1Q6RGY2_Oscillibacter_sp_57_20	VL	detectable	1.233	130	0.233	0.015	0.033
UniRef90_D4JBI5_Holdemanella_biformis	VL	detectable	1.213	130	0.226	0.015	0.033
UniRef90_A0A3E2VW53_Dorea_formicigenerans	VL	detectable	1.265	130	0.248	0.015	0.033
UniRef90_A6BGC3_Eubacterium_rectale	VL	detectable	1.101	130	0.248	0.015	0.033
UniRef90_A0A3E4V1D1_Dorea_formicigenerans	VL	detectable	1.416	130	0.278	0.015	0.034
UniRef90_D4C6A6_Faecalibacterium_prausnitzii	VL	detectable	1.586	130	0.241	0.016	0.034
UniRef90_A0A3E5AS08_Eubacterium_rectale	VL	detectable	1.284	130	0.233	0.016	0.034
UniRef90_A0A015YJI0_Bacteroides_vulgatus	VL	detectable	1.507	130	0.256	0.016	0.034
UniRef90_A0A174NV69_Bacteroides_vulgatus	VL	detectable	0.698	130	0.271	0.016	0.034
UniRef90_A0A3E2XRX6_Dorea_longicatena	VL	detectable	1.473	130	0.248	0.016	0.034
UniRef90_A0A174E4H8_Eubacterium_rectale	VL	detectable	1.457	130	0.233	0.016	0.034
UniRef90_N9XGD7_Dorea_longicatena	VL	detectable	1.501	130	0.278	0.016	0.034
UniRef90_A0A3E4HP53_Bacteroides_vulgatus	VL	detectable	1.255	130	0.263	0.016	0.034
UniRef90_A0A3E2TDA3_Faecalibacterium_prausnitzii	VL	detectable	1.594	130	0.241	0.016	0.034
UniRef90_C7H6T5_Faecalibacterium_prausnitzii	VL	detectable	1.174	130	0.256	0.016	0.035
UniRef90_J6HFP1_Dorea_longicatena	VL	detectable	1.743	130	0.263	0.016	0.035
UniRef90_E4M058_Blautia_wexlerae	VL	detectable	1.107	130	0.256	0.016	0.035
UniRef90_W6NZG1_Bacteroides_vulgatus	VL	detectable	1.353	130	0.248	0.017	0.036
UniRef90_D4IQJ2_Bacteroides_vulgatus	VL	detectable	1.451	130	0.263	0.017	0.036
UniRef90_B3XSP2_Finegoldia_magna	VL	detectable	1.499	130	0.226	0.017	0.036
UniRef90_A0A229I8Z0_Prevotella_copri	VL	detectable	0.972	130	0.301	0.017	0.037
UniRef90_A0A016BWL8_Bacteroides_vulgatus	VL	detectable	1.062	130	0.263	0.017	0.037
UniRef90_P30899_Bacteroides_vulgatus	VL	detectable	0.852	130	0.263	0.017	0.037
UniRef90_M4YXW5_Peptostreptococcus_anaerobius	VL	detectable	1.276	130	0.226	0.018	0.038
UniRef90_B0N9J6_Dorea_longicatena	VL	detectable	1.215	130	0.301	0.018	0.038
UniRef90_D4JS14_Coprococcus_comes	VL	detectable	0.906	130	0.263	0.018	0.038
UniRef90_B6FSS6_Coprococcus_comes	VL	detectable	0.984	130	0.286	0.018	0.038
UniRef90_D4JQL3_Dorea_longicatena	VL	detectable	1.509	130	0.248	0.018	0.039
UniRef90_D4KYI8_Dorea_longicatena	VL	detectable	1.406	130	0.271	0.018	0.039
UniRef90_A0A2A7BGR2_Faecalibacterium_prausnitzii	VL	detectable	1.044	130	0.226	0.019	0.040
UniRef90_A0A3E5DVA8_Bacteroides_vulgatus	VL	detectable	1.079	130	0.263	0.019	0.040
UniRef90_D4WMR9_Bacteroides_vulgatus	VL	detectable	1.638	130	0.256	0.019	0.041
UniRef90_A0A1D7PS63_Escherichia_coli	VL	detectable	1.365	130	0.233	0.019	0.041
UniRef90_F7JMP3_Dorea_longicatena	VL	detectable	1.291	130	0.301	0.019	0.041
UniRef90_D1PEP3_Prevotella_copri	VL	detectable	0.878	130	0.226	0.020	0.042
UniRef90_N9XGD7_Dorea_formicigenerans	VL	detectable	1.298	130	0.241	0.020	0.042
UniRef90_D4K6T2_Eubacterium_rectale	VL	detectable	1.393	130	0.271	0.020	0.043
UniRef90_A0A3E5AS08_Dorea_longicatena	VL	detectable	1.157	130	0.248	0.020	0.043
UniRef90_E1KN21_Bacteroides_vulgatus	VL	detectable	1.450	130	0.271	0.021	0.045
UniRef90_D4KU25_Roseburia_inulinivorans	VL	detectable	1.673	130	0.248	0.021	0.045
UniRef90_D4VAE5_Bacteroides_vulgatus	VL	detectable	0.806	130	0.271	0.022	0.046
UniRef90_A0A0M6WB40_Faecalibacterium_prausnitzii	VL	detectable	1.167	130	0.233	0.022	0.046
UniRef90_A6BGC7_Dorea_formicigenerans	VL	detectable	0.898	130	0.248	0.022	0.046
UniRef90_W0ERF2_Bacteroides_vulgatus	VL	detectable	1.429	130	0.233	0.022	0.046
UniRef90_E4MAW9_Bacteroides_vulgatus	VL	detectable	1.424	130	0.248	0.022	0.046
UniRef90_D4LNK5_Blautia_wexlerae	VL	detectable	0.936	130	0.278	0.022	0.046
UniRef90_R9JR68_Dorea_formicigenerans	VL	detectable	0.978	130	0.263	0.023	0.047
UniRef90_A0A134CL89_Faecalibacterium_prausnitzii	VL	detectable	1.249	130	0.286	0.023	0.048
UniRef90_D4LWJ1_Eubacterium_rectale	VL	detectable	1.322	130	0.226	0.023	0.048
UniRef90_A0A3E5E455_Bacteroides_vulgatus	VL	detectable	1.516	130	0.226	0.023	0.048
UniRef90_C4ZEM7_Eubacterium_rectale	VL	detectable	1.149	130	0.278	0.024	0.049
UniRef90_A0A2A6ZBS6_Faecalibacterium_prausnitzii	VL	detectable	0.961	130	0.286	0.024	0.049
UniRef90_A0A329UA64_Faecalibacterium_prausnitzii	VL	detectable	0.968	130	0.241	0.024	0.049
UniRef90_A0A1L5L421_Coprococcus_comes	VL	detectable	0.811	130	0.248	0.024	0.049
UniRef90_G9RSQ0_Dorea_longicatena	VL	detectable	1.265	130	0.331	0.024	0.049
UniRef90_A0A2A6ZFZ6_Faecalibacterium_prausnitzii	VL	detectable	1.175	130	0.263	0.024	0.050

UniRef90_F7JMP3_Coprococcus_comes	VL	detectable	1.153	130	0.248	0.024	0.050
UniRef90_D4LS50_Dorea_formicigenerans	VL	detectable	0.979	130	0.278	0.024	0.050
UniRef90_I9QVV4_Bacteroides_vulgatus	VL	detectable	1.150	130	0.256	0.024	0.050
UniRef90_A6BD33_Dorea_longicatena	VL	detectable	0.959	130	0.271	0.025	0.051
UniRef90_A0A1E3AP68_Dorea_formicigenerans	VL	detectable	1.079	130	0.263	0.025	0.051
UniRef90_D4LUC8_Dorea_longicatena	VL	detectable	1.629	130	0.301	0.025	0.052
UniRef90_D4JS14_Dorea_formicigenerans	VL	detectable	0.945	130	0.263	0.025	0.053
UniRef90_E6K7K7_Prevotella_sp_885	VL	detectable	1.237	130	0.308	0.025	0.053
UniRef90_A0A0K2HGC7_Bacteroides_vulgatus	VL	detectable	1.304	130	0.226	0.026	0.054
UniRef90_A0A2X2VRZ5_Dorea_longicatena	VL	detectable	1.018	130	0.323	0.026	0.055
UniRef90_R9JR68_Dorea_longicatena	VL	detectable	1.324	130	0.263	0.027	0.055
UniRef90_A0A173X477_Dorea_longicatena	VL	detectable	0.994	130	0.263	0.027	0.055
UniRef90_A0A3E4TAH0_Bacteroides_vulgatus	VL	detectable	1.343	130	0.271	0.027	0.055
UniRef90_C7H6T0_Faecalibacterium_prausnitzii	VL	detectable	1.094	130	0.226	0.027	0.056
UniRef90_C9YMF1_Eubacterium_rectale	VL	detectable	1.170	130	0.248	0.027	0.056
UniRef90_G5SL49_Bacteroides_vulgatus	VL	detectable	1.058	130	0.256	0.027	0.056
UniRef90_W0ERK2_Bacteroides_vulgatus	VL	detectable	1.631	130	0.271	0.027	0.056
UniRef90_UPI000E3F6FFE_Faecalibacterium_prausnitzii	VL	detectable	0.913	130	0.256	0.027	0.056
UniRef90_E5VDI7_Prevotella_copri	VL	detectable	1.708	130	0.286	0.027	0.057
UniRef90_E2ZEI9_Faecalibacterium_prausnitzii	VL	detectable	0.844	130	0.241	0.027	0.057
UniRef90_P10337_Bacteroides_vulgatus	VL	detectable	1.244	130	0.256	0.028	0.057
UniRef90_A0A133PU47_Bacteroides_vulgatus	VL	detectable	1.167	130	0.271	0.028	0.057
UniRef90_A0A173YA79_Dorea_longicatena	VL	detectable	1.571	130	0.293	0.028	0.058
UniRef90_D4JS14_Dorea_longicatena	VL	detectable	1.115	130	0.286	0.029	0.059
UniRef90_A0A2A7ABB4_Faecalibacterium_prausnitzii	VL	detectable	1.028	130	0.226	0.029	0.060
UniRef90_A0A0E2H2A6_Dorea_formicigenerans	VL	detectable	1.243	130	0.233	0.029	0.060
UniRef90_A8SVC3_Eubacterium_rectale	VL	detectable	1.438	130	0.263	0.029	0.060
UniRef90_A0A173YTU2_Faecalibacterium_prausnitzii	VL	detectable	1.325	130	0.293	0.029	0.060
UniRef90_A0A137T0L2_Prevotella_bivia	VL	detectable	0.730	130	0.248	0.030	0.061
UniRef90_A6NXA0_Roseburia_inulinivorans	VL	detectable	0.716	130	0.233	0.030	0.061
UniRef90_D4K9V7_Faecalibacterium_prausnitzii	VL	detectable	1.077	130	0.271	0.030	0.061
UniRef90_A0A173ZZU3_Faecalibacterium_prausnitzii	VL	detectable	0.873	130	0.226	0.030	0.061
UniRef90_A0A069S980_Bacteroides_vulgatus	VL	detectable	1.407	130	0.226	0.030	0.061
UniRef90_J5UNM3_Dorea_formicigenerans	VL	detectable	1.147	130	0.263	0.030	0.062
UniRef90_D4K4J9_Dorea_formicigenerans	VL	detectable	0.893	130	0.263	0.030	0.062
UniRef90_D7NG96_Bacteroides_vulgatus	VL	detectable	0.742	130	0.271	0.030	0.062
UniRef90_A6BGC7_Dorea_longicatena	VL	detectable	0.861	130	0.248	0.030	0.062
UniRef90_D4K6T2_Faecalibacterium_prausnitzii	VL	detectable	1.159	130	0.308	0.030	0.062
UniRef90_A0A127SMK3_Dorea_longicatena	VL	detectable	1.304	130	0.301	0.030	0.062
UniRef90_A8SVC3_Dorea_longicatena	VL	detectable	1.114	130	0.263	0.030	0.062
UniRef90_U2J131_Prevotella_sp_885	VL	detectable	0.787	130	0.278	0.031	0.063
UniRef90_A0A0E9FSE3_Dorea_longicatena	VL	detectable	1.315	130	0.301	0.031	0.063
UniRef90_G2T0F8_Dorea_longicatena	VL	detectable	1.112	130	0.338	0.031	0.063
UniRef90_R6XA92_Prevotella_disiens	VL	detectable	0.984	130	0.248	0.031	0.063
UniRef90_A0A174H8K5_Dorea_formicigenerans	VL	detectable	0.905	130	0.278	0.031	0.064
UniRef90_D4K8L8_Dorea_longicatena	VL	detectable	1.391	130	0.256	0.031	0.064
UniRef90_A0A139LMC3_Bacteroides_vulgatus	VL	detectable	1.595	130	0.256	0.031	0.064
UniRef90_A0A174HEF7_Roseburia_inulinivorans	VL	detectable	0.783	130	0.248	0.032	0.064
UniRef90_D4J4C3_Ruminococcus_torques	VL	detectable	1.168	130	0.271	0.032	0.065
UniRef90_A0A174FN26_Ruminococcus_torques	VL	detectable	0.814	130	0.248	0.032	0.065
UniRef90_A0A374NTF3_Dorea_formicigenerans	VL	detectable	1.261	130	0.256	0.032	0.065
UniRef90_B0P5G9_Eubacterium_rectale	VL	detectable	1.235	130	0.248	0.032	0.065
UniRef90_A8RSS0_Dorea_formicigenerans	VL	detectable	0.986	130	0.256	0.032	0.065
UniRef90_A8S7S0_Faecalibacterium_prausnitzii	VL	detectable	0.994	130	0.226	0.032	0.066
UniRef90_E2ZM22_Dorea_longicatena	VL	detectable	1.264	130	0.263	0.032	0.066
UniRef90_A0A174QSW1_Ruminococcus_torques	VL	detectable	1.180	130	0.323	0.033	0.066

UniRef90_A0A133Y188_Bacteroides_vulgatus	VL	detectable	0.755	130	0.271	0.033	0.067
UniRef90_D4L1Y4_Roseburia_inulinivorans	VL	detectable	1.176	130	0.241	0.033	0.067
UniRef90_A0A396EUL9_Bacteroides_vulgatus	VL	detectable	0.710	130	0.263	0.034	0.069
UniRef90_D4LWI4_Eubacterium_rectale	VL	detectable	1.215	130	0.293	0.034	0.069
UniRef90_A7AZM6_Eubacterium_rectale	VL	detectable	1.233	130	0.256	0.034	0.069
UniRef90_Q650G5_Bacteroides_vulgatus	VL	detectable	1.315	130	0.233	0.034	0.069
UniRef90_Q5LY60_Faecalibacterium_prausnitzii	VL	detectable	1.090	130	0.233	0.034	0.069
UniRef90_A0A395XBK7_Eubacterium_rectale	VL	detectable	0.889	130	0.271	0.034	0.070
UniRef90_A5KIS9_Ruminococcus_torques	VL	detectable	1.116	130	0.241	0.034	0.070
UniRef90_A0A096AHA2_Prevotella_disiens	VL	detectable	1.402	130	0.248	0.034	0.070
UniRef90_D4JZP9_Faecalibacterium_prausnitzii	VL	detectable	0.791	130	0.226	0.035	0.070
UniRef90_C0FQ15_Dorea_longicatena	VL	detectable	0.884	130	0.286	0.035	0.071
UniRef90_C3R255_Bacteroides_vulgatus	VL	detectable	1.348	130	0.256	0.035	0.071
UniRef90_D4J5X8_Eubacterium_rectale	VL	detectable	1.381	130	0.248	0.035	0.071
UniRef90_C0CYA8_Faecalibacterium_prausnitzii	VL	detectable	1.648	130	0.331	0.035	0.072
UniRef90_N9XGD7_Roseburia_inulinivorans	VL	detectable	0.929	130	0.241	0.036	0.072
UniRef90_C5EWQ2_Dorea_longicatena	VL	detectable	1.510	130	0.241	0.036	0.072
UniRef90_A5MC70_Streptococcus_oralis	VL	detectable	2.037	130	0.383	0.036	0.072
UniRef90_D4JS14_Faecalibacterium_prausnitzii	VL	detectable	1.280	130	0.301	0.036	0.073
UniRef90_A0A0P0M3R6_Bacteroides_vulgatus	VL	detectable	1.537	130	0.233	0.036	0.073
UniRef90_A7M5P2_Bacteroides_vulgatus	VL	detectable	0.970	130	0.263	0.036	0.073
UniRef90_A0A395ZWL7_Blautia_wexlerae	VL	detectable	1.396	130	0.233	0.036	0.073
UniRef90_D4K6T2_Ruminococcus_torques	VL	detectable	1.018	130	0.248	0.036	0.073
UniRef90_A0A1X1KXD3_Streptococcus_mitis	VL	detectable	1.922	130	0.376	0.036	0.074
UniRef90_A0A174H8K5_Ruminococcus_torques	VL	detectable	0.943	130	0.248	0.037	0.074
UniRef90_U5FB63_Dorea_longicatena	VL	detectable	1.463	130	0.271	0.037	0.075
UniRef90_A0A396GFV9_Dorea_formicigenerans	VL	detectable	1.224	130	0.331	0.037	0.075
UniRef90_A7M5N8_Bacteroides_vulgatus	VL	detectable	0.809	130	0.263	0.038	0.076
UniRef90_A0A395VSC2_Bacteroides_vulgatus	VL	detectable	0.889	130	0.233	0.038	0.076
UniRef90_A0A229I5D9_Prevotella_sp_CAG_520	VL	detectable	0.845	130	0.233	0.038	0.077
UniRef90_A0A174VAQ9_Prevotella_sp_AM42_24	VL	detectable	0.667	130	0.226	0.038	0.077
UniRef90_A0A229I4C9_Prevotella_copri	VL	detectable	1.126	130	0.226	0.038	0.077
UniRef90_A0A139NVU6_Streptococcus_oralis	VL	detectable	1.268	130	0.263	0.038	0.077
UniRef90_E2ZG82_Faecalibacterium_prausnitzii	VL	detectable	0.983	130	0.241	0.039	0.078
UniRef90_A0A174XP28_Faecalibacterium_prausnitzii	VL	detectable	0.892	130	0.271	0.039	0.078
UniRef90_A0A3E5DRZ1_Prevotella_sp_885	VL	detectable	0.772	130	0.256	0.039	0.079
UniRef90_B0G6W5_Faecalibacterium_prausnitzii	VL	detectable	1.491	130	0.271	0.040	0.080
UniRef90_R0CK04_Roseburia_inulinivorans	VL	detectable	0.915	130	0.263	0.040	0.080
UniRef90_G2T4S6_Dorea_longicatena	VL	detectable	1.383	130	0.241	0.040	0.081
UniRef90_A0A174Y9Z1_Dorea_formicigenerans	VL	detectable	0.978	130	0.271	0.040	0.081
UniRef90_G6AVU2_Prevotella_stercorea	VL	detectable	0.721	130	0.233	0.040	0.081
UniRef90_A0A173RNU0_Faecalibacterium_prausnitzii	VL	detectable	0.921	130	0.256	0.041	0.082
UniRef90_A0A1H9JXP7_Prevotella_sp_885	VL	detectable	0.950	130	0.308	0.041	0.083
UniRef90_A0A395XBK7_Dorea_longicatena	VL	detectable	0.751	130	0.271	0.041	0.083
UniRef90_A0A2A7BEA2_Dorea_longicatena	VL	detectable	0.922	130	0.241	0.041	0.083
UniRef90_W6NZ48_Bacteroides_vulgatus	VL	detectable	1.307	130	0.256	0.042	0.083
UniRef90_Q08425_Bacteroides_vulgatus	VL	detectable	0.914	130	0.271	0.042	0.084
UniRef90_A0A374N425_Bacteroides_vulgatus	VL	detectable	0.713	130	0.271	0.042	0.084
UniRef90_A0A099BUL8_Prevotella_bivia	VL	detectable	1.343	130	0.241	0.042	0.084
UniRef90_A6BGA7_Eubacterium_rectale	VL	detectable	0.835	130	0.248	0.042	0.084
UniRef90_A0A174J6X3_Prevotella_copri	VL	detectable	0.887	130	0.271	0.042	0.085
UniRef90_A0A173QWR1_Dorea_formicigenerans	VL	detectable	0.906	130	0.293	0.042	0.085
UniRef90_I4Z7P1_Bacteroides_vulgatus	VL	detectable	1.148	130	0.241	0.042	0.085
UniRef90_A0A379EFD7_Prevotella_disiens	VL	detectable	1.479	130	0.241	0.043	0.085
UniRef90_A0A379EGH6_Prevotella_disiens	VL	detectable	1.827	130	0.241	0.043	0.086
UniRef90_A0A2A7BB71_Blautia_wexlerae	VL	detectable	1.132	130	0.233	0.043	0.086

UniRef90_D4K6T2_Roseburia_inulinivorans	VL	detectable	0.786	130	0.241	0.043	0.087
UniRef90_A8RDX0_Roseburia_inulinivorans	VL	detectable	0.897	130	0.256	0.043	0.087
UniRef90_A0A396A1S7_Coprococcus_comes	VL	detectable	0.581	130	0.226	0.043	0.087
UniRef90_G5SL50_Bacteroides_vulgatus	VL	detectable	1.067	130	0.263	0.044	0.088
UniRef90_A0A174SJG2_Blautia_wexlerae	VL	detectable	1.013	130	0.241	0.044	0.088
UniRef90_A0A396G9A0_Dorea_formicigenerans	VL	detectable	0.950	130	0.241	0.045	0.090
UniRef90_A0A0P0LKC3_Bacteroides_vulgatus	VL	detectable	1.118	130	0.263	0.046	0.091
UniRef90_A6BGA7_Coprococcus_comes	VL	detectable	0.840	130	0.233	0.046	0.091
UniRef90_Q9XBG8_Bacteroides_vulgatus	VL	detectable	0.763	130	0.271	0.046	0.091
UniRef90_E1GV60_Prevotella_timonensis	VL	detectable	1.743	130	0.248	0.046	0.092
UniRef90_G6AUX6_Prevotella_sp_885	VL	detectable	0.664	130	0.248	0.046	0.092
UniRef90_A6BGA7_Dorea_longicatena	VL	detectable	0.815	130	0.248	0.046	0.093
UniRef90_A0A174E4H8_Faecalibacterium_prausnitzii	VL	detectable	1.115	130	0.263	0.047	0.093
UniRef90_E1KNG8_Bacteroides_vulgatus	VL	detectable	1.048	130	0.233	0.047	0.093
UniRef90_R0CK04_Dorea_longicatena	VL	detectable	1.235	130	0.233	0.047	0.094
UniRef90_E4M058_Eubacterium_rectale	VL	detectable	1.145	130	0.301	0.047	0.094
UniRef90_B7ANM9_Dorea_formicigenerans	VL	detectable	0.831	130	0.248	0.048	0.095
UniRef90_A0A3E4SB49_Prevotella_copri	VL	detectable	0.715	130	0.263	0.048	0.096
UniRef90_A4EBI3_Collinsella_aerofaciens	VL	detectable	1.197	130	0.293	0.048	0.096
UniRef90_A0A174C1Q1_Roseburia_inulinivorans	VL	detectable	0.801	130	0.256	0.048	0.096
UniRef90_A0A395ZWL7_Dorea_formicigenerans	VL	detectable	0.806	130	0.241	0.049	0.097
UniRef90_A8SHC7_Eubacterium_rectale	VL	detectable	0.889	130	0.248	0.049	0.098
UniRef90_D4LWI4_Faecalibacterium_prausnitzii	VL	detectable	1.269	130	0.323	0.050	0.099
UniRef90_A0A0E9FSE3_Eubacterium_rectale	VL	detectable	1.080	130	0.256	0.050	0.099
UniRef90_A0A3E4SDQ7_Prevotella_copri	VL	detectable	0.808	130	0.241	0.050	0.099
UniRef90_G6AY02_Prevotella_sp_885	VL	detectable	0.737	130	0.286	0.050	0.099
UniRef90_C7H7J2_Faecalibacterium_prausnitzii	VL	detectable	0.785	130	0.226	0.050	0.099
UniRef90_A0A174VDA7_Dorea_formicigenerans	VL	detectable	1.058	130	0.293	0.050	0.099
UniRef90_A0A0U0KMK0_Streptococcus_mitis	VL	detectable	-1.156	130	0.286	0.050	0.099
UniRef90_A0A098Z1S5_Streptococcus_oralis	VL	detectable	-1.118	130	0.271	0.050	0.099
UniRef90_A0A0E9GUR6_Streptococcus_oralis	VL	detectable	-1.151	130	0.316	0.050	0.099
UniRef90_Q9K1C1_Neisseria_flavescens	VL	detectable	-0.867	130	0.248	0.049	0.097
UniRef90_A0A0B7M484_Streptococcus_infantis	VL	detectable	-0.874	130	0.241	0.049	0.097
UniRef90_B5E2H6_Streptococcus_infantis	VL	detectable	-0.945	130	0.241	0.048	0.096
UniRef90_A0A098Z5C2_Streptococcus_oralis	VL	detectable	-1.179	130	0.248	0.047	0.095
UniRef90_A0A3A5CDW0_Neisseria_sicca	VL	detectable	-0.738	130	0.226	0.047	0.094
UniRef90_Q97R66_Streptococcus_oralis	VL	detectable	-0.900	130	0.263	0.047	0.093
UniRef90_M5JV55_Streptococcus_mitis	VL	detectable	-0.879	130	0.293	0.047	0.093
UniRef90_A0A0F2E304_Streptococcus_mitis	VL	detectable	-1.026	130	0.278	0.047	0.093
UniRef90_E0Q3M9_Streptococcus_infantis	VL	detectable	-0.838	130	0.286	0.046	0.092
UniRef90_Q9JTI8_Neisseria_flavescens	VL	detectable	-1.025	130	0.226	0.046	0.092
UniRef90_E9FPX8_Streptococcus_infantis	VL	detectable	-0.698	130	0.286	0.046	0.092
UniRef90_P63373_Streptococcus_oralis	VL	detectable	-1.333	130	0.241	0.046	0.092
UniRef90_Q97QG6_Streptococcus_mitis	VL	detectable	-1.088	130	0.346	0.046	0.091
UniRef90_F9HAP5_Streptococcus_mitis	VL	detectable	-1.903	130	0.301	0.046	0.091
UniRef90_A0A139PUH8_Streptococcus_oralis	VL	detectable	-1.029	130	0.263	0.046	0.091
UniRef90_A0A154TFR0_Neisseria_flavescens	VL	detectable	-0.900	130	0.241	0.045	0.090
UniRef90_A0A098ZQD6_Streptococcus_oralis	VL	detectable	-1.261	130	0.338	0.045	0.090
UniRef90_Q04KG2_Streptococcus_oralis	VL	detectable	-1.783	130	0.353	0.044	0.088
UniRef90_I0J9D9_Streptococcus_infantis	VL	detectable	-0.971	130	0.233	0.044	0.088
UniRef90_A0A0B7LIN4_Streptococcus_mitis	VL	detectable	-0.636	130	0.263	0.044	0.087
UniRef90_Q04HV1_Streptococcus_oralis	VL	detectable	-1.461	130	0.293	0.044	0.087
UniRef90_P63384_Streptococcus_infantis	VL	detectable	-0.837	130	0.263	0.044	0.087
UniRef90_V8IAL5_Streptococcus_infantis	VL	detectable	-0.701	130	0.248	0.043	0.087
UniRef90_S7YT34_Streptococcus_infantis	VL	detectable	-0.771	130	0.233	0.043	0.087
UniRef90_A8AZ08_Streptococcus_infantis	VL	detectable	-0.984	130	0.248	0.043	0.086

UniRef90_Q9K177_Neisseria_flavescens	VL	detectable	-1.288	130	0.226	0.043	0.086
UniRef90_D2ZSG6_Neisseria_flavescens	VL	detectable	-0.988	130	0.271	0.043	0.086
UniRef90_A0A139PUH8_Streptococcus_mitis	VL	detectable	-1.146	130	0.271	0.043	0.085
UniRef90_P0A4M7_Streptococcus_mitis	VL	detectable	-1.068	130	0.271	0.042	0.084
UniRef90_A9M125_Neisseria_flavescens	VL	detectable	-1.163	130	0.226	0.040	0.081
UniRef90_A0A0T9F4L8_Streptococcus_mitis	VL	detectable	-1.265	130	0.256	0.040	0.081
UniRef90_E8JY29_Streptococcus_infantis	VL	detectable	-1.335	130	0.248	0.040	0.081
UniRef90_A0A0I6XZA2_Streptococcus_oralis	VL	detectable	-0.964	130	0.308	0.040	0.081
UniRef90_A8AWM9_Streptococcus_oralis	VL	detectable	-1.492	130	0.293	0.040	0.080
UniRef90_Q97QP2_Streptococcus_oralis	VL	detectable	-1.184	130	0.323	0.040	0.080
UniRef90_D6ZMN9_Streptococcus_mitis	VL	detectable	-0.783	130	0.278	0.039	0.078
UniRef90_Q8DRE1_Streptococcus_oralis	VL	detectable	-1.461	130	0.256	0.039	0.078
UniRef90_B8ZJN3_Streptococcus_oralis	VL	detectable	-1.740	130	0.353	0.039	0.078
UniRef90_Q97PM1_Streptococcus_oralis	VL	detectable	-1.014	130	0.248	0.038	0.077
UniRef90_A0A0B7M2D5_Streptococcus_oralis	VL	detectable	-1.572	130	0.308	0.038	0.076
UniRef90_A0A0F2E1Y9_Streptococcus_oralis	VL	detectable	-1.500	130	0.323	0.038	0.076
UniRef90_Q9K1C1_Neisseria_sicca	VL	detectable	-0.849	130	0.241	0.037	0.076
UniRef90_I1ZK55_Streptococcus_mitis	VL	detectable	-0.982	130	0.263	0.037	0.075
UniRef90_A0A3A5DM74_Neisseria_flavescens	VL	detectable	-0.961	130	0.286	0.037	0.074
UniRef90_A0A133S0N3_Streptococcus_mitis	VL	detectable	-0.769	130	0.248	0.037	0.074
UniRef90_A0A150NNZ5_Streptococcus_mitis	VL	detectable	-1.387	130	0.248	0.036	0.073
UniRef90_E9FPR1_Streptococcus_oralis	VL	detectable	-1.936	130	0.353	0.036	0.073
UniRef90_A3CPH6_Streptococcus_oralis	VL	detectable	-1.179	130	0.278	0.036	0.073
UniRef90_A0A062WMJ4_Streptococcus_oralis	VL	detectable	-1.276	130	0.271	0.036	0.072
UniRef90_Q5M434_Streptococcus_mitis	VL	detectable	-1.816	130	0.263	0.036	0.072
UniRef90_A0A139QLB4_Streptococcus_mitis	VL	detectable	-1.044	130	0.316	0.035	0.071
UniRef90_I0SCW7_Streptococcus_mitis	VL	detectable	-0.593	130	0.278	0.035	0.071
UniRef90_A9M1L0_Neisseria_flavescens	VL	detectable	-1.213	130	0.233	0.035	0.071
UniRef90_Q9JYY0_Neisseria_sicca	VL	detectable	-0.828	130	0.226	0.035	0.070
UniRef90_Q3K3Q0_Streptococcus_mitis	VL	detectable	-1.704	130	0.331	0.034	0.070
UniRef90_X5EN23_Neisseria_flavescens	VL	detectable	-1.042	130	0.248	0.034	0.069
UniRef90_D2ZSG6_Rothia_dentocariosa	VL	detectable	-0.887	130	0.241	0.034	0.068
UniRef90_P65504_Streptococcus_oralis	VL	detectable	-0.804	130	0.241	0.034	0.068
UniRef90_P0A0Y5_Neisseria_flavescens	VL	detectable	-0.917	130	0.226	0.033	0.068
UniRef90_Q04K26_Streptococcus_mitis	VL	detectable	-1.388	130	0.316	0.033	0.068
UniRef90_A0A150NX26_Streptococcus_infantis	VL	detectable	-0.993	130	0.241	0.033	0.068
UniRef90_A0A064BXF0_Streptococcus_oralis	VL	detectable	-1.602	130	0.346	0.033	0.067
UniRef90_A0A0T8C7T0_Streptococcus_oralis	VL	detectable	-1.180	130	0.286	0.032	0.066
UniRef90_Q5F835_Neisseria_sicca	VL	detectable	-1.010	130	0.226	0.032	0.065
UniRef90_A8AZ08_Streptococcus_mitis	VL	detectable	-2.020	130	0.376	0.032	0.065
UniRef90_F9Q0B2_Streptococcus_oralis	VL	detectable	-1.805	130	0.331	0.031	0.064
UniRef90_D0W0B3_Neisseria_flavescens	VL	detectable	-1.161	130	0.226	0.031	0.064
UniRef90_Q8DR23_Streptococcus_oralis	VL	detectable	-0.829	130	0.256	0.031	0.063
UniRef90_P25821_Neisseria_flavescens	VL	detectable	-1.228	130	0.241	0.031	0.063
UniRef90_P0DB88_Streptococcus_infantis	VL	detectable	-1.173	130	0.256	0.031	0.063
UniRef90_A0A150NG45_Streptococcus_oralis	VL	detectable	-0.838	130	0.301	0.030	0.062
UniRef90_E0Q354_Streptococcus_oralis	VL	detectable	-1.321	130	0.323	0.029	0.060
UniRef90_A0A0B7LB97_Streptococcus_infantis	VL	detectable	-0.704	130	0.241	0.029	0.060
UniRef90_A0A0F2DFD2_Streptococcus_mitis	VL	detectable	-0.862	130	0.248	0.028	0.059
UniRef90_F9LYG9_Streptococcus_mitis	VL	detectable	-1.239	130	0.256	0.028	0.058
UniRef90_A0A0F2DPR5_Streptococcus_mitis	VL	detectable	-1.422	130	0.323	0.028	0.058
UniRef90_X8HGW1_Streptococcus_oralis	VL	detectable	-1.492	130	0.241	0.028	0.057
UniRef90_A0A0Y0HIE3_Streptococcus_oralis	VL	detectable	-1.282	130	0.271	0.028	0.057
UniRef90_E5UI13_Neisseria_sicca	VL	detectable	-1.100	130	0.248	0.027	0.057
UniRef90_P65443_Streptococcus_oralis	VL	detectable	-0.884	130	0.301	0.027	0.056
UniRef90_P66114_Streptococcus_oralis	VL	detectable	-1.592	130	0.263	0.026	0.054

UniRef90_A0A0T9H215_Streptococcus_mitis	VL	detectable	-0.878	130	0.301	0.025	0.052
UniRef90_Q04K52_Streptococcus_oralis	VL	detectable	-1.496	130	0.263	0.025	0.052
UniRef90_C6M9J2_Neisseria_flavescens	VL	detectable	-1.354	130	0.226	0.025	0.051
UniRef90_D3A106_Neisseria_flavescens	VL	detectable	-0.942	130	0.233	0.024	0.050
UniRef90_W1UT46_Streptococcus_oralis	VL	detectable	-1.431	130	0.271	0.024	0.050
UniRef90_A8AZD5_Streptococcus_oralis	VL	detectable	-1.154	130	0.338	0.023	0.049
UniRef90_Q9K0Y2_Neisseria_flavescens	VL	detectable	-1.308	130	0.226	0.023	0.048
UniRef90_Q5F973_Neisseria_sicca	VL	detectable	-0.900	130	0.233	0.022	0.047
UniRef90_A0A064BZF6_Streptococcus_oralis	VL	detectable	-0.858	130	0.271	0.022	0.047
UniRef90_A0A062WUM5_Streptococcus_oralis	VL	detectable	-0.891	130	0.301	0.022	0.046
UniRef90_Q04JQ8_Streptococcus_infantis	VL	detectable	-0.948	130	0.248	0.022	0.046
UniRef90_P35594_Streptococcus_infantis	VL	detectable	-1.044	130	0.241	0.022	0.046
UniRef90_Q97QG6_Streptococcus_oralis	VL	detectable	-0.862	130	0.271	0.022	0.046
UniRef90_F9PXD6_Streptococcus_mitis	VL	detectable	-0.823	130	0.271	0.022	0.046
UniRef90_P0A3Q0_Streptococcus_infantis	VL	detectable	-1.149	130	0.263	0.021	0.045
UniRef90_Q47810_Streptococcus_mitis	VL	detectable	-1.889	130	0.286	0.021	0.045
UniRef90_Q04LL3_Streptococcus_infantis	VL	detectable	-1.015	130	0.256	0.021	0.043
UniRef90_A0A0U0KE62_Streptococcus_infantis	VL	detectable	-1.086	130	0.241	0.020	0.043
UniRef90_F9HAP5_Streptococcus_oralis	VL	detectable	-1.613	130	0.286	0.019	0.041
UniRef90_R6A3D4_Streptococcus_mitis	VL	detectable	-1.436	130	0.316	0.019	0.040
UniRef90_A0A0B7LIN4_Streptococcus_oralis	VL	detectable	-1.031	130	0.271	0.019	0.040
UniRef90_Q9JZN9_Neisseria_flavescens	VL	detectable	-1.481	130	0.241	0.018	0.038
UniRef90_Q97SC7_Streptococcus_oralis	VL	detectable	-0.939	130	0.248	0.017	0.037
UniRef90_A0A081PSA5_Streptococcus_oralis	VL	detectable	-1.594	130	0.248	0.017	0.037
UniRef90_F2B9R9_Neisseria_flavescens	VL	detectable	-1.336	130	0.241	0.017	0.037
UniRef90_A0A133ZTR9_Gemella_haemolysans	VL	detectable	-0.955	130	0.226	0.017	0.036
UniRef90_C1CMA0_Streptococcus_infantis	VL	detectable	-1.115	130	0.233	0.016	0.035
UniRef90_A0A0B7M047_Streptococcus_oralis	VL	detectable	-1.905	130	0.316	0.016	0.035
UniRef90_A0A064BX67_Streptococcus_oralis	VL	detectable	-1.131	130	0.271	0.016	0.035
UniRef90_Q04LC5_Streptococcus_oralis	VL	detectable	-1.359	130	0.256	0.015	0.033
UniRef90_P95379_Neisseria_flavescens	VL	detectable	-1.268	130	0.263	0.015	0.033
UniRef90_A0A0F2DFD2_Streptococcus_oralis	VL	detectable	-0.852	130	0.241	0.015	0.033
UniRef90_A0A095ZDN4_Streptococcus_oralis	VL	detectable	-1.249	130	0.286	0.015	0.032
UniRef90_A8AZM0_Streptococcus_infantis	VL	detectable	-1.411	130	0.241	0.015	0.032
UniRef90_M5K861_Streptococcus_oralis	VL	detectable	-1.991	130	0.331	0.014	0.031
UniRef90_Q8DS22_Streptococcus_oralis	VL	detectable	-1.466	130	0.316	0.014	0.030
UniRef90_D4FT63_Streptococcus_infantis	VL	detectable	-1.124	130	0.263	0.014	0.030
UniRef90_Q97R65_Streptococcus_oralis	VL	detectable	-1.760	130	0.248	0.013	0.030
UniRef90_D6ZMN9_Streptococcus_oralis	VL	detectable	-1.216	130	0.301	0.013	0.030
UniRef90_C6M5M2_Neisseria_flavescens	VL	detectable	-1.087	130	0.233	0.013	0.029
UniRef90_L1NWJ9_Neisseria_sicca	VL	detectable	-0.999	130	0.241	0.012	0.027
UniRef90_C6M7N5_Neisseria_flavescens	VL	detectable	-1.489	130	0.286	0.012	0.026
UniRef90_C6M392_Neisseria_sicca	VL	detectable	-1.214	130	0.241	0.012	0.026
UniRef90_A0A133RZC7_Streptococcus_mitis	VL	detectable	-1.905	130	0.316	0.011	0.025
UniRef90_P0A4N1_Streptococcus_oralis	VL	detectable	-0.958	130	0.323	0.010	0.023
UniRef90_I1ZK55_Streptococcus_oralis	VL	detectable	-2.295	130	0.308	0.010	0.022
UniRef90_Q97QS5_Streptococcus_oralis	VL	detectable	-1.637	130	0.323	0.010	0.022
UniRef90_A0A0T8AEZ7_Streptococcus_mitis	VL	detectable	-1.430	130	0.241	0.008	0.020
UniRef90_D3A1Q6_Neisseria_flavescens	VL	detectable	-1.768	130	0.226	0.003	0.009
