# Supplementary material for: Oral and anal microbiome from HIV-exposed individuals: role of host-associated factors in taxa composition and metabolic pathways
Source: NPJ Biofilms Microbiomes. 2023 Jul 12;9:48. doi: 10.1038/s41522-023-00413-4 (PMC10338440; doi:10.1038/s41522-023-00413-4)
Supplement: Supplementary file 2 — Reporting Summary [file 41522_2023_413_MOESM2_ESM.pdf]

Corresponding author(s): DAPR NPJBIOFILMS-02079R1
Ezequiel Lacunza
Martín C Abba

Last updated by author(s): Jun 15, 2023

Reporting Summary

Nature Portfolio wishes to improve the reproducibility of the work that we publish. This form provides structure for consistency and transparency in reporting. For further information on Nature Portfolio policies, see our Editorial Policies and the Editorial Policy Checklist.

Statistics

For all statistical analyses, confirm that the following items are present in the figure legend, table legend, main text, or Methods section.

n/a	Confirmed	
☐	☒	The exact sample size (*n*) for each experimental group/condition, given as a discrete number and unit of measurement
☐	☒	A statement on whether measurements were taken from distinct samples or whether the same sample was measured repeatedly
☐	☒	The statistical test(s) used AND whether they are one- or two-sided<br>*Only common tests should be described solely by name; describe more complex techniques in the Methods section.*
☐	☒	A description of all covariates tested
☐	☒	A description of any assumptions or corrections, such as tests of normality and adjustment for multiple comparisons
☐	☒	A full description of the statistical parameters including central tendency (e.g. means) or other basic estimates (e.g. regression coefficient) AND variation (e.g. standard deviation) or associated estimates of uncertainty (e.g. confidence intervals)
☒	☐	For null hypothesis testing, the test statistic (e.g. *F*, *t*, *r*) with confidence intervals, effect sizes, degrees of freedom and *P* value noted<br>*Give P values as exact values whenever suitable.*
☒	☐	For Bayesian analysis, information on the choice of priors and Markov chain Monte Carlo settings
☒	☐	For hierarchical and complex designs, identification of the appropriate level for tests and full reporting of outcomes
☒	☐	Estimates of effect sizes (e.g. Cohen's *d*, Pearson's *r*), indicating how they were calculated

Our web collection on statistics for biologists contains articles on many of the points above.

Software and code

Policy information about availability of computer code

Data collection	DNA samples were processed for shotgun metagenomics sequencing.<br>We performed 100 nt paired-end sequencing using an Illumina NovaSeq 6000 System and obtained about 80 million reads per sample.<br>Raw data (510 GiB) was downloaded from Illumina BaseSpace to our dedicated server using BaseMount tools, and processed with the bioBakery toolkits.<br>Quality control on metagenomics sequencing data (Fastq files), were performed with KneadData tools, which separate the human (host) and the non-human (microbiome) reads for further QC-based filtering.
Data analysis	For determining the relative differential abundance and the multivariable association between subjects' metadata and microbial features, we used the MaAsLin2 package from the bioBakery suite in R/Bioconductor<br>Whole genome metagenomics pathway analysis was adopted in the HMP Unified Metabolic Analysis Network 3 (HUMAnN3) pipeline to assess the potential differences in metabolic pathways. HUMAnN3 identifies the species profile from metagenomics shotgun sequencing data, aligns reads to their pan-genomes, performs translated search on unclassified reads, and quantifies gene families and pathways<br>For profiling the composition of microbial communities at the species-level, we run MetaPhlAn 3.0 using default parameters. MetaPhlAn relies on unique clade-specific marker genes identified from ~17,000 reference genomes (~13,500 bacterial and archaeal, ~3,500 viral, and ~110 eukaryotic). This bioinformatics tool provides the relative abundances of each microbial clade with species-level resolution.

For manuscripts utilizing custom algorithms or software that are central to the research but not yet described in published literature, software must be made available to editors and reviewers. We strongly encourage code deposition in a community repository (e.g. GitHub). See the Nature Portfolio guidelines for submitting code & software for further information.

Data

Policy information about availability of data

All manuscripts must include a data availability statement. This statement should provide the following information, where applicable:

- Accession codes, unique identifiers, or web links for publicly available datasets
- A description of any restrictions on data availability
- For clinical datasets or third party data, please ensure that the statement adheres to our policy

All data generated or analyzed during this study are included in this published article [and its supplementary information files].

Research involving human participants, their data, or biological material

Policy information about studies with human participants or human data. See also policy information about sex, gender (identity/presentation), and sexual orientation and race, ethnicity and racism.

Reporting on sex and gender	The Gender Identity Law, Number 26743, was passed in Argentina in May 2012 to guarantee the free development of individuals according to their gender identity, whether their chosen gender is the same to or different from their sex assigned at birth. This law not only guarantees the rectification of the sex registered and the change of name and sex in all documents that certify their identity but also permits access to comprehensive healthcare, hormone treatments, and partial or total surgical interventions without requiring judicial or administrative authorization. These procedures only require the patient's informed consent.<br>This study included an Argentinian cohort of Transgender Women and Men who have sex with Men. The terms were asssigned for the purpose of the research according to the gender identity provided by the participants.
Reporting on race, ethnicity, or other socially relevant groupings	This study did not take into consideration or report any race, ethnicity, or other socially relevant grouping variables.
Population characteristics	The study collected data from participants on clinical variables, sexual practices and consumption habits. Clinical data included gender (Men who have Sex with Men and Transgender Women), age (Mean 34.5; Median 33), HIV status, current CD4+ T-cell counts, current viral load (VL), use of antiretroviral therapy (ART), and anal cytology results indicating the presence of epithelial lesions, specifically Atypical Squamous Cells of Undetermined Significance (ASCUS), Low-grade Squamous Intraepithelial Lesion (LSIL), and High-Grade Squamous Intraepithelial Lesion (HSIL). Sexual behavior variables comprised Age at First Sexual Intercourse (AFSI), Age of Anal Sex Initiation (AASI), Age of Oral Sex Initiation (AOFSI), Number of Sexual Partners in the Last Month (NSPLM), Number of Sexual Partners in Life (NSPL), Condom Use in Anal (CUAS) and Oral Sex (CUOS), and whether participants are or have been Commercial Sex Workers (CSW). It is important to note that none of the TGW included in the study have undergone gender-affirming surgery. Consumption habits included Alcohol, Tobacco, and intravenous or non-intravenous drug use.
Recruitment	This study included an Argentinian cohort of HIV-positive and HIV-negative cases of Transgender Women (TGW) and Men who have Sex with Men (MSM) recruited at Fundación Huésped, Buenos Aires, Argentina. Participants were age >18 at the beginning of the study (median: 33 years; range: 19-58 years). A total of 130 samples (59 oral swabs and 71 anal swabs) were obtained from 47 MSM and 31 TGW. Of the 78 participants, 50 were HIV-positive and 28 were HIV-negative. When recruited, most of the participants with HIV were on antiretroviral therapy (ART). Samples were collected in Qiagen specimen collection device (Qiagen, USA) by qualified staff at Fundación Huésped.
Ethics oversight	All participants signed informed consent before being involved in the project. The study was approved by the institutional review board (Comité de Bioética, Fundación Huésped).<br>Fundación Huésped is an organization of Argentine civil society that has performed scientific and social work since 1989 to promote a fair, healthy, and sustainable society with access to human rights, care, and services. The organization promotes individual rights and access to health care in the absence of discrimination. Fundación Huésped strives to be a national leader organization, with regional and international perspective, and a public health reference with a focus on sexual and reproductive health, HIV/AIDS, and other transmissible diseases.<br>Through strategic alliances with civil society, the State, the private sector, and academic institutions, Fundación Huésped is a leading organization in advocacy, clinical, epidemiological, and social research; in technological training; and in dissemination of these ideas.

Note that full information on the approval of the study protocol must also be provided in the manuscript.

Field-specific reporting

Please select the one below that is the best fit for your research. If you are not sure, read the appropriate sections before making your selection.

☒ Life sciences ☐ Behavioural & social sciences ☐ Ecological, evolutionary & environmental sciences

For a reference copy of the document with all sections, see nature.com/documents/nr-reporting-summary-flat.pdf

Life sciences study design

All studies must disclose on these points even when the disclosure is negative.

Sample size	A comprehensive study was conducted involving 78 participants, consisting of 47 MSM (men who have sex with men) and 31 TGW (transgender women). A total of 130 samples were collected, comprising 59 oral swabs and 71 anal swabs. The sample size was carefully determined to ensure a minimum of 30 samples per group, taking into account practical considerations such as processing costs and statistical power. This approach allowed for a well-balanced and efficient study design, optimizing resources without compromising the validity of the results.
Data exclusions	No data were excluded from the analysis
Replication	We used default parameters for normalization (TSS method), transformation (Log), analysis method (LM), correction method (BH), and significance threshold (q-value<0.25). Although association in MaAsLin 2.0 is considered significant at a q-value of below 0.25, a cut-off used in previous microbiome studies, for more stringency we selected the associated features (species, pathways, gene families) with a q-value < 0.15 (nominal p-value<0.05)]. Other Variables were included in the multivariate model to account for their potential impact on the microbiome as confounding factors. However, they were not the primary focus of this study and were only considered as covariates to adjust for additional factors.
Randomization	To establish the groups we assigned them based on the gender, HIV status and the location site of the sample origin. Furthermore a randomization process was employed based on the taxonomic profile of bacterial and virus species found in the samples. The unsupervised grouping method categorized the samples into iIn order to establish the groups we assigned them based on the HIV status and the location site of the sample origin. Furthermore a randomization process was employed based on the taxonomic profile of bacterial and virus species found in the samples. The unsupervised grouping method categorized the samples into their respective oral and anal sites. Furthermore, covariates were carefully incorporated into the model to ensure adjustments were made appropriately. This approach not only ensured a balanced representation of the different sites but also accounted for potential confounding factors, thus enhancing the accuracy and reliability of the study's findings.
Blinding	Since all the analyses conducted in this study rely on the implementation of standardized algorithms, the concept of blinding is not applicable or relevant. The use of these established and widely accepted algorithms ensures consistency and objectivity throughout the analysis process.

Reporting for specific materials, systems and methods

We require information from authors about some types of materials, experimental systems and methods used in many studies. Here, indicate whether each material, system or method listed is relevant to your study. If you are not sure if a list item applies to your research, read the appropriate section before selecting a response.

Materials & experimental systems

n/a	Involved in the study	
☒	☐	Antibodies
☒	☐	Eukaryotic cell lines
☒	☐	Palaeontology and archaeology
☒	☐	Animals and other organisms
☐	☒	Clinical data
☒	☐	Dual use research of concern
☒	☐	Plants

Methods

n/a	Involved in the study	
☒	☐	ChIP-seq
☒	☐	Flow cytometry
☒	☐	MRI-based neuroimaging

Clinical data

Policy information about clinical studies

All manuscripts should comply with the ICMJE guidelines for publication of clinical research and a completed CONSORT checklist must be included with all submissions.

Clinical trial registration	NA
Study protocol	NA
Data collection	NA
Outcomes	NA
